# The ‘bIUreactor’: An Open-Source 3D Tissue Research Platform

**DOI:** 10.1007/s10439-024-03481-5

**Published:** 2024-03-26

**Authors:** Elizabeth Butch, Matthew Prideaux, Mark Holland, Justin-Thuy Phan, Cole Trent, Victor Soon, Gary Hutchins, Lester Smith

**Affiliations:** 1 Department of Radiology and Imaging Sciences, Indiana University School of Medicine, Indianapolis, IN USA; 2 Indiana Center for Musculoskeletal Health, Indiana University School of Medicine, Indianapolis, IN USA; 3 Smith BioFab Lab, Department of Radiology and Imaging Sciences, Indiana University School of Medicine, Indianapolis, IN USA

**Keywords:** Biofabrication, Bioprinting, Bioreactor, 3D printing, 3D tissue, Hardware, Open-source, Tissue culture, Tissue engineering

## Abstract

We developed the open-source bIUreactor research platform for studying 3D structured tissues. The versatile and modular platform allows a researcher to generate 3D tissues, culture them with oxygenated perfusion, and provide cyclic loading, all in their own lab (*in laboratorium*) for an all in cost of $8,000 including 3D printer, printing resin, and electronics. We achieved this by applying a design philosophy that leverages 3D printing, open-source software and hardware, and practical techniques to produce the following: 1. perfusible 3D tissues, 2. a bioreactor chamber for tissue culture, 3. a module for applying cyclic compression, 4. a peristaltic pump for providing oxygenated perfusion to 3D tissues, 5. motor control units, and 6. open-source code for running the control units. By making it widely available for researchers to investigate 3D tissue models and easy for them to use, we intend for the bIUreactor to democratize 3D tissue research, therefore increasing the pace and scale of biomedical research discoveries using 3D tissue models.

**Supplementary Information:**

The online version contains supplementary material available at 10.1007/s10439-024-03481-5.

## Introduction

Tissue Engineering is the production (biofabrication) of functional tissues comprised cells, biomaterials (synthetic, reconstituted, and/or cell secreted). A critical element of tissue engineering is the bioreactor which is necessary to biofabricate, contain, and culture the tissues with repeatable predictably. The complex demands of combined perfusion and mechanical stimulation, usability, reusability, and sterilization result in expensive bioreactors that are cumbersome to use and possess limited features. We have developed the bIUreactor, a modular platform system that a user can, in their own lab, build, install, and use to research 3D-structured tissues. Our open-source platform is intended to democratize 3D tissue research by making it widely available and easily accessible for biomedical researchers. The bIUreactor was developed using a design doctrine intended to result in easy to fabricate and operate culture devices (Supplement 1).

This platform is replete with the following features*:

1. A device and method the researcher can use to form spheroid microtissues.
2. A Flow Circuit to contain spheroid-based tissues during perfusion-supported formation and maturation.
  - Silicone seals, gaskets, and grommets made from 3D printed molds.
  - The entire flow circuit can be autoclaved as an assembly, minimizing contamination risk.
3. A Compressor Module for aseptically applying cyclic mechanical compression at user selected rates and displacements.
4. A Peristaltic Pump capable of safely perfusing tissues at consistent rates.
  - A single pump motor operating up to 4 pump heads at a time.
5. Needle-Free Valves that allow the aseptic addition and removal of media from the bioreactor fluid circuit.
6. Aseptic tissue observation during culture.
7. Control systems for controlling the motors powering the pump and the compression module.
  - Solder-Free Circuit Assembly
  - Pre-written code
8. 3D printed boxes for containing pump and compressor motor control units
9. A list of the 3D printing equipment and materials needed. (Supplement 5)
10. A list of the tubing and silicone as well as source vendors. (Supplement 5)
11. A list of the electronic components and vendor sources. (Supplement 5)
12. A cost breakdown of all the components so that researchers can integrate this system easily into their budget. (Supplement 5)
13. Detailed fabrication, assembly, installation, calibration, and use instructions, with images. (Supplement 4)
14. PreForm files of all components (available upon request through GitHub)

*Parts and components contacting cells are 3D printed from autoclavable and reusable biomaterial resin.

### Transition from 2D to 3D Tissue Culture Models Should be a Research Priority

In natural tissues, vascular perfusion dynamically transports nutrient-enriched blood deep into their 3D structure to provide essential support for cell metabolism [1]. Perfusion is required for most tissue metabolism, formation and health, beginning at embryogenesis and required constantly throughout life. Cellular and extracellular matrix (ECM) properties observed in natural tissues are mostly conserved or reapproximated in biofabricated 3D tissues but are lost or limited in 2D culture (the current research standard) [2–6]. To produce research models accurately recreating natural tissue properties and responses, transition from 2D to 3D tissue culture models should be a biomedical research priority [7–10]. Cell-dense 3D-structured tissues, however, pose the following practical challenges:

1. Cells in human tissues are densely packed together within extracellular matrix (ECM) requiring persistent and metabolite-rich delivery of nutrients (perfusion) to the cells, to support metabolism.
2. Biofabricated tissues sometimes cannot withstand the culture environment.
3. ECM restricts cellular access to nutrients, which may change their metabolic demands and resultant activity.
4. Tissue density and thickness must be taken into consideration as they can present challenges for some imaging modalities.

The dearth of 3D tissue models/platforms contributes to a significant knowledge gap in understanding of natural tissue behavior.

3D printing, its ever-growing library of materials, and the diverse number of applications makes it an ideal method for generating bioreactors. 3D printed designs can be produced with many features integrated into larger features (monobloc), including fasteners, perfusion channels, and chambers, thereby reducing the number of parts required, limiting waste, and reducing the number of assembly steps. Biomed Clear (Catalog number: RS-F2-BMCL-01, Formlabs, Boston, MA, USA), an autoclavable and biocompatible medical grade 3D printing resin, is an excellent candidate as a bioreactor material. Not only is this autoclavable USP Class VI biomaterial manufactured in ISO 13485 facilities and supported with an FDA Master File, it is also readily available and can be printed on a small footprint Form 3 (Formlabs), Desktop 3D printer. Replacement parts made from BioMed Clear can be reprinted on demand in a few hours or overnight.

The Teburu [11], FABRICA [12], Schmid [13], and other 3D printed tissue perfusion bioreactor designs are 3D printed from sterilizable biomaterial resins and allow for perfusion [14]. Some even possess sensors which track tissue status during culture. Despite their research utility, each bioreactor platform still has drawbacks that undermine their potential for widespread adoption, with the most prevalent being;

1. Limited or no reusability.
2. Complicated assembly after sterilization.
3. Reliance on multiple fasteners or other small components that are difficult to track.
4. Reliance on autoclave-incompatible parts.
5. Require significant machining after printing.
6. Third-party or closed-source control systems.
7. Engineering or machining know-how required is beyond many user’s skills.

These drawbacks warrant in-depth reconsideration of how the advantages of 3D printing can be leveraged at every level (part, component, module, system, and complete platform) and every stage of production (fabrication, assembly, calibration, and operation) to simplify 3D tissue research. The limitations of current 3D tissue culture approaches in general calls for a thoughtful clean-sheet approach to culture 3D tissue platform design. The outcome of this clean-sheet approach should allow scientists deep analysis of 3D tissue models without being mired in the details of engineering such a system.

To account for the limitations of current bioreactor offerings, we have developed the bIUreactor 3D Tissue Research Platform. Our platform (hardware, software, and method) leverages the design freedom of 3D printing and the versatility of open-source concepts to generate a low-cost research solution capable of forming, perfusing, and cyclically compressing 3D tissues. With a modular design, additional modules such as sensors and environmental control systems can be developed and installed at a later date, futureproofing the platform. The user can produce the bIUreactor in their own lab because of the wide availability of the electronic components used, the maturity of desktop 3D printing, and relative affordability of both,. We therefore expect the bIUreactor will lower the barrier to entry for biologists interested in 3D tissue research, which will increase the number of 3D tissue researchers and lead to new biomedical breakthroughs.

Additionally, the system can easily be positioned in high resolution small field-of-view imaging systems (Positron Emission Tomography [PET], X-ray Computed Tomography [CT], etc.) enabling measurement of regional tissue characteristics, function, and metabolism without physically perturbing the tissue sample. Imaging enables comparison of tissues at baseline and in response to physiologic stimuli (perfusion changes, reagents, cyclic tissue compression, etc.) Since tissues in vivo are thick and cell dense, the ability to observe functional and metabolic activity within the bulk of a similarly structured 3D tissue will yield research outcomes more representative of a natural tissue than 2D studies or those using cells seeded within a reconstituted scaffold.

### Spheroid-Based Biofabrication

Spheroid properties make them ideal building blocks for 3D tissue biofabrication [15]. Spheroids are formed from aggregates of cells, resulting in microtissues comprised only of cells embedded within their own self-secreted ECM. They can be formed using several techniques ranging from simple hanging-drop to custom spheroid forms, making them accessible to most researchers [15, 16]. High cell-cell and cell-ECM interaction in spheroids is on par with natural tissues, which is important for tissue formation/maturation and cell function, particularly in 3D architecture [17]. They exhibit predictable and well-characterized nutrient transfer due to their symmetrical shapes and predictable packing in confined volumes. When properly sized with a radius below the oxygen diffusion limit (~ 250 microns) [18–20], cells on the spheroid surface and inner core can receive nutrients they need to support metabolism.

To form larger tissues, spheroids can be seeded atop one another in a pile, forming a self-supporting structure. The spheroids will begin fusing to one another [5, 12, 21, 22] although gaps form between them, forming a “leaky” tissue [23]. While this may seem to be a disadvantage, these naturally formed gaps serve as a 3D network of perfusible channels for nutrient-rich media to pass through during seeding and throughout culture. Utilizing continuous perfusion to keep the channels open allows “self-supported perfusion during formation and maturation” (SSuPerForM).

## Materials and Methods (Further Details, Including a User Manual, are Provided in Supplementary Documents)

### Computer-Aided Design (CAD) Modeling

**Fig. 1:**
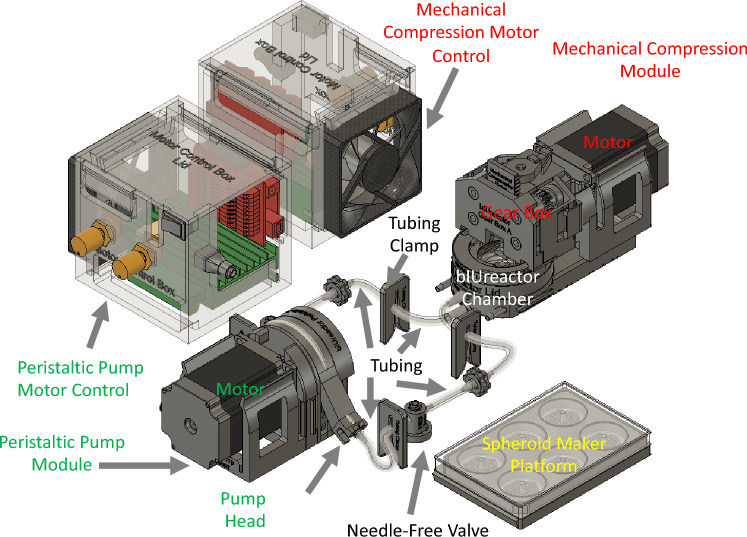
Schematic of the entire assembled bIUreactor Research Platform capable of providing simultaneous perfusion and cyclic mechanical compression. The Spheroid Maker Platform is for making spheroid microtissues used to comprise SSuPerForM Tissues. The bIUreactor Chamber, which contains the Tissue, can be integrated with a Compressor Module comprised a Motor mounted to a Gearbox. The Compressor Module provides cyclic compression to the Tissue contained within the bIUreactor Chamber. The bIUreactor with the Compressor Module mounted to it can fit within a standard cell culture incubator and within a 120 mm diameter Positron Emission Tomography (PET) bore. The bIUreactor Chamber, Lids, and Tubing, comprising the Flow Circuit are autoclavable for sterilization purposes. The Peristaltic Pump Module comprises a Motor which drives a Pump Head. The Tubing of the Flow Circuit is secured in the Pump Head. The Pump can support up to 4 pump heads, providing continuous perfusive flow to the tissues. Note that the Grommet and Gasket Molds and the Tissue Podium are not included in this figure

bIUreactor platform devices, including modules, and the molds for making silicone components were modeled using Autodesk Fusion 360 (Autodesk, San Rafael, CA, USA) on a MacBook Pro (Apple Computers, Cupertino, CA, USA) (Fig. 1).The device parts were designed following the aforementioned design requirements (Supplement 1). bIUreactor components were nested to minimize the space they consume in the autoclave and incubator. Once a satisfactory design was achieved, the components were converted to stereolithography (.stl) files and transferred to the Preform (Formlabs) 3D printing software. Note: Although the parts are printed or casted in translucent material, they are rendered in gray with colored hatching to provide contrast and context. The list of parts and their materials is provided in Supplement 5.

### Rapid Prototyping, 3D Printing, and Material Selections

To test for fit and basic function, design prototypes were printed in Draft Resin (Formlabs) on either a Form 2 or a Form 3 desktop 3D printer from Formlabs. Final designs and molds needing to withstand high temperature vulcanization (HTV) were printed in BioMed Clear on the Form 3B. The part design files were then transferred from the MacBook to the printer and the parts were printed.

### Post Processing

Completed parts were washed in 95% isopropanol for 20 min in a Form Wash (Formlabs), followed by another 5-min wash in a fresh 95% isopropanol bath and then dried using forced air. Parts were then post-cured in a Form Cure and supports were removed with flush cutters.

### Casting Silicone Parts

Molds for silicone parts were 3D printed using Biomed Clear. See Chapters 2 and 5–7 in the User Manual (Supplement 4) for more information. Silicone parts were made from Elastosil 610 (Wacker) but can also be made from Sylgard 184 (Dow, Michigan, USA). Briefly, molds were lightly coated with mold release. The silicone was mixed into a syringe and centrifuged to remove air bubbles. Silicone was dispensed into the molds and vulcanized at 200 °C for 20 min. In some cases, the filled molds were centrifuged at 500×*g* to remove bubbles formed during silicone dispensing. The molded parts were cooled after vulcanization and removed by hand.

### Motor Control

Components used to provide controlled rotational torque to the Gear Box and the Peristaltic Pump are listed and described in Supplement 5. Circuit design and Arduino code (also called a sketch) for the Mechanical Actuator and the Peristaltic Pump are provided in Supplements 3 and 4. Arduino is supported on Linux, Mac OS (Version 10.14: “Mojave” or newer), and Windows (Win 10 and newer) systems with 64 bit architecture. The systems are also required to have 256 MB RAM, 600 MB of disk space, and a network connection[24]”. Once installed the Arduino code was uploaded. Simple push-in connectors and pin connectors were used to connect wiring instead of complicated and potentially hazardous soldering.

### Cyclic Compressor Module Testing

The ability of the Compressor Module to provide consistent compression over the course of 5-min sessions while simultaneously providing perfusion over a simulated 5-day period was tested. A calibration (Supplement 4) was performed to set the piston displacement level and loading frequency applied to the SSuPerForM tissue. The Compression Module was set to cycle the Piston at a displacement of 1.25 mm and a loading rate of 18 cycles per minute (cpm) for 5 min. The bIUreactor was loaded with a Platen and a simulated SSuPerForM Tissue comprised agarose gel beads. This was coupled to the Compressor Module, and placed inside of a cell culture incubator set to 37 °C. The system was allowed to equilibrate overnight and then 5-min compression tests, representing 1 test per day over 5 days, were performed over the course of an hour. The compression distance of the piston was checked after each 5-min compression cycle. After testing, the Compressor module gears and bearing surfaces were checked for wear particles and friction marks.

### Pump Testing

The ability of the pump to perfuse tissues within 4 bIUreactors for up to 5 days was tested. Four sets of bIUreactor Chambers were attached to a Peristaltic Pump setup with 4 Pump Heads. The system was calibrated (Supplement 4) and operated with a pump flow rate of 10 ml/min. Hourly, photographic and thermal images of the Pump Heads, the Stepper Motor, and Control Box were collected using a FLIR thermal camera (FLIR, Model FLIR-E6390, Wilsonville, OR) to track the system temperature. Motor temperature was tracked to ensure it was at a level safe for handling without heat PPE and would not damage underlying table tops. 140 °F is the generally accepted upper temperature limit prescribed for hot surfaces that humans may come into skin contact with, per California Code of Regulations, Title 8, Section 3308, Hot Pipes and Surfaces.

After 5 days of pump operation, the flow rate was measured and compared to the initial 10 ml/min flow rate. A qualitative analysis was made to determine the degree of wear on the tubing and other friction surfaces.

### Flow Circuit Preparation

Prior to autoclaving, Tygon 3350 silicone tubing (1.52 mm inner diameter, Cole-Parmer Ismatec, Wertheim, Germany) was attached to the inlet and outlet of the bIUreactor chamber. The lids and silicone parts were placed onto the Chamber. The Mesh and Platen were placed into the Chamber. Needle-Free Valves were placed in the flow circuit near the Inlet and Outlet to facilitate medium transfer into and out of the flow circuit. This completed the flow circuit. The bIUreactor components were then pumped with 70–80% ethanol bath, washed with water, and air dried.

### Autoclaving

The spheroid makers and assembled Flow Circuit were autoclaved with a 20-min sterilization phase at 121 °C and a 5-min dry phase.

### Spheroid Production, SSuPerForM Tissue Production, and Culture in the bIUreactor

**Fig. 2:**
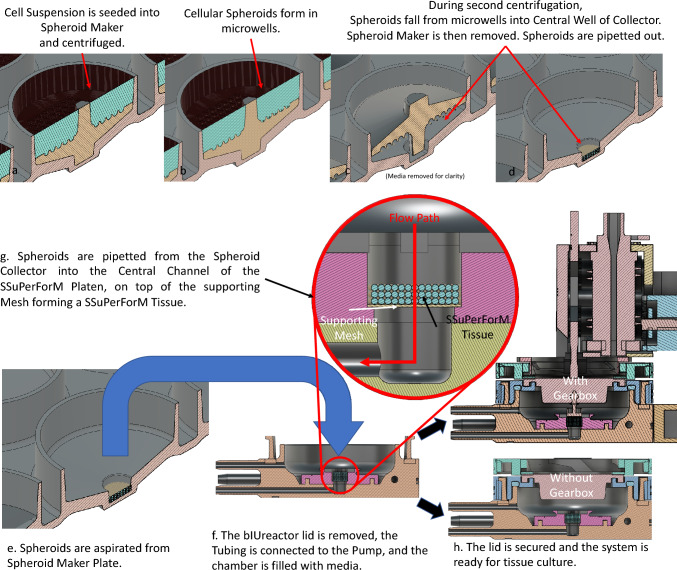
Schematic description of the Spheroid making process using the Spheroid Maker and Spheroid Collector and the SSuPerForM Tissue Formation process. **a** Spheroid Maker-Collector assemblies are positioned in the well of a 6-well plate with the Maker on top of the Collector with the microwells concave-sides up. The reservoir is filled with a cell suspension. **b** The suspension is centrifuged, forcing cells into the microwells. **c** and **d** After overnight incubation and the spheroids have formed, the Spheroid Maker is inverted at 200×*g* for 2 min to force the spheroids into the central well of the Spheroid Collector. If all the spheroids did not immediately fall into the Central Well, they were coaxed into the central well by gently orbiting the Collector or plate full of Collectors. The Spheroids were then aspirated from the Well of each Collector using a 1000 µl pipettor, often in one draw. **e** and **f** Spheroids are transferred by pipette into an open bIUreactor filled with media. **g** Spheroids are pipetted from the Spheroid Maker Plate into the Central Channel of the SSuPerForM Platen, forming a SSuPerForM Tissue. 4. The lid is secured and the bIUreactor is ready for perfusive tissue culture

SSuPerForM Tissue constructs were made using cellular spheroids made from late osteoblast murine IDG-SW3 cells [25], meaning they are comprised only of cells and the extracellular matrix they secrete and they can undergo perfusion immediately upon formation and throughout maturation. These features make them ideal for forming larger tissues and nutrition-regulated maturation. Briefly, (Fig. 2A) sterile Spheroid Makers were placed into custom 3D printed (BioMed Clear) 6-well plates with the dimple-like microwells facing up. Next, 4.8 million IDG-SW3 cells (passage number = 23–25) were suspended in 2 ml of culture medium (α-modified Eagle's medium [α-MEM] plus 10% fetal bovine serum [FBS] and 1% penicillin/streptomycin) and pipetted into the Spheroid Maker. Each plate was covered with a lid, centrifuged at 500×*g* for 5 min to force the cells into the microwells, and incubated (37 °C, 5% CO_2_, and humidified air)_. IDG-SW3 spheroids formed overnight during incubation. To harvest spheroids, the central grip on each Spheroid Maker was gripped with sterile forceps to invert the Spheroid Makers into a microwell-side down orientation inside the 6-well plate. The covered plate was centrifuged again to force the spheroids into the center of each well of the 6-well plate. After centrifugation, the plate of spheroids was set aside for bIUreactor Chamber preparation

Cell culture media was dispensed through the tubing into the bIUreactor Chamber at a rate of 10 ml/min using a Pump. This filled the chamber with media and primed the tubing. With the Pump running, the spheroids were pipetted from each well with a simple draw of a 1000 µl micropipettor and then transferred over top of the Mesh secured in to the Central Channel of the Platen. The flow pulled the pile of Spheroids to the Mesh, thus forming the SSuPerForM Tissue (Fig. 2B). A total of 480 to 720 spheroids (4–6 Spheroid Makers worth) were used for each SSuPerForM Tissue. The Lids were secured onto the tissue culture chamber and the bIUreactor was then placed carefully into the incubator. The tubing was threaded through the back of the incubator and connected to the Pump. The Pump was driven at 10 ml/min for 5 days. On the 3rd or 4th day of culture the Peristaltic Pump was stopped, fresh media was exchanged through the Needle-Free valves, and the Pump was restarted.

### PET Imaging and Imaging Analysis

After 5 days of culture the bIUreactor assembly was prepared for tissue metabolism kinetic analysis using the IndyPET III Positron Emission Tomography (PET) scanner and [^18^F] FDG radiotracer. The tissue culture chamber was mounted to the IndyPET III scanner gantry so the culture chamber could be placed in the center of the PET scanner bore. The Pump was started the PET scan was initiated, and the radiotracer was injected into the flow circuit. After 40 min, the Pump was stopped and two fresh media flushes were performed, leaving only radiotracer taken up and retained by the tissue. PET images were reconstructed using a Filtered Backprojection Algorithm into a temporal sequence of images to enable visualization of the distribution of Flourine−18 during the recirculation, washout, and tissue retention phases of the study. SSuPerForM Tissue uptake and retention of the radiotracer are indicators of metabolically active tissues. See also, Supplement 4, Media Exchange.

### Tissue Removal

At the end of the study, the Platen containing the SSuPerForM tissue was removed from the bIUreactor and the center of the Platen was placed over the center post of the Tissue Podium. By pushing down gently on the Platen, the tissue was pushed up and out of the Platen. The tissue was then gently removed from the supporting Mesh using a pair of forceps.

### Doppler Ultrasound Testing of Flow Through SSuPerForM Tissues Within bIUreactor Chamber

To assess the potential of measuring flow characteristics through the SSuPerForM Tissues with and without the End-Effector, Color Doppler cineloops and Pulse-Wave Spectral Doppler images were acquired using a commercial ultrasound imaging system (Aixplorer, SuperSonic Imagine, Aix-en-Provence, France) with a high-frequency SL22-7Lab linear array transducer (nominal bandwidth from 7 to 22 MHz). The bIUreactor flow circuit was assembled as described in the tissue culture section, with the exception that the lids were kept off the chamber, water was used instead of culture medium, and 500 µm diameter agarose beads (Cyfuse, K.K., Tokyo, Japan) were used instead of spheroids. Cornstarch was mixed into the water to serve as a reflecting medium for producing a measurable ultrasound signal. The transducer was partially submerged into the water solution over the agarose beads and the Doppler images were obtained with the primary flow direction parallel to the insonifying beam direction. Flow characteristics through the SSuPerForM Tissues were measured at 0, 1, 5, and 10 ml/min flow rates.

## Results

### bIUreactor Design Overview

**Fig. 3:**
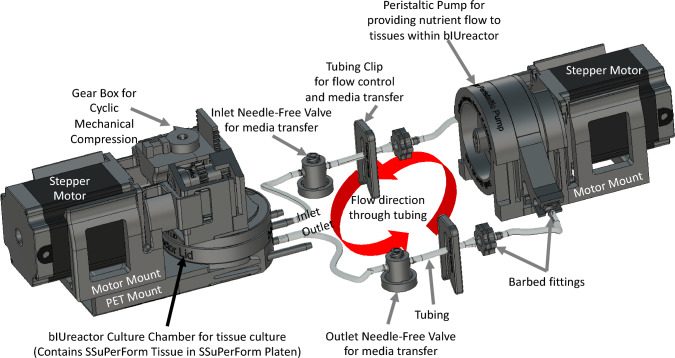
Overview of the bIUreactor Chamber and Peristaltic Pump. Tissues made from Spheroids formed in the Spheroid Maker are contained within the bIUreactor Culture Chamber. The Tissues are perfused by the Peristaltic Pump while simultaneously being cyclically compressed by the Compressor. The Tubing Clips, Barbed Fittings, and Needle-Free Valves are used to control flow direction during media transfer and pump manipulation

**Fig. 4:**
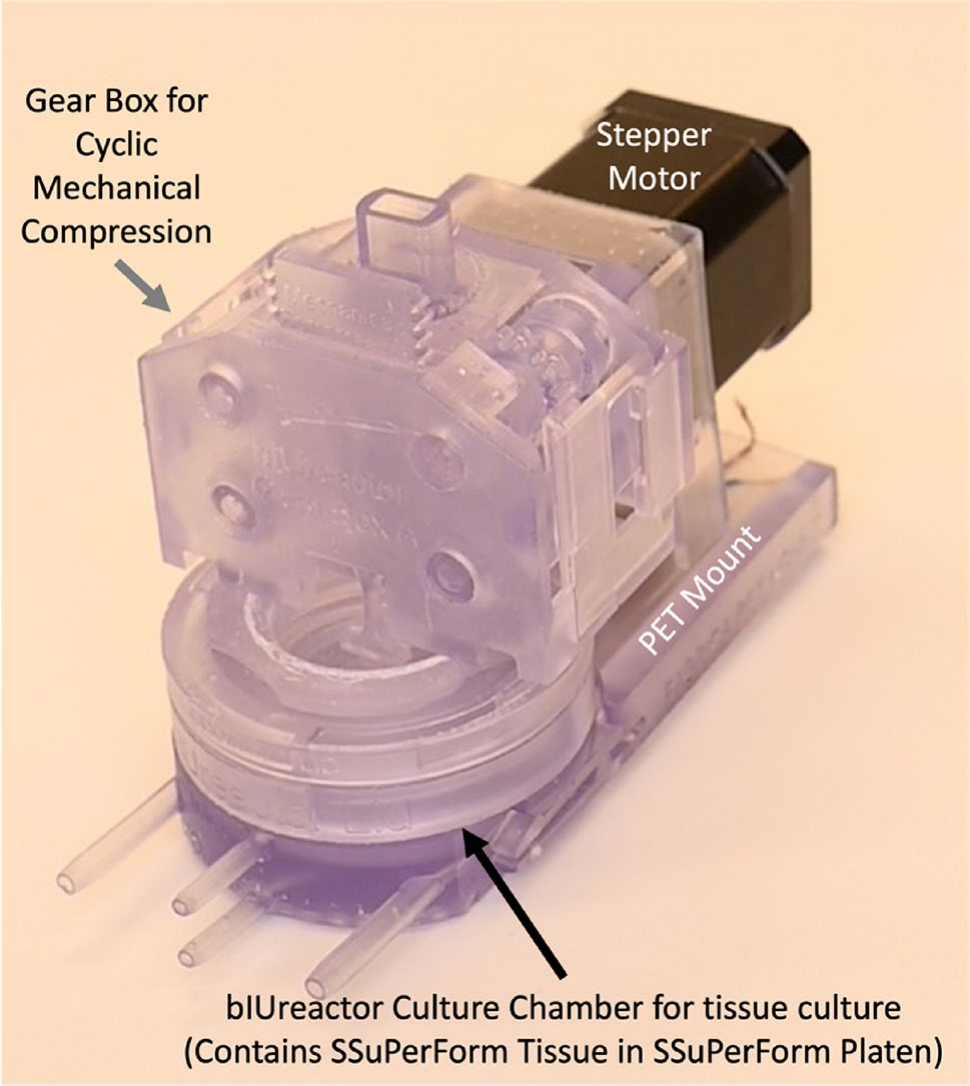
Photo of bIUreactor Chamber with Compressor Module. A Stepper Motor attached to the Gearbox and drives a Mechanical Compression Piston which presses the top of a special silicone Grommet. The Grommet transfers force and motion to the End-Effector, which compresses the tissue

**Fig. 5:**
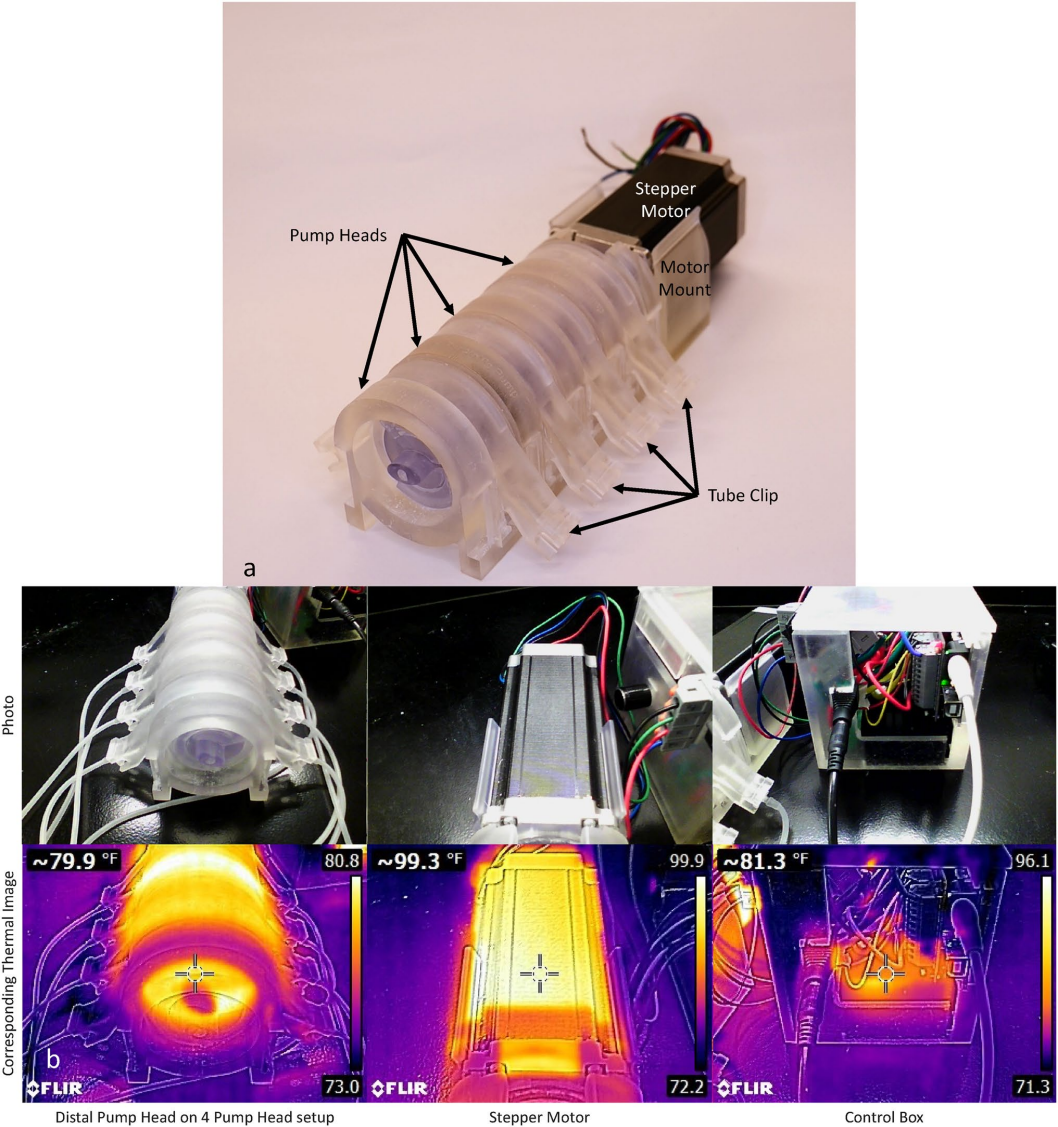
Photo of Peristaltic Pump with 4 pump heads and Tube Clips. The motor is connected to the Pump Heads by means of a Motor Mount. 5B. Photographic and thermal images of the Peristaltic Pump set up

All components (Supplements 4 and 5) were successfully designed in CAD (Figs. 1 and 3) and printed/casted or purchased (Figs. 4 and 5).

### Cyclic Compressor Module Testing

Simultaneous Cyclic Compression and perfusion of the simulated SSuPerForM Tissue was achieved. The Piston displacement after 5 trials was 1.32 ± 0.007 mm. The initial and final loading rates were 17.5 cpm. There was minimal wear or particulate debris observed as a result of gearbox operation.

### Pump Results

Component temperature, flow rate, and RPM of a 4-Pump Head Peristaltic Pump setup were measured over the course of 5 days (Fig. 5). The Stepper Motor in the 4-Pump Head set up reached a maximum of 137° F and then reached a steady state temperature of 130.8 ± 4.0° F. the Occupational Safety and Health Administration limits the safe operating temperature of contact surfaces to a maximum of 140° F. The pump head reached a steady state temperature of 79.08 ± 2.8° F. The motor controller initially reached a steady state temperature of 160 °C until the cooling fan was connected after the first 20 h. The motor controller temperature then dropped to a steady state of 90.8 ± 3.0° F. The Stepper Motor rotations per minute remained consistent at 50 ± 0.47 rpm over the duration of the study. The 10 ml/min flow rate set in each bIUreactor at the start of the experiment was also observed at the end of 5 days. There were some wear particles on the Rack surfaces, although this was kept to a minimum and is expected to reduce drastically as the Pump is “worn in”. Rollers and Key Components showed little to no wear. Further wear analysis and a potential material change to wear bearing components will require further study. The Tubing was flat but remained patent and showed a continued ability to pump after 5 days.

### SSuPerForM Tissues in the Platen can be Perfused with or Without the End Effector

Doppler ultrasound was used to confirm perfusion of water (a stand in for cell culture medium) through to cells in the SSuPerForM Tissue bulk and that is a function of volumetric flow rate. This is critical since flow regulated nutrient perfusion to cells within the tissue bulk is paramount to controllable tissue culture and predictable tissue culture outcomes.

**Fig. 6:**
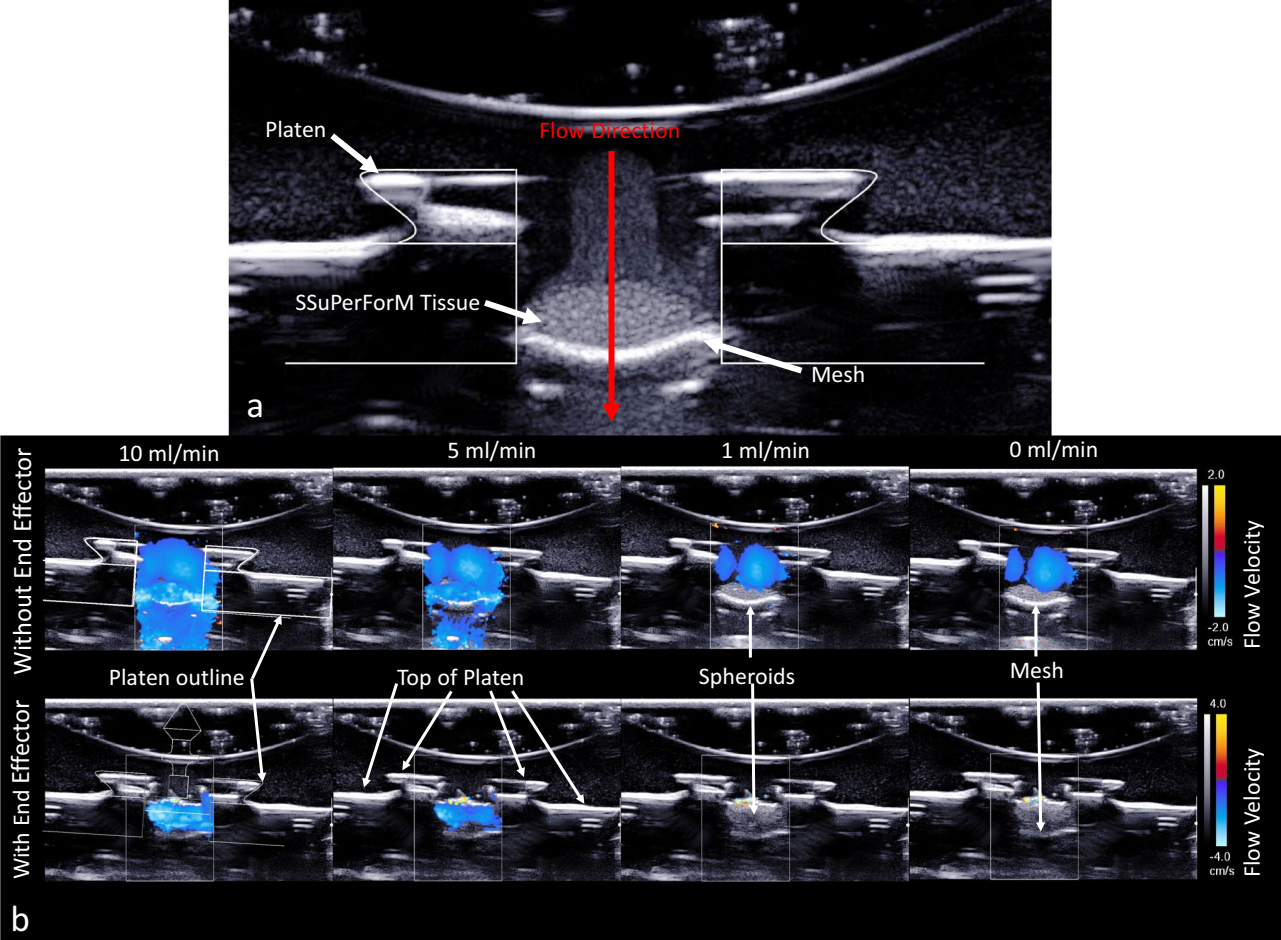
Doppler Ultrasound showing flow through agarose bead SSuPerForM Tissues formed in Platens. **A** Doppler graph of the tissue within the Platen before flow is applied. The spheroids forming the SSuPerForM Tissue are visible, as is the central channel containing the Tissue. The thin Mesh visible under the tissue provides support while permitting perfusion. **B** The lower row shows flow can also pass through the End Effector and perfuse the SSuPerForm Tissue. Note the different velocity scales next to each row of flow profiles

Outlines of the Platen or End Effector and Platen cross-sections are overlaid on top of Color Doppler images of flow through the tissues loaded in the platen with and without the platen (Fig. 6). Without the platen, perfusion through the SSuPerTissue was observed at 10, 5, 1, and 0 ml/min, although the flow profiles at 1 and 0 ml/min looked similar. This suggests that some of the flow observed at lower flow rate is an artifact of the cornstarch medium settling. With the End Effector, flow was observed only at 10 and 5 ml/min, indicating a reduction in flow due to End Effector interference. Note the flow velocity scales are different for the configurations with or without the End-Effector.

### Spheroid Production and SSuPerForM Tissue Culture in the bIUreactor Chamber

**Fig. 7:**
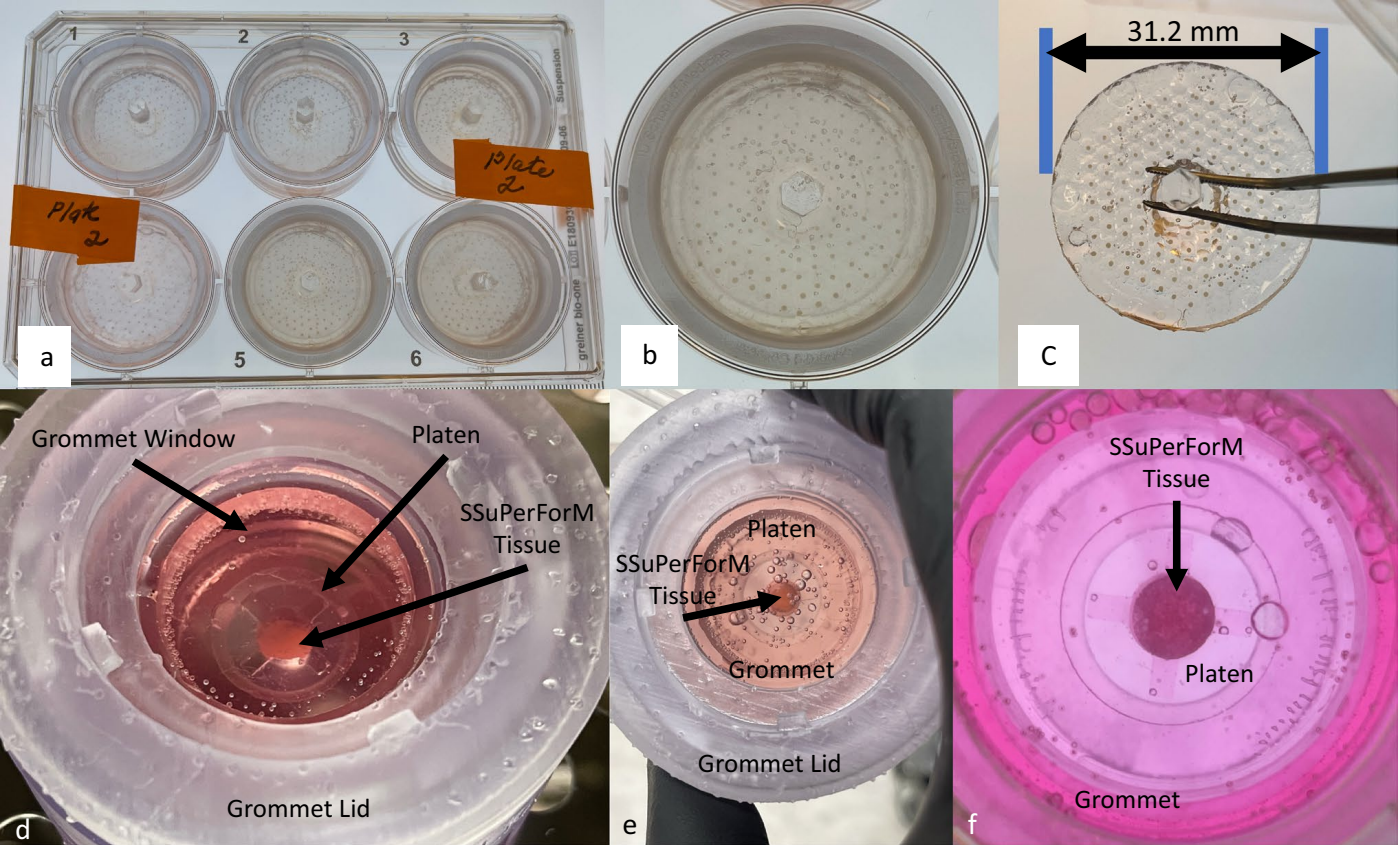
**A** A six-well plate containing six Spheroid Makers with spheroids in each microwell. The Spheroid Makers are nested within the Spheroid Maker Plate. **B** Closeup of freshly formed Spheroids in a Spheroid Maker. **C** Closeup of a Spheroid maker just prior to centrifugation for Spheroid removal. The Spheroid Maker can be manipulated aseptically by sterile forceps. **D** Photos of SSuPerForM Tissue in bIUreactor Chamber. **E** The SSuPerForM Tissue can be observed through the Grommet secured by the Grommet Lid. The platform is translucent to allow for different imaging approaches. **F** Closeup of the SSuPerForM Tissue immediately after formation. The tissue is nested within the Platen, which is nested within the bIUreactor

Spheroids were easily observed in the Spheroid Makers, when held over a light background or backlit (Fig. 7A–C). The average diameter was 502.22 µm with a standard deviation of 49.13 µm and *p* = 0.003. The spheroids exhibited a maximum diameter of 622.71 µm and a minimum diameter of 409.69 µm. The SSuPerForM Tissue diameters matched the inner diameter of the Platen, which is 7 mm. The tissue height (*n* = 3) was 1.23 ± 0.23 mm.

**Fig. 8:**
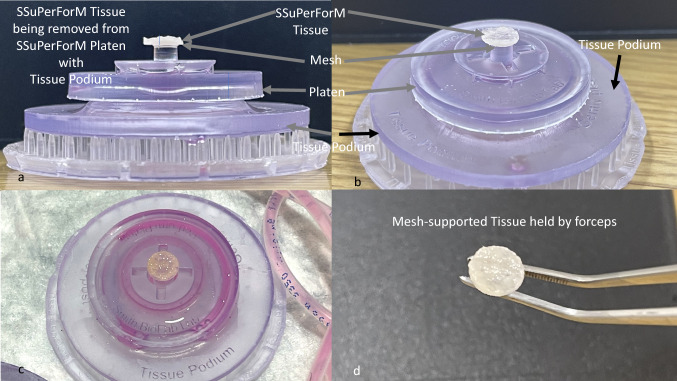
**A**–**C** The SSuPerForM Tissue and Mesh are suspended on top of the post where they can be removed easily with forceps. **B** and **C** Oblique views of tissue supported by tissue podium. Photo of Mesh-Supported SSuPerForM tissue held by forceps

The IDG-SW3 SSuPerForM Tissues were perfused for 5 days at 10 ml/min. The SSuPerForM nested in the Platen could be easily observed through the Grommet window of the fully assembled bIUreactor Chamber, especially when held over a light background or backlit (Fig. 7E and F). After 5 Days, the tissue could be removed using the Tissue Podium (Fig. 8A–D). The resulting tissues were compacted disks that could be handled with forceps with or without the backing of the Mesh (Fig. 8D).

### PET Imaging and SSuPerForM Tissue Viability in bIUreactor

**Fig. 9:**
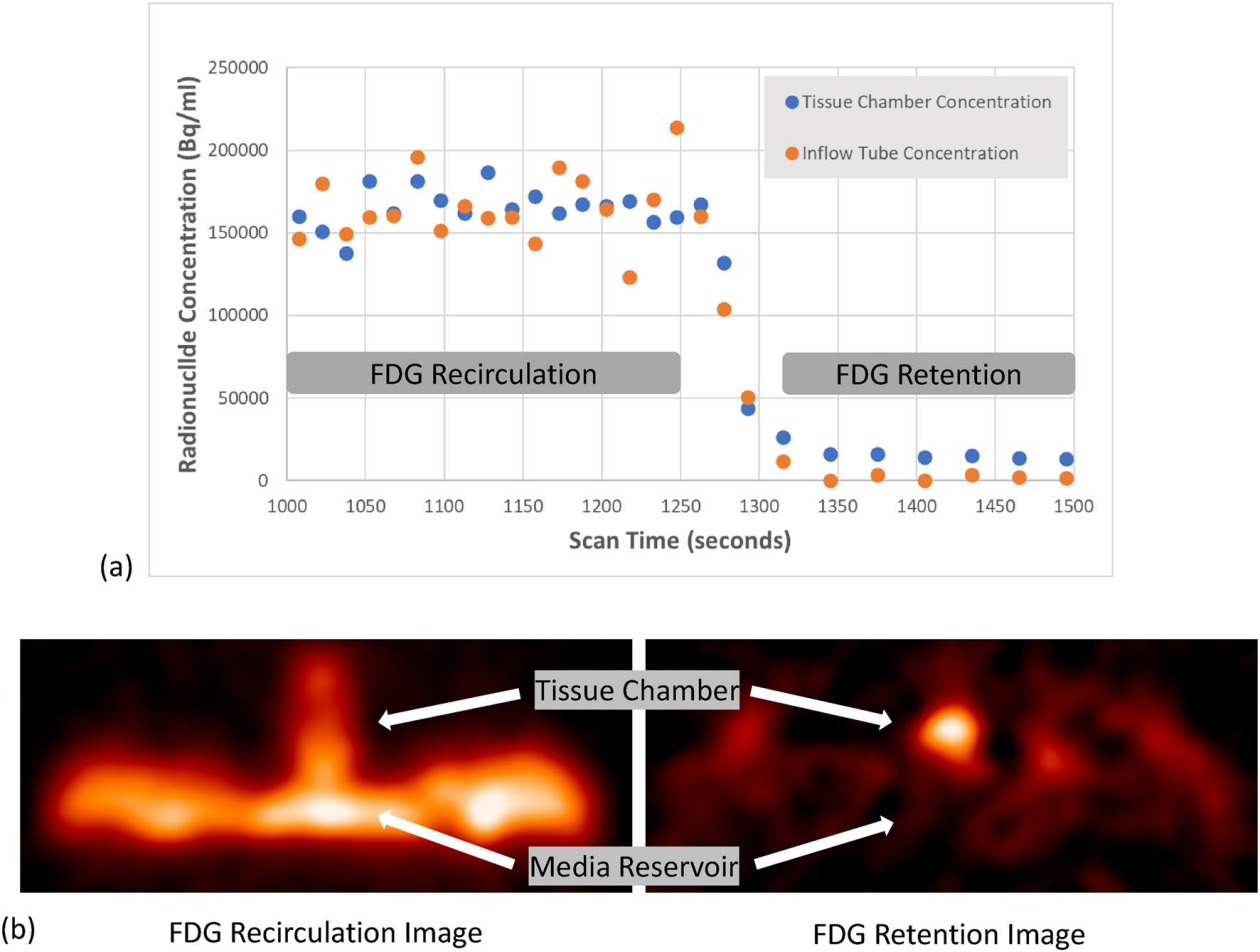
[^18^F]FDG kinetics within the bIUreactor. **A** The plot shows the Fluorine−18 concentration in the inlet tube to the bIUreactor (orange dots) and the tissue chamber (blue dots). During the recirculation phase the signal in the tissue chamber is dominated by the [^18^F] FDG in the culture media (left side of **A** and **B**). Following washout of the media containing [^18^F] FDG, retention associated with metabolism of [^18^F] FDG is clearly evident in **B** and the right side of the plot

After 5 days of culture in the bIUreactor, the IDG-SW3 SSuPerForM tissue was scanned in the IndyPET III. The chart in Fig. 9 demonstrates the temporal behavior of [^18^F] FET-FDG within the bIUreactor and SSuPerForM tissue during FDG recirculation and media flush portions of the study. Metabolic retention of FDG in the tissue chamber following washout of the FDG from the culture media is shown in the right-hand PET image in Fig. 9, demonstrating metabolic viability of the SSuPerForM tissue after 5 days of contiguous perfusion. Figure 9 below is of a single run of the PET analysis.

## Discussion

We have developed and demonstrated the use of a novel open-source, modular, 3D tissue biofabrication research platform by leveraging properties of 3D printing, open-source electronics, and spheroid culture. By following the Design Philosophy set forth in Supplement 1, we have produced the first modular reusable open-source bioreactor platform that provides for biofabrication of 3D structured scaffold-free tissues, simultaneous perfusion and compression, sample observation during culture, and simple harvesting/removal after culture. To our knowledge, there are no other platforms that provide all of these capabilities in an open source manner. Notably, the bIUreactor mitigates shortcomings of other bioreactor platforms by facilitating easy production and operation by the user-scientist *in laboratorium*. Explicitly, the bIUreactor allows 3D tissue biomedical research without users needing to perform prohibitively costly or complex engineering. There are several practical features to the bIUreactor design which enhance its utility. Examples include:

- Open-source.
- Integrated fasteners, which reduce the need to track separate fasteners (screws, nuts or bolts).
- Assembly of the entire Flow Circuit, including Chamber, Lids, Fittings, and Tubing, prior to sterilization allows autoclaving after assembly, minimizing contamination risk.
- No soldering required.
- Immediate tissue perfusion.
- Physical and visual tissue access while mitigating contamination risk.
- 3D printed Needle-Free Valves for aseptic port access for medium exchange.

### Tissue Fabrication

The SSuPerForM Tissue dimensions are determined by the size of the opening in the platen and the limits of tissue perfusion, which should be determined for each tissue type. In the current design, the tissue Platen opening was 7 mm in diameter and up to 4 mm high. Other tissue designs can be prescribed by adjusting the design of the Platen opening and the Mesh.

### bIUReactor Chamber and Actuator Function

#### PET Imaging

Functional imaging with positron emission tomography (PET) measures and visualizes changes in metabolic and other biological processes. We decided to forgo histological tissue assessment and instead used PET imaging of SSuPerForM glucose uptake and retention as a direct indicator of continuous tissue metabolism in the bIUreactor. Contiguous tissue viability over 5 days shows that the platform is suitable for culturing cell-dense tissues. For the first time we have demonstrated PET being used to image metabolite uptake and retention by a 3D structured tissue comprised entirely of cells and their self-secreted ECM [26–28]. Although the common user may not rely on PET for their research, PET compatibility makes the bIUreactor a powerful platform for drug discovery, radiopharmaceutical validation, genetic testing, and other situations where changes in tissue metabolism and/or biochemistry are useful indicators of tissue response to an input.

## Longer Term Studies

Analysis of longer term performance must and will take much longer (e.g. 5–40 days per study) and greater expertise than this introduction of a comprehensive biofabrication platform entails. There are new materials being released that may be better suited to long term performance. We will also need to include an engineer/author specializing in polymer wear. There is also the opportunity for the community to contribute to the further development of this platform. This is the benefit of making the system open source. Users within the research community are able to build, modify, and scrutinize the system to their satisfaction.

### Flow Verification Using Computational Fluid Dynamics (CFD)

The flow conditions at low flow rates associated with the End-Effector could not be resolved using Doppler Ultrasound. Future studies will rely on CFD analysis of perfused SSuPerForM Tissues to determine the flow state at high resolution in lumenal low flow rate conditions. At higher flow rates, however, we were able to use Doppler ultrasound to demonstrate that the tissues were being actively perfused throughout their bulk with or without the intervening compressing End Effector. This information about the flow field will allow tissue engineers to tune the culture conditions to provide desired perfusion and nutrition to their tissue cultures. Future modules will include Doppler Ultrasound modules that will permit real time flow imaging of the tissue constructs during culture.

### Simplified Setup and Operation

Note the mitigation of soldering for securing wire circuits. Normally, wiring is secured using a low-melting-temperature metal (solder) which is heated to its melting temperature at the junction of a wire and contacts on the circuit board. This cools into a solid junction, making a conductive joint. Soldering is painstaking and requires significant skill, which may be beyond the purview of the bIUreactor User. The Shield Kit makes conductive joints between the Arduino and the Stepper Motor Controller using simple push-in spring connectors without soldering. Similarly, Lever Wire Nut Connectors connect wires to ensure a stable, protected wire circuit. Future research must involve ensuring the design can accommodate many users with different hand sizes and manipulation capabilities. This may involve implementation of grip modules for different gripping styles and situations.

### Operational (Microenvironment) Envelope

In vivo, cells are sensitive to their mechanical, electrical, and chemical microenvironment, which regulate all cell activity, including metabolism, differentiation, ECM deposition, and proliferation. Well-designed bioreactors are capable of applying these microenvironmental factors in a controllable manner.

The operational envelope of the microenvironment produced by the bIUreactor has not been determined, requiring further testing and validation for specific cell and tissue types. However, the ability of the bIUreactor to provide perfusion and cyclic compression makes it ideal for: relatively stiff tissues that are subjected to both such as bone; for tissues that require significant perfusion such as skin, brain, and muscular tissues; and for tissues requiring perfusion-free compression such as cartilage. Further characterization would be required for much softer tissues such as blood or bone marrow. Softer tissues that flow, like blood, would be susceptible to damage while passing through the peristaltic system while it would be difficult to produce and maintain the structure of marrow tissues. Establishment of a quantifiable operational envelope will require further investigation and the development of several sensing modules.

### Maker Spaces and 3D Printer Access

Desktop 3D printing has matured into a robust technology/industry with new material applications ranging dental prosthetics, end-use ceramic parts, mechanical devices, and medical applications. This is coupled with a relatively low 3D printer cost and compact size, allowing a research lab to house several in their facility and print their own lab devices. The proliferation of maker spaces and off-site 3D printing services also allows labs to outsource 3D printing for their research needs if housing a 3D printer in-lab is not feasible. Compared to the costs and complexities of engineering/fabricating devices using conventional “machining” methods (sawing, milling, grinding, sanding, etc.), the 3D printed bIUreactor poses a distinct advantage.

### Modularity and Futureproofing

The bIUreactor is relatively basic, providing only oxygenated perfusion and cyclic mechanical compression at present. However, due to the thoughtfully considered precepts set forth by the Design Philosophy and the modularity leveraged by the bIUreactor hardware, our platform is futureproofed for new concepts. This is an important feature since pressure sensing, pH sensing, oxygen control, temperature control, and other modules need to be developed for the bIUreactor to be a truly comprehensive research platform. Moreover, MRI-compatible modules, which must be free of ferromagnetic materials (such as those found in the stepper motors currently implemented in the bIUreactor) are also a goal.

To make the bIUreactor widely available, we have set up a GitHub (https://github.iu.edu/smitlej), which will be used to distribute instructions for the platform, provide downloadable 3D print files and software, and provide a development community. Through our GitHub, our plans are to develop and characterize more modules and refine the platform. The authors assume no legal liability stemming from fabrication, setup, or use of the bIUreactor Platform.

### Open-Source as a Research Tool: OpenFlexure as an Example

Open-source concepts present an excellent medium for distribution of scientific instrumentation, research know-how, and education as it allows members of a community to study, build, modify, update, and improve projects without expensive paywall barriers or roadblocks associated with proprietary models. Arduino and Raspberry Pi are capable open-source electronics platforms based on easy-to-use hardware and software. With their electronics boards, simple software, sensors, switches, and motors, open-source electronics can be used to automate and control research platforms such as bioreactors discussed herein or optics platforms such as the OpenFlexure microscope platform successfully deployed by OpenFlexure.org and Bath University. Open Flexure, open-source itself, has a simple “Build, Install, Use” approach with easy-to-follow instructions provided both in text and visual form. OpenFlexure Microscope parts are 3D-printed from .stl files provided online. The Raspberry Pi-based software used to operate the microscope is installed from GitHub, an open-source internet host used for software development and version control. Raspberry Pi and Arduino have large support communities, meaning their open-source status allows users to develop their own code and customize their own electronics boards. With their online forum, users of the OpenFlexure platform can seek community guidance on each stage of the microscope Build, Install, Use process. OpenFlexure therefore demonstrates how 3D printed, low-cost, and open-source tools can empower scientists. In a new field like biofabrication and 3D tissue research, the open-source bIUreactor Research Platform will serve to expand research possibilities available to biology labs studying realistic tissue models.

### Supplementary Information

Below is the link to the electronic supplementary material.

Supplementary file1 (PDF 45 kb)

Supplementary file2 (PDF 67352 kb)

Supplementary file3 (PDF 231 kb)

Supplementary file4 (PDF 46876 kb)

Supplementary file5 (PDF 74 kb)
