# Supplementary material for: The ‘bIUreactor’: An Open-Source 3D Tissue Research Platform
Source: Ann Biomed Eng. 2024 Mar 26;52(6):1678–92. doi: 10.1007/s10439-024-03481-5 (PMC11082015; doi:10.1007/s10439-024-03481-5)
Supplement: Supplementary file 1 — Supplementary file1 (PDF 45 kb) [file 10439_2024_3481_MOESM1_ESM.pdf]

Supplement 1
Design Requirements

The bIUreactor shall feature biocompatible components which are:

- 3D-printed for on-site fabrication using low-cost desktop 3D printers or casted onsite from 3D-printed parts
- Designed for easy 3D printing fabrication, component repair, and part replacement in 24 hours or less
- Designed to require minimal fabrication, electronics, or coding expertise
- Designed for fabrication and assembly by a user with a biology (non-engineering) background
- Embossed with each component name to simplify assembly
- Assembled without the need for tools, screws, or non-integrated fasteners
- Made from autoclavable materials for easy sterilization and multiple uses
- Designed for medium exchange and tissue access (physical and visual) while mitigating contamination risk
- Designed to allow simple integration with real-time imaging modalities. For example,
    - Positron emission tomography (PET) scanners have small diameter central bores
    - Magnetic resonance imaging (MRI) scanners require non-ferrous materials, including drives

The bIUreactor tissue fabrication and culture method shall feature:

- Minimal or no mechatronic automation but relies primarily on manual, hand-based tissue biofabrication techniques when possible
- Microtissues comprised of cells embedded within their own cell-secreted matrix, such as spheroids or other scaffold-free tissues, when possible
- A technique and workflow which minimizes contamination risk
- A technique and tissue structure which allows immediate and persistent perfusion to reduce tissue necrosis and/or hypoxia
- Non-destructive tissue removal from the tissue culture chamber at experiment termination

The bIUreactor platform shall permit:

- Assembly of the entire culture platform, including chamber, fittings, and tubing, prior to sterilization
- The entire assembly to be autoclave sterilized at the same time, minimizing contamination risk
- Mounting of the tissue within the sterile perfusion chamber already filled with media and immediately perfused (even without the lid), mitigating necrosis and hypoxia
- Easy tissue access with as little disassembly as possible
- Easy tissue access without disassembling the flow circuit
- Aseptic visual tissue observation without disassembling the flow circuit or removing the lid
- Aseptic port accessibility of sensors and fluid circuits
- Externalization of components or devices that do not require incubation, allowing more bIUreactor experiments per incubator
- Continuous oxygenated perfusion culture for up to 35 days
- Perfusion and mechanical loading powered and controlled by open-source electronics
    - With non-metallic parts and pneumatics, when metal parts and electronics are not feasible, such as MRI
- Secure and safe circuitry fabrication and assembly without soldering
- Episodic cyclical mechanical stimulation of tissue up to 5 hours per episode

To future proof and ensure continued and widespread usability, the bIUreactor shall also feature:

- Open-source or low-cost access to part, component, module, system, and platform designs, when possible, for easy, community-based implementation
- Open-Source or low-cost electronics and code, including GitHub hosting, for update, troubleshooting, and version control provisioning
- Modular design to permit inclusion of new module designs and new capabilities
- Compatibility with both scaffold-free and scaffold-dependent tissues
- bIUreactor Research Platform drawings, parts list, mold information, and Arduino code
