# Supplementary material for: The ‘bIUreactor’: An Open-Source 3D Tissue Research Platform
Source: Ann Biomed Eng. 2024 Mar 26;52(6):1678–92. doi: 10.1007/s10439-024-03481-5 (PMC11082015; doi:10.1007/s10439-024-03481-5)
Supplement: Supplementary file 2 — Supplementary file2 (PDF 67352 kb) [file 10439_2024_3481_MOESM2_ESM.pdf]

Supplement 2
Detailed Methods

bIUreactor Platform drawings, parts list, mold information, and Arduino code

Computer-Aided Design (CAD) Modeling

bIUreactor platform devices, including end-use devices, modules, and the devices used to mold silicone components, were modeled using Autodesk Fusion 360 (Autodesk, San Rafael, CA, USA) on a MacBook Pro (Apple Computers, Cupertino, CA, USA). The device parts were designed following the aforementioned design requirements, also making sure bioreactor components are nested to minimize the space they consume in the incubator. The SLA 3D printing process relies on integrated supports to ensure part integrity during printing which must be removed after the printing process is complete. Therefore, we were careful to ensure the supports were: not in places where a flat interface was needed; not in difficult to access locations within the part; not risking inadvertent damage, not occluding flow. The names of the parts were debossed on their surface to help identify them during assembly. The parts were also modeled so that all parts for each individual device could be printed in one batch on a Form 3 (Formlabs, Somerville, MA, USA) desktop 3D printer. Once a satisfactory design was achieved, the components were converted to .stl files and transferred to the Preform (Formlabs) 3D printing software. Note: although the parts are printed or casted in translucent material, they are rendered in gray with colored hatching to provide contrast and context.

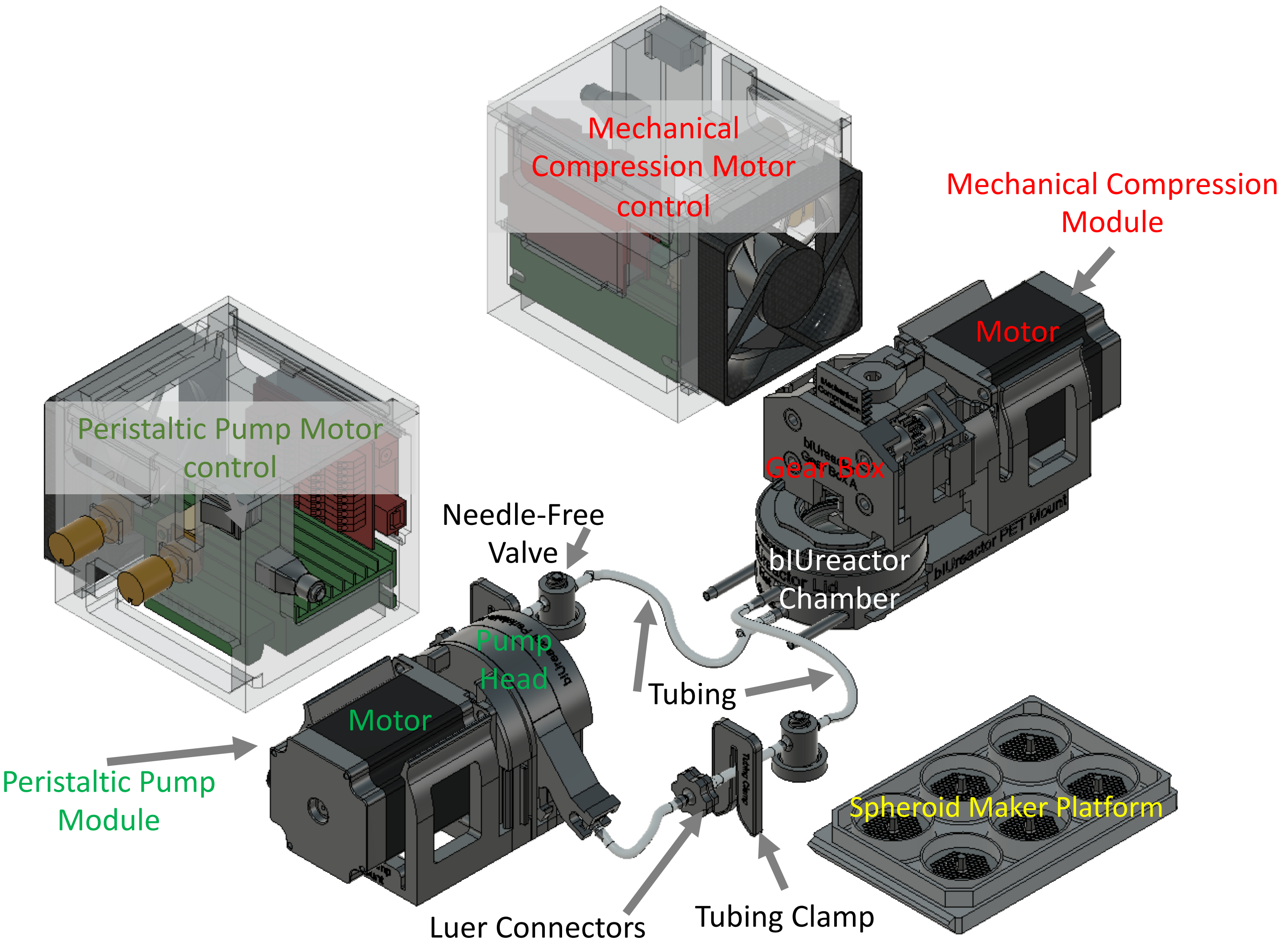

bIUreactor Tissue Culture Chamber Design

The bIUreactor Tissue Culture Chamber (Figure 10) was designed to aseptically contain the SSuPerForM Tissue nested within the SSuPerForM Platen while allowing perfusion through the tissue. The twist locking lid clip mechanism securing the bIUreactor lid to the bIUreactor chamber is designed to close smoothly so as to prevent disturbing the spheroids. A snapping mechanism would be violent enough to knock the spheroids out of the SSuPerForM Platen Central Channel, thus preventing tissue formation. The clear silicone Grommet is enclosed within the bIUreactor Lid and the Grommet Lid to allow viewing of the tissue during culture without needing to open the bioreactor chamber, which would expose the tissue and risking contamination. The two lids are also secured together with twist locking lid clips integrated into the lids such that the grommet is compressed between the lids, forming a seal and preventing contamination. The system is designed such that an over-pressure will escape through the interface between the Grommet and Grommet Lid and seal once pressure is relieved. This design choice prevents overpressure from causing a tube to separate from a connection and potentially drain the bIUreactor completely. The SSuPerForM Platen (Figure 11) is secured to the floor of the bIUreactor Chamber by means of the bottom annular grip, which also forms a labyrinth seal between the outlet port and the Central Channel in the SSuPerForM Platen. The Outlet port at the floor of the bIUreactor Chamber connects to tubing that connects to the Peristaltic Pump and back to the Inlet. The Inlet allows media to enter the chamber but the Top Annular Grip (Figure 11) reduces the effects of media flowing directly into the Central Channel, possibly disrupting the spheroids. The polypropylene Mesh (Mesh opening: 149 µm, Thickness: 193 µm, Spectra/Mesh Woven Filters) sits within the center of the Platen, holding up the SSuPerForM Tissue and allowing flow to pass through it. The Mesh was punched from a larger disk using a 7 mm biopsy punch (WellTech Rapid-Core 7.0)

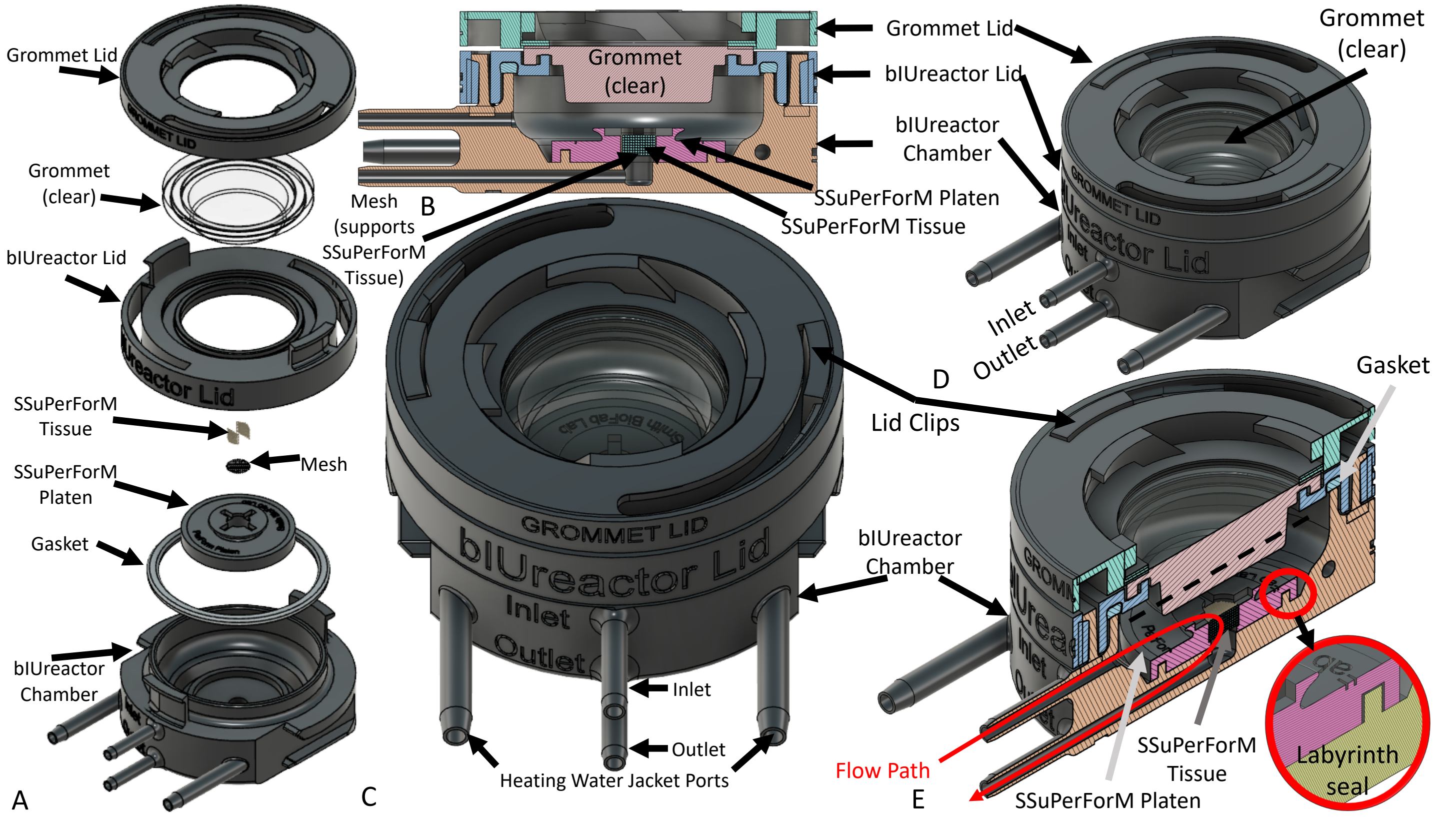

Figure 10. CAD schematic of the bIUreactor Chamber and accompanying components. The bIUreactor is designed to provide an aseptic culture environment, perfusion, and containment to a SSuPerForM tissue. A. Exploded diagram of the bIUreactor Culture Chamber. B. The SSuPer Tissue contained within the center of the SSuPerForM Platen, and thus in line with the flow path, is perfused when the inlet and outlet are connected to a peristaltic pump. C, D, and E. Media flows through the inlet and outlet and mixes within the chamber. The Grommet, which allows aseptic observation of the tissue without opening the containment, is secured to the bIUreactor Lid by the Grommet Lid. The Grommet Lid compresses the Grommet to the bIUreactor Lid, forming a seal. There is a gasket in the bIUreactor lid that prevents leakage from the bIUreactor Chamber and contamination into the bIUreactor. E. The Grommet extends below the

dashed media fill line so there is no additional refractory medium (i.e., air) distorting the view. This feature allows a clear view of the tissue in the bIUreactor Chamber. The SSuPerForM Platen situates in the chamber with its center openings aligned with the opening of the outlet on the chamber floor. The Labyrinth Seal ensures that fluid flow is contained in the center of the platen, and thus, solely through the SSuPerForM tissue, perfusing it. The Tissue is nested within the Platen, which is nested within the bIUreactor in the path of flow. There are water jacket ports for keeping the bIUreactor warm when outside incubator.

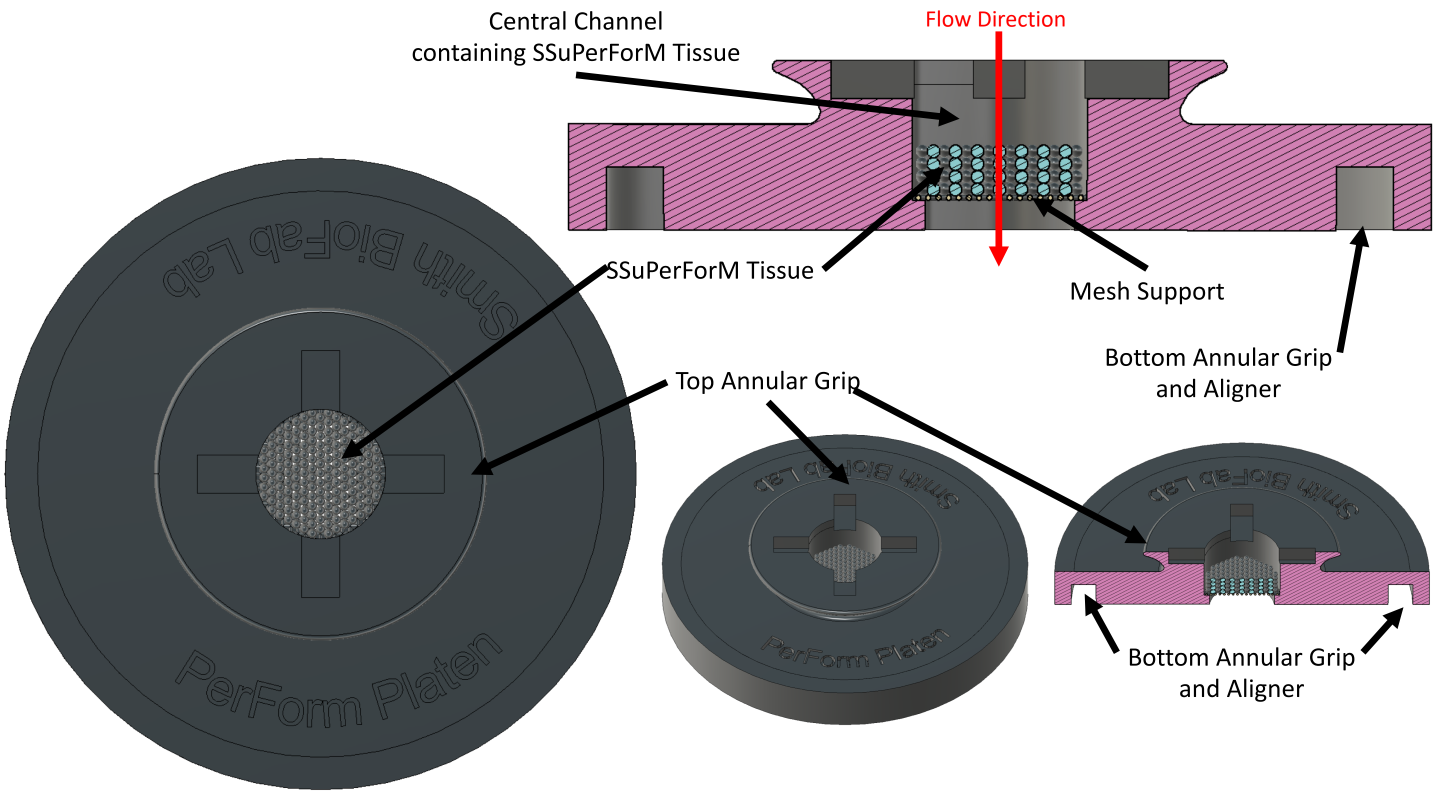

Figure 11. CAD of SSuPerForM Platen with SSuPerForM Tissue. The SSuPerForM Platen, shown here with a SSuPerForM Tissue and mesh in place, is designed to contain and channel perfusive flow through tissue. The Central Channel containing the spheroids contains a mesh that supports the spheroids. Flow is capable of passing through the porous SSuPerForM Tissue and the mesh, allowing perfusion. There are annular grips for aseptically handling the platen, and therefore the tissue, with forceps without risking tissue damage.

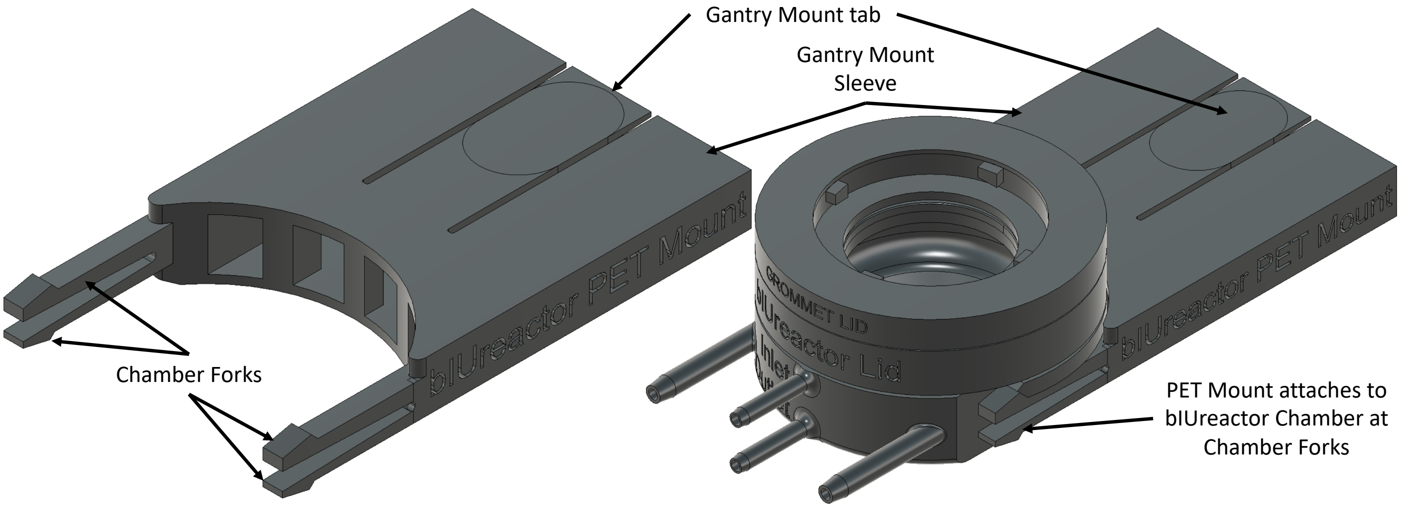

Figure 12. The PET Mount holds the bIUreactor in place in the PET imager. The Chamber Forks slot into openings on the side of the bIUreactor Chamber. The forks are designed for easy chamber detachment from the mount.

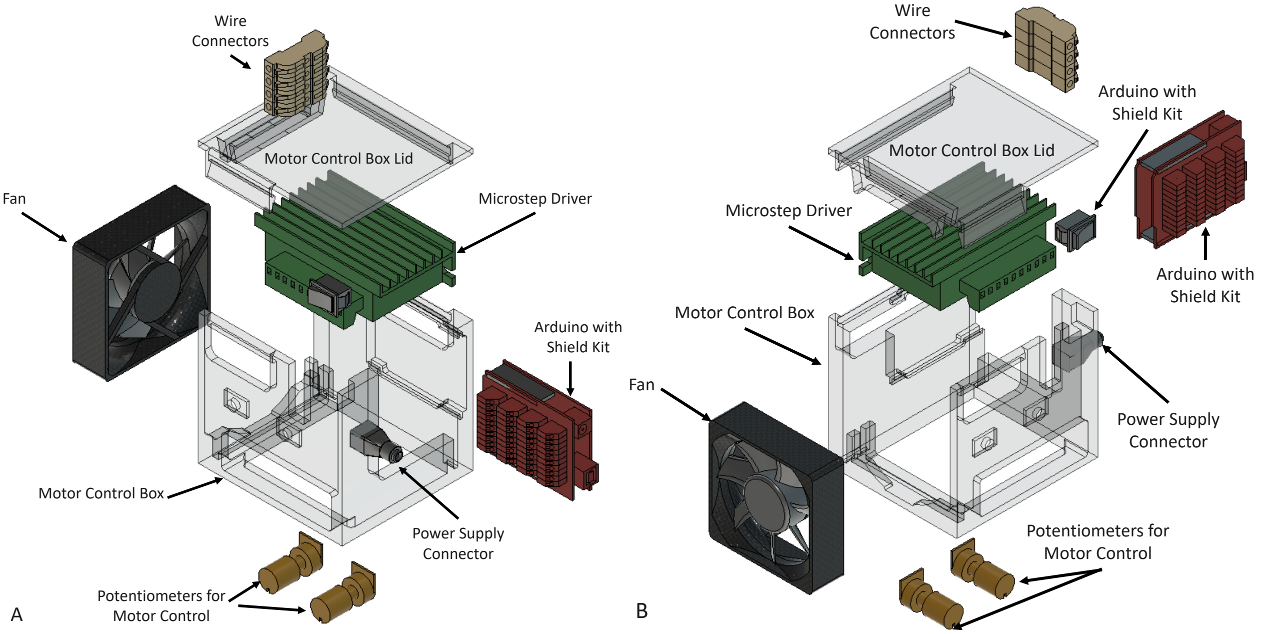

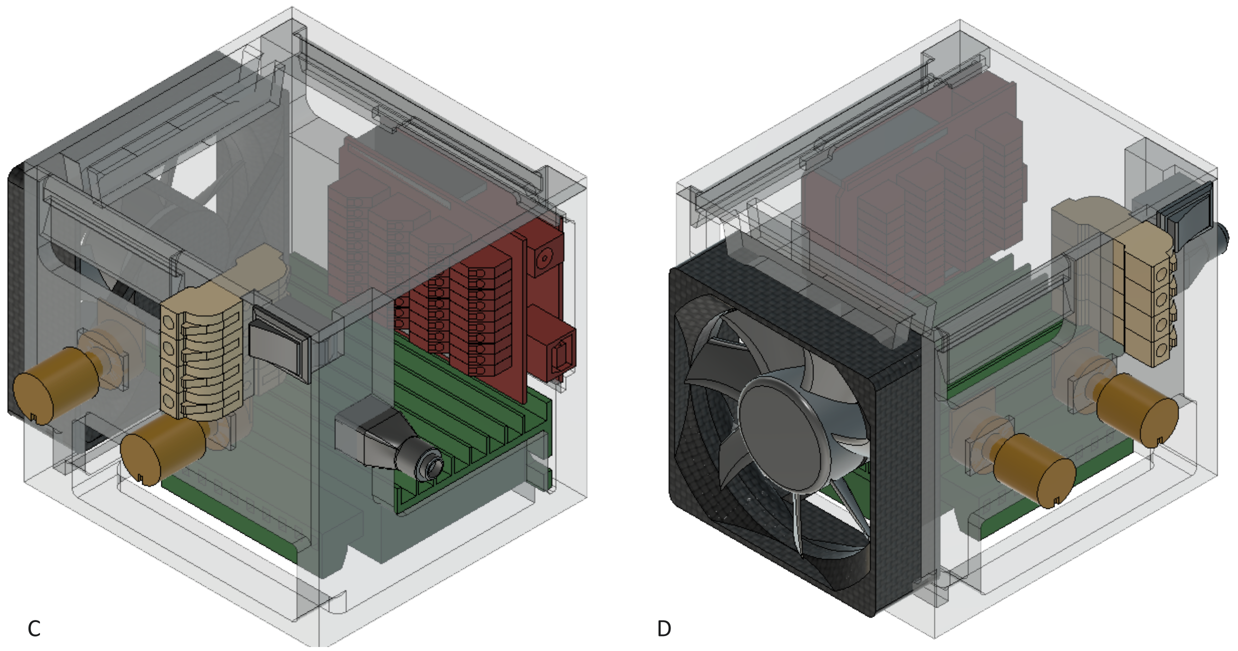

Figure 15. Side (A) and Front (B) exploded view of Motor Control Box for both the Cyclic Mechanical Compression Module and the Peristaltic Pump Motor. The Control system features fixtures for securing a Switch, an Arduino with a Motor Shield, a Micro Step Driver, Wire Connectors, a Power Supply Connector, and a 12 V DC fan.

Cyclic Mechanical Compression Module for bIUreactor Tissue Culture Chamber

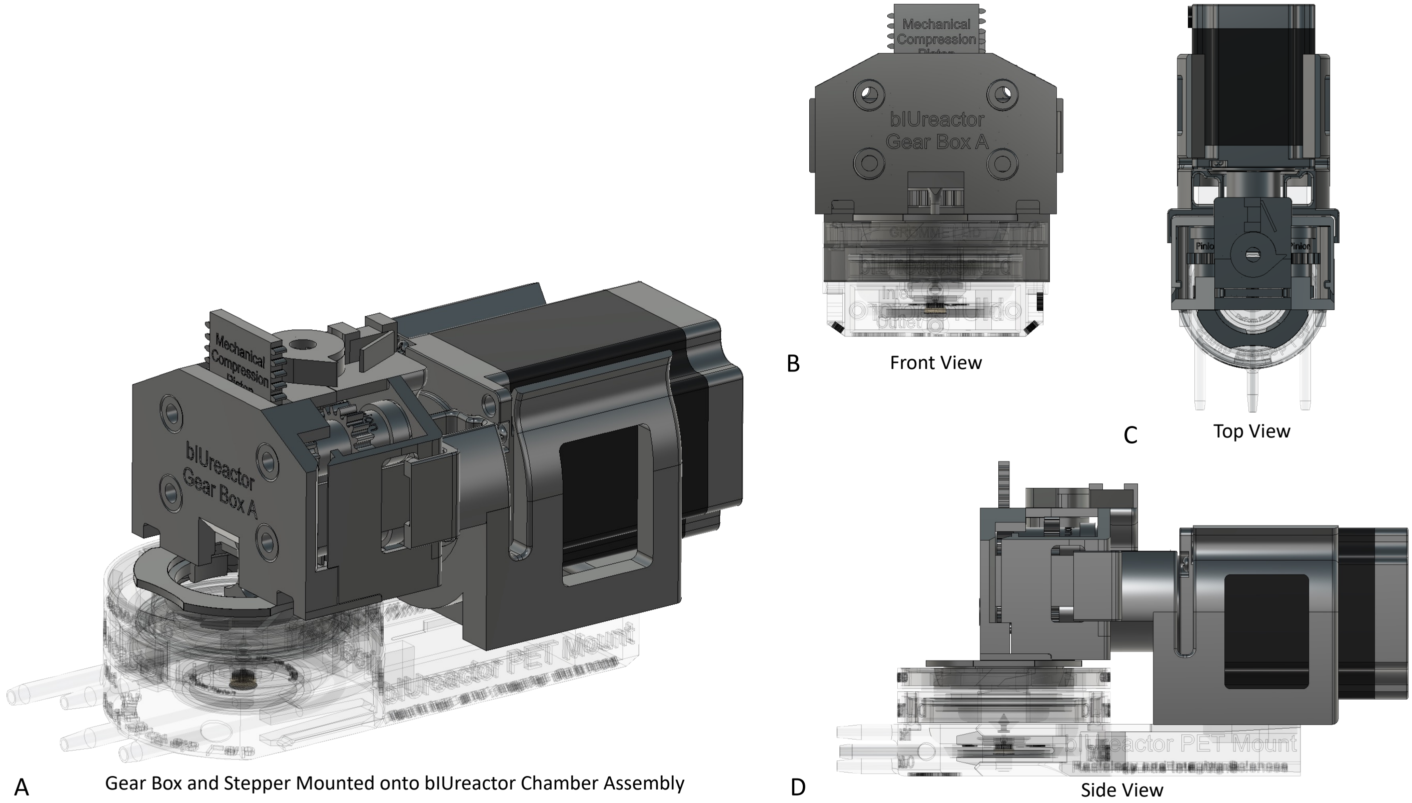

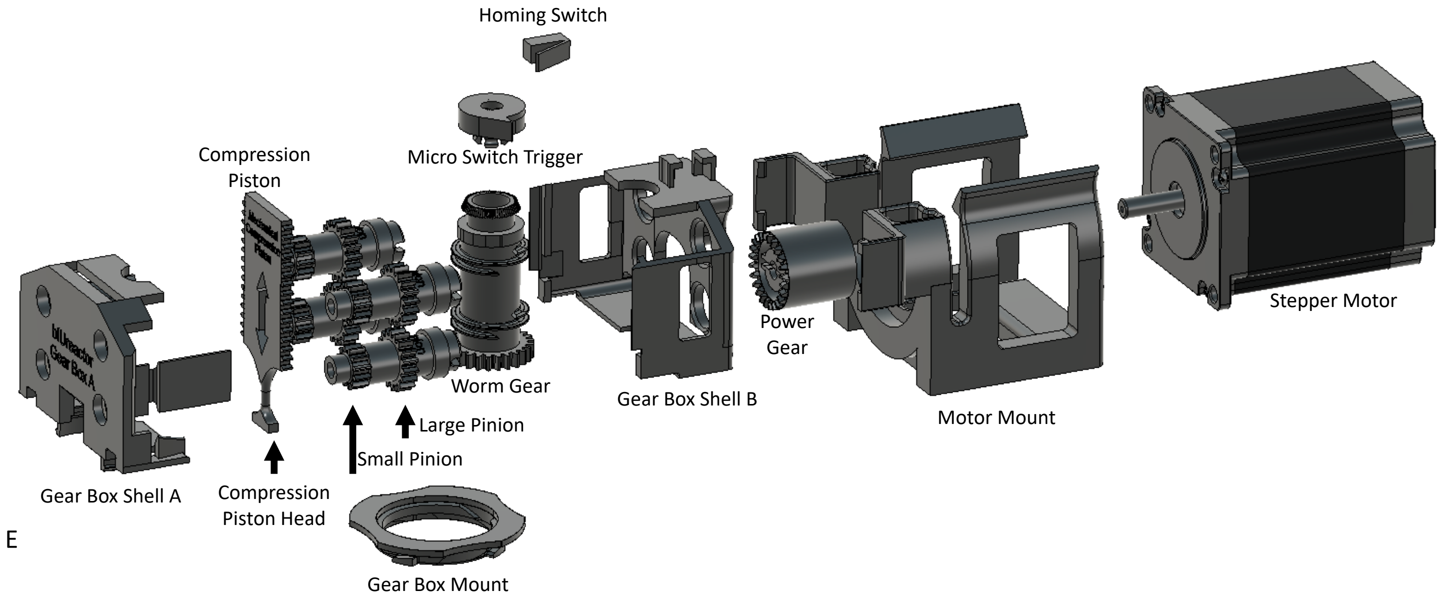

Figure 1. Gear Box Module applies cyclic mechanical compression to the tissue within the bIUreactor Chamber. A. Oblique view of Gear Box and Stepper Motor mounted onto the bIUreactor Chamber Assembly. B. Front view of Gear Box. C. Top view of Gear Box. D. Side view of Gear Box. E. Exploded view showing Gearbox module components.

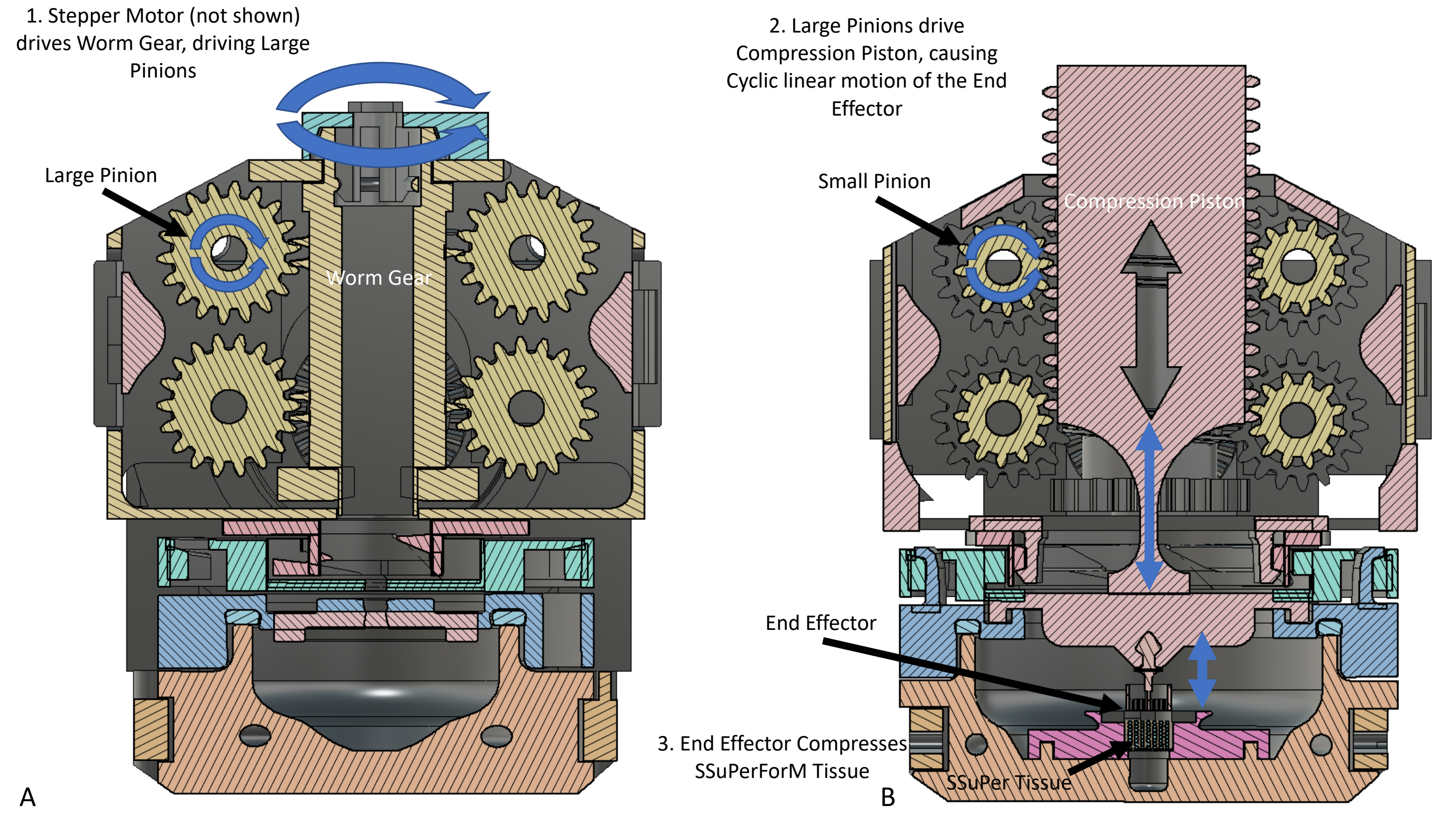

Figure 2. Section Views indicating how the Gear Box Module applies cyclic compression to tissues inside the bIUreactor Chamber. A. Front View Section showing the Worm Gear driving the large Pinions in the Gear Box. B. Front view Section showing Small Pinions driving the Piston in the Gear Box.

The Grommet provides a window into the chamber and features divot designed to accept a vaned End Effector that rests over the tissue within the central channel. The cyclic compression from the piston is then transferred through the Grommet, to the End Effector, and then to the tissue within the Platen. The vanes within the End Effector are designed to direct flow through the effector and into the tissue (Figure), thus allowing simultaneous nutrient perfusion and cyclic

compression. Glycerol (Sigma-Aldrich, St. Louis) was used as a lubricant for the Gear box and the Peristaltic Pump due to its availability in most labs, its heat capacity, and its antimicrobial properties (75–77). Glycerol was brushed onto all friction surfaces within the Gear Box. The parts were then assembled as shown in Figure below.

During perfusion, the tissue axially contracts. Initial test runs are performed to determine the tissue height after 24 hours of perfusion-assisted contraction. The new tissue height and mesh thickness are subtracted from the distance to the top of the platen to determine the Tare Height. Tare Height is the distance the End Effector needs to be displaced in order to be in contact with the Tissue (tare position). The knob at the top of the worm is turned until the end effector is flush with the top of the Platen. The worm/piston gearing ratio is 360 degrees per 2.5 mm. A calibration is performed to set the piston displacement level and loading frequency, thus setting the amount of strain and loading rate, respectively, applied to the tissue. The displacement potentiometer is adjusted, the motor drive is powered on, and the displacement of the end effector is measured. These steps are repeated until the desired displacement is achieved. Similarly, to set the loading frequency, the frequency potentiometer is adjusted, the motor drive is powered on, and the number of cycles per minute is counted. This adjustment is performed until the desired frequency is achieved. The drive system is programmed so that, when powered on, the Piston immediately starts pushing down at the set (tare) displacement and frequency.

Once the system is calibrated and in the Tare position, the Micro Switch Trigger is placed securely onto the Worm Gear with the Trigger holding the Homing Switch in the closed position to set the Home position. As a result, when the drive system is powered on, the Work Gear and Micro Switch Trigger will rotate as the Piston is pushed down. When the Worm Gear returns, the Micro Switch Trigger will actuate the Homing Switch, homing the system and starting the process again. See Supplement 1 for more information regarding Compression Module operation and assembly.

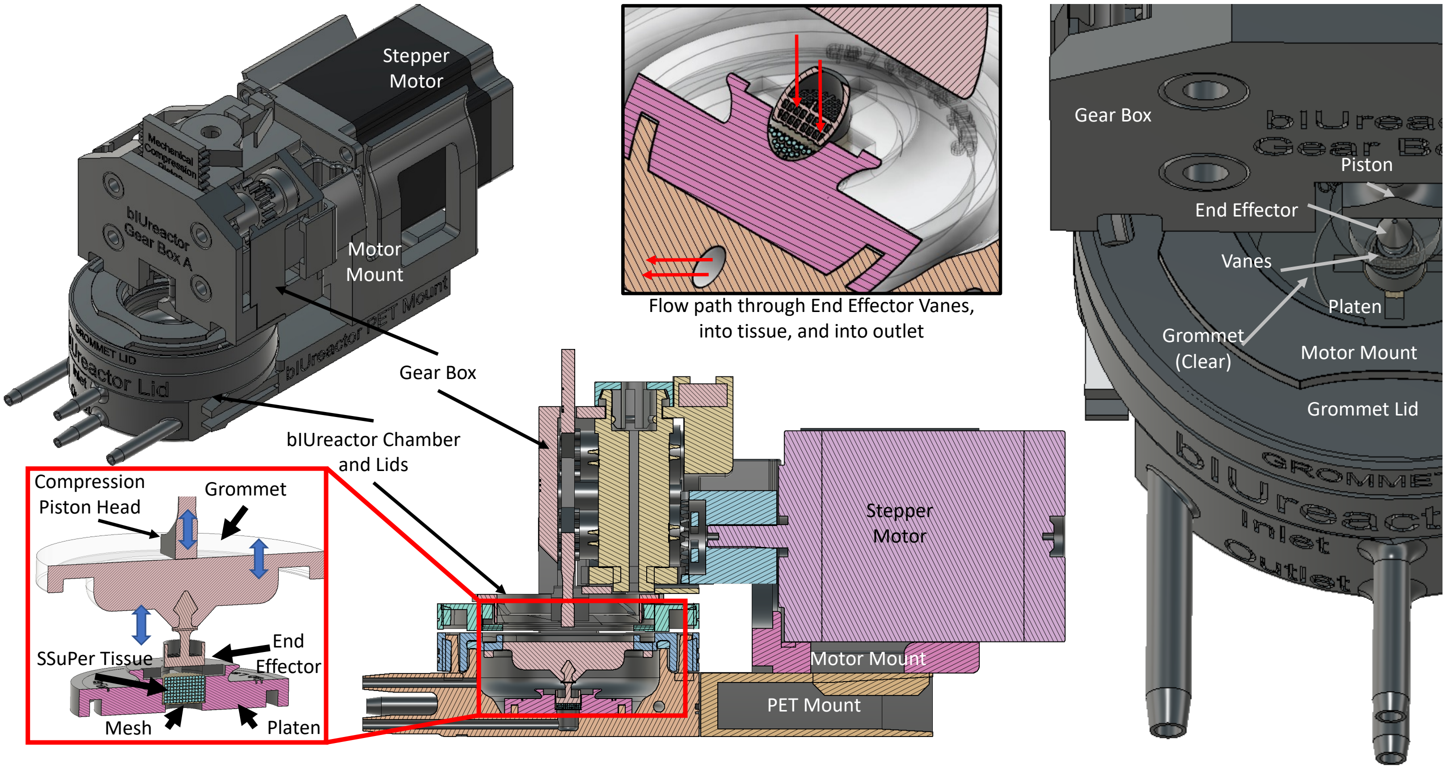

Piston compressing Grommet, and thus the End Effector, compresses the SSuPer Tissue while still permitting perfusion.

Figure 15. A. Gear Box mounted onto bIUreactor. B. Schematic showing the orientation of the End Effector in relation to the Piston Head, Grommet, and SSuPerForm Grommet. C. Flow can pass through the End Effector, allowing simultaneous perfusive flow and compression. D. To simplify calibration and setting Tare Height, the Culture area can be visualized through the Grommet.

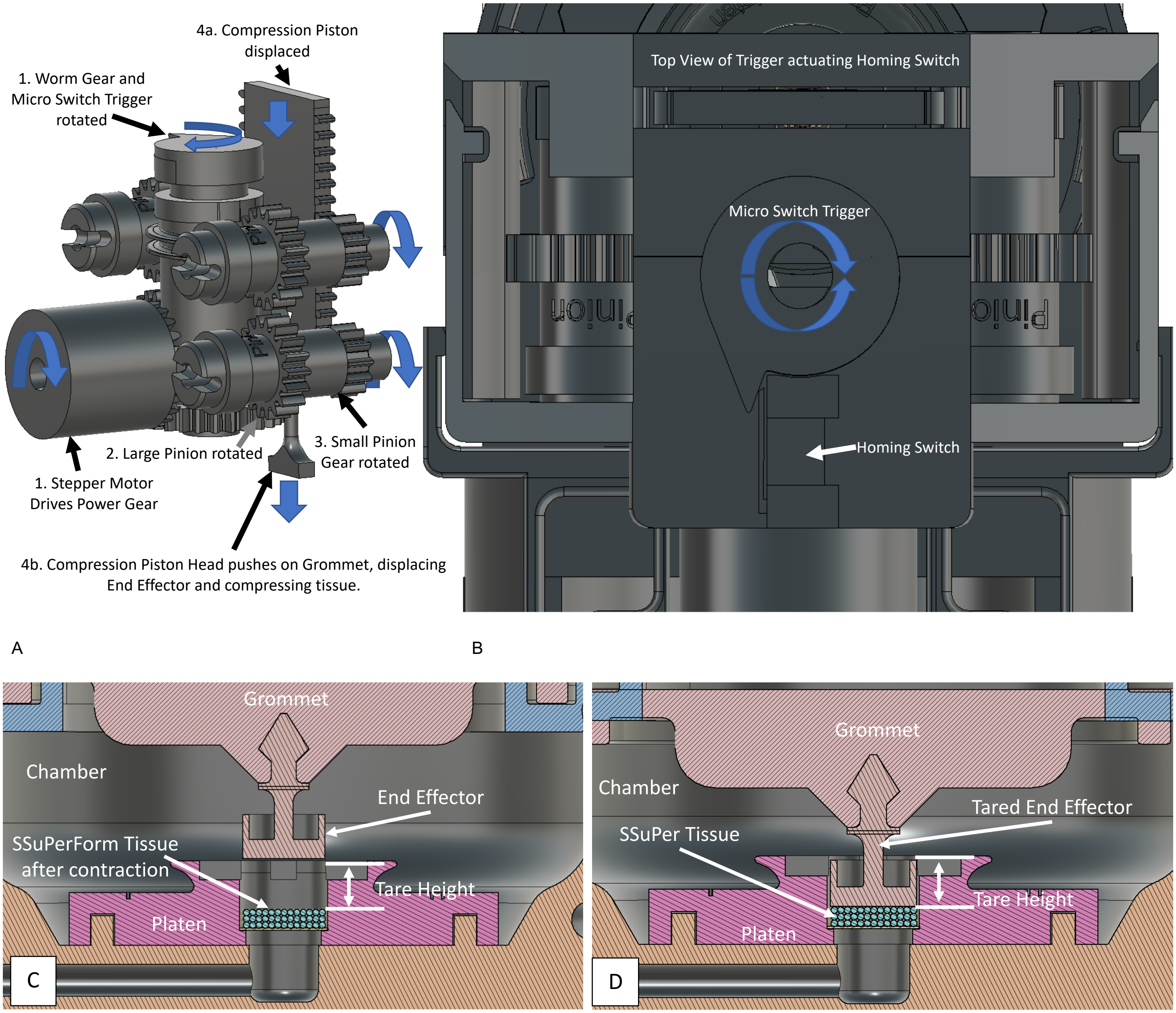

Figure 16. A. The motor drives the (1) Power Gear, which drives the Worm Gear and attached Micro Switch Trigger. The (2) Worm Gear drives the Large Pinions, which transfer power through the Small Pinion which causes the Compression Piston to translate in a linear motion. The linear motion transfers through the Grommet to the End Effector, thus compressing the tissue. B. The Micro Switch Trigger actuates the Homing Switch setting the home position after each stroke cycle. The Piston is displaced 2.5 mm per 360 degrees of Worm Gear rotation. C. To tare the End Effector to the tissue, after initial tissue contraction, the tissues surface is a prescribed distance (Tare Height) from the Tissue. D. By rotating the Worm Gear, the Tare Adjustment puts the End Effector in contact with the Tissue (Tare Position) so that cyclic mechanical compression can be effectively applied. The Micro Switch Trigger is capped onto the Worm Gear so that the Homing Switch is closed. Once the Compression system is adjusted for frequency and displacement and the Tare set, the Motor control system will start cyclically compressing the tissue downward from the Tare Position.

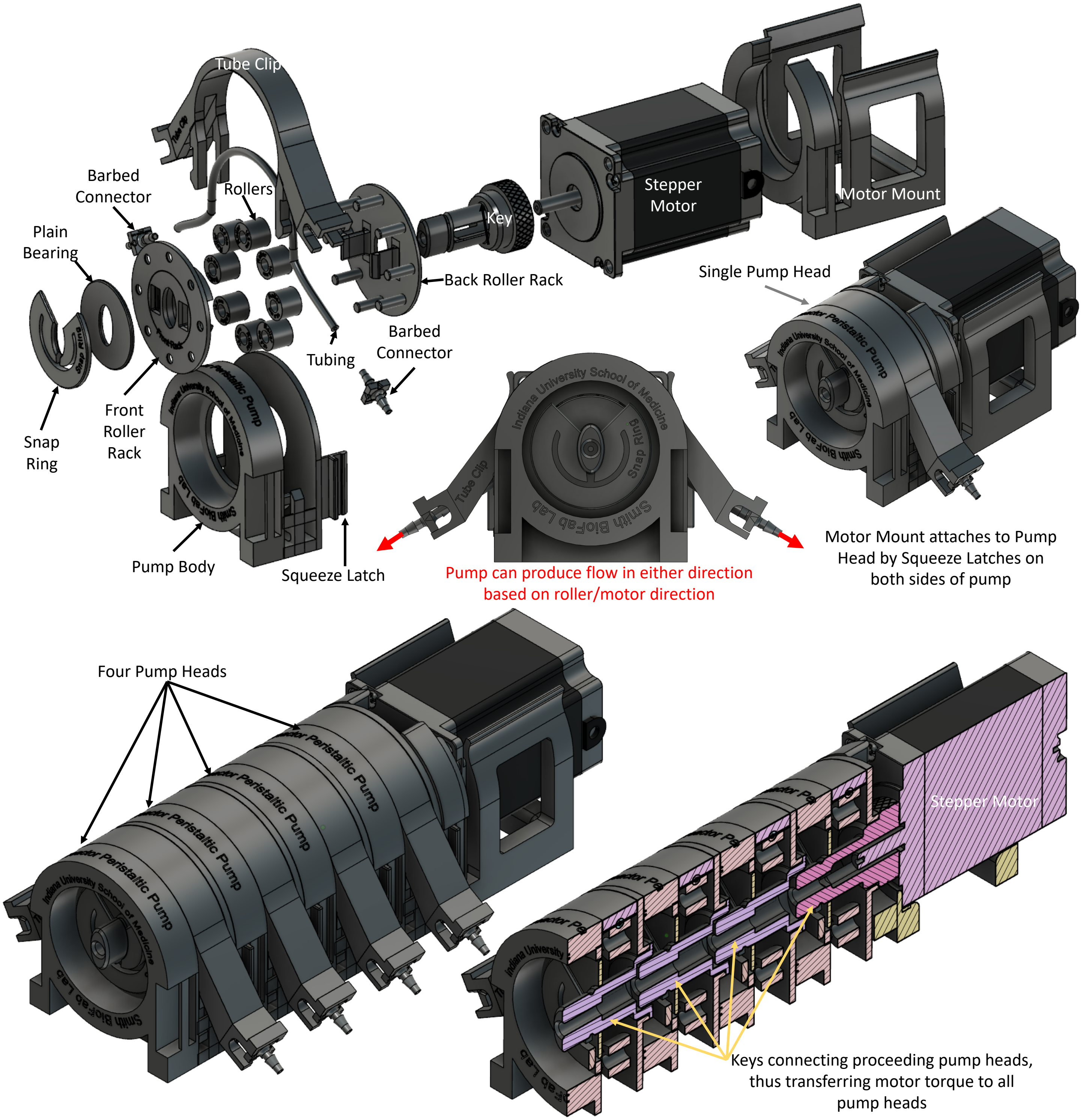

Figure 18. CAD and schematic of Peristaltic Pump. The Peristaltic Pump is designed to be fully disassembled in case parts need to be cleaned or replaced. With the exception of the motor and the tubing, the entire pump is 3D printed and each module (Pump Head or Motor Mount module) can be printed at the same time in a Form 3 desktop 3D printer. Per the bIUreactor Design Philosophy, there are no screws required to assemble the pump and the pump can be assembled or disassembled with no tools. Barbed connectors are included in this illustration to indicate the pump inlets/outlets and where the connectors are integrated. The Pump Head consists of all components except the Stepper Motor and the Motor Mount.

Rapid Prototyping and 3D Printing

Prototyping and Material Selection

To test for fit and basic function, design prototypes were printed in Draft Resin (Formlabs) on either a Form 2 or a Form 3 desktop 3D printer from Formlabs. Once designs were nearing finalization, they were printed in Clear V4 resin (Formlabs), which better matches the mechanical properties of the final material. Final designs were printed in BioMed Clear (Formlabs) on the Form 3B due to the resin's biocompatible, autoclavable, and wear-resistant properties.(59,62) Parts, such as molds, that needed to withstand the high temperature vulcanization (HTV) process were also made from BioMed Clear. After transfer to Preform, the device material was selected in the software, the parts were oriented to minimize the number of internal supports and to fit each module in a single batch. The parts were then transferred from the MacBook to the printer and the parts were allowed to print onto the build platform inside the printer (Figure 2).

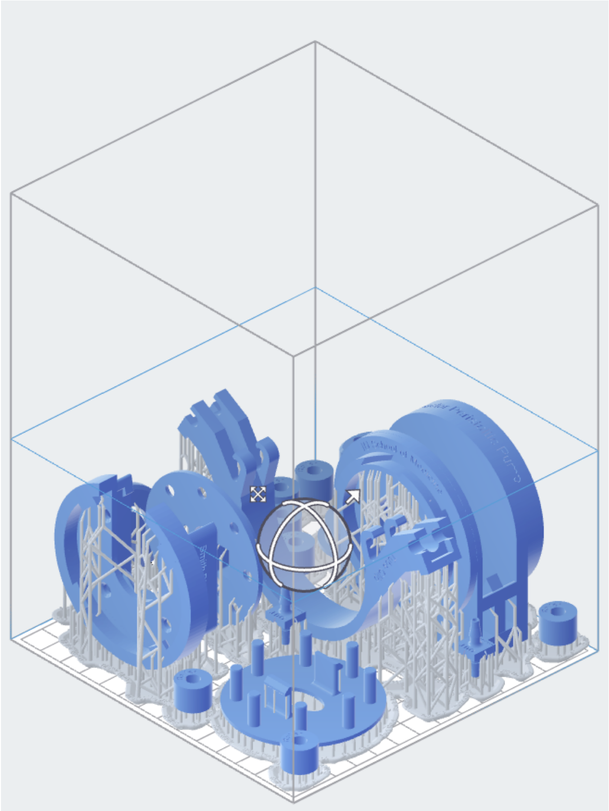

Figure 2. Screen capture from Preform software showing all of the individual 3D printed parts of a Peristaltic Pump head in one print batch. Pump parts are in blue and supports are gray.

Post Processing

When printing completed, the finalized parts were removed from the build platform using a steel putty knife and post-processed. Briefly, parts made from BioMed Clear were washed in 95% isopropanol for 20 minutes in a Form Wash (Formlabs) dedicated to cleaning cell-contacting parts. The parts were washed again in a second 95% isopropanol bath. The parts were then dried using forced air. Biomed Clear parts were then post-cured in a Form Cure for 60 minutes at 60 deg C. Supports were removed from the post-cured parts using flat snips included with the 3D printer.

Casting silicone parts

Silicone parts were made from Elastosil 610 (Wacker), a silicone capable of room temperature vulcanization (RTV) or high temperature vulcanization (HTV). Post-processed molds used for casting were lightly coated with a layer of mold release. The two-part molds were designed to be held together using binder clips and so they were clipped together

after coating. While the mold release settled, the two-part Elastosil resin was mixed, debubbled, and prepared for mold injection. Briefly, the two silicone components were loaded into a 9:1 syringe and pumped through a static mixer into a syringe tube (with the plunger removed) and capped at the bottom with a Luer cap. The tube containing the mixed silicone was centrifuged at 500xg for 5 minutes to remove air bubbles formed during the mixing. The plunger was placed onto the syringe and the cap was removed, making the silicone ready for casting.

Spheroid Maker casting

Molds were designed for the silicone casting of Spheroid Makers, Grommets and Gaskets, and Silicone Inserts for the Needle-Free Valves (Figure 10). The Sprue on the two-part Spheroid Maker Mold allows easy silicone injection with a syringe and vents on the top of the mold permit air to escape during injection so that bubbles do not form. Silicone can be simply poured into the open Gasket and Grommet Molds. The open design of the Grommet Mold leads to a smooth finish top side, which allows for clear view of tissues inside the chamber when the bottom of the Grommet is submerged into culture media (much like an immersion lens on a microscope).

Grommet and Gasket casting

Silicone was pumped into the open molds and the excess was scrapped off using a laboratory spatula to ensure a flat surface. When a Grommet was being made for mechanical compression, the End Effector insert was installed prior to filling. The Gasket Mold Lid was then secured over the Grommet Mold and the filled molds were then vulcanized at 200 deg C for 20 minutes. After vulcanization, the molds were allowed to cool and the silicone parts were gently removed using by hand. Extra silicone that escaped through the mold vent was trimmed away with.

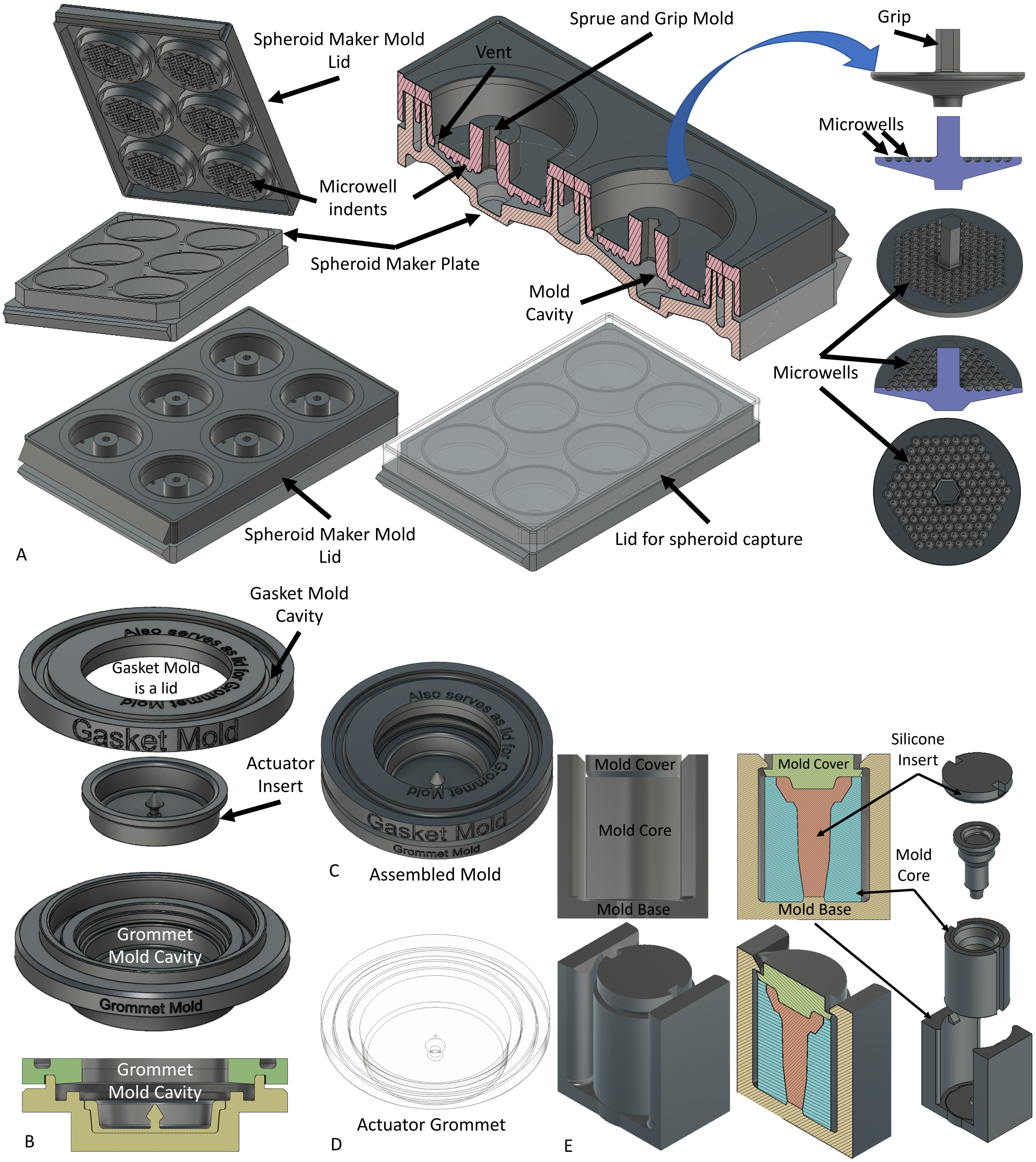

Figure 10. CAD models of molds. A. The Spheroid Maker Mold features a central sprue for silicone injection that also becomes the Grip, as well as vents for releasing trapped air. The part names are

debossed to help users identify them. B. Exploded diagram of Grommet and Gasket Mold. The Grommet and Gasket mold is designed for casting two grommet designs and the gasket, each of which aseptically seal the bIUreactor. The Gasket Mold is used to cast silicone gaskets and also serves as the lid to the Grommet Mold. Without the Actuator insert, the resulting Grommet simply provides a window into the chamber while sealing the chamber from the external environment. When the Actuator Insert is included, the resulting Actuator Grommet acts as a window and seal while holding the vaned End Effector which transfers cyclic compressive forces from the gearbox to the tissues within the bIUreactor Chamber. C. Assembled Gasket and Grommet Mold. D. Resulting casted Actuator Grommet. E. Mold for Silicone Insert used in Needle-Free Valve.

Spheroid Maker

The Spheroid Maker Plate and Lid possess six sets of Spheroid Maker molds arranged in the 6-well plate design. The Plate serves as a mold and holder for the Spheroid Maker as well as a Collector for Spheroid production. To cast Spheroid Makers, the lid and plate were coated with mold release and assembled together, Silicone was injected into the mold cavity and plates were either centrifuged at 500 rpm for 4 hours and then the filled molds were then vulcanized at 200 deg C for 20-30 minutes. The Spheroid Makers were then removed and they, along with the plate, were cleaned with soap and water to remove residue and prepare them for Spheroid Production. See Supplement 1.

The Spheroid Maker features a field of 120 microwells under a reservoir and grips on the top and bottom. The grips permit easy forceps handling since the device needs to be aseptically inverted during use. A 6-well Collector plate was also produced to contain the Spheroid Makers and to condense the spheroids into a centralized bolus after Spheroid formation.

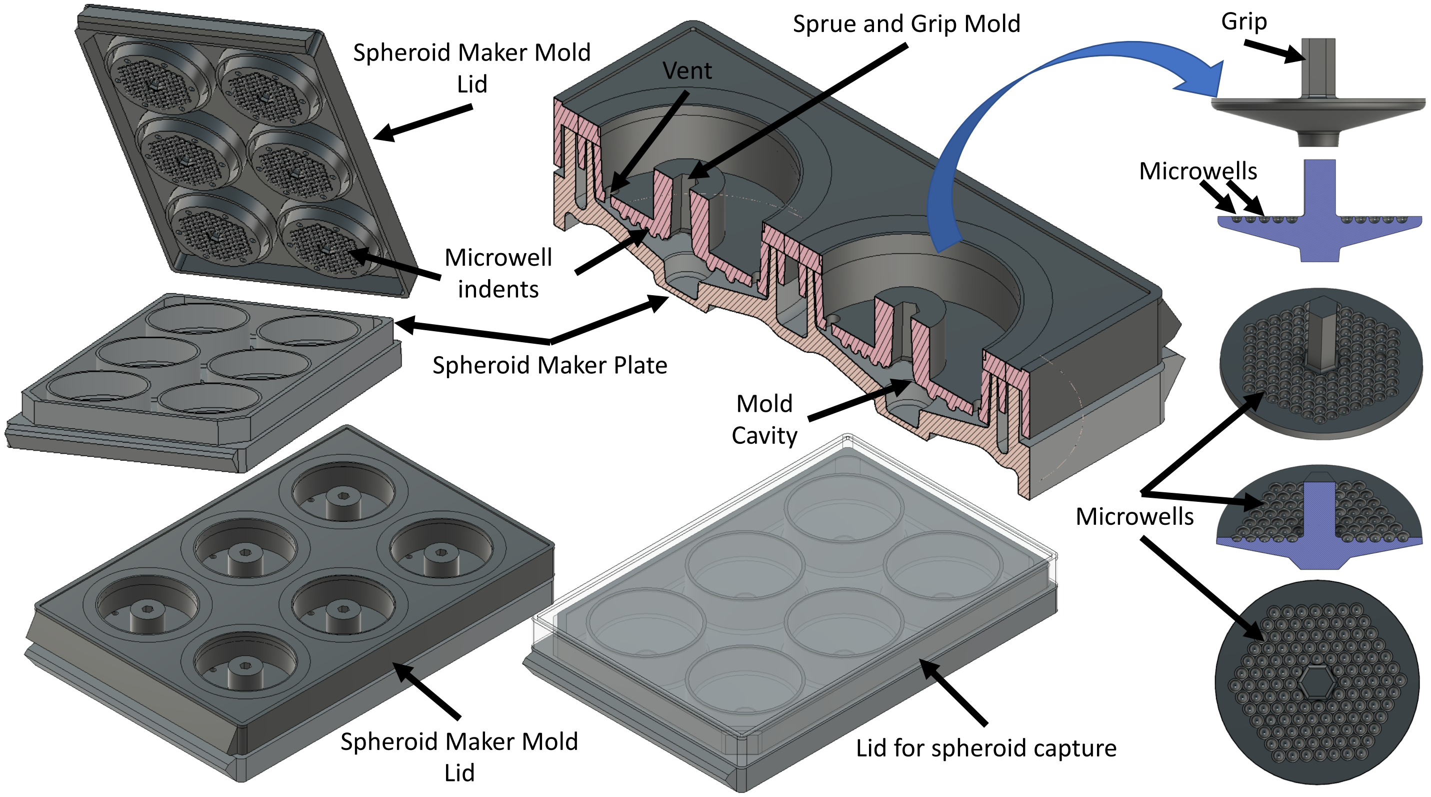

Figure 8. CAD of Spheroid Maker Mold Lid, Spheroid Maker Mold Plate, and Spheroid Maker. The Plate serves as a mold for the Spheroid Maker as well as plate for Spheroid production. To cast Spheroid Makers, the Lid and Plate are coated with mold release. Since silicone is normally clear, silicone parts in the figures below are rendered in gray so their features can be observed. The Spheroid Maker features microwells for spheroid formation and a central grip for aseptic handling with forceps. The Spheroid Collector features a central collector concavity the spheriods will fall into during centrifugation out of the Spheroid Maker. There are annular grips on the top and bottom designed for aseptic handling with forceps. The part is debossed with its part name and instructions for use with the Spheroid Maker.

Control and Circuitry for Cyclic Mechanical Compression motor and Peristaltic Pump motor

The components used to provide controlled rotational torque to the Gear Box and the Peristaltic Pump are listed and described in Supplement 3. Circuit design for the Mechanical Actuator and the Peristaltic Pump are provided below.

Normally, wiring is secured using a low-melting-temperature metal (solder) which is heated to its melting temperature at the junction of a wire and contacts on the circuit board and allowed to cool into a solid junction. Soldering requires an electrically-powered heat source and significant skill, which may be beyond the skill of the bIUreactor user. Therefore, the Shield Kit (Figure) is used to secure wiring between the Arduino and the stepper motor controller because it relies on simple push-in spring connectors to secure the wiring instead of complicated and potentially hazardous soldering. Similarly, Lever Wire Nut Connectors (not pictured) were used to connect wires to each other in the circuit to ensure a stable, protected wire circuit.

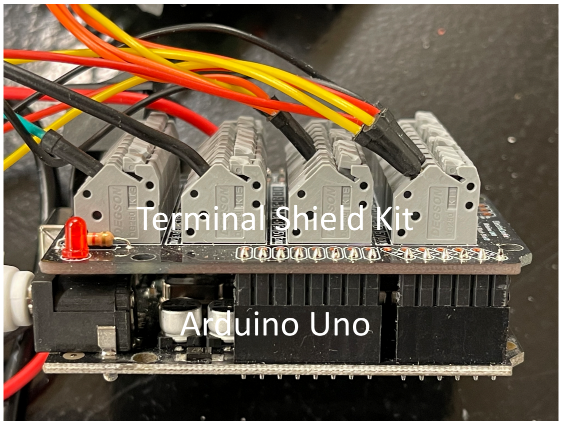

Figure 10. Terminal Shield Kit mounted on top of Arduino UNO. The Shield Kit secures wiring between the Arduino and the Microstep Driver using simple push-in spring connectors instead of soldering.

The circuit diagrams used to drive the Gear Box and the Peristaltic Pump are shown below:

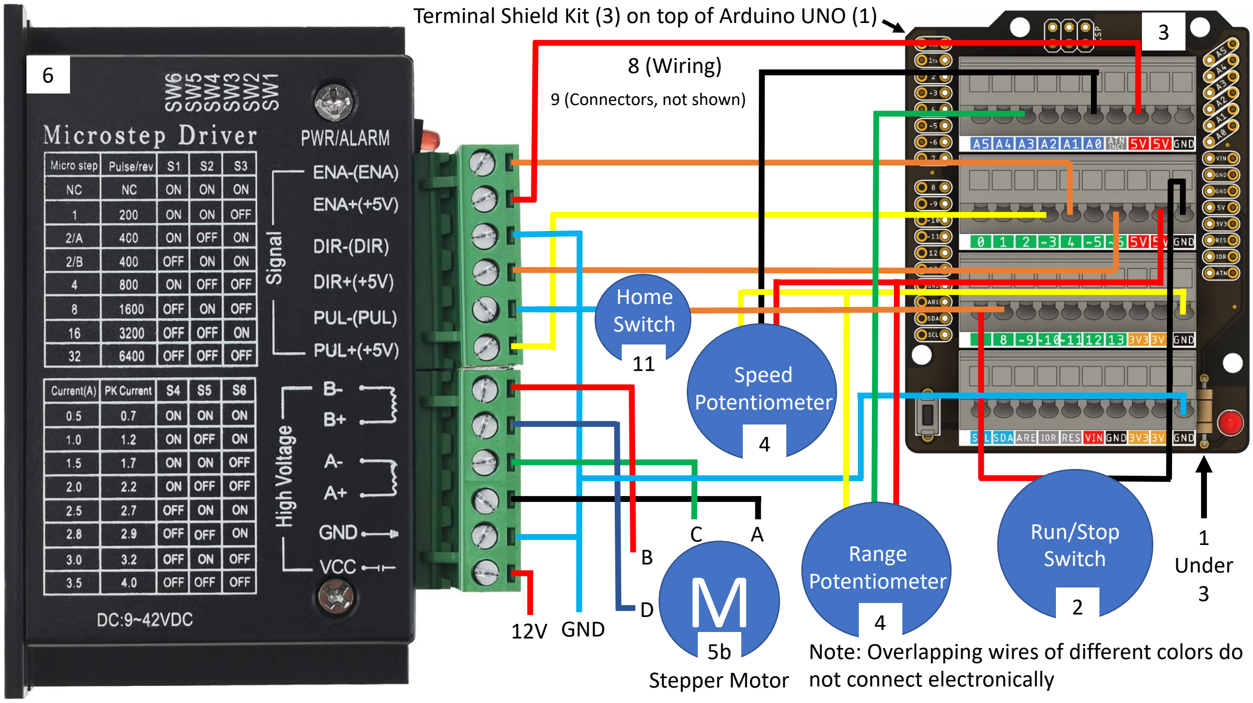

Figure 11. Cyclic Mechanical Compression Module Controller and circuit.The potentiometer for Speed controls the cyclic rate of compression, while the potentiometer for Range controls the maximum range of angle travelled during compression. The Run/Stop Switch starts and stops motor. The user can change Speed and Range only when the button is in the STOP position and these new settings will take immediate effect from the current motor position when the button is in the RUN position. The user must calibrate speed and range.

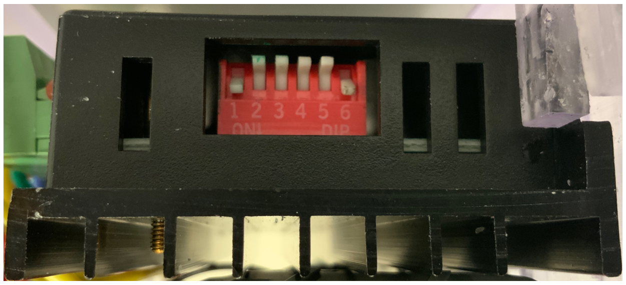

Figure 12. Microstep Driver DIP switch configuration for the Mechanical Compression Module Motor.

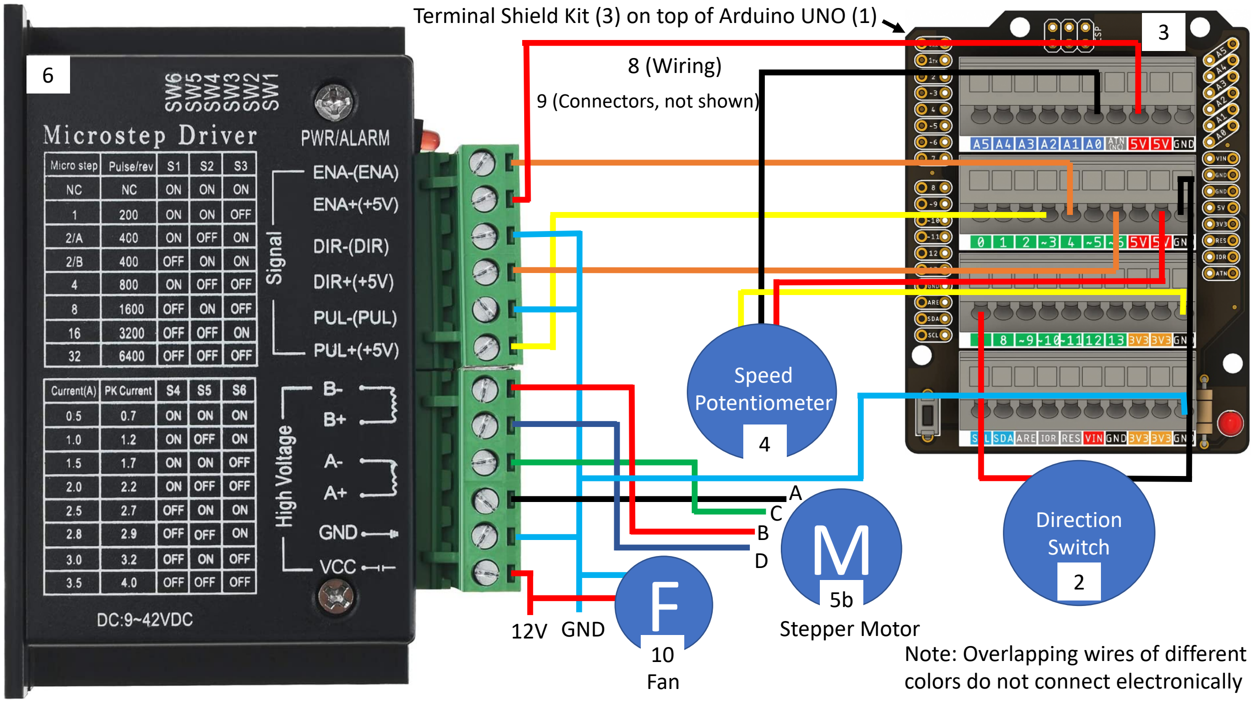

Figure 13. Peristaltic Pump Controller and circuit. The potentiometer for Speed controls the pump speed. The Direction Switch controls running the direction of flow – changing it to the opposite position reverses the flow. The user must calibrate flow rate based on rotational speed.

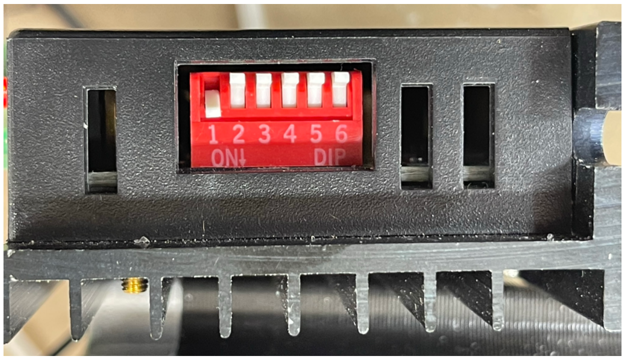

Figure 14. Microstep Driver DIP switch configuration for the Peristaltic Pump Motor

Cyclic Compression module testing

The ability of the Cyclic Compression Module to provide consistent compression over the course of five 5 minutes sessions while simultaneously providing perfusive flow was tested. A calibration was performed to set the piston

displacement level and loading frequency applied to the SSuPerForM tissue. The displacement potentiometer was adjusted, the motor drive was powered on, and the displacement of the Piston was measured using digital Vernier calipers. These steps were repeated until the desired displacement (1.25 mm) was achieved. Similarly, to set the loading frequency, the frequency potentiometer was adjusted, the motor drive was powered on, and the number of cycles per minute (cpm) was counted. These steps were performed until the desired frequency (18 cpm) was achieved. Simultaneous Cyclic Compression and perfusion of the simulated SSuPerForM Tissue was achieved. The bIUreactor was loaded with a Platen and a simulated SSuPerForM Tissue, coupled to the Compression Module, and placed inside of a cell culture incubator set to 37 Deg C. The Compression Module was set to move the Piston at a displacement of 1.25 mm and a loading rate of 18 cpm for 5 minutes(63,64). This was performed a total of 5 times to simulate a 5 day experiment. At the start of each run and after 5 minutes, distance of the top of the Piston from the top of the Gearbox in the crest and wave positions were measured using Vernier calipers. After testing, qualitative analysis was made to determine the degree of wear on dynamic contact or friction contact parts (i.e., gears, and bearing surfaces).

Pump testing

The ability of the pump to provide perfusive flow for up to 5 days was tested. Four sets of bIUreactor Chambers were attached to a Peristaltic Pump setup with 4 Pump Heads. The system was operated with a pump flow rate of 10 ml/minute. The amount of water collected in a graduate test tube over the course of one minute was used to determine the flow rate at the start and the end of the 5 day study. Hourly, photographic and thermal images of the Pump Heads, the Stepper Motor, and Control Box were collected using a FLIR thermal camera (FLIR, Model FLIR-E6390, Wilsonville, OR) to track the system temperature. After 5 days of pump operation, the flow rate was measured and compared to the initial 10 ml/minute flow rate. An analysis was made to determine the degree of wear on the tubing and wear surfaces (i.e., rollers, Front and Back Rack contact surfaces, the inner surface of the snap ring, and the outer surface of the key base). Wear is expected and some parts may need to be replaced, it is important to know which parts should be replaced after a study and which should be kept in order to maintain performance while minimizing waste.

SSuPerForM Tissue culture using the complete bIUreactor platform

bIUreactor Chamber and flow circuit preparation

Prior to first autoclaving, before being used in a tissue culture study, Tygon 3350 silicone tubing (Tubing, 1.52 mm inner diameter, Cole-Parmer Ismatec, Wertheim, Germany) was used to make the flow circuit between the Peristaltic Pump and the bIUreactor Tissue Culture Chamber (Chamber) (Figure). Two sections of tubing (300 cm long, each, Port Tubing) were attached to the inlet and outlet, respectively, of the perfusion chamber. The free ends were then attached to a series of 5 tubing sections (Peristaltic Tubing), 145 mm in length, with each section in the series attached by a Barbed Connector or Barbed Luer (male and female) Connectors. Near the Inlet and Outlets, Needle-Free Valves were placed in the flow circuit to facilitate medium transfer into and out of the Tubing Circuit. The bIUreactor Chamber was then fully submerged in a 70-80% ethanol bath(65)(66)(67), along with the bIUreactor Lid, Grommet, Grommet Lid, SSuPerForM Platen, and the Mesh. The Tubing was connected to the Peristaltic Pump and ethanol was pumped through the Chamber for a minimum of 1 hour, followed by submersion and pumping with water. The parts were then air dried.

Autoclaving

To autoclave for use in a study, the tissue culture bioreactor was completely assembled by placing the Mesh inside of the SSuPerForM platen, securing the platen in the center of the tissue culture, and placing the Lids and Grommet atop the tissue culture chamber (without twist-locking them into place). The entire assembly underwent steam autoclaving at 121 deg C with a 15-minute sterilization phase and a 5-minute dry phase. Following sterilization, the assembly was placed into a tissue culture hood and allowed to cool. All connections that may have loosened during the autoclaving

processes were checked and tightened. The Grommet lid was twisted on top of the bIUreactor Lid, thereby securing the bIUreactor Lid to the bIUreactor Chamber, securing the Grommet Lid to the bIUreactor lid, and securing the Grommet into place. The Grommet Lid-Grommet-bIUreactor Lid Assembly (GGB Assembly) was aseptically removed from the bIUreactor Chamber to prepare the tissue culture chamber for SSuPerForM Tissue Production.

Spheroid Microtissue Production

Self-Supporting Perfused during Formation and Maturation (SSuPerForM) Tissue constructs were made using cell aggregates (spheroids) made from murine IDG-SW3 cells. IDG-SW3 cells are late osteoblasts, which produce large amounts of collagen that mineralizes into a bone-like structure in culture(68,69). SSuPerForM tissue structures can undergo and withstand perfusion immediately upon fabrication and throughout maturation. The spheroid microtissues are comprised only of cells and the extracellular matrix they secrete, making them ideally-suited for forming larger tissues and further maturation.(13,29,39,70,71) Briefly, autoclaved 120-microwell spheroid makers were placed into custom designed and 3D printed 6-well plates, reservoir side up. Next, 4.8 million IDG-SW3 cells (pn=23-25) were suspended in 2 ml of control culture medium (α-modified Eagle's medium [α-MEM] plus 10% fetal bovine serum [FBS] and 1% penicillin/streptomycin) and pipetted into the Spheroid Maker. The plate was covered with a lid, centrifuged at 500xg for 5 minutes to force the cells into the microwells, and then incubated overnight. After overnight incubation, spheroids were formed in the spheroid maker microwells. To make it easy to remove and transfer the spheroids, the handles and the annular grip on the spheroid makers were used to invert spheroid makers inside of the 6-well plate. The plate was centrifuged again so the spheroids would fall into the center of each well of the 6-well plate. The spheroids could then be collected with a simple draw of a 1000 µL micropipettor. A total of 24 Spheroid Makers was used to produce spheroids.

PET Imaging and Imaging Analysis

After five days of culture the Pump was briefly stopped and disconnected so the chamber could be removed from the incubator. The pump was then reconnected and the assembly was prepared for tissue metabolism kinetic analysis using the IndyPET III Positron Emission Tomography (PET) scanner (73). The tissue culture chamber was mounted to the IndyPET III scanner gantry by means of a custom mount so the culture chamber could be placed in the center of the PET scanner bore (Figure 5). Once inside the PET bore, a 60-minute PET scan was initiated. The pump was then stopped briefly and a 3-5 ml bolus of media containing a dose of 138 µCi (5.106 MBq) [^{18}F] FDG radiotracer was aseptically injected into the inlet-side stopcock, and a matching bolus of media from the chamber was removed through the outlet stopcock. The pump was restarted to provide perfusion and to circulate the radiotracer into the media and the tissue sample within the bioreactor. After 40 minutes, the pump was stopped and 30 ml of fresh media flush was performed (with a complementary volume of radiotracer-doped media removed) at 10 ml/min followed by a second 30 ml flush at 5 ml/min. The flushes removed excess [^{18}F] FDG, leaving only the radiotracer taken up by the tissue sample within the bioreactor. See Supplement 2. PET images were reconstructed using a Filtered Backprojection Algorithm into a temporal sequence of images to enable visualization of the distribution of Flourine-18 during the recirculation, washout, and tissue retention phases of the study.

Media Exchange or Radiotracer Injection

To replace nutrient-depleted media, Tubing Clamps are first mounted proximal to the Peristaltic Pump Tubing, preventing backflow. The Needle-Free Valves are then cleaned with alcohol wipes to remove contaminants. A syringe filled with 20-30 ml of fresh media (Fresh Syringe) is connected to the Needle-Free Valve on the Inlet side and an empty, closed syringe (Collector Syringe) is connected to the Needle-Free Valve on the Outlet side (Figure 3). Media or radiotracer was pushed out of the Fresh Syringe such that fresh media flows into the Culture Chamber and depleted media flows out of the Chamber into the Collector Syringe. (Figure) The Collector Syringe may be drawn at the same time to support drawing out depleted media and prevent excessive pressure build up. Once the media volume has been

fully withdrawn, the syringes are removed, any excess media was wiped clean from the valves, the Tubing Clamps were removed, and the Pump was restarted to reintroduce nutrient perfusion.

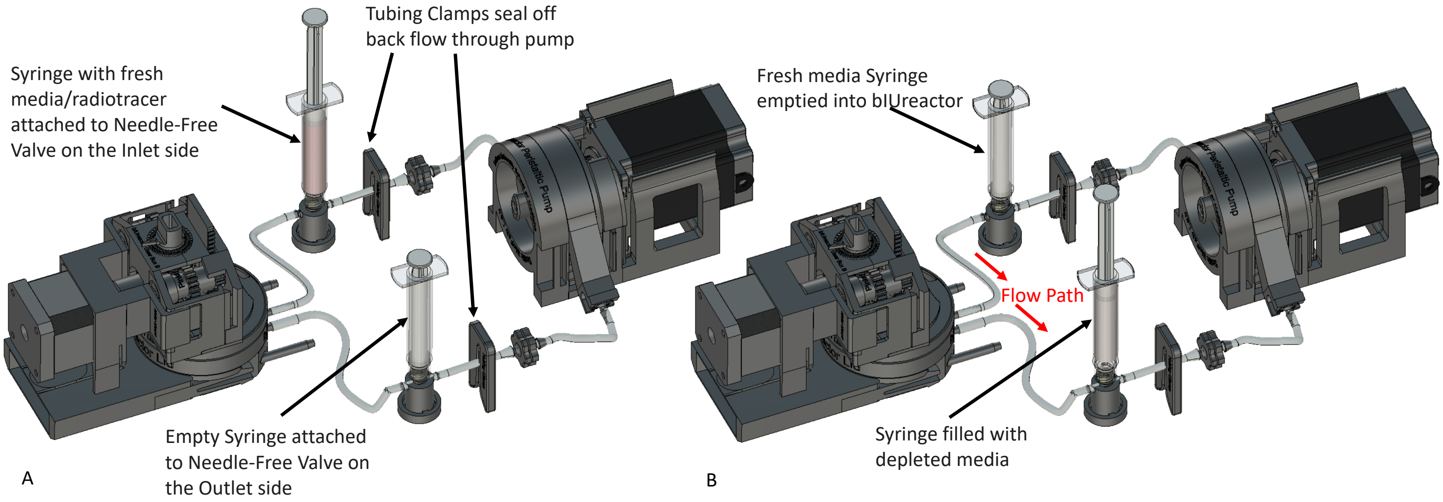

Figure 3. Configuration for exchanging media or injecting a radiotracer. A. The syringe with fresh media or radiotracer is attached to the inlet-side Needle-Free Valve and an empty syringe is attached to the outlet-side needle-free valve. The Tubing Clamps seal off back flow through the Pump. B. Fresh media or radiotracer is flowed into the bIUreactor and the outlet-side syringe is drawn up at the same rate, drawing up spent media.

Cleaning Post-Experiment

To clean the parts after a study, all components except for the Mesh and Silicone Tubing were disassembled and washed with soapy water. The bIUreactor Chamber Ports, the Barbed Connectors, and other connectors were cleaned using 1.3 mm to 2 mm diameter cleaning brushes. The silicone Tubing was washed with 70% ethanol, and the Mesh was discarded. All parts were then rinsed with deionized water. The parts were then allowed to air dry until ready for next autoclaving and use. Parts were not assembled or autoclaved until dry.

Tissue removal

At the end of the study, the platen containing the SSuPerForM tissue was removed by gripping the platen with forceps. The platen was aligned over the central post of the tissue podium and the platen was then gently lowered over the stalk. This action pushed the mesh, and the tissue it was supporting, up and out of the platen. The tissue was then gently removed from the mesh using a pair of forceps or prepared with the mesh in place. (Supplement 2 Figure)

Flow Circuit Mechanical Control

Barbed Connectors (Figure 6) used to adjoin tubing within the flow circuit are specially designed to indicate the flow direction. The Barbed Connectors fit securely to the Connector Holders on the ends of the Peristaltic Pump Tube Clip (Figure 7), simplifying setup and helping ensure the flow circuit is set up correctly. Barbed Luer Hose Connectors (male and female) and Luer Caps (male and female) (Figure 8), were designed for easy connection to tubing and/or for capping tubes. The large knurls on the bodies of the connectors were designed to ease grip and handling while wearing gloves in a wet environment. Tubing Clamps (Figure 9) are used to prevent possibly disruptive flow through Tubing when the bIUreactor Chamber is being handled or when media is being transferred through the stopcock. The Clamp is designed for use on several tube diameters and they are designed to fit through the opening in the back of the incubator. There is a slight raised lip around the perimeter of the clamp to facilitate easy grip and handling. Descriptions of remaining components, including the Tubing Clamps, Needle-Free Valves, and Luer Connectors, and molds are provided in Supplemental Information.

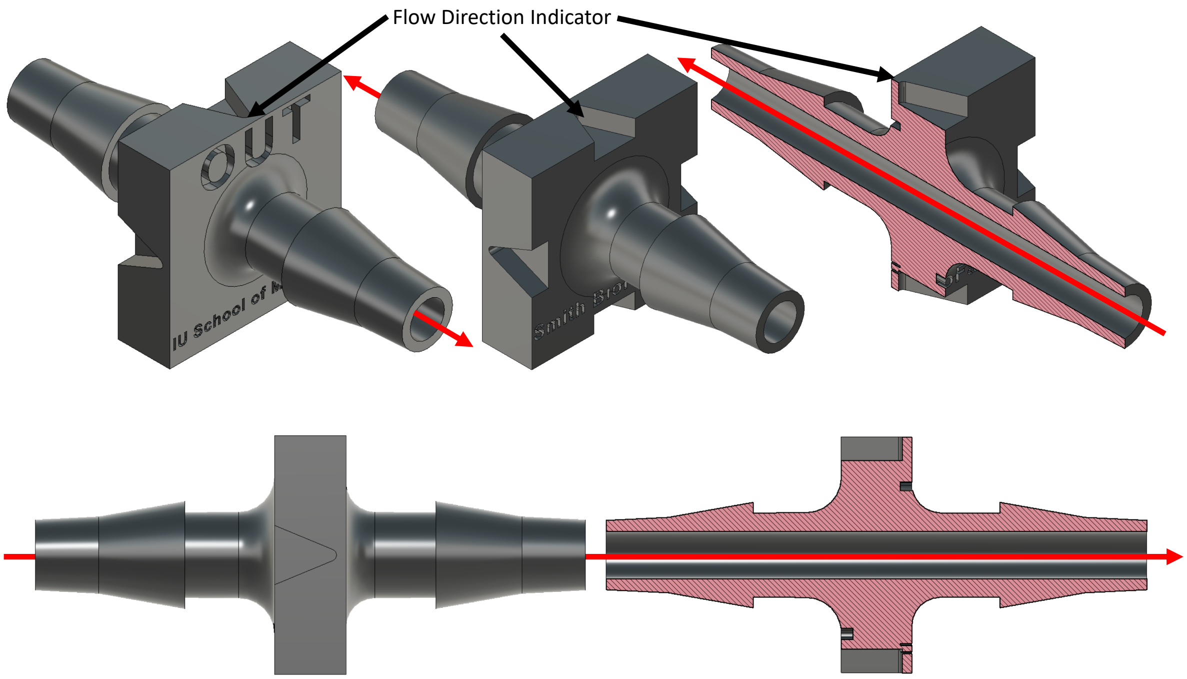

Figure 6. CAD of the Barbed Connector. The Barbed Connector, shown above in 5 different views, has been designed to connect tubing and also help the user identify the flow direction within the flow circuit. Around the perimeter, there are arrowheads pointing to the out direction. There is also text with the word "OUT" on the outward side of the connector. There is no text indicating the "in" direction. Red arrows indicate media flow path. The connector dimensions also fit securely into Connector Holders on the ends of the Tube Clip.

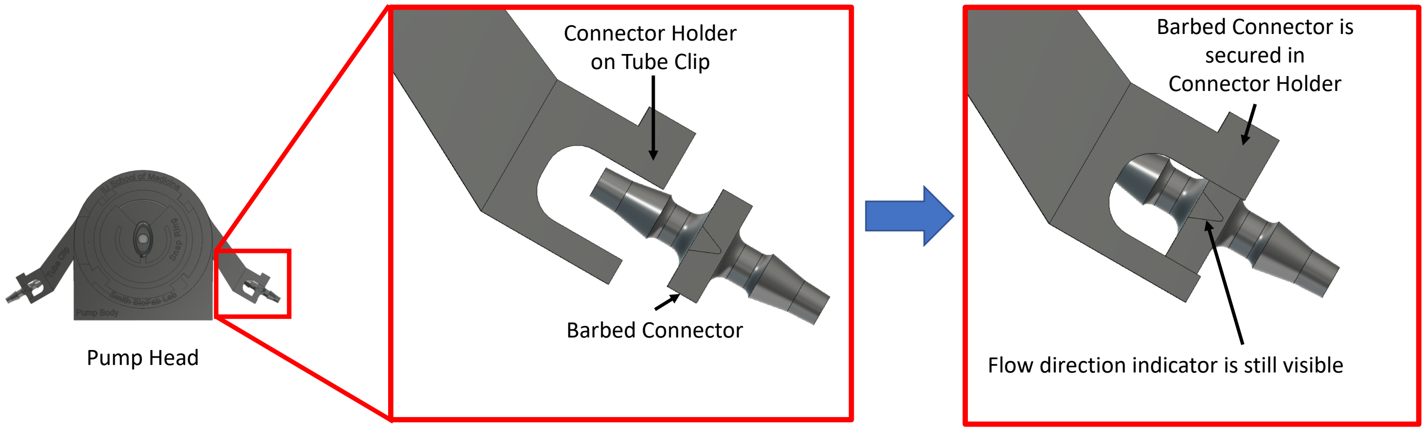

Figure 7. Peristaltic Pump Head Connector Holder holding a Barbed Connector in Place. The Holder is intended to keep the Connector in place during pumping, thus preventing the tubing from being drawn into the Pump Head Rollers. The flow direction indicators on the Barbed Connector is still visible, helping the user identify the pumps flow direction.

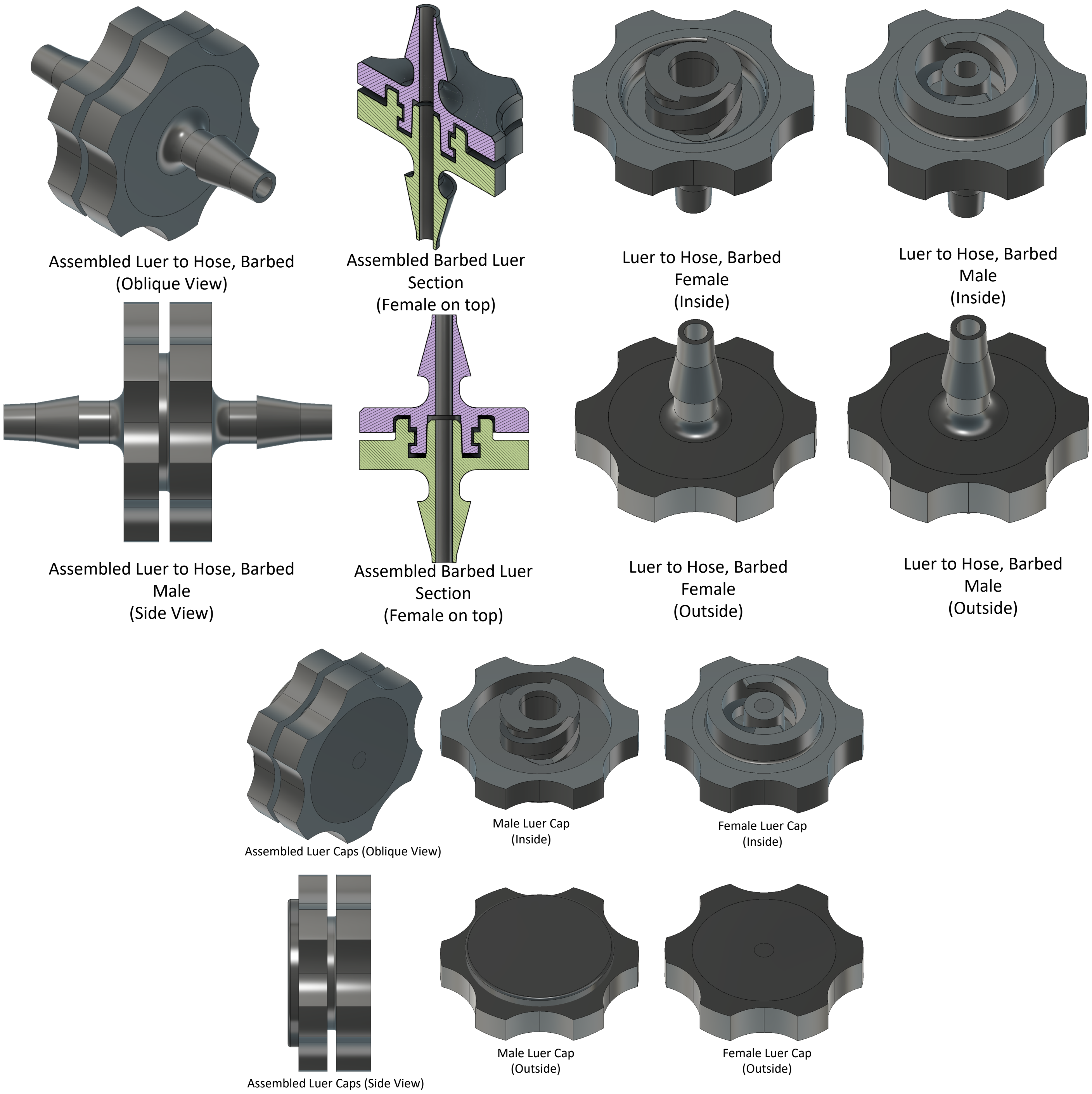

Figure 8. CAD of Barbed Luer Connectors (Male and Female) and Capped Luer Connectors (Male and Female).

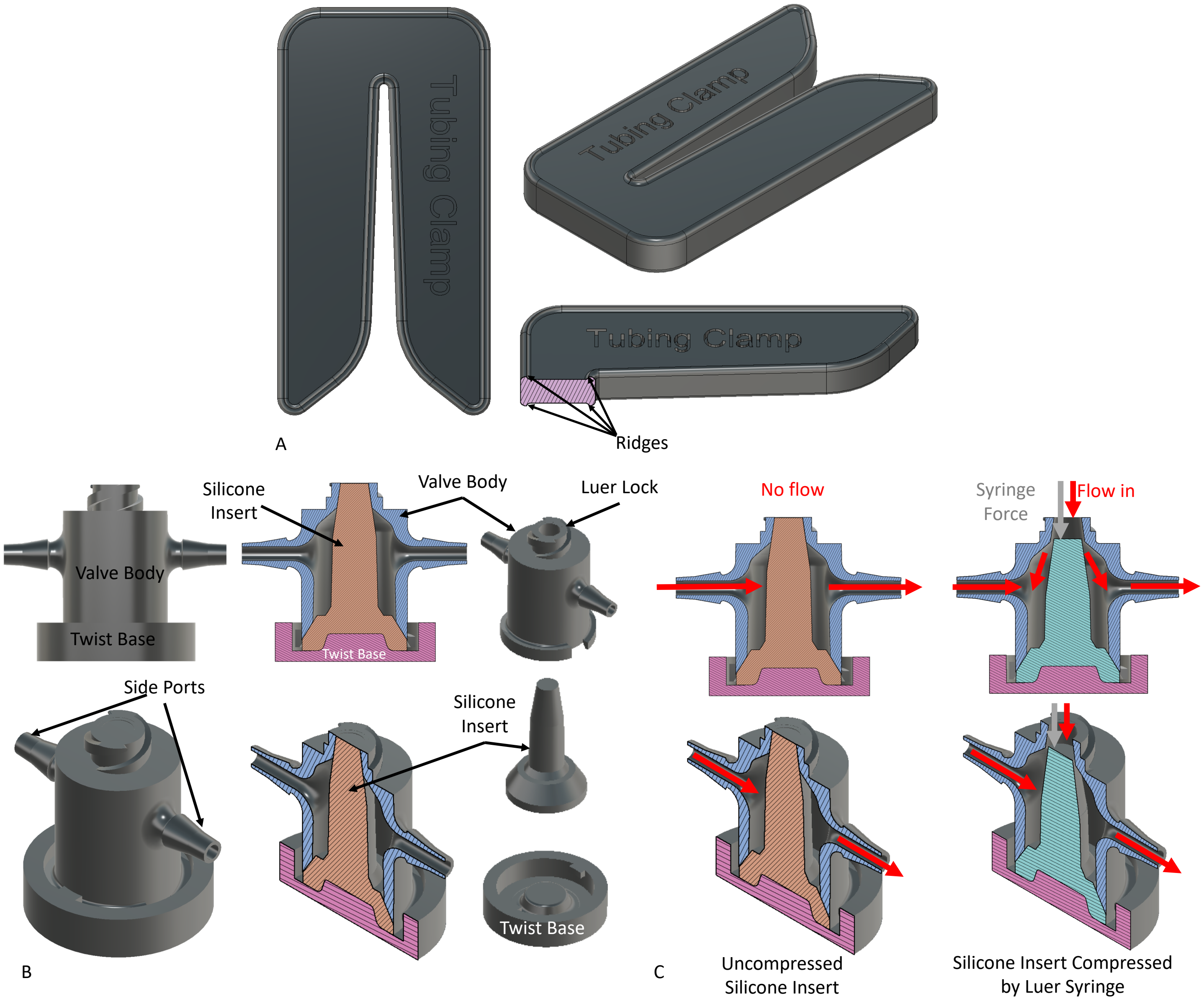

Figure 9. CAD of Tubing Clamps and Needle-Free Valve used to control flow. A. Tubing Clamps are used to occlude media flow through Tubing when the bIUreactor Chamber is being handled or when media is being transferred through the stopcock. The Clamp is designed for use on several tube diameters and they are designed to fit through the opening in the back of the incubator. There are ridges around the Clamp perimeter to facilitate easy grip. B. The 3-part Needle-Free Valve is held together by a Twist Base secured to the Valve Body, thereby containing the Silicone Insert. C. The Valve allows unencumbered flow through the side ports. Flow is stopped when the Tubing is clamped. The top portion of the valve body can receive a Luer Syringe whereby fluid can be introduced into the valve. Once the syringe is removed, the Silicone Insert occludes the Luer opening so flow can again only pass through the side ports. The Tubing Clamps in combination with the Needle-Free Valve serves as a 3-way Stopcock.

Tissue removal

At the end of the study, the platen containing the SSuPerForM tissue was removed by gripping the platen with forceps. The platen was aligned over the central post of the tissue podium and the platen was then gently lowered over the stalk. This action, pushed the mesh, and the tissue it was supporting, up and out of the platen. The tissue was then gently removed from the mesh using a pair of forceps or prepared with the mesh in place (Figure 6).

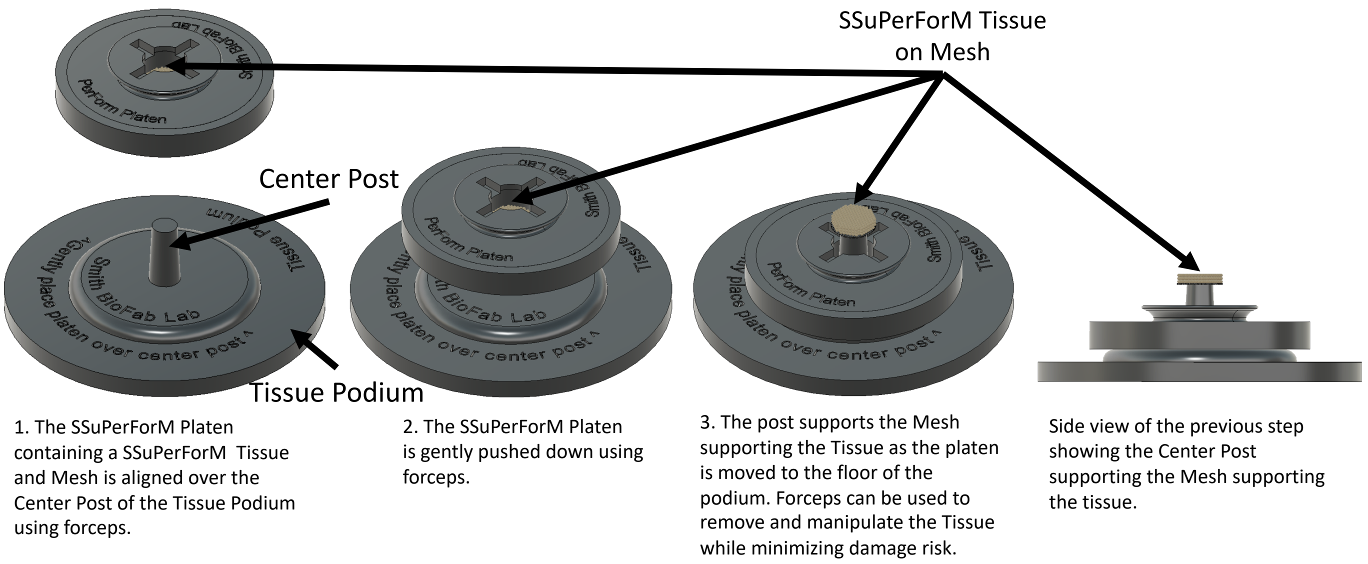

Figure 6. Schematic of the tissue removal procedure using the Tissue Podium.

Tissue Podium

The Tissue Podium (Figure 11), is used to push the SSuPerForM Tissue through the center of the Platen for tissue harvesting.

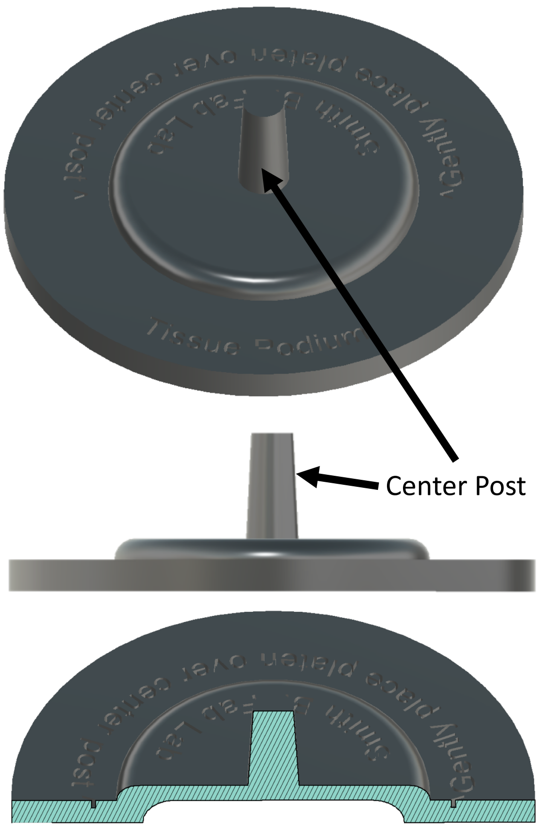

Figure 11. CAD of the Tissue Podium. Tissue Podium is designed for the SSuPerForM Platen to be axially aligned over the center post and pressed downward. This motion will aseptically and gently force the tissue on the mesh out of the SSuPerForM Platen's Central Channel. Once free of the central channel, forceps can be used to lift the tissue by the supporting mesh without risking tissue disruption. A description and accompanying image of tissue removal using the Tissue Podium is described in the results.

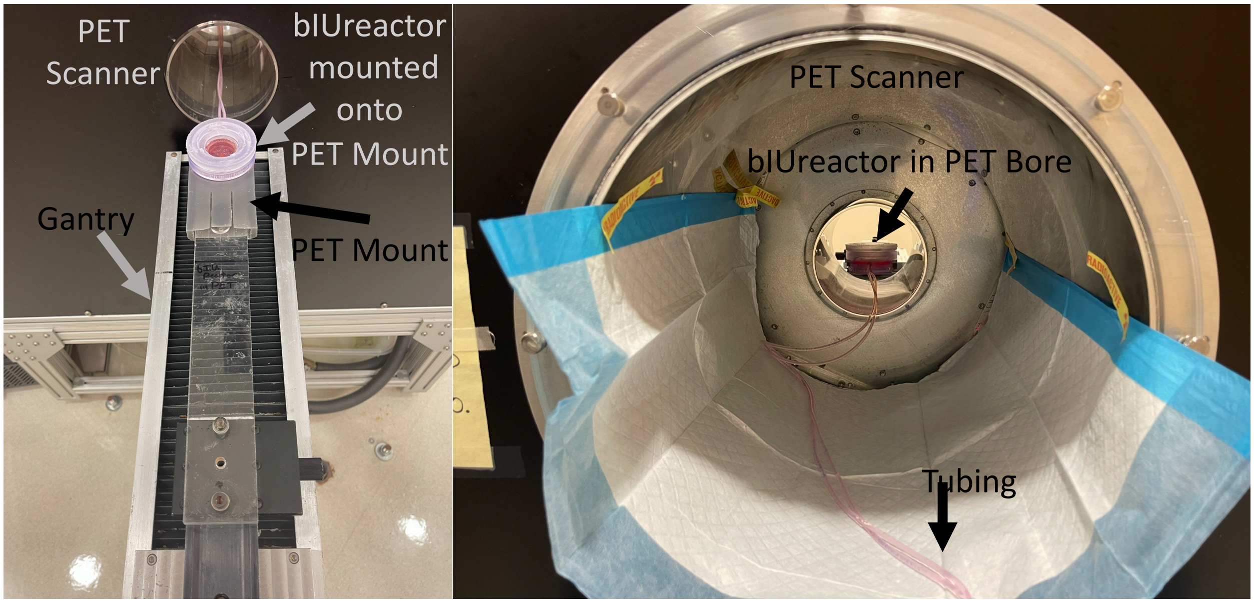

Figure 5. bIUreactor positioning in IndyPET III gantry. The left hand photograph shows the bIUreactor positioned on the IndyPET III bed. The right hand photograph demonstrates positioning of the bIUreactor within the bore of the imaging system.

Arduino Motor Control

Setup and Arduino Code are provided in Supplement 3.

Circuit diagarms for motor control are provided in Supplement 4, User Manual.

Code for Arduino Controller for Cyclic Mechanical Compression Module:

```
/*

Purpose: Stepper control for IU Bioreactor system (Indiana University School of Medicine)

vsoon@iupui.edu: First rev 0.1 (6/4/21)



   Components: TB6600 Stepper Driver, Arduino Uno, Push Terminal Arduino Shield, Stepper Motor (4-wire),

             ON/OFF button for Enable, 2x 10 kOhm Potentiometers (Speed, Range) Control, Power Supplies (5V,12V)


   Connections:


   Arduino Pin             TB6600 Pin              Stepper Motor Color(p/n 17hs19-2004S1      Potentiometers

   -----------             ----------              -------------------                        --------------

     4 -->(ENA-)                ENA- -->(Arduino Pin4)

     5V-->(ENA+)                 ENA+ -->(Arduino +5V)

                           DIR- -->(Arduino GND)

     6 -->(DIR+)                DIR+ -->(Arduino Pin6)

                           PUL- -->(Arduino GND)

     3 -->(PUL+)                PUL+ -->(Arduino Pin3)

                           B-  -->(Red Wire Motor)       Red Wire   -> (B-)

                           B+  -->(Blue Wire Motor)      Blue Wire  -> (B+)

                           A-  -->(Green Wire Motor)     Green Wire -> (A-)

                           A+  -->(Black Wire Motor)     Black Wire -> (A+)

                           GND --> (GND Power Supply)

                           VCC --> (+12V Power Supply)


     A0 --> (Black Wire Pot. for Speed)                                        (Black Wire Pot. for Speed)

     A3 --> (Black Wire Pot. for Range)                                        (Black Wire Pot. for Range)

     GND --> (Yellow Wires of Potentiometers)                                     Yellow Wires of Pot to GND

     +5V --> (Black Wires of Potentiometers)                                     Red Wires of Pot to +5V


 Control ON/OFF Switch(Black to Arduino GND, Red to Arduino Pin 7)

 HOME Switch(Green to Arduino GND, Blue to Arduino Pin 8)


 *HOME Switch is used to detect when stepper has moved to HOME position - it is a micro switch (NORMALLY OFF) which will close to GND when the stepper motor has moved to the HOME position.
(In the code this HOME switch is monitored when the motor is moving BACK).  We need this code to ensure that we will move to a known HOME position

 even if the stepper motor has missed steps in moving.


 WE WANT THE SPEED POT TO CONTROL THE OSCILLATION RATE and THE RANGE(TRAVEL) POT TO CONTROL THE AMOUNT OF TRAVEL

 THEREFORE, FOR A SMALL TRAVEL AND A HIGH HZ RATE THE RESULTING TIME PER COUNTER-INCREMENT IN THE ISR WILL BE SMALL, ETC.


Refs:

  Stepper motor stuff in https://www.pjrc.com/teensy/td_libs_AccelStepper.html

  Timer interrupt stuff from https://learn.adafruit.com/multi-tasking-the-arduino-part-2/timers


Libraries Used:  AccelStepper and TimerOne - to install, from Arduino IDE, select Sketch > Include Library > Manage Libraries... ,

               and do a search for these libraries, and install them.
```

```
*/

#include <AccelStepper.h>
#include <TimerOne.h>

AccelStepper Xaxis(1, 3, 6); // 1 specifies its a stepper motor, pin 3 = step, pin 6 = direction

int StepperSpeedPort = A0;
int StepperRangePort = A3;
int StepperEnablePort = 4;  // use Digital Pin 4 for ENA -- Active High
int StartPort = 7; // overall Enable Switch
int HomePort = 8; // Home Switch
boolean enable_flag_step_update = false;

float valSpeed = 0, valRange = 0;
float CurrentSpeed = 1;
float CurrentRange = 1;
float CurrentRangePosVal = 1;

int curPos;
int cnt = 0, speedCnt;
float tmp = 0.0;

// stepper motor used has 200 steps-per-rotation (motor stepper driver has micro-stepping capability so we will double steps-per-rotation to 2x)
// we will step motor in the interrupt-service-routine (isr_update_step()) running at ISR_INTERVAL millisecond rate
#define DEG_PER_STEP (360.0/(2*200.0)) // using 400 microsteps per revolution using TB6600 1/2 step setting (see TB6600 manual for specific switch settings)
#define MAX_TRAVEL_DEG 1.0*360.0
#define MAX_SPEED_REV 8.0
#define ISR_INTERVAL 0.65

// Interrupt is called once a millisecond,
// we use this to update the stepper motor

void isr_update_step() //SIGNAL(TIMER0_COMPA_vect)
{
  if (enable_flag_step_update == false)
    return;

  if (digitalRead(StartPort) == LOW) {
    if (cnt > speedCnt) {
        Xaxis.run();
        cnt = 0;
    }
    else {
     cnt++;
    }
  }
```

```
    // Change direction at the limits
   if (Xaxis.distanceToGo() == 0)  {

      if (CurrentRange > 0) {
        CurrentRange = -(CurrentRangePosVal);
        Xaxis.move(CurrentRange);
        Xaxis.run();
      }
      else { // CurrentRange < 0 so going backward
        if (digitalRead(HomePort) == LOW) { // must see if we hit home yet if going backward
          CurrentRange = CurrentRangePosVal;
          Xaxis.move(CurrentRange);
          Xaxis.run();
        }
        else // we're not at HOME even though we're going backward and has finished CurrentRange number of steps
        {
            // for this case we must keep going back until we have hit home
            CurrentRange = -5; // move only a small number of steps and check again
            Xaxis.move (CurrentRange);
            Xaxis.run();
        }
      }
   }
}

// read pot settings for Range of travel, and Speed
// given we want to move TRAVEL_DEG at a rate of SPEED_HZ, then travel_deg_per_sec = TRAVEL_DEG/SPEED_HZ
// given ISR_INTERVAL rate (in msec), then total number of intervals in SPEED_HZ is 1000/(SPEED_HZ*ISR_INTERVAL)
// we need to step TRAVEL_DEG/DEG_PER_STEP steps at a rate of SPEED_HZ so this gives us
// (1000/(SPEED_HZ*ISR_INTERVAL)) / (TRAVEL_DEG/DEG_PER_STEP) isr_timeouts/step
//
void read_settings()
{
    valSpeed = float(analogRead(StepperSpeedPort))/1023.0;  // read the input pin
    CurrentSpeed = (MAX_SPEED_REV*valSpeed); // in Hz (1/s)
    if (CurrentSpeed < 0.05)
      CurrentSpeed = 0.05;

    valRange = (analogRead(StepperRangePort))/1023.0;  // read the input pin
    CurrentRange = (MAX_TRAVEL_DEG*(valRange)*(1/DEG_PER_STEP)) + 1; // number of steps per repetition
    if (CurrentRange <= 5)
      CurrentRange = 5.0;

    CurrentRangePosVal = CurrentRange;

    tmp = (1000.0/(CurrentSpeed*ISR_INTERVAL))/(CurrentRange);     //(CurrentRange/CurrentSpeed);    // isr_intervals/step
    speedCnt = round(tmp);
```

```
    cnt = speedCnt;
    Serial.println("Speed Reading: " +String(valSpeed));
    Serial.println("Range Reading: " + String(valRange));
    Serial.println("SpeedCnt: " + String(tmp));
}

void setup() {

 Timer1.initialize(ISR_INTERVAL*1000); //Initialize timer1 with 0.65 millisecond period (ISR_INTERVAL = 0.65, see #define above)
 Timer1.attachInterrupt(isr_update_step);

 // Timer2.initialize();
 // Timer2.attachInterrupt(&isr_update_step);

  Serial.begin(9600);           // set up Serial library at 9600 bps
  Serial.println("IU Bioreactor Stepper Control!");

  Serial.println("Stepper Control Started !");
  Serial.println("=========================");

  Xaxis.setMaxSpeed(600);
  Xaxis.setAcceleration(550);
  curPos = Xaxis.currentPosition();
  Serial.println("Position:");
  Serial.println(curPos);

  read_settings(); // read pot settings for Range of travel, and Speed

//  Xaxis.setSpeed(5000); //CurrentSpeed);///CurrentSpeed);
//  Xaxis.move(CurrentRange);

  pinMode(StepperEnablePort, OUTPUT);
  digitalWrite(StepperEnablePort, HIGH); // disable motor
  pinMode(StartPort, INPUT_PULLUP);
  pinMode(HomePort, INPUT_PULLUP);
}

void loop() {

// if start button is OFF (startPort HIGH) then we do the following:
///    turn off ENA of motor driver - this stops the rotation and allows freewheeling of motor to desired location
///    we update the Speed and Range values
///    we DO NOT  exit but wait until start button is ON (this forces StartPort LOW).
 if (digitalRead(StartPort) == HIGH) {
    cli();
    digitalWrite(StepperEnablePort, LOW); // turn off ENA to disable motor driver
    enable_flag_step_update = false;
```

```
    while (digitalRead(StartPort) == HIGH) {
      delay(1000); // delay 1 second
    }

    digitalWrite(StepperEnablePort, HIGH);  // enable motor driver

    read_settings(); // read pot settings for Range of travel, and Speed

    Xaxis.move(CurrentRange-2);
    Xaxis.setSpeed(CurrentSpeed);

     enable_flag_step_update = true;
    sei();
  }
```

} Code for Arduino Controller for Peristaltic Pump:

```
/*
Purpose: Stepper control for IU Peristaltic system (Indiana University School of Medicine)
vsoon@iupui.edu: First rev 0.1 (6/19/21)
    Components: TB6600 Stepper Driver, Arduino Uno, Push Terminal Arduino Shield, Stepper Motor (4-wire),
                ON/OFF button for Direction Control, 1x 10 kOhm Potentiometers (Speed) Control, Power Supplies (5V,12V)
    Connections:
    Arduino Pin              TB6600 Pin              Stepper Motor Color(p/n 17hs19-2004S1       Potentiometers
    -----------              ----------              -------------------              --------------
        4 -->(ENA-)                ENA- -->(Pin4 Ard)
        5V-->(ENA+)                  ENA+ -->(5V Ard)
                             DIR- -->(PUL-)(GND)
        6 -->(DIR+)                DIR+ -->(Pin6 Ard)
                             PUL- -->(DIR-)(GND)
        3 -->(PUL+)                PUL+ -->(Pin3 Ard)
                             B-  -->(Red Wire Motor)        Red Wire   -> (B-)
                             B+  -->(Blue Wire Motor)       Blue Wire  -> (B+)
                             A-  -->(Green Wire Motor)      Green Wire -> (A-)
                             A+  -->(Black Wire Motor)      Black Wire -> (A+)
                             GND --> (GND Power Supply)
                             VCC --> (+12V Power Supply)
       A0  --> (Black Wire Pot. for Speed)
       A3  --> (Black Wire Pot. for Range)
       GND --> (Yellow Wires of Potentiometers)                                    Yellow Wires of Pot to GND
       +5V --> (Black Wires of Potentiometers)                                    Red Wires of Pot to +5V
      7    --> SWITCH(RED)  - toggle switch to change direction of rotation
      GND  --> SWITCH(BLACK)
TB6600 Stepper Motor Board has the following DIP Switch Settings: SW1(ON), SW2(ON), SW3(OFF), SW4(ON), SW5(OFF), SW6(ON)
refs:
  stepper stuff from https://www.pjrc.com/teensy/td_libs_AccelStepper.html
  Timer interrupt stuff from https://learn.adafruit.com/multi-tasking-the-arduino-part-2/timers
```

```
*/

#include <AccelStepper.h>

AccelStepper Xaxis(1, 3, 6); // pin 3 = step, pin 6 = direction

int StepperSpeedPort = A0;

int StepperRangePort = A3;

int StepperEnablePort = 4;  // use Digital Pin 4 for ENA -- Active High

int StartPort = 7; // overall Enable Switch

int valSpeed = 0, valRange = 0;

int CurrentSpeed = 1, PreviousSpeed = 0;

int CurrentRange = 1, PreviousRange = 0;

#define MIN_SPEED  2

#define MIN_RANGE  2

int curPos, StartPos, EndPos;

int cnt = 0, speedCnt;

int CURRENT_RANGE = 1000;

int DirState = 0, PrevDirState = 0;

// Interrupt is called once a millisecond,

// we use this to update the stepper motor

SIGNAL(TIMER0_COMPA_vect)

{

  unsigned long currentMillis = millis();

   if (cnt > speedCnt) {

        Xaxis.run();

        cnt = 0;

    }

    else {

     cnt++;

    }

}

void setup() {

  Serial.begin(9600);           // set up Serial library at 9600 bps

  Serial.println("IU Peristaltic Pump Stepper Control!");

  Serial.println("Stepper Control Started !");

  Serial.println("========================");

  Xaxis.setMaxSpeed(1000);

  Xaxis.setAcceleration(5000);

  curPos = Xaxis.currentPosition();

  Serial.println("Position:");

  Serial.println(curPos);

  valSpeed = analogRead(StepperSpeedPort);  // read the input pin

  CurrentSpeed = int(100.0*valSpeed/1023.0);

  speedCnt = 1*(100 - CurrentSpeed);

  CurrentRange =  CURRENT_RANGE;

  Xaxis.move(CurrentRange);

  pinMode(StepperEnablePort, OUTPUT);

  pinMode(StartPort, INPUT_PULLUP);

  digitalWrite(StepperEnablePort, HIGH);  // enable motor driver

  // Timer0 is already used for millis() - we'll just interrupt somewhere
```

```
  // in the middle and call the "Compare A" function below
  OCR0A = 0xAF;
  TIMSK0 |= _BV(OCIE0A);
  cnt = 0;

   DirState = PrevDirState = digitalRead(StartPort);
    valSpeed = analogRead(StepperSpeedPort);  // read the input pin
    CurrentSpeed = int(500.0*valSpeed/1023.0);
    speedCnt = 25*(500 - CurrentSpeed);
    CurrentRange = CURRENT_RANGE;
    Xaxis.move(CurrentRange);
    Xaxis.setSpeed(CurrentSpeed);
}
void loop() {
  DirState = digitalRead(StartPort);
  valSpeed = analogRead(StepperSpeedPort);  // read the input pin
  CurrentSpeed = int(25.0*valSpeed/1023.0);
  speedCnt = (25 - CurrentSpeed);
  Xaxis.move(CurrentRange);
  if (DirState != PrevDirState) { // reverse direction of flow
    PrevDirState = DirState;
    CurrentRange = -CurrentRange;
    Xaxis.move(CurrentRange);
    Xaxis.setSpeed(CurrentSpeed);
  }
}
```
