# Supplementary material for: The ‘bIUreactor’: An Open-Source 3D Tissue Research Platform
Source: Ann Biomed Eng. 2024 Mar 26;52(6):1678–92. doi: 10.1007/s10439-024-03481-5 (PMC11082015; doi:10.1007/s10439-024-03481-5)
Supplement: Supplementary file 3 — Supplementary file3 (PDF 231 kb) [file 10439_2024_3481_MOESM3_ESM.pdf]

Arduino Motor Control: Compression and Pump Module Setup and Programming

Smith, Lester J
SMITH BIOFAB LAB

Table of Contents

Chapter 1. Motor Control setup and Programming *2*

Chapter 2. Code for Arduino Controller for Cyclic Mechanical Compression Module: *2*

Chapter 3. Code for Arduino Controller for Peristaltic Pump: *5*

Chapter 1. Motor Control setup and Programming

Arduino is an open source computer platform for the control of electronic devices using Arduino microcontroller boards. The Arduino board is programmed within the Arduino Integrated Development Environment (IDE) running on Linux, PC, or Mac (see https://arduino.cc for download information).

Note that the source code requires 2 additional libraries to be installed within the Arduino IDE. In the Arduino IDE, select Sketch > Include Library >Manage Libraries. Perform a Search for the AccelStepper and TimerOne libraries and then install them. The code for the Compression Module and the Pump are provided below (note: the wiring diagram provided in the User manual performs the same tasks described in the connections below, but with different wire colors.

The code for the Arduino boards for both the Peristaltic Pump and Compressor module are open-source. The mimimum computer requirements for installing and using Arduino IDE 2 are as follows:

Windows: Wint 10 and newer, 64 bits
Linux: 64 bits
Mac OS X: Version 10.14: “Mojave” or newer, 64 bits

The steps for Arduino setup are as follows:

1. Connect a USB cable between your computer and your Arduino Board
2. Download and install the Arduino IDE 2 from https://wiki-content.arduino.cc/en/software
    a. Select the appropriate download for your computer system
3. Open an Arduino Sketch
4. Select the Serial Port corresponding to the Arduino Uno
5. Delete any text in the Sketch
6. Copy the Compressor or Pump code below and paste it into the Sketch
7. Go to Sketch > Include Library > Manage Libraries
8. Search for and install libraries AccelStepper and TimerOne
9. Press the “Upload” button in the upper left corner of the IDE
    a. The code will be compiled and uploaded to the Arduino board
10. Once the code is uploaded, the Arduino is ready for installation into the Motor Control Box Assembly

Chapter 2. Code for Arduino Controller for Cyclic Mechanical Compression Module:

```
/*
Purpose: Stepper control for IU Bioreactor system (Indiana University School of Medicine)
vsoon@iupui.edu: First rev 0.1 (6/4/21)


   Components: TB6600 Stepper Driver, Arduino Uno, Push Terminal Arduino Shield, Stepper Motor (4-wire),
           ON/OFF button for Enable, 2x 10 kOhm Potentiometers (Speed, Range) Control, Power Supplies (5V,12V)

   Connections:

   Arduino Pin              TB6600 Pin              Stepper Motor Color(p/n 17hs19-2004S1      Potentiometers
   -----------            ----------          -------------------               --------------
     4 -->(ENA-)               ENA- -->(Arduino Pin4)
     5V-->(ENA+)                 ENA+ -->(Arduino +5V)
                          DIR- -->(Arduino GND)
     6 -->(DIR+)               DIR+ -->(Arduino Pin6)
                          PUL- -->(Arduino GND)
     3 -->(PUL+)                PUL+ -->(Arduino Pin3)
                          B-  -->(Red Wire Motor)        Red Wire   -> (B-)
                          B+  -->(Blue Wire Motor)       Blue Wire  -> (B+)
                          A-  -->(Green Wire Motor)      Green Wire -> (A-)
                          A+  -->(Black Wire Motor)      Black Wire -> (A+)
                          GND --> (GND Power Supply)
                          VCC --> (+12V Power Supply)

    A0 --> (Black Wire Pot. for Speed)                                        (Black Wire Pot. for Speed)
    A3 --> (Black Wire Pot. for Range)                                        (Black Wire Pot. for Range)
    GND --> (Yellow Wires of Potentiometers)                                      Yellow Wires of Pot to GND
    +5V --> (Black Wires of Potentiometers)                                      Red Wires of Pot to +5V

 Control ON/OFF Switch(Black to Arduino GND, Red to Arduino Pin 7)
```

```
 HOME Switch(Green to Arduino GND, Blue to Arduino Pin 8)

 *HOME Switch is used to detect when stepper has moved to HOME position - it is a micro switch (NORMALLY OFF) which will close to GND when the stepper motor has moved to the HOME position.
(In the code this HOME switch is monitored when the motor is moving BACK).  We need this code to ensure that we will move to a known HOME position
 even if the stepper motor has missed steps in moving.

 WE WANT THE SPEED POT TO CONTROL THE OSCILLATION RATE and THE RANGE(TRAVEL) POT TO CONTROL THE AMOUNT OF TRAVEL
 THEREFORE, FOR A SMALL TRAVEL AND A HIGH HZ RATE THE RESULTING TIME PER COUNTER-INCREMENT IN THE ISR WILL BE SMALL, ETC.

Refs:
  Stepper motor stuff in https://www.pjrc.com/teensy/td_libs_AccelStepper.html
  Timer interrupt stuff from https://learn.adafruit.com/multi-tasking-the-arduino-part-2/timers

Libraries Used:  AccelStepper and TimerOne - to install, from Arduino IDE, select Sketch > Include Library > Manage Libraries... ,
                 and do a search for these libraries, and install them.
*/

#include <AccelStepper.h>
#include <TimerOne.h>

AccelStepper Xaxis(1, 3, 6); // 1 specifies its a stepper motor, pin 3 = step, pin 6 = direction

int StepperSpeedPort = A0;
int StepperRangePort = A3;
int StepperEnablePort = 4;  // use Digital Pin 4 for ENA -- Active High
int StartPort = 7; // overall Enable Switch
int HomePort = 8; // Home Switch
boolean enable_flag_step_update = false;

float valSpeed = 0, valRange = 0;
float CurrentSpeed = 1;
float CurrentRange = 1;
float CurrentRangePosVal = 1;

int curPos;
int cnt = 0, speedCnt;
float tmp = 0.0;

// stepper motor used has 200 steps-per-rotation (motor stepper driver has micro-stepping capability so we will double steps-per-rotation to 2x)
// we will step motor in the interrupt-service-routine (isr_update_step()) running at ISR_INTERVAL millisecond rate
#define DEG_PER_STEP (360.0/(2*200.0)) // using 400 microsteps per revolution using TB6600 1/2 step setting (see TB6600 manual for specific switch settings)
#define MAX_TRAVEL_DEG 1.0*360.0
#define MAX_SPEED_REV 8.0
#define ISR_INTERVAL 0.65

// Interrupt is called once a millisecond,
// we use this to update the stepper motor

void isr_update_step() //SIGNAL(TIMER0_COMPA_vect)
{
  if (enable_flag_step_update == false)
    return;

  if (digitalRead(StartPort) == LOW) {
    if (cnt > speedCnt) {
        Xaxis.run();
        cnt = 0;
    }
    else {
     cnt++;
    }
  }
    // Change direction at the limits
   if (Xaxis.distanceToGo() == 0)  {

      if (CurrentRange > 0) {
        CurrentRange = -(CurrentRangePosVal);
        Xaxis.move(CurrentRange);
        Xaxis.run();
      }
      else { // CurrentRange < 0 so going backward
        if (digitalRead(HomePort) == LOW) { // must see if we hit home yet if going backward
          CurrentRange = CurrentRangePosVal;
          Xaxis.move(CurrentRange);
          Xaxis.run();
        }
        else // we're not at HOME even though we're going backward and has finished CurrentRange number of steps
        {
            // for this case we must keep going back until we have hit home
            CurrentRange = -5; // move only a small number of steps and check again
            Xaxis.move (CurrentRange);
            Xaxis.run();
        }
      }
  }
}

// read pot settings for Range of travel, and Speed
// given we want to move TRAVEL_DEG at a rate of SPEED_HZ, then travel_deg_per_sec = TRAVEL_DEG/SPEED_HZ
// given ISR_INTERVAL rate (in msec), then total number of intervals in SPEED_HZ is 1000/(SPEED_HZ*ISR_INTERVAL)
// we need to step TRAVEL_DEG/DEG_PER_STEP steps at a rate of SPEED_HZ so this gives us
// (1000/(SPEED_HZ*ISR_INTERVAL)) / (TRAVEL_DEG/DEG_PER_STEP) isr_timeouts/step
//
void read_settings()
{
    valSpeed = float(analogRead(StepperSpeedPort))/1023.0;  // read the input pin
    CurrentSpeed = (MAX_SPEED_REV*valSpeed); // in Hz (1/s)
    if (CurrentSpeed < 0.05)
      CurrentSpeed = 0.05;

    valRange = (analogRead(StepperRangePort))/1023.0;  // read the input pin
    CurrentRange = (MAX_TRAVEL_DEG*(valRange)*(1/DEG_PER_STEP)) + 1; // number of steps per repetition
```

```
    if (CurrentRange <= 5)
      CurrentRange = 5.0;

    CurrentRangePosVal = CurrentRange;

    tmp = (1000.0/(CurrentSpeed*ISR_INTERVAL))/(CurrentRange);     //(CurrentRange/CurrentSpeed);    // isr_intervals/step
    speedCnt = round(tmp);
    cnt = speedCnt;
    Serial.println("Speed Reading: " +String(valSpeed));
    Serial.println("Range Reading: " + String(valRange));
    Serial.println("SpeedCnt: " + String(tmp));
}

void setup() {

 Timer1.initialize(ISR_INTERVAL*1000); //Initialize timer1 with 0.65 millisecond period (ISR_INTERVAL = 0.65, see #define above)
 Timer1.attachInterrupt(isr_update_step);

 // Timer2.initialize();
 // Timer2.attachInterrupt(&isr_update_step);

  Serial.begin(9600);          // set up Serial library at 9600 bps
  Serial.println("IU Bioreactor Stepper Control!");

  Serial.println("Stepper Control Started !");
  Serial.println("========================");

  Xaxis.setMaxSpeed(600);
  Xaxis.setAcceleration(550);
  curPos = Xaxis.currentPosition();
  Serial.println("Position:");
  Serial.println(curPos);

  read_settings(); // read pot settings for Range of travel, and Speed

//  Xaxis.setSpeed(5000); //CurrentSpeed);///CurrentSpeed);
//  Xaxis.move(CurrentRange);

  pinMode(StepperEnablePort, OUTPUT);
  digitalWrite(StepperEnablePort, HIGH); // disable motor
  pinMode(StartPort, INPUT_PULLUP);
  pinMode(HomePort, INPUT_PULLUP);
}

void loop() {

// if start button is OFF (startPort HIGH) then we do the following:
///    turn off ENA of motor driver - this stops the rotation and allows freewheeling of motor to desired location
///    we update the Speed and Range values
///    we DO NOT  exit but wait until start button is ON (this forces StartPort LOW).
 if (digitalRead(StartPort) == HIGH) {
    cli();
    digitalWrite(StepperEnablePort, LOW); // turn off ENA to disable motor driver
    enable_flag_step_update = false;

    while (digitalRead(StartPort) == HIGH) {
      delay(1000); // delay 1 second
    }

    digitalWrite(StepperEnablePort, HIGH);  // enable motor driver

    read_settings(); // read pot settings for Range of travel, and Speed

    Xaxis.move(CurrentRange-2);
    Xaxis.setSpeed(CurrentSpeed);

     enable_flag_step_update = true;
    sei();
  }

}
```

Chapter 3. Code for Arduino Controller for Peristaltic Pump:

```
/*
Purpose: Stepper control for IU Peristaltic system (Indiana University School of Medicine)
vsoon@iupui.edu: First rev 0.1 (6/19/21)
   Components: TB6600 Stepper Driver, Arduino Uno, Push Terminal Arduino Shield, Stepper Motor (4-wire),
            ON/OFF button for Direction Control, 1x 10 kOhm Potentiometers (Speed) Control, Power Supplies (5V,12V)
   Connections:
   Arduino Pin              TB6600 Pin            Stepper Motor Color(p/n 17hs19-2004S1      Potentiometers
   -----------              ----------            -------------------                 --------------
      4 -->(ENA-)              ENA- -->(Pin4 Ard)
      5V-->(ENA+)               ENA+ -->(5V Ard)
                            DIR- -->(PUL-)(GND)
      6 -->(DIR+)              DIR+ -->(Pin6 Ard)
                            PUL- -->(DIR-)(GND)
      3 -->(PUL+)               PUL+ -->(Pin3 Ard)
                            B-  -->(Red Wire Motor)        Red Wire   -> (B-)
                            B+  -->(Blue Wire Motor)       Blue Wire  -> (B+)
                            A-  -->(Green Wire Motor)      Green Wire -> (A-)
                            A+  -->(Black Wire Motor)      Black Wire -> (A+)
                            GND --> (GND Power Supply)
                            VCC --> (+12V Power Supply)
     A0  --> (Black Wire Pot. for Speed)
     A3  --> (Black Wire Pot. for Range)
     GND --> (Yellow Wires of Potentiometers)                                     Yellow Wires of Pot to GND
     +5V --> (Black Wires of Potentiometers)                                      Red Wires of Pot to +5V
    7    --> SWITCH(RED)  - toggle switch to change direction of rotation
    GND  --> SWITCH(BLACK)
TB6600 Stepper Motor Board has the following DIP Switch Settings: SW1(ON), SW2(ON), SW3(OFF), SW4(ON), SW5(OFF), SW6(ON)
refs:
  stepper stuff from https://www.pjrc.com/teensy/td_libs_AccelStepper.html
  Timer interrupt stuff from https://learn.adafruit.com/multi-tasking-the-arduino-part-2/timers
*/
#include <AccelStepper.h>
AccelStepper Xaxis(1, 3, 6); // pin 3 = step, pin 6 = direction
int StepperSpeedPort = A0;
int StepperRangePort = A3;
int StepperEnablePort = 4;  // use Digital Pin 4 for ENA -- Active High
int StartPort = 7; // overall Enable Switch
int valSpeed = 0, valRange = 0;
int CurrentSpeed = 1, PreviousSpeed = 0;
int CurrentRange = 1, PreviousRange = 0;
#define MIN_SPEED  2
#define MIN_RANGE  2
int curPos, StartPos, EndPos;
int cnt = 0, speedCnt;
int CURRENT_RANGE = 1000;
int DirState = 0, PrevDirState = 0;
// Interrupt is called once a millisecond,
// we use this to update the stepper motor
SIGNAL(TIMER0_COMPA_vect)
{
  unsigned long currentMillis = millis();
   if (cnt > speedCnt) {
      Xaxis.run();
      cnt = 0;
   }
   else {
    cnt++;
   }
}
void setup() {
  Serial.begin(9600);          // set up Serial library at 9600 bps
  Serial.println("IU Peristaltic Pump Stepper Control!");
  Serial.println("Stepper Control Started !");
  Serial.println("========================");
  Xaxis.setMaxSpeed(1000);
  Xaxis.setAcceleration(5000);
  curPos = Xaxis.currentPosition();
  Serial.println("Position:");
  Serial.println(curPos);
  valSpeed = analogRead(StepperSpeedPort);  // read the input pin
  CurrentSpeed = int(100.0*valSpeed/1023.0);
  speedCnt = 1*(100 - CurrentSpeed);
  CurrentRange =  CURRENT_RANGE;
  Xaxis.move(CurrentRange);
  pinMode(StepperEnablePort, OUTPUT);
  pinMode(StartPort, INPUT_PULLUP);
  digitalWrite(StepperEnablePort, HIGH);  // enable motor driver
  // Timer0 is already used for millis() - we'll just interrupt somewhere
  // in the middle and call the "Compare A" function below
  OCR0A = 0xAF;
  TIMSK0 |= _BV(OCIE0A);
  cnt = 0;

   DirState = PrevDirState = digitalRead(StartPort);
    valSpeed = analogRead(StepperSpeedPort);  // read the input pin
    CurrentSpeed = int(500.0*valSpeed/1023.0);
    speedCnt = 25*(500 - CurrentSpeed);
    CurrentRange = CURRENT_RANGE;
    Xaxis.move(CurrentRange);
    Xaxis.setSpeed(CurrentSpeed);
}
void loop() {
  DirState = digitalRead(StartPort);
  valSpeed = analogRead(StepperSpeedPort);  // read the input pin
  CurrentSpeed = int(25.0*valSpeed/1023.0);
  speedCnt = (25 - CurrentSpeed);
  Xaxis.move(CurrentRange);
  if (DirState != PrevDirState) { // reverse direction of flow
    PrevDirState = DirState;
```

```
    CurrentRange = -CurrentRange;
    Xaxis.move(CurrentRange);
    Xaxis.setSpeed(CurrentSpeed);
  }
}
```
