# Supplementary material for: The ‘bIUreactor’: An Open-Source 3D Tissue Research Platform
Source: Ann Biomed Eng. 2024 Mar 26;52(6):1678–92. doi: 10.1007/s10439-024-03481-5 (PMC11082015; doi:10.1007/s10439-024-03481-5)
Supplement: Supplementary file 4 — Supplementary file4 (PDF 46876 kb) [file 10439_2024_3481_MOESM4_ESM.pdf]

bIUreactor Research Platform

User Manual

Smith, Lester J
SMITH BIOFAB LAB

Table of Contents

Chapter 1. Setting up 3D files *3*

PreForm Setup 3

Chapter 2. 3D printing and post-processing *3*

Chapter 3. Motor Control Programming *0*

Code for Arduino Controller for Cyclic Mechanical Compression Module: 0

Code for Arduino Controller for Peristaltic Pump: 3

Chapter 4. Tubing and Connector Assembly *5*

Making Tubing Set 6

Clamping Tubes 6

Connecting Tubing 7

Luer Caps 8

Chapter 5. Silicone Preparation *9*

Mixed by stirring 9

Mixed with Kwik Gard 9

Chapter 6. Casting Spheroid Makers *10*

Chapter 7. Casting Grommet and Gasket *12*

Chapter 8. Peristaltic Pump Assembly *15*

Peristaltic Pump Exploded Diagram 16

Roller Assembly 18

Pump Head Assembly 19

Motor-Motor Mount Assembly and Mounting Pump Head 21

Adding an additional Pump Head 22

Photo of Fully-Assembled Pump 23

Chapter 9. Needle-Free Valve Casting, Assembly, and Operation *24*

Needle-Free Valve Casting 25

Needle-Free Valve Assembly 26

Needle-Free Valve Mode of Operation 27

Chapter 10. bIUreactor Chamber Assembly *28*

bIUreactor Chamber Exploded Diagram 29

Lid Assembly 30

Chamber Assembly 31

Mating Lid to Chamber 32

Chapter 11. Compressor Assembly *33*

Compressor Exploded Diagram 34

Mating Gearbox to Motor Mount 40
Installing PET Mount 41
Chapter 12. Mating Chamber to Gearbox ***42***
Mating Chamber Assembly to Gearbox Assembly 43
Chapter 13. Motor Control Box Assembly ***45***
Motor Control Box Exploded Diagram 46
Motor Control Box Assembly 47
Motor Control Box Assembly 48
Compressor Circuit Diagram 49
Peristaltic Pump Circuit Diagram 50
Chapter 14. Motor Control and Calibration ***51***
Compressor Calibration 52
Peristaltic Pump Calibration 53
Chapter 15. bIUreactor Research Platform Culture Experiment Preparation Instructions ***54***
bIUreactor Chamber Mode of Operation 55
Making Tubing Set 56
bIureactor Flow Circuit Assembly and Autoclaving 57
bIUreactor Preparation for Spheroid SSuPerForM Seeding 58
SSuPerForM Seeding 59
Spheroid Harvest, Seeding, and SSuPerForm Production 61
Chamber Transfer and Perfusion 62
Chapter 16. Cyclic Mechanical Compression Setup ***63***
Compressor Mode of Operation 64
Gearing 67
Taring End-Effector to top of SSuPerForM Tissue 68
Chapter 17. Media Exchange ***69***
Chapter 18. Visual and Physical Tissue Access ***70***
Chapter 19. Tissue Removal ***70***
Chapter 20. Cleanup ***71***

Chapter 1. Setting up 3D files

The bIUreactor was developed and implemented using the Form 3B+ (Somerville, Massachussetts, USA), which allows printing with their resin BioMed Clear. See Supplement 3 for information about acquiring the printer. PreForm Files are available for download at https://github.iu.edu/smitlej.

PreForm Setup

The parts in the Preform files you downloaded are oriented and laid out for immediate printing without further manipulation. The parts are organized into the Assembly Groups below and they are arranged so that as few prints as possible will result in a complete functional component.

1. Chamber Group
2. Gearbox Group
3. Grommet and Gasket Mold Group
4. Spheroid Maker Group (May require multiple print runs)
5. Peristaltic Pump Head Group
6. Pump Motor Group
7. Motor Control Box Group

If you decide to modify the files or the parts, they should be oriented to allow resin drainage and minimize the number of supports that cannot be easily removed. Any interfaces should be free of supports. The print resin is preselected for BioMed Clear. Use Mini Rafts as the Raft Type.

Chapter 2. 3D printing and post-processing

1. Follow Formlabs guidelines for printing BioMed Clear on the Form 3B+.
2. Remove parts from the build platform using the Formlabs provided scraper.
3. Wash the printed parts in 95% isopropanol, per Formlabs instructions.
    a. This can be done in a Form Wash or in a bath of isopropanol.
    b. Warning: Isopropanol is flammable. Wear proper PPE and take proper precautions necessary to prevent injury or death from mishandling.
4. Dry the parts in air or dry quickly using pressurized air.
5. Cure the part at 60 deg C for 60 minutes in 405 nm UV light.
    a. This can be accomplished using a Form Cure or in a curing cabinet. Be sure to take safety precautions to prevent injury due to UV light exposure.
6. Use the Formlabs provided flush cutters (flat snips) to remove 3D printing supports.
    a. Many supports can be removed by hand. Supports, however, can be sharp. Be careful not to cut yourself.

Chapter 3. Motor Control Programming

The code for the Arduino boards for both the Peristaltic Pump and Compressor module are open-source and can installed as described below:

1. Connect a USB cable between your computer and your Arduino Board
2. Download and install the Arduino IDE from https://wiki-content.arduino.cc/en/software
    a. Select the appropriate download for your computer system
3. Open an Arduino Sketch
4. Select the Serial Port corresponding to the Arduino Uno
5. Delete any text in the Sketch
6. Copy the Compressor or Pump code below and paste it into the Sketch
7. Go to Sketch > Include Library > Manage Libraries
8. Search for and install libraries AccelStepper and TimerOne
9. Press the "Upload" button in the upper left corner of the IDE
    a. The code will be compiled and uploaded to the Arduino board
10. Once the code is uploaded, the Arduino is ready for installation into the Motor Control Box Assembly

Code for Arduino Controller for Cyclic Mechanical Compression Module:

```
/*
Purpose: Stepper control for IU Bioreactor system (Indiana University School of Medicine)
vsoon@iupui.edu: First rev 0.1 (6/4/21)


   Components: TB6600 Stepper Driver, Arduino Uno, Push Terminal Arduino Shield, Stepper Motor (4-wire),
            ON/OFF button for Enable, 2x 10 kOhm Potentiometers (Speed, Range) Control, Power Supplies (5V,12V)

   Connections:

   Arduino Pin            TB6600 Pin            Stepper Motor Color(p/n 17hs19-2004S1      Potentiometers
   -----------            ----------            -------------------                        --------------
      4 -->(ENA-)            ENA- -->(Arduino Pin4)
      5V-->(ENA+)              ENA+ -->(Arduino +5V)
                          DIR- -->(Arduino GND)
      6 -->(DIR+)            DIR+ -->(Arduino Pin6)
                          PUL- -->(Arduino GND)
      3 -->(PUL+)            PUL+ -->(Arduino Pin3)
                          B-  -->(Red Wire Motor)       Red Wire   -> (B-)
                          B+  -->(Blue Wire Motor)      Blue Wire  -> (B+)
                          A-  -->(Green Wire Motor)     Green Wire -> (A-)
                          A+  -->(Black Wire Motor)     Black Wire -> (A+)
                          GND --> (GND Power Supply)
                          VCC --> (+12V Power Supply)

     A0 --> (Black Wire Pot. for Speed)                                     (Black Wire Pot. for Speed)
     A3 --> (Black Wire Pot. for Range)                                     (Black Wire Pot. for Range)
     GND --> (Yellow Wires of Potentiometers)                                  Yellow Wires of Pot to GND
     +5V --> (Black Wires of Potentiometers)                                  Red Wires of Pot to +5V

 Control ON/OFF Switch(Black to Arduino GND, Red to Arduino Pin 7)
 HOME Switch(Green to Arduino GND, Blue to Arduino Pin 8)

 *HOME Switch is used to detect when stepper has moved to HOME position - it is a micro switch (NORMALLY OFF) which will close to GND when the stepper motor has moved to the HOME position.
(In the code this HOME switch is monitored when the motor is moving BACK).  We need this code to ensure that we will move to a known HOME position
 even if the stepper motor has missed steps in moving.

 WE WANT THE SPEED POT TO CONTROL THE OSCILLATION RATE and THE RANGE(TRAVEL) POT TO CONTROL THE AMOUNT OF TRAVEL
 THEREFORE, FOR A SMALL TRAVEL AND A HIGH HZ RATE THE RESULTING TIME PER COUNTER-INCREMENT IN THE ISR WILL BE SMALL, ETC.

Refs:
  Stepper motor stuff in https://www.pjrc.com/teensy/td_libs_AccelStepper.html
  Timer interrupt stuff from https://learn.adafruit.com/multi-tasking-the-arduino-part-2/timers

Libraries Used:  AccelStepper and TimerOne - to install, from Arduino IDE, select Sketch > Include Library > Manage Libraries... ,
              and do a search for these libraries, and install them.
*/

#include <AccelStepper.h>
#include <TimerOne.h>

AccelStepper Xaxis(1, 3, 6); // 1 specifies its a stepper motor, pin 3 = step, pin 6 = direction

int StepperSpeedPort = A0;
int StepperRangePort = A3;
int StepperEnablePort = 4;  // use Digital Pin 4 for ENA -- Active High
int StartPort = 7; // overall Enable Switch
int HomePort = 8; // Home Switch
boolean enable_flag_step_update = false;

float valSpeed = 0, valRange = 0;
float CurrentSpeed = 1;
float CurrentRange = 1;
float CurrentRangePosVal = 1;
```

```
int curPos;
int cnt = 0, speedCnt;
float tmp = 0.0;

// stepper motor used has 200 steps-per-rotation (motor stepper driver has micro-stepping capability so we will double steps-per-rotation to 2x)
// we will step motor in the interrupt-service-routine (isr_update_step()) running at ISR_INTERVAL millisecond rate
#define DEG_PER_STEP (360.0/(2*200.0)) // using 400 microsteps per revolution using TB6600 1/2 step setting (see TB6600 manual for specific switch settings)
#define MAX_TRAVEL_DEG 1.0*360.0
#define MAX_SPEED_REV 8.0
#define ISR_INTERVAL 0.65

// Interrupt is called once a millisecond,
// we use this to update the stepper motor

void isr_update_step() //SIGNAL(TIMER0_COMPA_vect)
{
  if (enable_flag_step_update == false)
    return;

  if (digitalRead(StartPort) == LOW) {
    if (cnt > speedCnt) {
        Xaxis.run();
        cnt = 0;
    }
    else {
     cnt++;
    }
  }
    // Change direction at the limits
   if (Xaxis.distanceToGo() == 0)  {

      if (CurrentRange > 0) {
        CurrentRange = -(CurrentRangePosVal);
        Xaxis.move(CurrentRange);
        Xaxis.run();
      }
      else { // CurrentRange < 0 so going backward
        if (digitalRead(HomePort) == LOW) { // must see if we hit home yet if going backward
          CurrentRange = CurrentRangePosVal;
          Xaxis.move(CurrentRange);
          Xaxis.run();
        }
        else // we're not at HOME even though we're going backward and has finished CurrentRange number of steps
        {
            // for this case we must keep going back until we have hit home
            CurrentRange = -5; // move only a small number of steps and check again
            Xaxis.move (CurrentRange);
            Xaxis.run();
        }
      }
  }
}

// read pot settings for Range of travel, and Speed
// given we want to move TRAVEL_DEG at a rate of SPEED_HZ, then travel_deg_per_sec = TRAVEL_DEG/SPEED_HZ
// given ISR_INTERVAL rate (in msec), then total number of intervals in SPEED_HZ is 1000/(SPEED_HZ*ISR_INTERVAL)
// we need to step TRAVEL_DEG/DEG_PER_STEP steps at a rate of SPEED_HZ so this gives us
// (1000/(SPEED_HZ*ISR_INTERVAL)) / (TRAVEL_DEG/DEG_PER_STEP) isr_timeouts/step
//
void read_settings()
{
    valSpeed = float(analogRead(StepperSpeedPort))/1023.0;  // read the input pin
    CurrentSpeed = (MAX_SPEED_REV*valSpeed); // in Hz (1/s)
    if (CurrentSpeed < 0.05)
      CurrentSpeed = 0.05;

    valRange = (analogRead(StepperRangePort))/1023.0;  // read the input pin
    CurrentRange = (MAX_TRAVEL_DEG*(valRange)*(1/DEG_PER_STEP)) + 1; // number of steps per repetition
    if (CurrentRange <= 5)
      CurrentRange = 5.0;

    CurrentRangePosVal = CurrentRange;

    tmp = (1000.0/(CurrentSpeed*ISR_INTERVAL))/(CurrentRange);     //(CurrentRange/CurrentSpeed);    // isr_intervals/step
    speedCnt = round(tmp);
    cnt = speedCnt;
    Serial.println("Speed Reading: " +String(valSpeed));
    Serial.println("Range Reading: " + String(valRange));
    Serial.println("SpeedCnt: " + String(tmp));
}

void setup() {

 Timer1.initialize(ISR_INTERVAL*1000); //Initialize timer1 with 0.65 millisecond period (ISR_INTERVAL = 0.65, see #define above)
 Timer1.attachInterrupt(isr_update_step);

 // Timer2.initialize();
 // Timer2.attachInterrupt(&isr_update_step);

  Serial.begin(9600);          // set up Serial library at 9600 bps
  Serial.println("IU Bioreactor Stepper Control!");

  Serial.println("Stepper Control Started !");
  Serial.println("========================");

  Xaxis.setMaxSpeed(600);
  Xaxis.setAcceleration(550);
  curPos = Xaxis.currentPosition();
  Serial.println("Position:");
  Serial.println(curPos);
```

```
  read_settings(); // read pot settings for Range of travel, and Speed

//  Xaxis.setSpeed(5000); //CurrentSpeed);///CurrentSpeed);
//  Xaxis.move(CurrentRange);

  pinMode(StepperEnablePort, OUTPUT);
  digitalWrite(StepperEnablePort, HIGH); // disable motor
  pinMode(StartPort, INPUT_PULLUP);
  pinMode(HomePort, INPUT_PULLUP);
}

void loop() {

// if start button is OFF (startPort HIGH) then we do the following:
///    turn off ENA of motor driver - this stops the rotation and allows freewheeling of motor to desired location
///    we update the Speed and Range values
///    we DO NOT  exit but wait until start button is ON (this forces StartPort LOW).
 if (digitalRead(StartPort) == HIGH) {
    cli();
    digitalWrite(StepperEnablePort, LOW); // turn off ENA to disable motor driver
    enable_flag_step_update = false;

    while (digitalRead(StartPort) == HIGH) {
      delay(1000); // delay 1 second
    }

    digitalWrite(StepperEnablePort, HIGH);  // enable motor driver

    read_settings(); // read pot settings for Range of travel, and Speed

    Xaxis.move(CurrentRange-2);
    Xaxis.setSpeed(CurrentSpeed);

     enable_flag_step_update = true;
    sei();
  }

}
```

Code for Arduino Controller for Peristaltic Pump:

```
/*
Purpose: Stepper control for IU Peristaltic system (Indiana University School of Medicine)
vsoon@iupui.edu: First rev 0.1 (6/19/21)
   Components: TB6600 Stepper Driver, Arduino Uno, Push Terminal Arduino Shield, Stepper Motor (4-wire),
            ON/OFF button for Direction Control, 1x 10 kOhm Potentiometers (Speed) Control, Power Supplies (5V,12V)
   Connections:
   Arduino Pin              TB6600 Pin              Stepper Motor Color(p/n 17hs19-2004S1       Potentiometers
   -----------              ----------              -------------------                          --------------
      4 -->(ENA-)               ENA- -->(Pin4 Ard)
      5V-->(ENA+)                 ENA+ -->(5V Ard)
                            DIR- -->(PUL-)(GND)
      6 -->(DIR+)               DIR+ -->(Pin6 Ard)
                            PUL- -->(DIR-)(GND)
      3 -->(PUL+)                PUL+ -->(Pin3 Ard)
                            B-  -->(Red Wire Motor)         Red Wire   -> (B-)
                            B+  -->(Blue Wire Motor)        Blue Wire  -> (B+)
                            A-  -->(Green Wire Motor)       Green Wire -> (A-)
                            A+  -->(Black Wire Motor)       Black Wire -> (A+)
                            GND --> (GND Power Supply)
                            VCC --> (+12V Power Supply)
     A0  --> (Black Wire Pot. for Speed)
     A3  --> (Black Wire Pot. for Range)
     GND --> (Yellow Wires of Potentiometers)                                      Yellow Wires of Pot to GND
     +5V --> (Black Wires of Potentiometers)                                      Red Wires of Pot to +5V
    7    --> SWITCH(RED)  - toggle switch to change direction of rotation
    GND  --> SWITCH(BLACK)
TB6600 Stepper Motor Board has the following DIP Switch Settings: SW1(ON), SW2(ON), SW3(OFF), SW4(ON), SW5(OFF), SW6(ON)
refs:
  stepper stuff from https://www.pjrc.com/teensy/td_libs_AccelStepper.html
  Timer interrupt stuff from https://learn.adafruit.com/multi-tasking-the-arduino-part-2/timers
*/
#include <AccelStepper.h>
AccelStepper Xaxis(1, 3, 6); // pin 3 = step, pin 6 = direction
int StepperSpeedPort = A0;
int StepperRangePort = A3;
int StepperEnablePort = 4;  // use Digital Pin 4 for ENA -- Active High
int StartPort = 7; // overall Enable Switch
int valSpeed = 0, valRange = 0;
int CurrentSpeed = 1, PreviousSpeed = 0;
int CurrentRange = 1, PreviousRange = 0;
#define MIN_SPEED  2
#define MIN_RANGE  2
int curPos, StartPos, EndPos;
int cnt = 0, speedCnt;
int CURRENT_RANGE = 1000;
int DirState = 0, PrevDirState = 0;
// Interrupt is called once a millisecond,
// we use this to update the stepper motor
SIGNAL(TIMER0_COMPA_vect)
{
  unsigned long currentMillis = millis();
   if (cnt > speedCnt) {
       Xaxis.run();
       cnt = 0;
   }
   else {
    cnt++;
   }
}
void setup() {
  Serial.begin(9600);          // set up Serial library at 9600 bps
  Serial.println("IU Peristaltic Pump Stepper Control!");
  Serial.println("Stepper Control Started !");
  Serial.println("========================");
  Xaxis.setMaxSpeed(1000);
  Xaxis.setAcceleration(5000);
  curPos = Xaxis.currentPosition();
  Serial.println("Position:");
  Serial.println(curPos);
  valSpeed = analogRead(StepperSpeedPort);  // read the input pin
  CurrentSpeed = int(100.0*valSpeed/1023.0);
  speedCnt = 1*(100 - CurrentSpeed);
  CurrentRange =  CURRENT_RANGE;
  Xaxis.move(CurrentRange);
  pinMode(StepperEnablePort, OUTPUT);
  pinMode(StartPort, INPUT_PULLUP);
  digitalWrite(StepperEnablePort, HIGH);  // enable motor driver
  // Timer0 is already used for millis() - we'll just interrupt somewhere
  // in the middle and call the "Compare A" function below
  OCR0A = 0xAF;
  TIMSK0 |= _BV(OCIE0A);
  cnt = 0;

   DirState = PrevDirState = digitalRead(StartPort);
    valSpeed = analogRead(StepperSpeedPort);  // read the input pin
    CurrentSpeed = int(500.0*valSpeed/1023.0);
    speedCnt = 25*(500 - CurrentSpeed);
    CurrentRange = CURRENT_RANGE;
    Xaxis.move(CurrentRange);
    Xaxis.setSpeed(CurrentSpeed);
}
void loop() {
  DirState = digitalRead(StartPort);
  valSpeed = analogRead(StepperSpeedPort);  // read the input pin
  CurrentSpeed = int(25.0*valSpeed/1023.0);
  speedCnt = (25 - CurrentSpeed);
  Xaxis.move(CurrentRange);
  if (DirState != PrevDirState) { // reverse direction of flow
    PrevDirState = DirState;
```

```
    CurrentRange = -CurrentRange;
    Xaxis.move(CurrentRange);
    Xaxis.setSpeed(CurrentSpeed);
  }
}
```

Chapter 4. Tubing and Connector Assembly

You will need:

1. Not Printed
    1. Tubing (Tygon 3350)
    2. Scissors
2. Printed (BioMed Clear V1 Resin)
    1. Barbed Connector
    2. Male Luer Connector
    3. Female Luer Connector
    4. Male Luer Cap
    5. Female Luer Cap

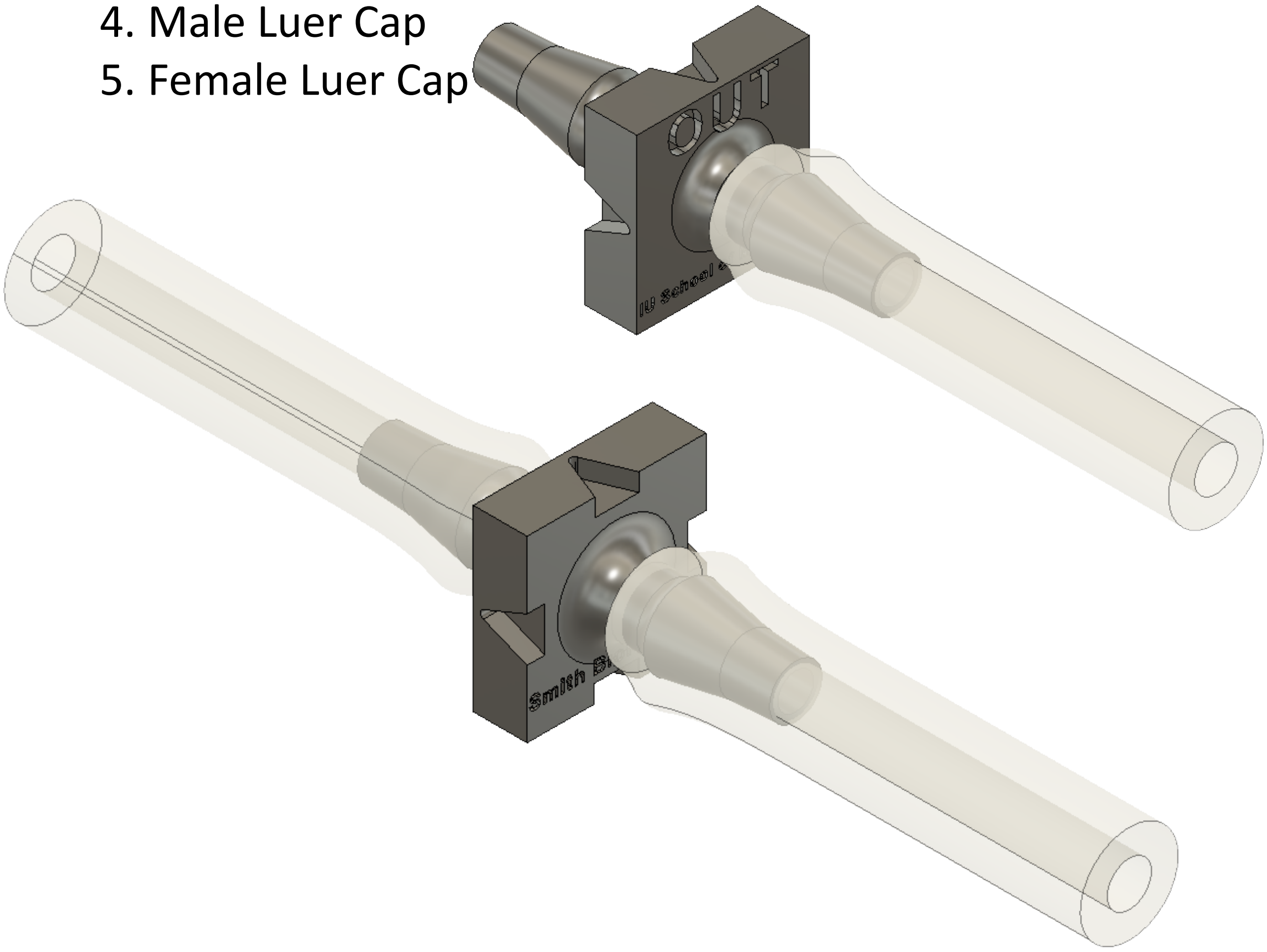

Making Tubing Set

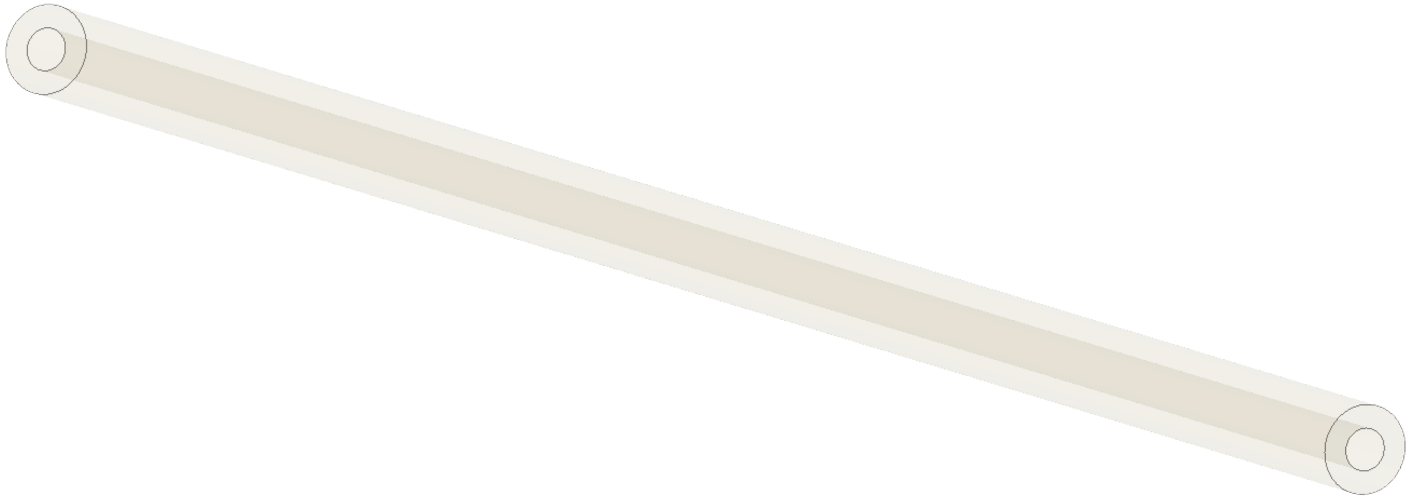

Figure 4-1. Cut Tubing to length per instructions.

Clamping Tubes

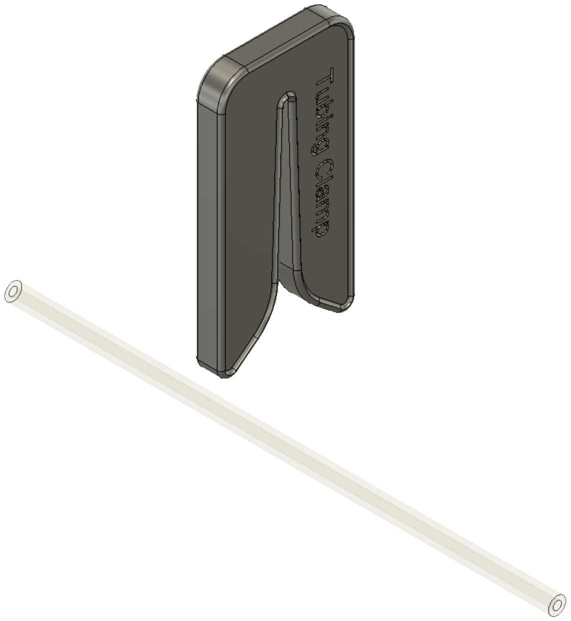

Figure 4-2. To block flow, slide the Clamp over the Tubing

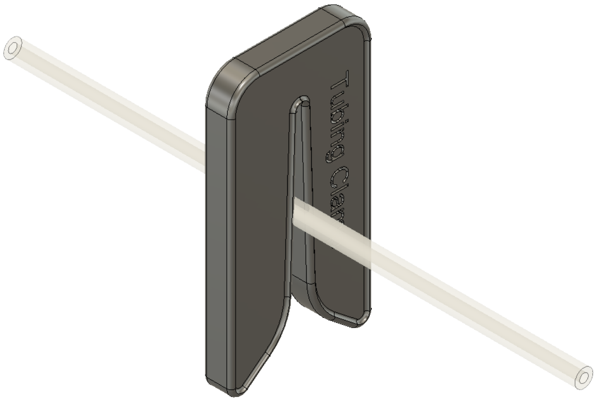

Figure 4-3. To allow flow, slide the Clamp off of the Tubing

Connecting Tubing

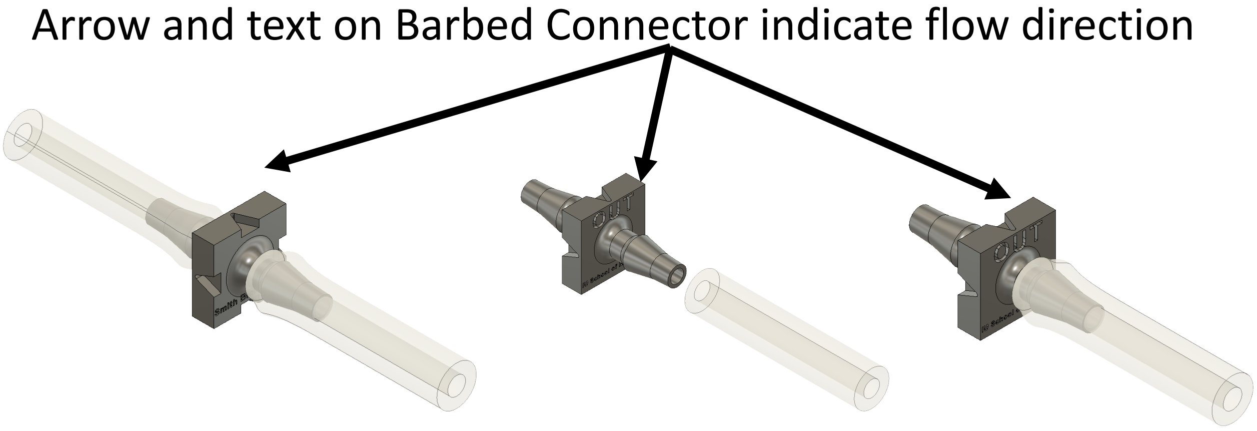

Figure 4-4. For the Barbed Connector, ensure you are mindful of the indicated flow direction

Figure 4-5. Align Tubing with barbed nipple and Push Tubing onto the barbed end.

Figure 4-6. Ensure the Tubing is pushed over the crest of the Barb.

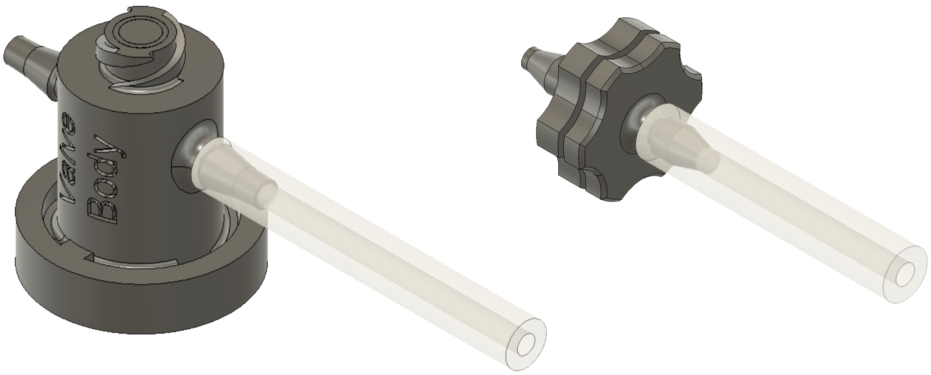

Figure 4-7. The same can be done for Needle-Free Valves and Luer Connectors, respectively.

Luer Connectors

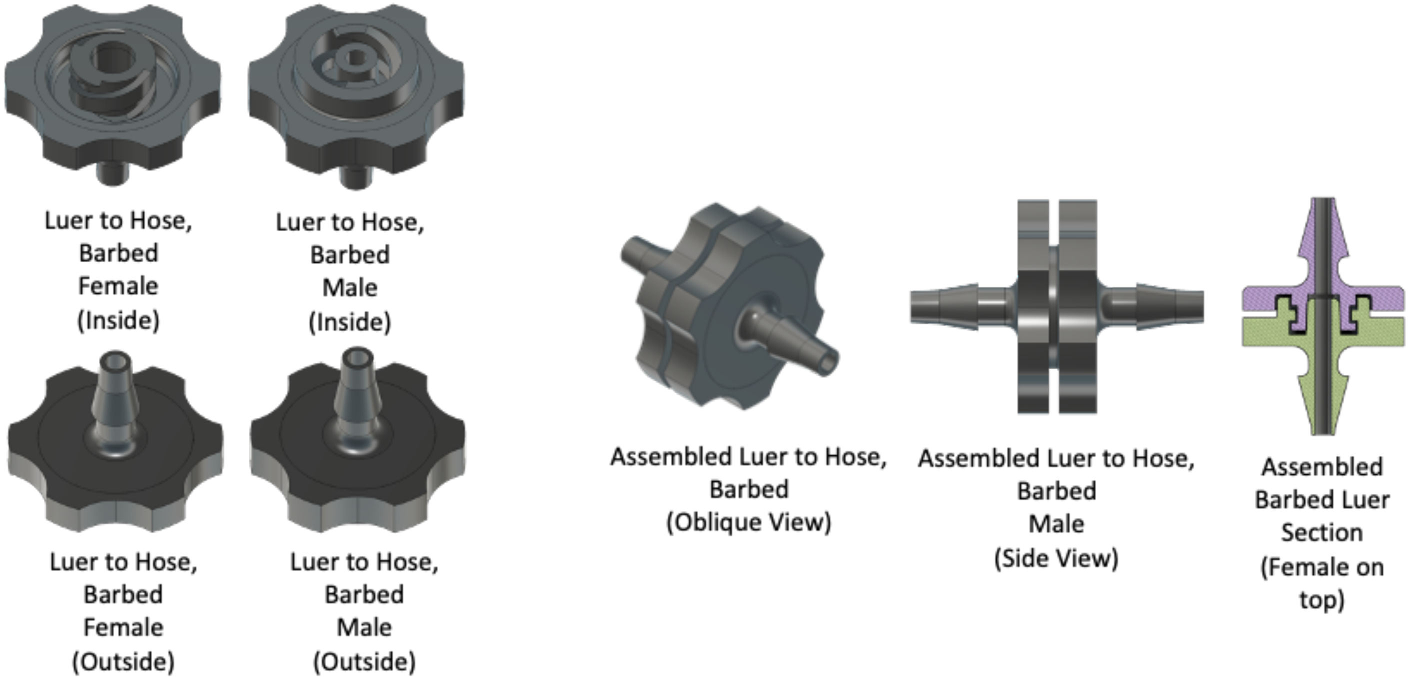

Figure 4-8. Male and Female Barbed Luer Connectors.

Luer Caps

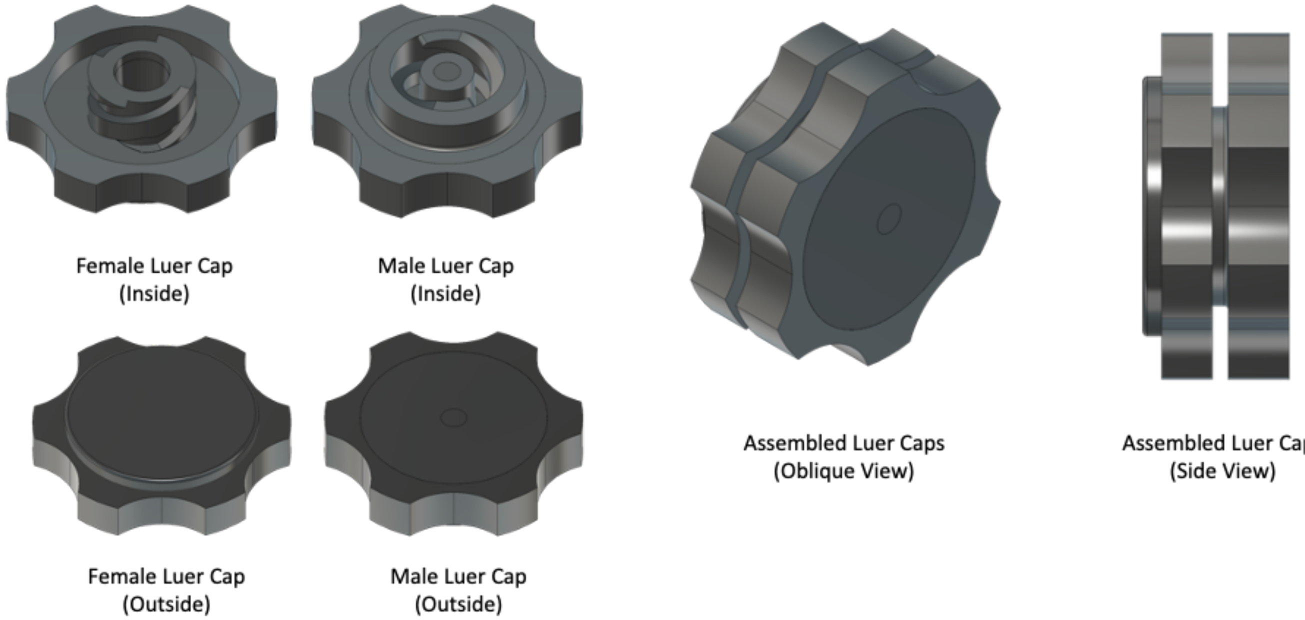

Figure 4-9. Male and Female Barbed Luer Caps.

Chapter 5. Silicone Preparation

You will need:

1. Not Printed
    1. Silicone (Elastosil, Sylgard 184, or other biocompatible, optically-clear, and autoclavable silicone)
    2. Syringe or Kwik Gard Kit
    3. Spatula
    4. Centrifuge

Mixed by stirring

This method can be used for any compatible silicone type.

1. Place Luer onto Syringe.
2. Remove Plunger from Syringe.
3. Pour silicone components into the Syringe Tube at the correct ratios, making up a volume of 40 ml.
4. Mix silicone thoroughly with a spatula.
    1. The silicone will become filled with air bubbles.
5. Place Plunger onto Syringe Tube and invert so the trapped air bubble floats to the capped end of the Syringe.
6. Centrifuge for 5 minutes at 500xg.
7. Remove Cap.
8. Slowly and firmly push air out of the Syringe.
9. Proceed to Chapter 6.

Mixed with Kwik Gard

1. Place Luer onto Syringe.
2. Remove Plunger from Syringe.
3. Secure Dispensing Tip to cartridge.
4. Dispense silicone into the Syringe Tube or directly into the Mold.
    1. The Dispenser Tip will mix the components.
    2. This process should not introduce bubbles but if it does, follow the Mixed by Stirring directions.
5. Follow manufacturer instructions for curing.
6. Proceed to Chapter 6.

Chapter 6. Casting Spheroid Makers

You will need:

1. Not Printed
    1. Freshly mixed Silicone (Elastosil or Sylgard 184, see previous chapter)
    2. Syringe
    3. Mold release
    4. Spatula or stirring bar
    5. Capped Test Tubes
    6. Centrifuge
    7. Hydrogen peroxide
2. Printed (BioMed Clear V1 Resin)
    1. Spheroid Maker Lid Mold
    2. Spheroid Maker Plate and Mold Base (2x)
    3. 50-60 ml Syringe
    4. Female Luer Cap
3. Optional
    1. Curing oven

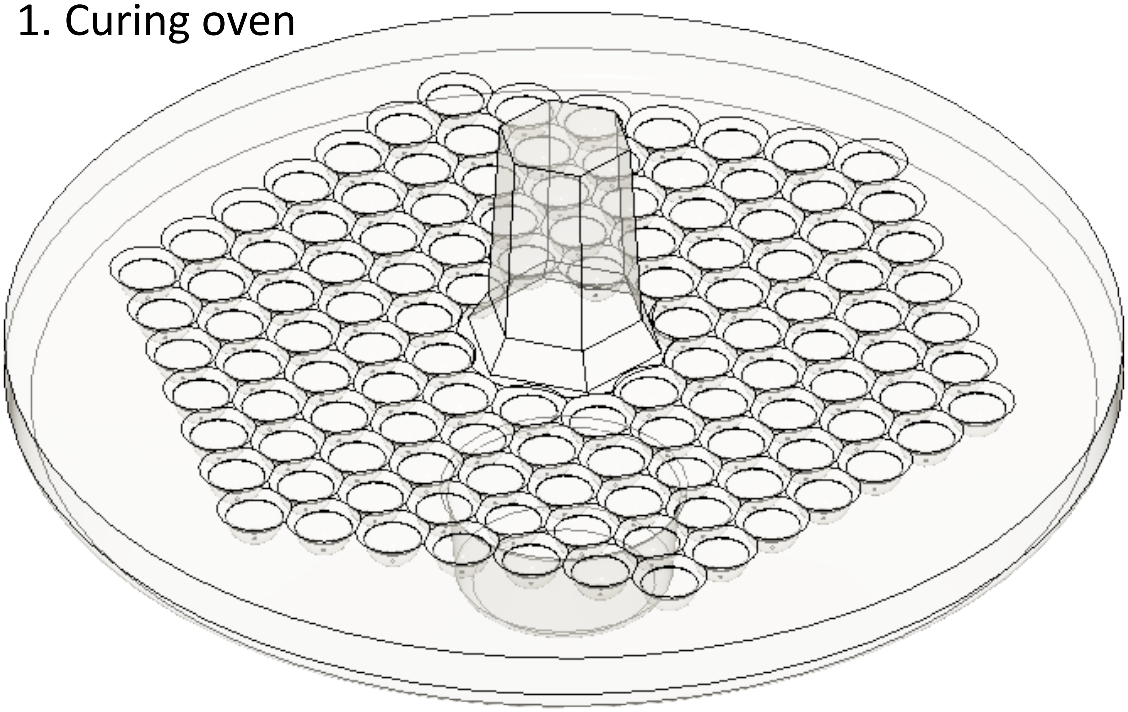

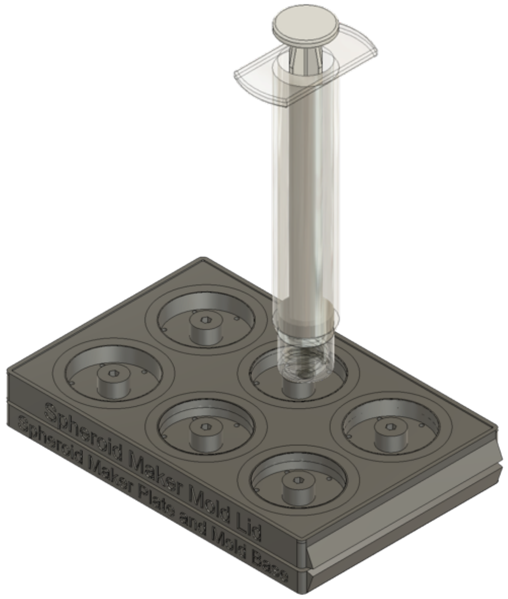

Figure 6-1. Pump silicone from syringe into the opening over each well. Centrifuge at 500xg for 5 minutes, if desired. Cure per silicone provider's instructions.

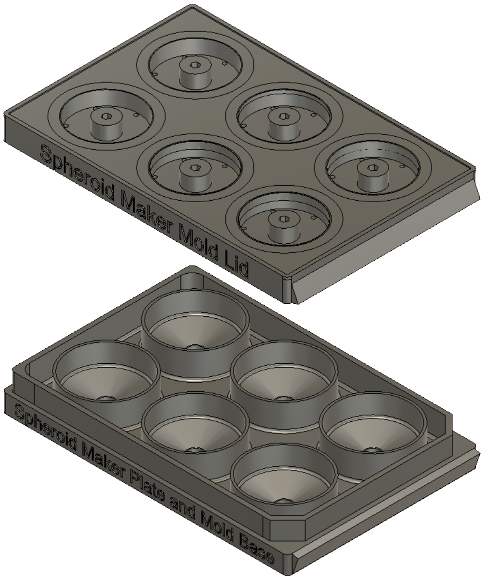

Figure 6-2. Spray mold release into each well of the Spheroid Maker Plate and onto the entire underside of the Spheroid Maker Lid. Place Spheroid Maker Lid Mold onto Spheroid Maker Plate.

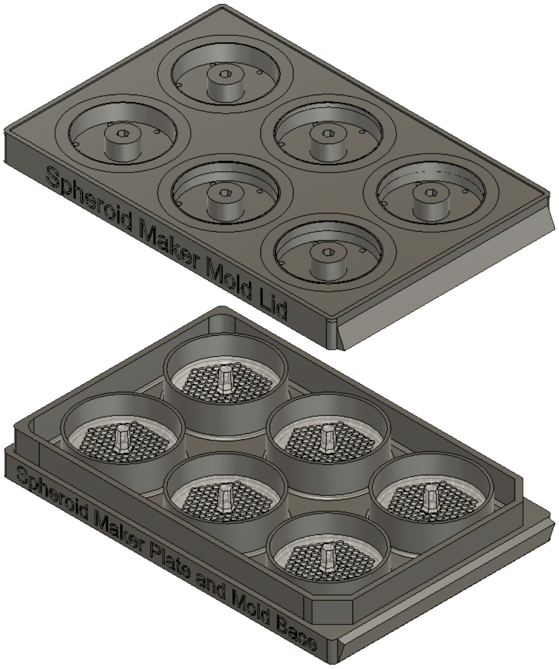

Figure 6-3. Slowly and carefully remove Lid from Plate.

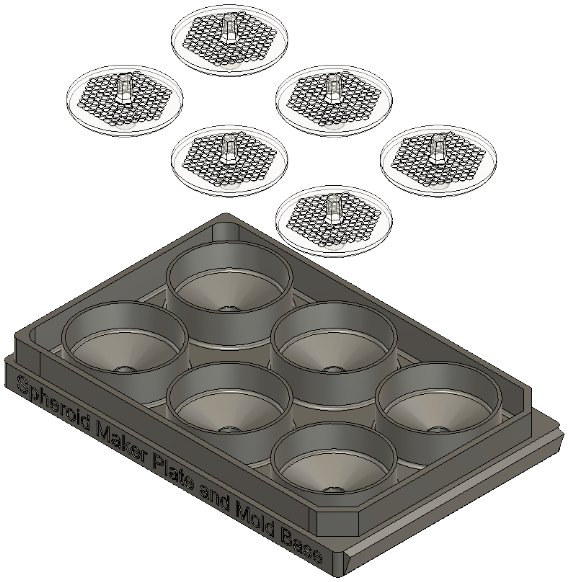

Figure 6-4. Slowly remove spheroid makers from Molds using forceps. Clean with soap and water, then with 3% hydrogen peroxide, and then with fresh water.

Chapter 7. Casting Grommet and Gasket

You will need:

1. Not Printed
    1. Silicone (Elastosil or Sylgard 184)
    2. Mold release
2. Printed
    1. Grommet Mold
    2. Gasket Mold
    3. End-Effector Insert
    4. Optional
    5. Centrifuge
    6. Capped test tubes
    7. Curing oven
    8. Syringe

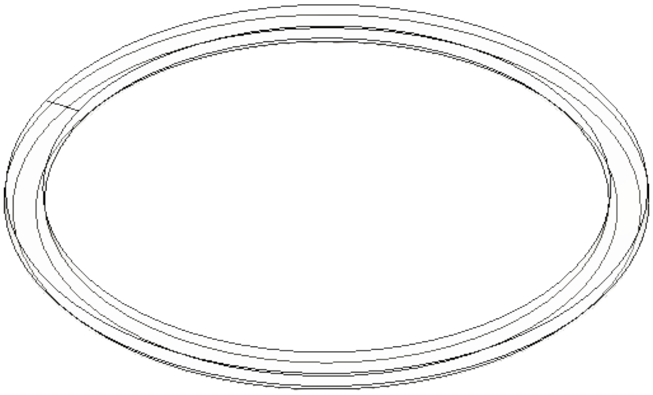

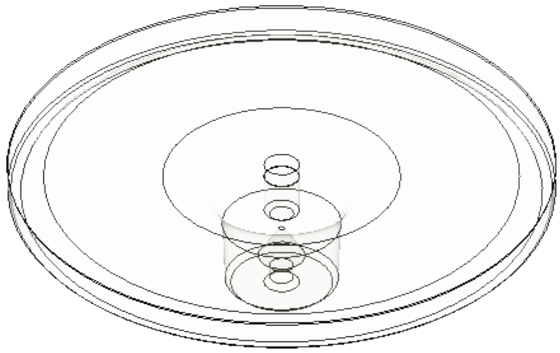

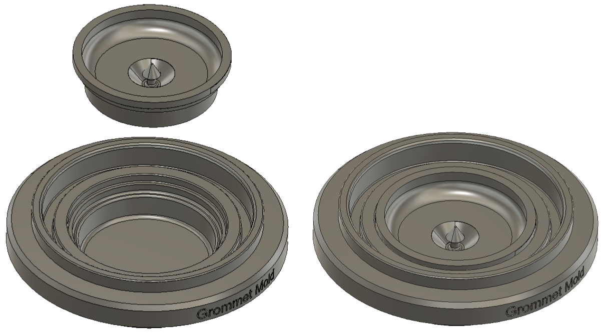

Figure 7-1. Place the End-effector Insert into the Grommet Mold (if making a Grommet for cyclic compression). Spray mold release onto mold.

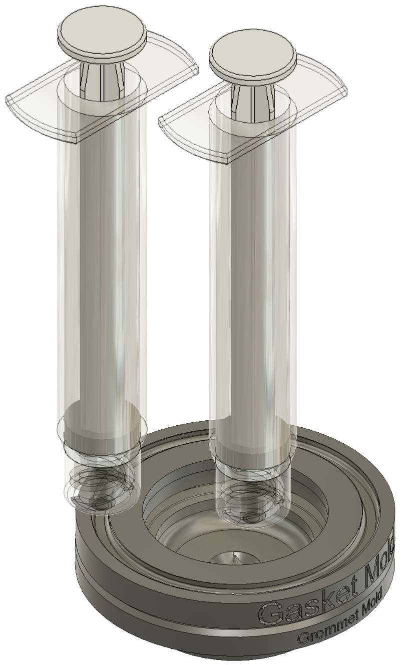

Figure 7-2. Fill molds with bubble-free silicone using a syringe. See Chapter on Casting Spheroid Makers. Cure silicone per Manufacturer instructions.

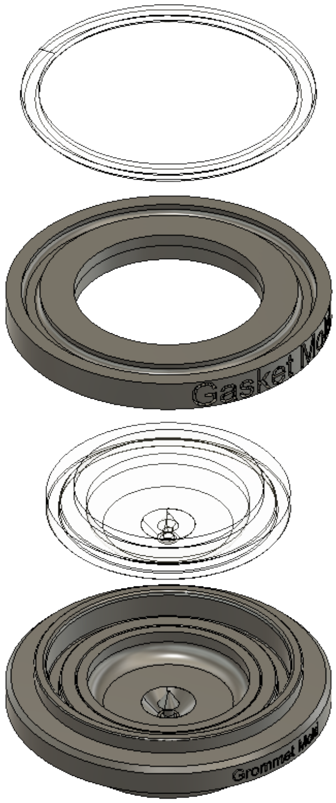

Figure 7-3. Demold Grommet and Gasket.

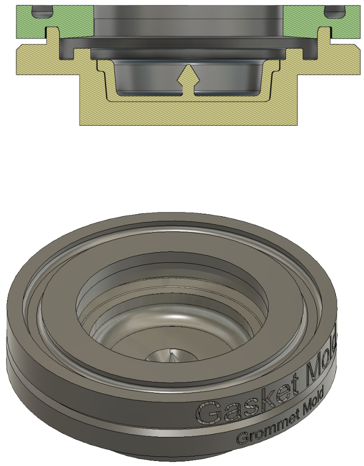

Figure 7-4. Place Gasket Mold on top of Grommet Mold.

Chapter 8. Peristaltic Pump Assembly

You will need:

1. Peristaltic Pump Exploded Diagram
2. Not Printed
    1. Silicone Tubing (Tygon 3350)
        1. 3x10 cm
        2. 2x 300 cm
    2. Glycerol Lubricant
    3. Scissors or another cutting implement
3. Printed
    1. Barbed Connectors (5x)
    2. Pump Head Body
    3. Tube Clip
    4. Rollers
    5. Front Rack
    6. Back Rack
    7. Bearing
    8. Snap Ring
    9. Motor Mount

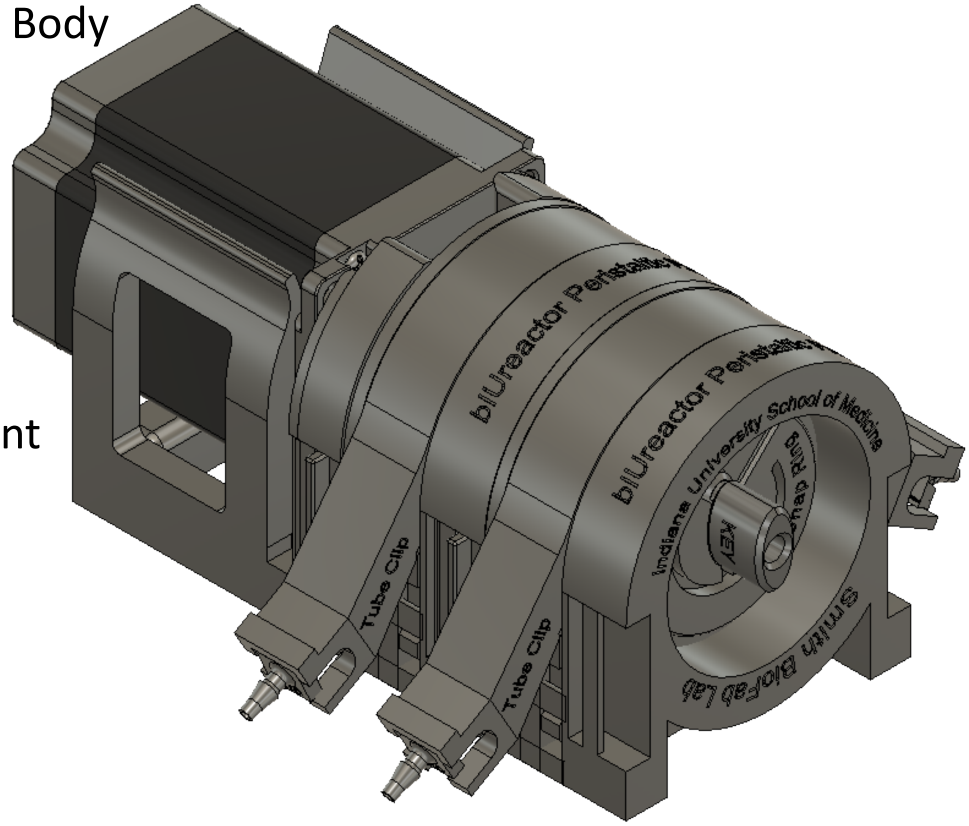

Peristaltic Pump Exploded Diagram

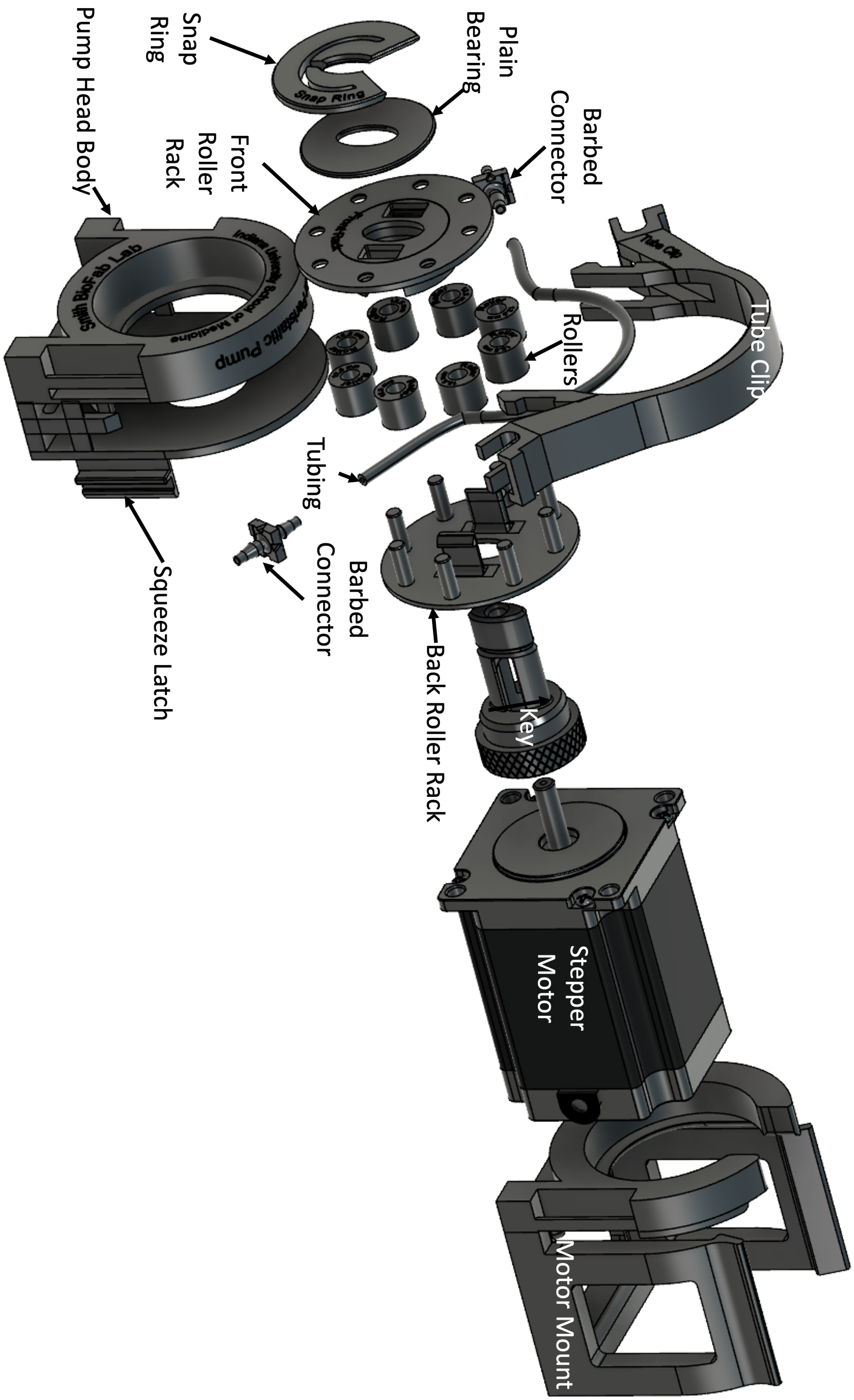

Figure 8-1. Peristaltic Pump Exploded Diagram.

Roller Assembly

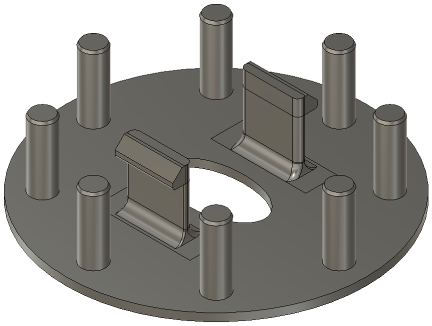

Figure 8-2. Place Back Rack with Spindles facing up.

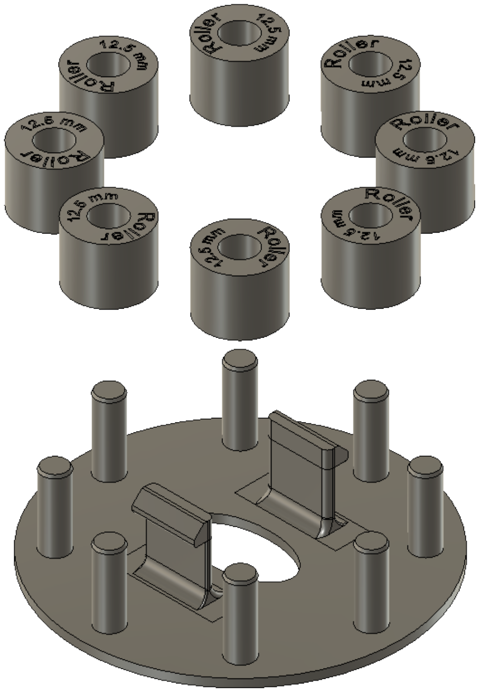

Figure 8-3. Place Rollers onto Spindles.

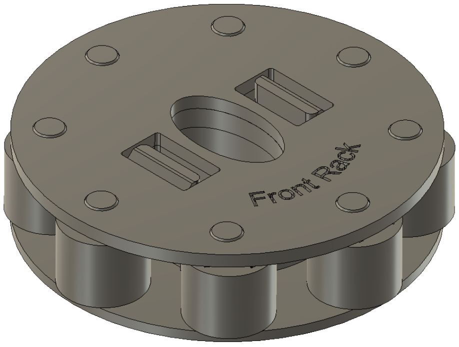

Figure 8-4. Roller Assembly is now complete.

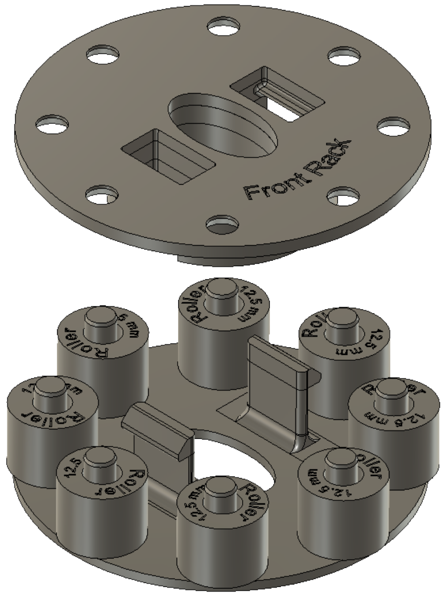

Figure 8-5. Secure Front Rack onto Back Rack. Ensure Spindles are aligned with holes on Back Rack. Ensure Back Rack Clips into Front Rack. Brush Glycerol onto Rollers.

Pump Head Assembly

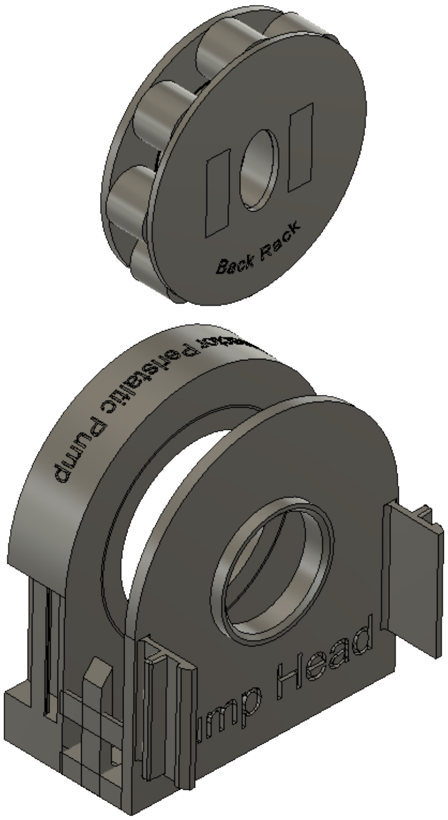

Figure 8-6. Place Roller Assembly in Pump Head Body.

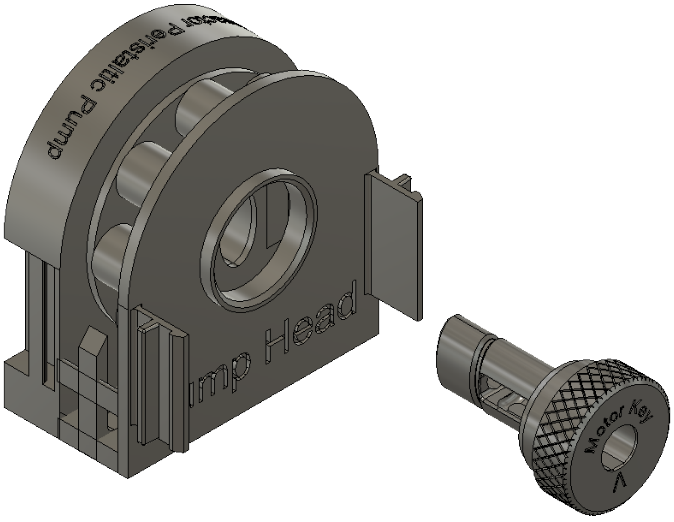

Figure 8-7. Place Motor Key into the slot of the Roller Assembly.

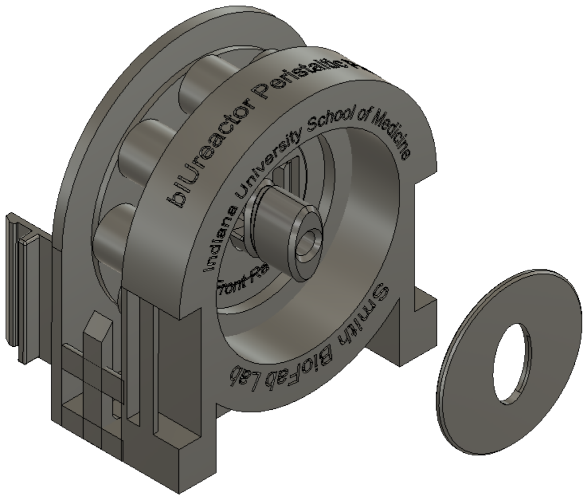

Figure 8-8. Turn the assembly around. Place Plain Bearing onto Key.

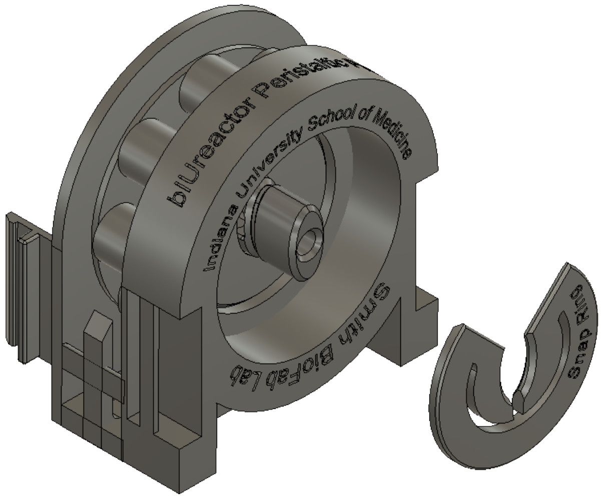

Figure 8-9. Secure Snap Ring onto Key. Ensure it snaps into place.

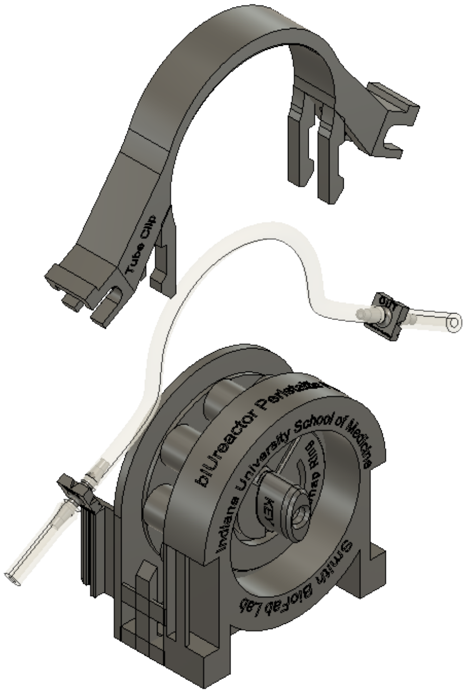

Figure 8-10. Drape the Tubing Assembly over the Rollers. Secure the Tube Clip onto the Pump Head Body.

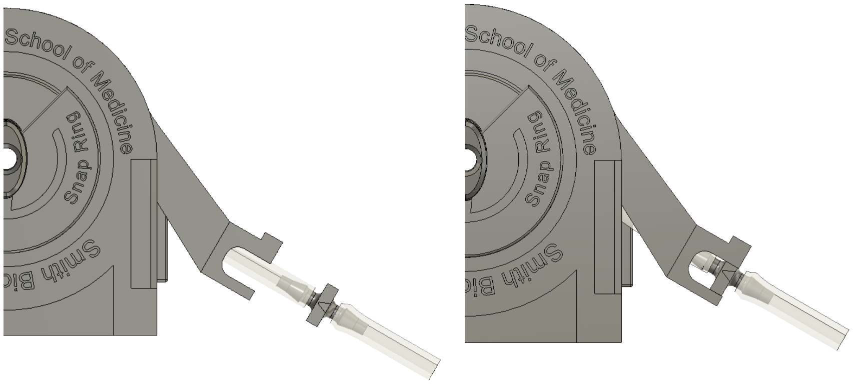

Figure 8-11. Clip the Barbed Connectors into the holders on the end of the Tube Clip.

Motor-Motor Mount Assembly and Mounting Pump Head

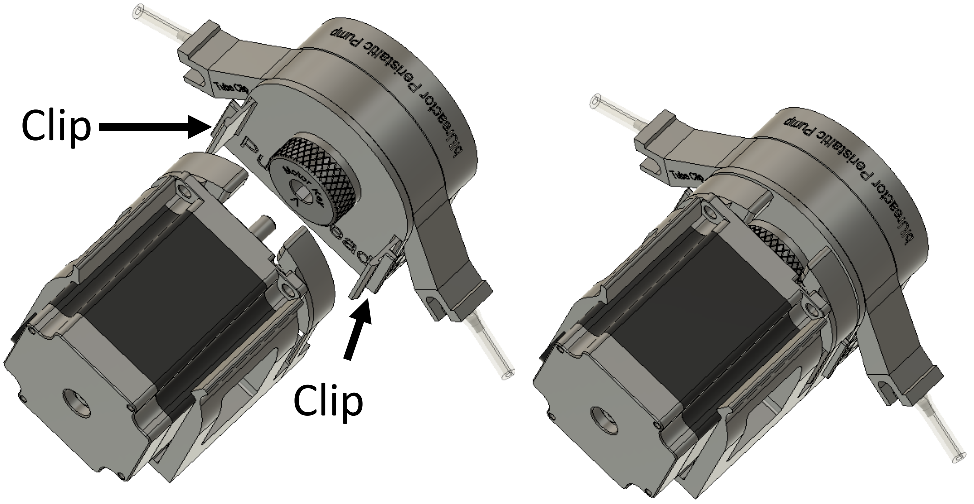

Figure 8-12. Align Stepper Motor shaft with Motor Key D slot. Squeeze Pump Head clips gently. Push Motor Assembly onto Pump Head until the Clips snap into place. This completes Pump Assembly.

Adding an additional Pump Head

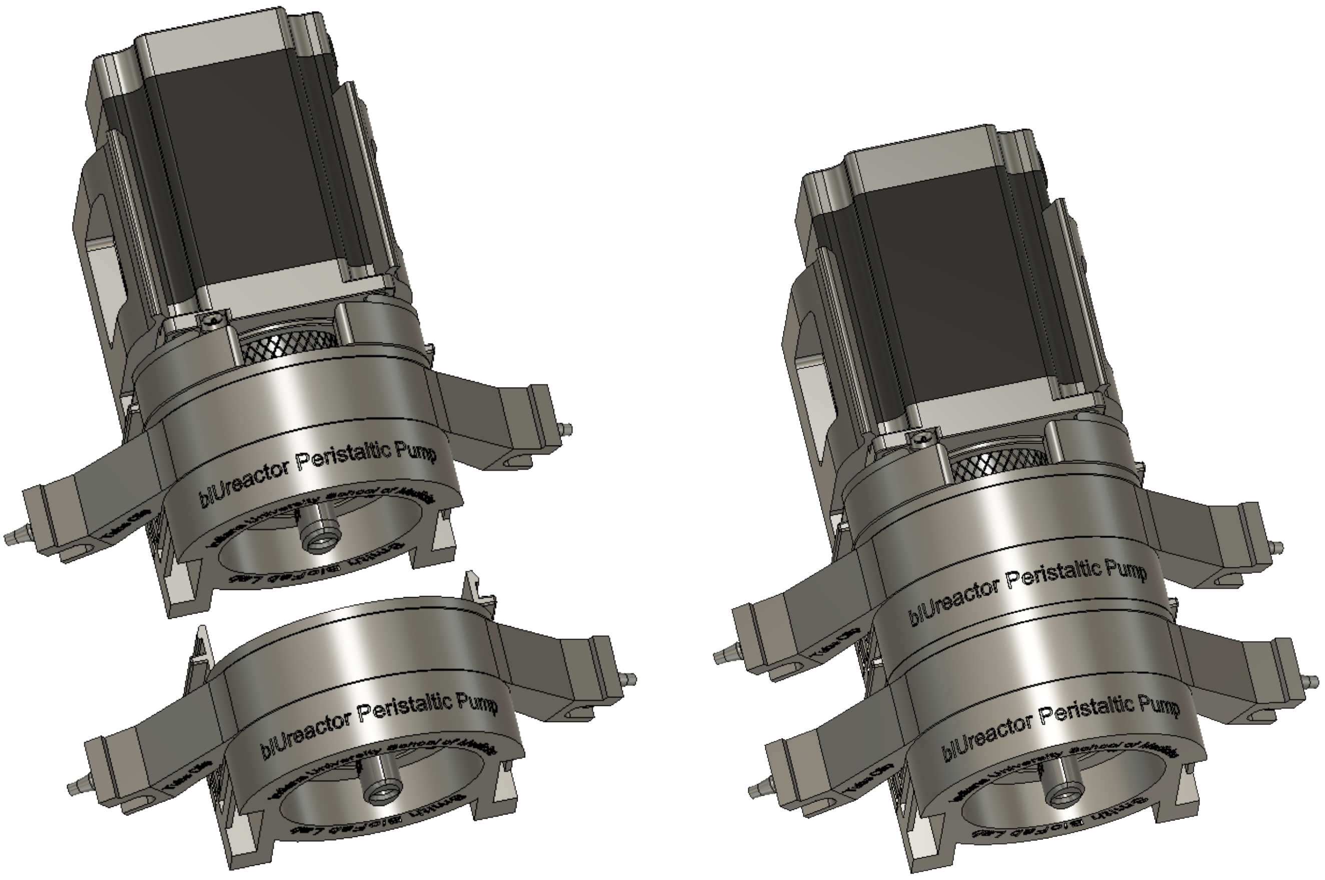

Figure 8-13. Each Stepper Motor can power up to 4 Pump Heads. After the first Pump Head, the remaining Pump Heads have a Key inserted through the Roller Assembly, not a Motor Key. Align Keys. Squeeze Pump Head clips and push the additional Pump Head on the previously installed Pump Head

Photo of Fully-Assembled Pump

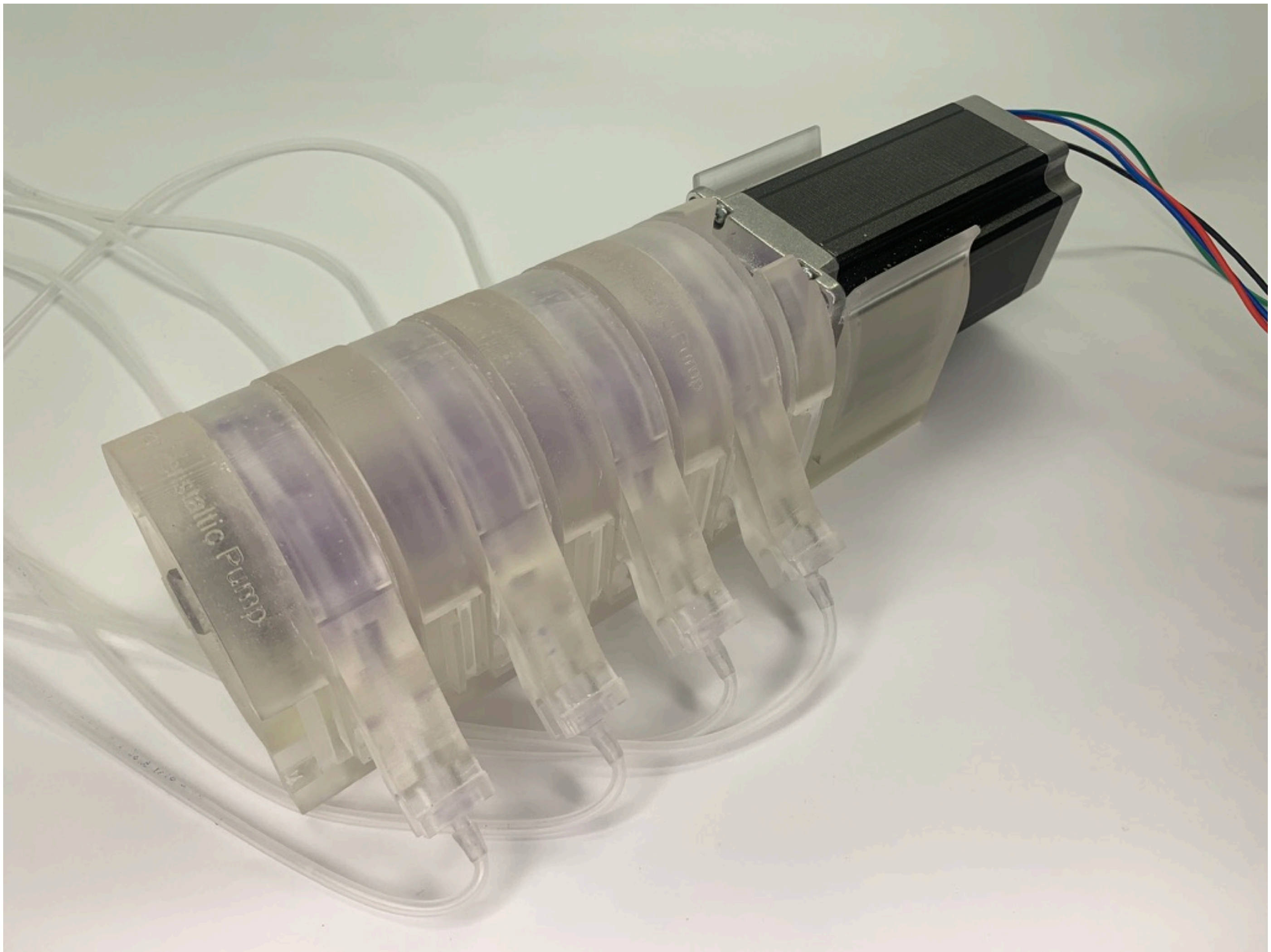

Figure 8-14. Fully-Assembled Peristaltic Pump with 4 pump heads

You will need:

1. Not printed
    1. Silicone (Elastosil or Sylgard 184)
    2. Mold Release
    3. Glycerol Lubricant
    4. Syringe
2. Printed
    1. Valve Body
    2. Insert Base
    3. Mold Cover
3. Optional
    1. Capped Test Tubes
    2. Centrifuge

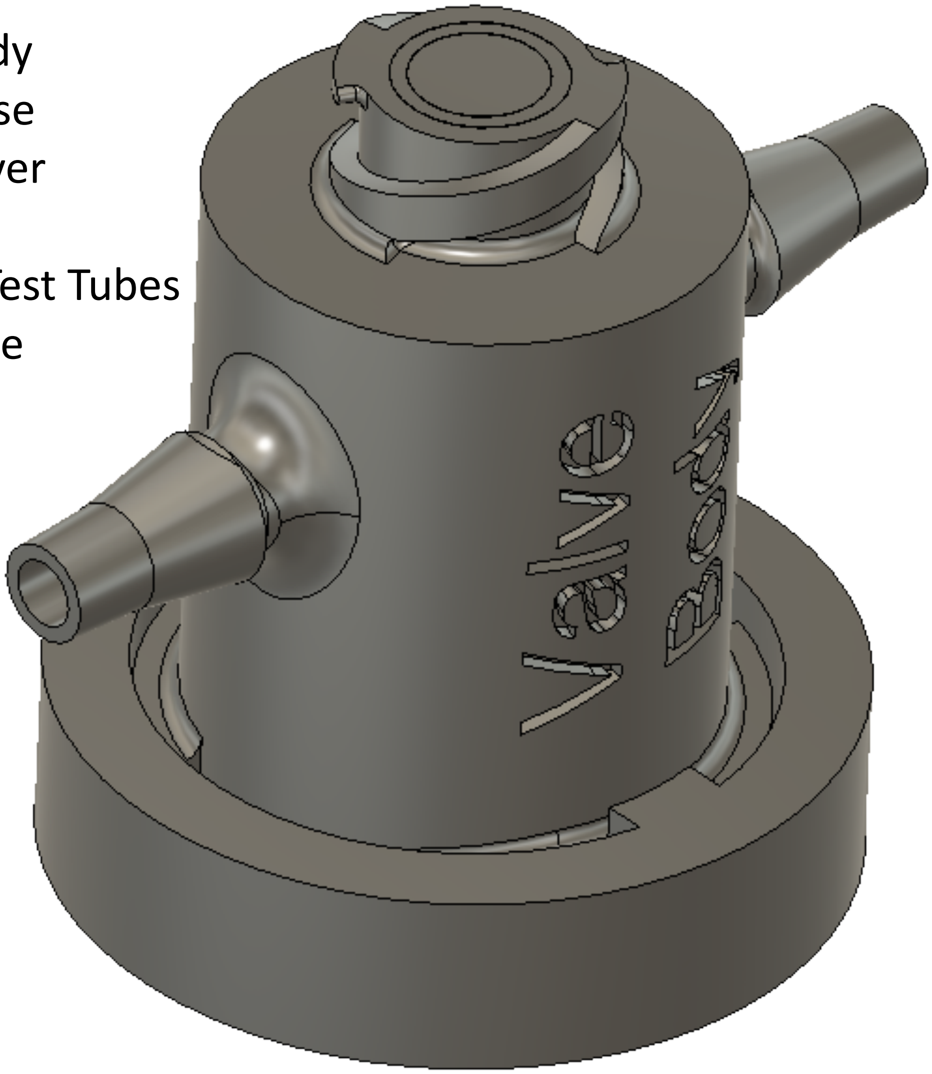

Needle-Free Valve Casting

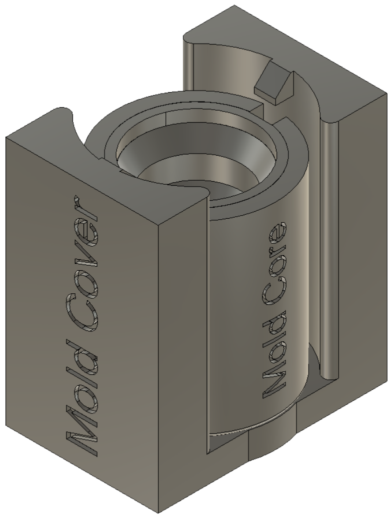

Figure 9-1. Secure Mold Core into Mold Cover.

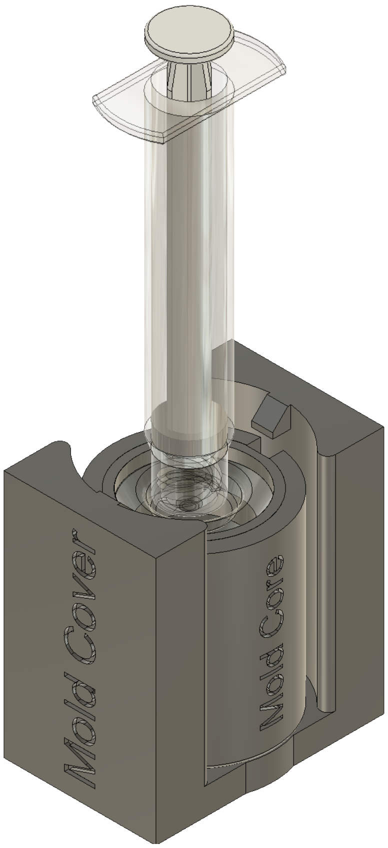

Figure 9-2. Pump silicone into Mold Core.

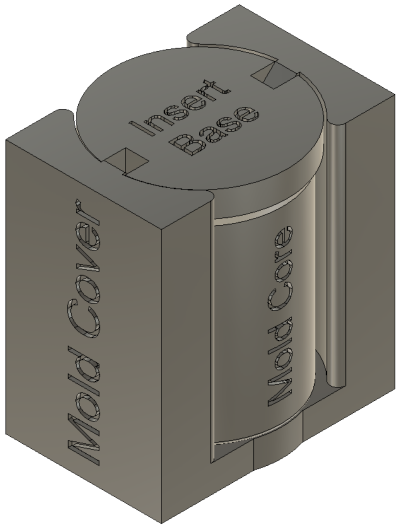

Figure 9-3. Secure Insert Base to Mold Cover over Mold Core. Cure silicone per manufacturer instructions.

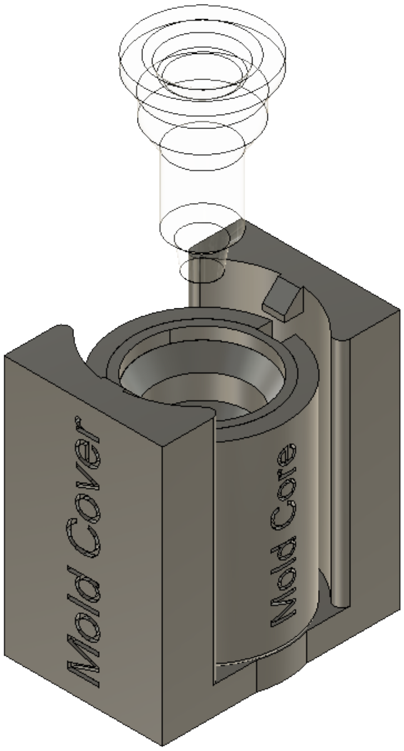

Figure 9-4. Demold Insert.

Needle-Free Valve Assembly

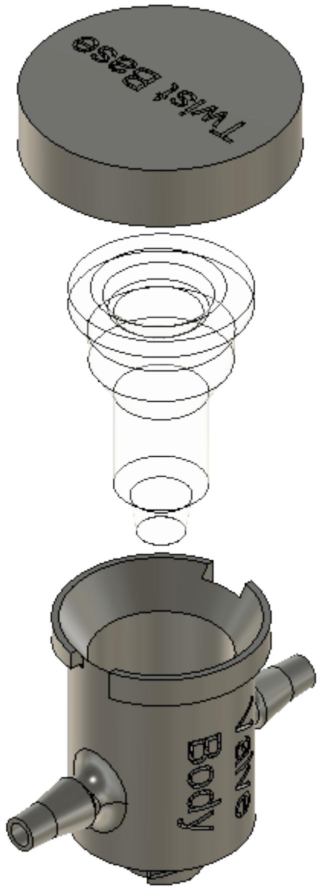

Figure 9-5. Place Silicone Insert into Valve Body.

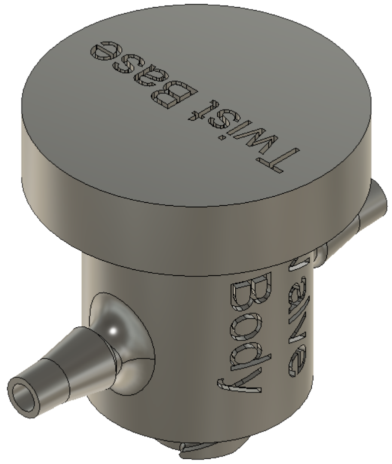

Figure 9-6. Cover with Twist Base. Twist Base text should be aligned with Valve Body Text.

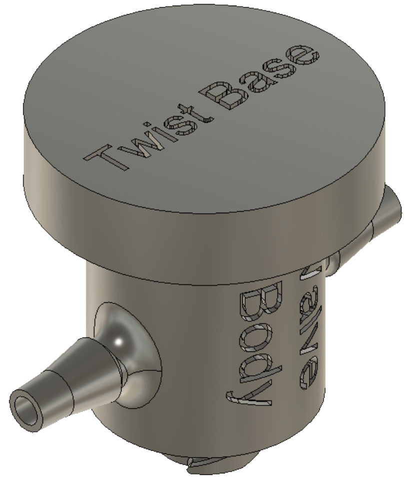

Figure 9-7. Rotate Twist Base 90 degrees Clockwise.

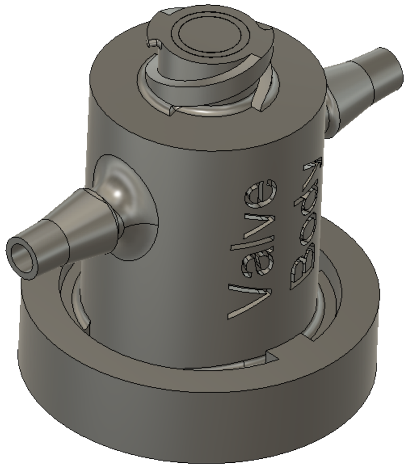

Figure 9-8. Needle-Free Valve Assembly is complete.

Needle-Free Valve Mode of Operation

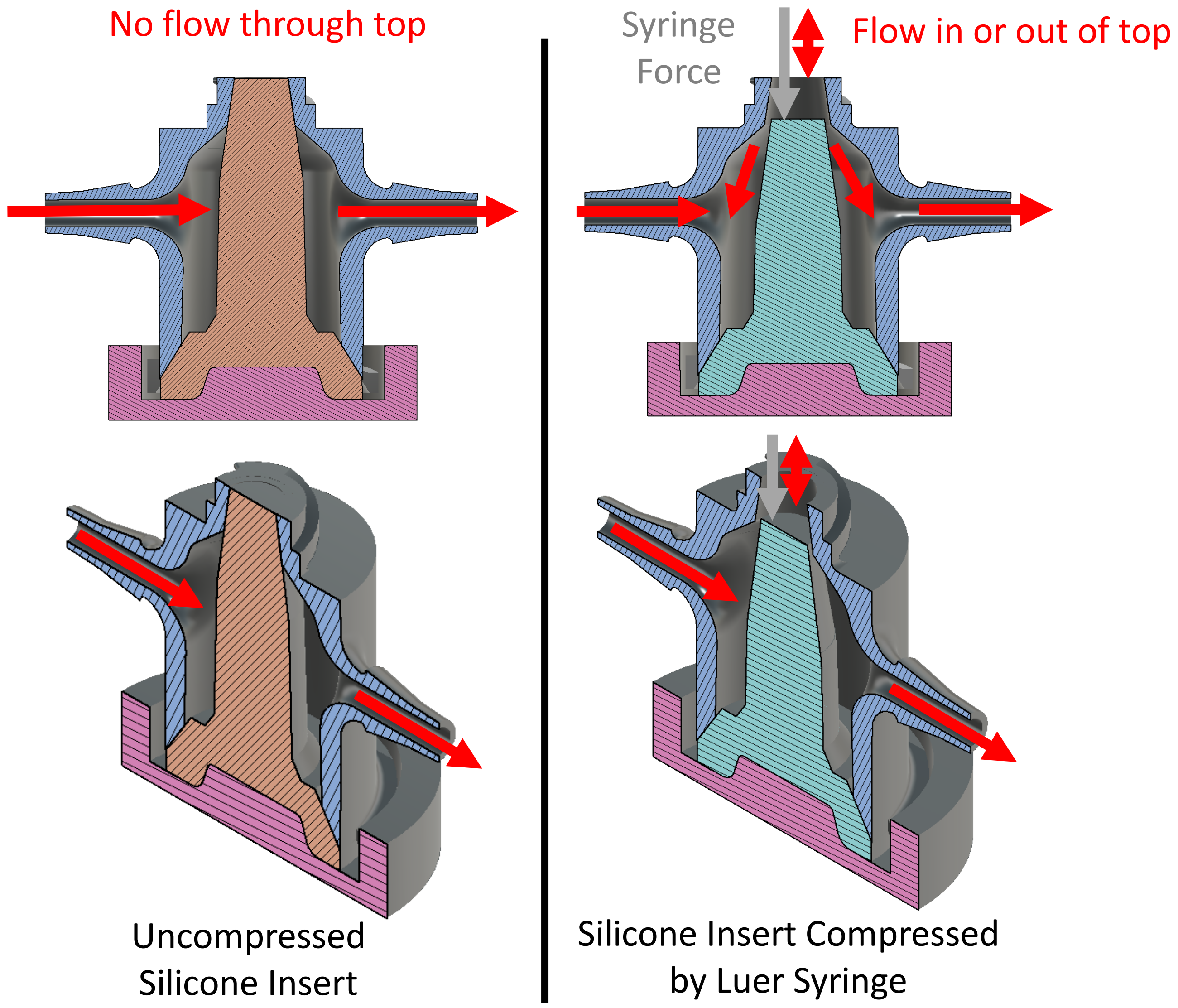

Figure 9-9. Diagram indicating Needle-Free Valve mode of operation.

Chapter 10. bIUreactor Chamber Assembly

You will need:

1. bIureactor Chamber Assembly Exploded Diagram
2. Not printed
    1. Mesh
3. Printed
    1. Grommet Lid
    2. bIUreactor Lid
    3. bIUreactor Chamber
    4. End Effector
4. Casted Silicone
    1. Grommet
    2. Gasket

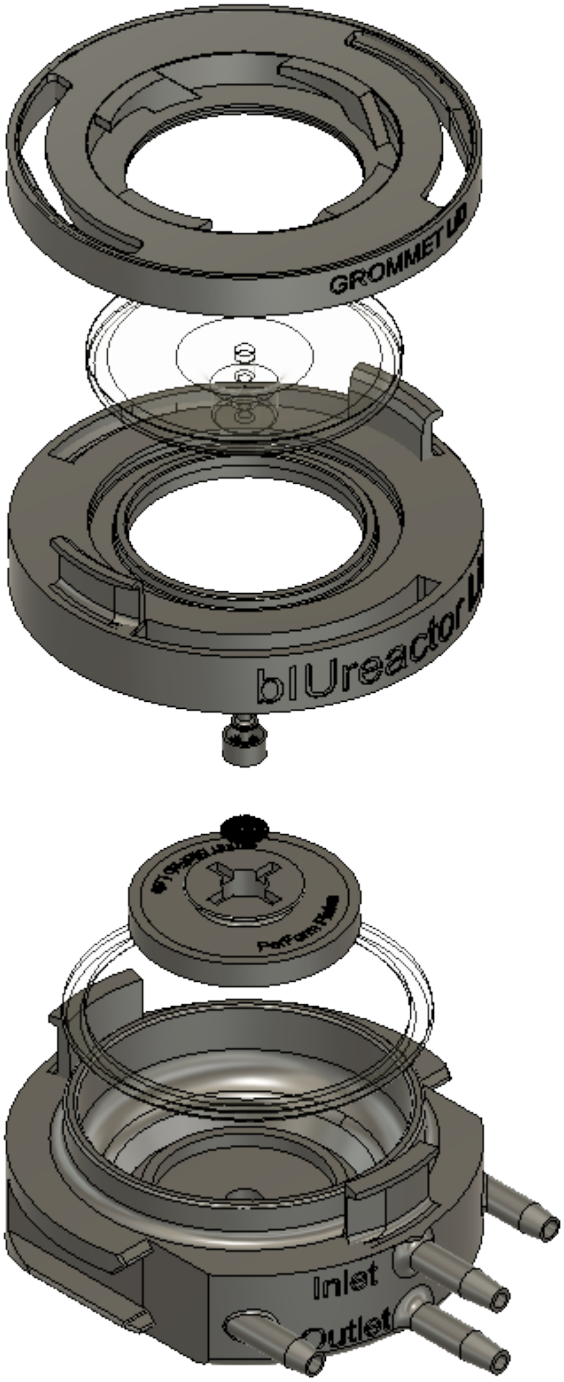

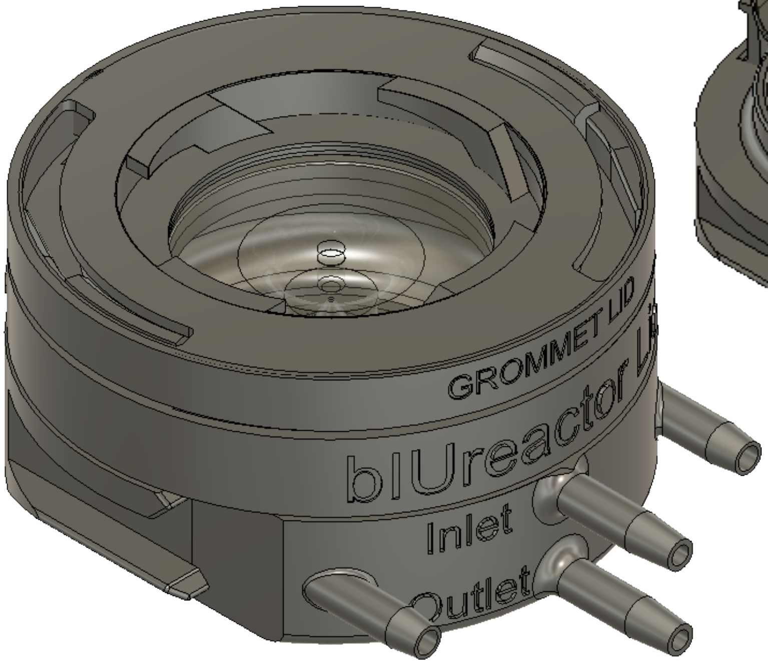

bIUreactor Chamber Exploded Diagram

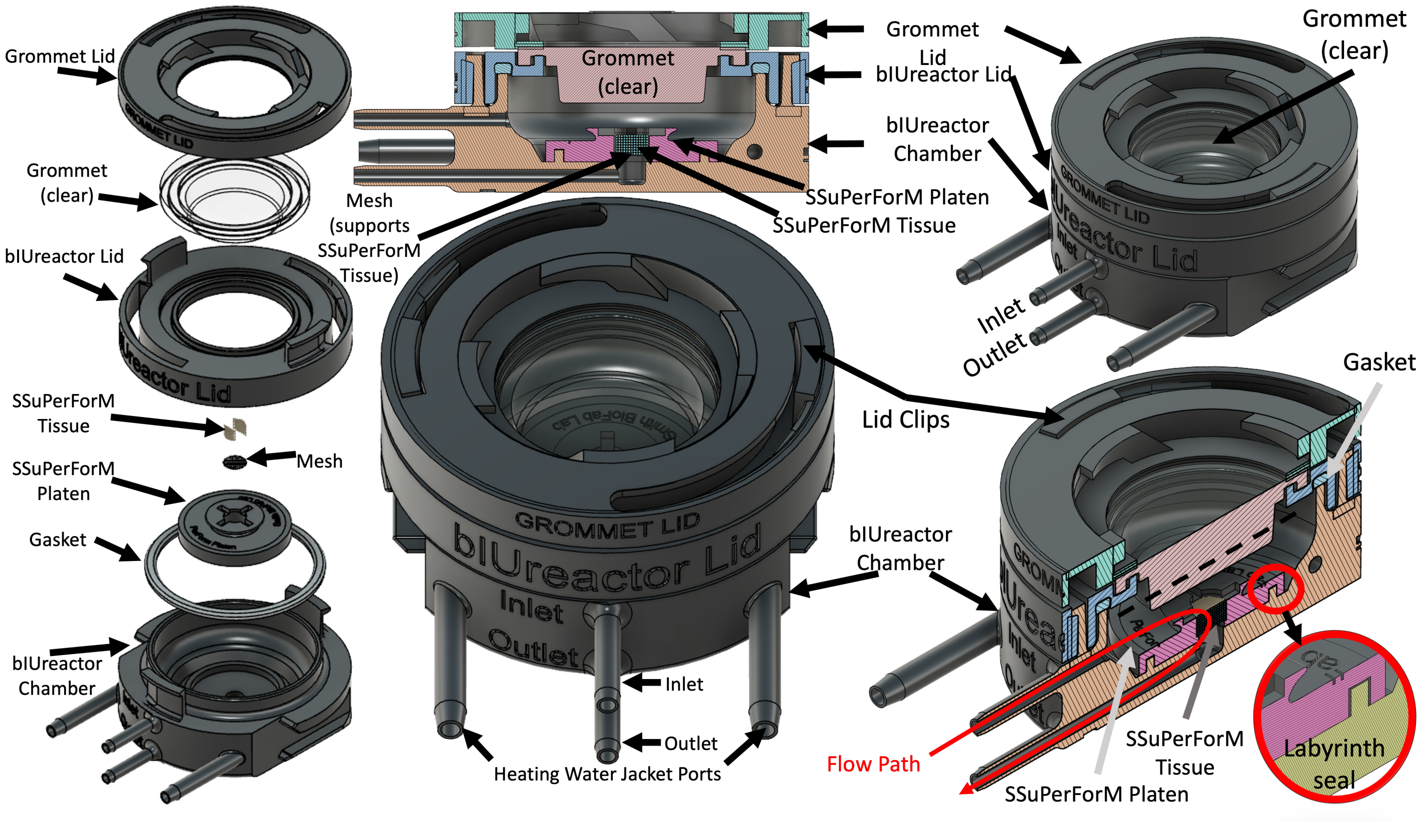

Figure 10-1. bIUreactor Chamber Exploded Diagram.

Lid Assembly

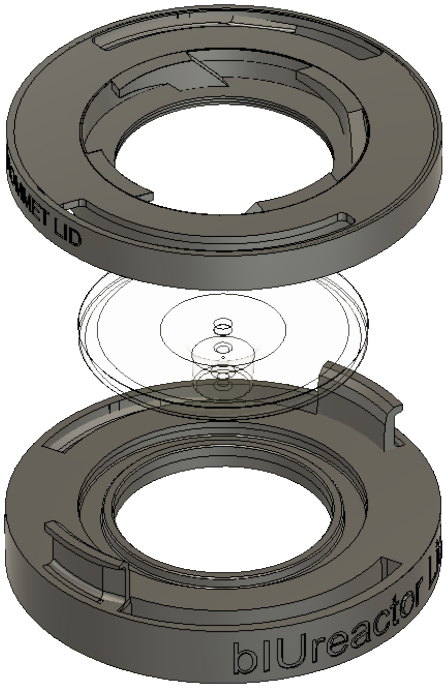

Figure 10-2. Place Grommet on top of bIUreactor Lid.

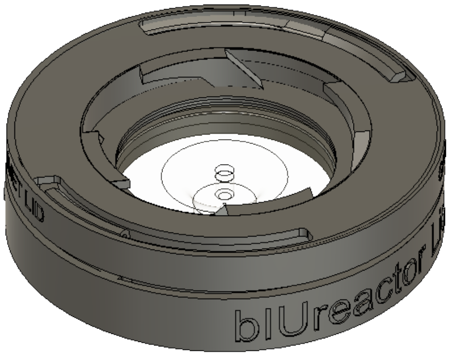

Figure 10-3. Place Grommet Lid on top of bIUreactor Lid with Grommet Lid text oriented 90 degrees to bIUreactor Lid.

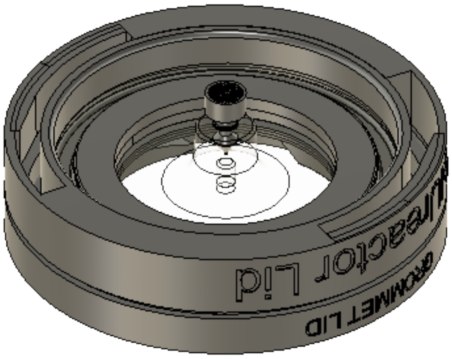

Figure 10-4. This completes Lid Assembly.

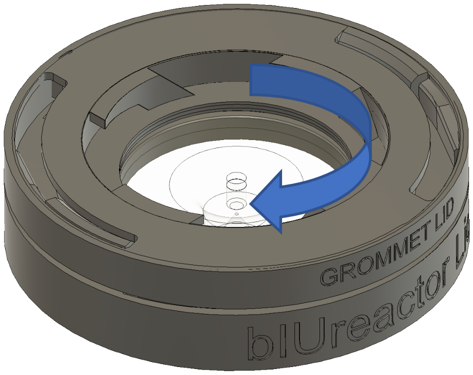

Figure 10-5. Twist Grommet Lid 90 degrees, securing the Grommet between the two lids. This is the Lid Assembly.

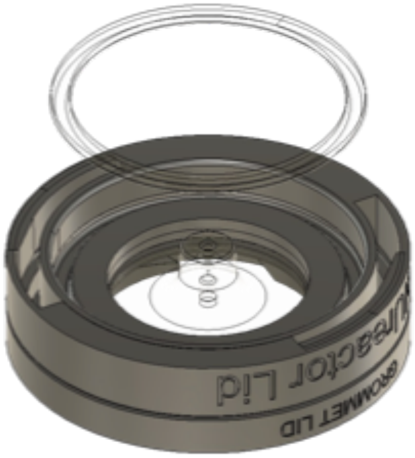

Figure 10-6. Invert Lid Assembly. Place Gasket inside groove inside bIUreactor Lid. Push the Gasket gently to ensure it is fully seated.

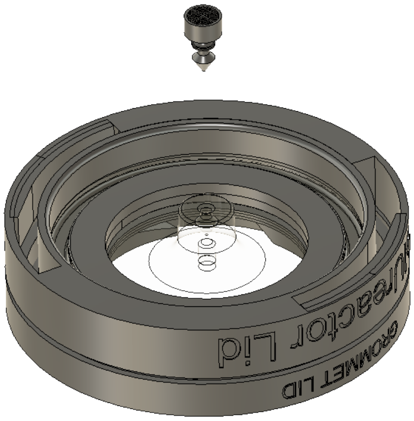

Figure 10-7. Secure End Effector into opening at the bottom of the Grommet.

Chamber Assembly

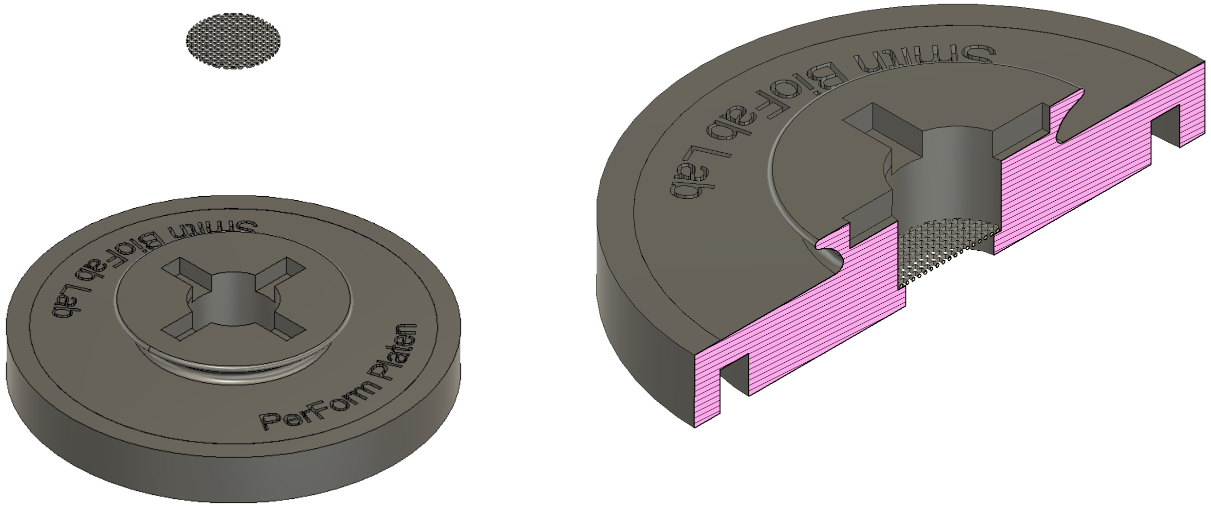

Figure 10-8. Cut out a 6-7 mm disk of Mesh using a Biopsy Punch. Place Mesh into Platen center, ensuring it rests on the inner annular edge. The Mesh can be handled using forceps.

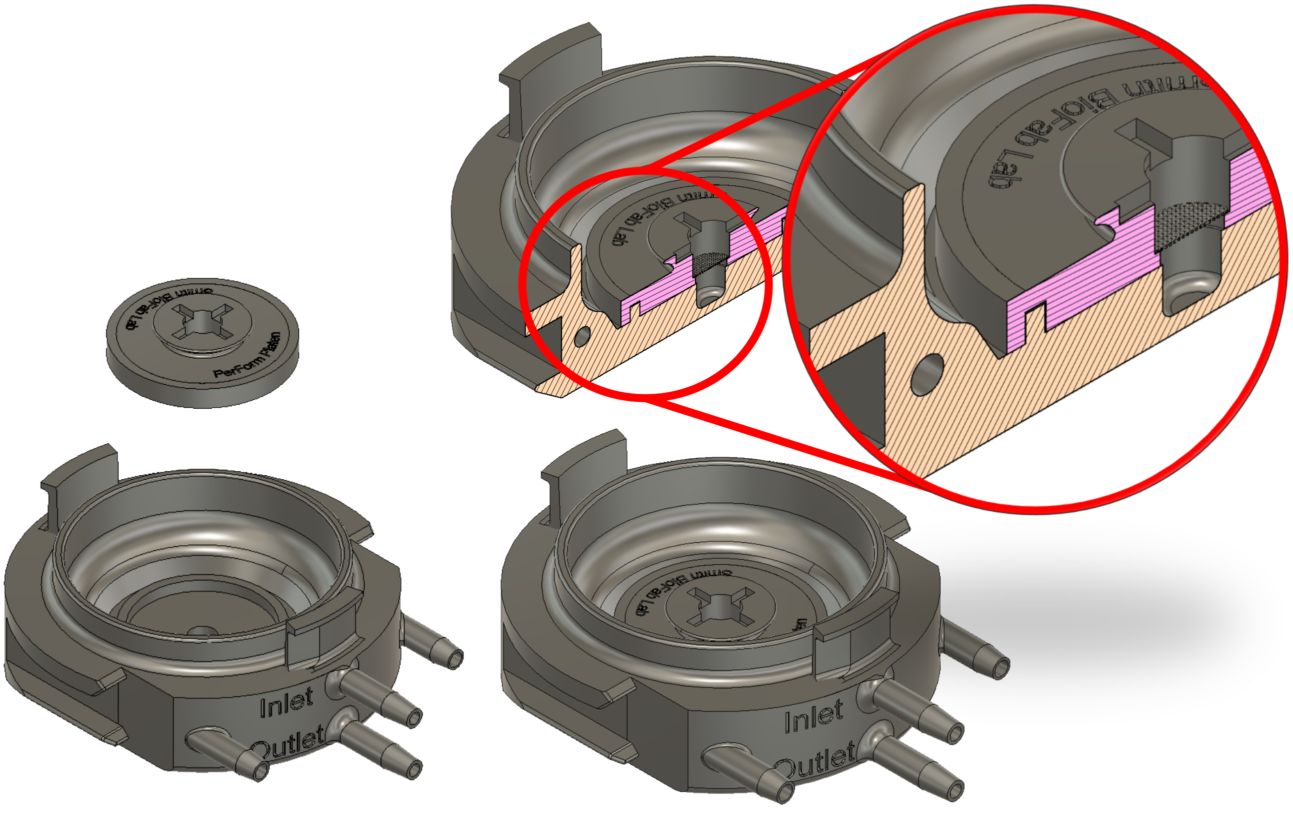

Figure 10-9 Place Platen into Chamber, ensuring the groove on the bottom of the Platen fits snuggly over the ring in the Chamber center.

Mating Lid to Chamber

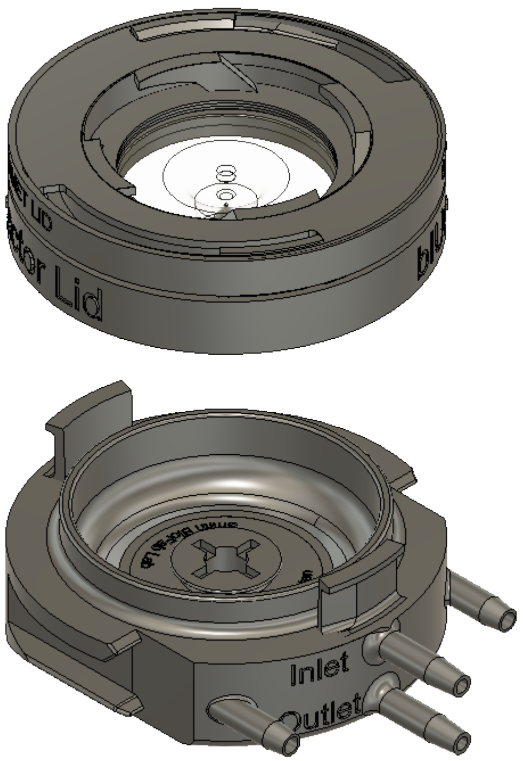

Figure 10-10. Align the openings on the bottom of the lid assembly with the tabs on the bIUreactor. Place the Lid Assembly onto the Chamber.

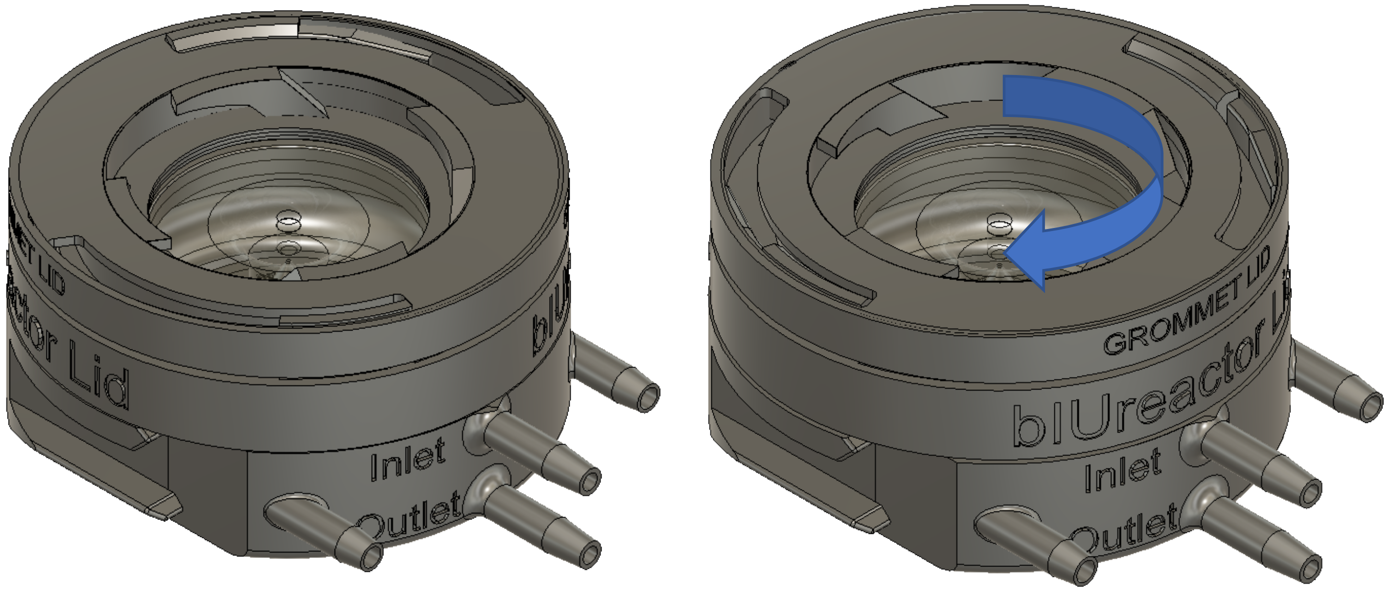

Figure 10-11. Twist the Lid Assembly until the lettering on the Lid is aligned with the Inlet and Outlet on the Chamber. This completes the bIUreactor Chamber Assembly.

Chapter 11. Compressor Assembly

You will need:

1. Assemblies
    1. bIUreactor Chamber Assembly
2. Not printed
    1. Stepper Motor
    2. Exploded Compressor Diagram
3. Printed
    1. Motor Mount
    2. GearBox A
    3. GearBox B
    4. Pinion (x4)
    5. Piston
    6. Worm Shaft
    7. Switch Key
    8. Cap
    9. Microswitch
    10. PET Mount

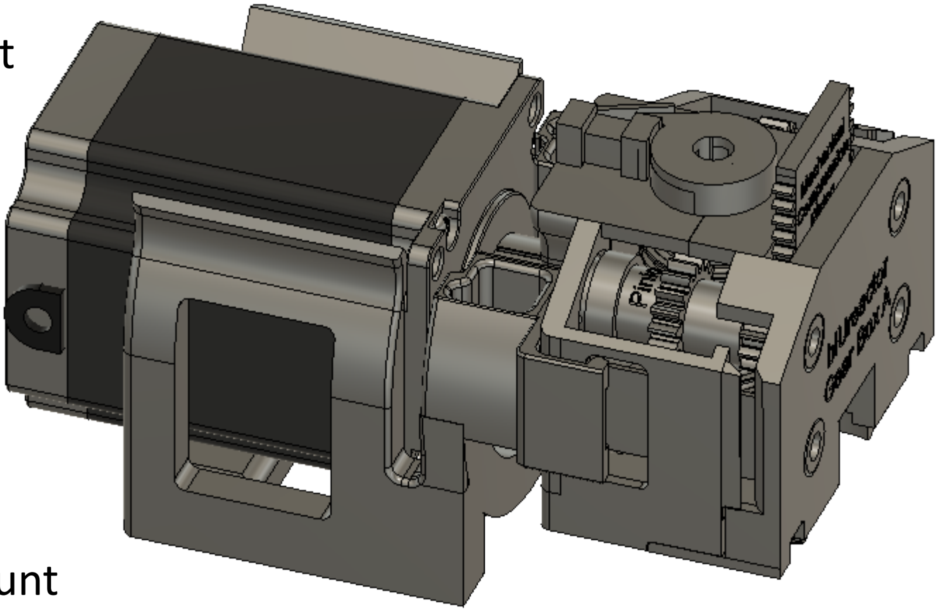

Compressor Exploded Diagram

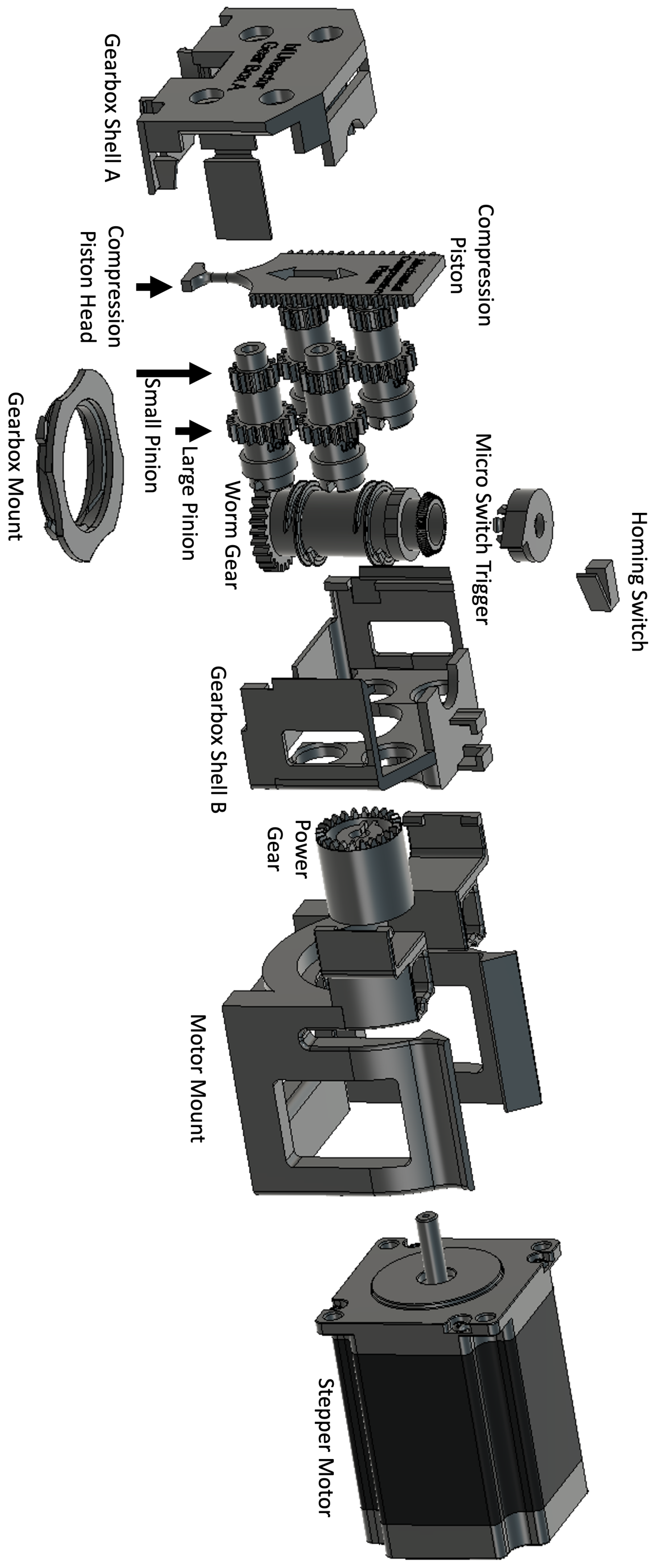

Figure 11-1. Compressor Exploded Diagram.

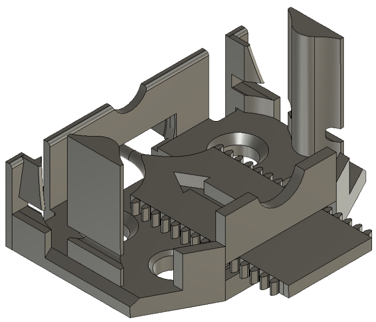

Figure 11-2. Insert Piston into slot as shown.

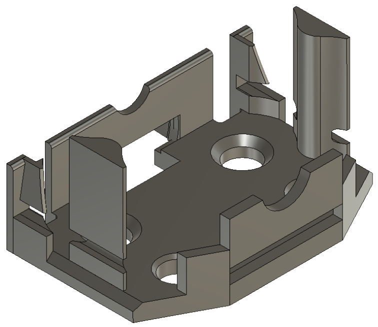

Figure 11-3. Lay Gearbox A with lettering facing down.

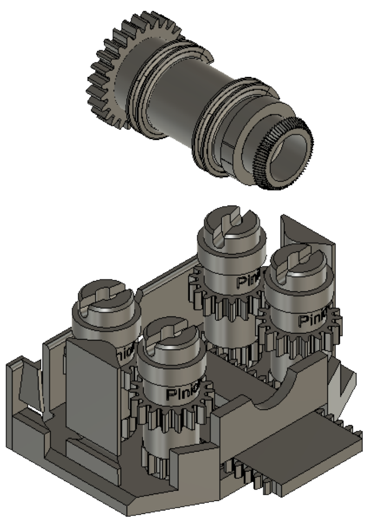

Figure 11-4. Place Worm Shaft into GearBox A between the Pinions. Ensure Pinion teeth engage with Worm gears.

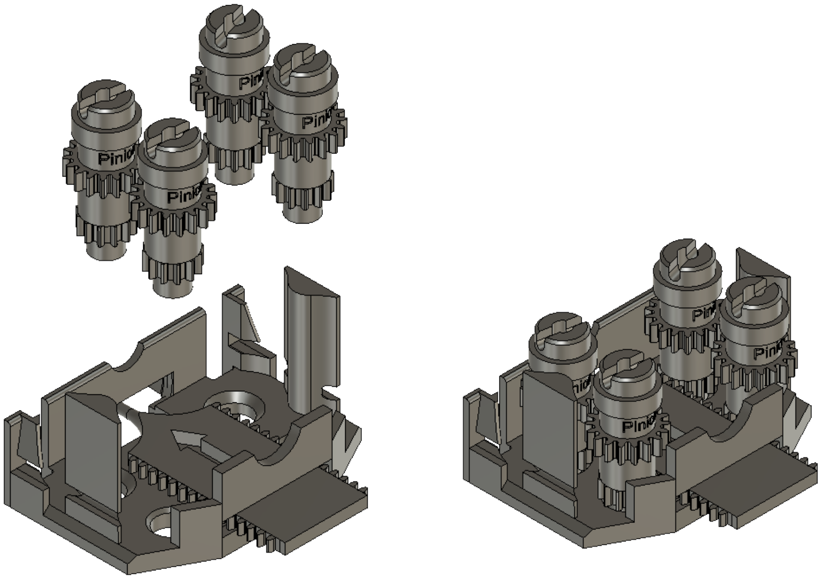

Figure 11-5. Place Pinions into holes on GearBox A with Pinion text on top. Ensure Pinion gears mesh with the Piston teeth.

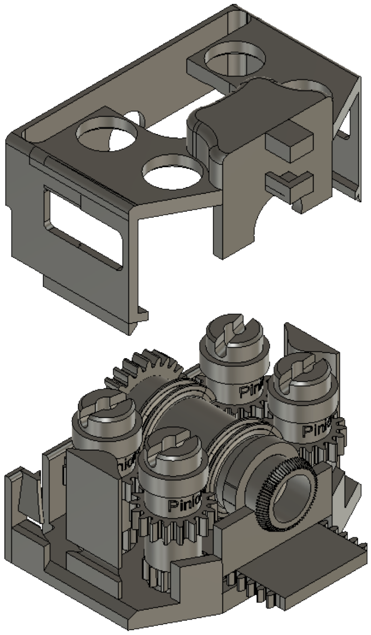

Figure 11-6. Secure GearBox B to GearBox A. The two components should snap into place.

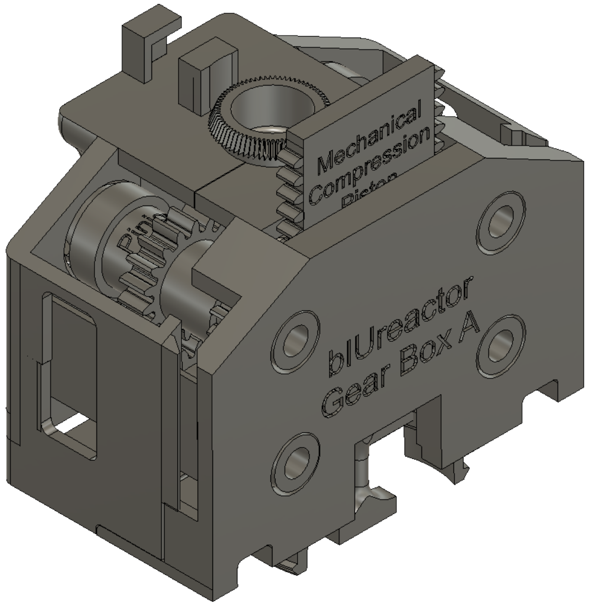

Figure 11-7. Stand Gearbox Upright.

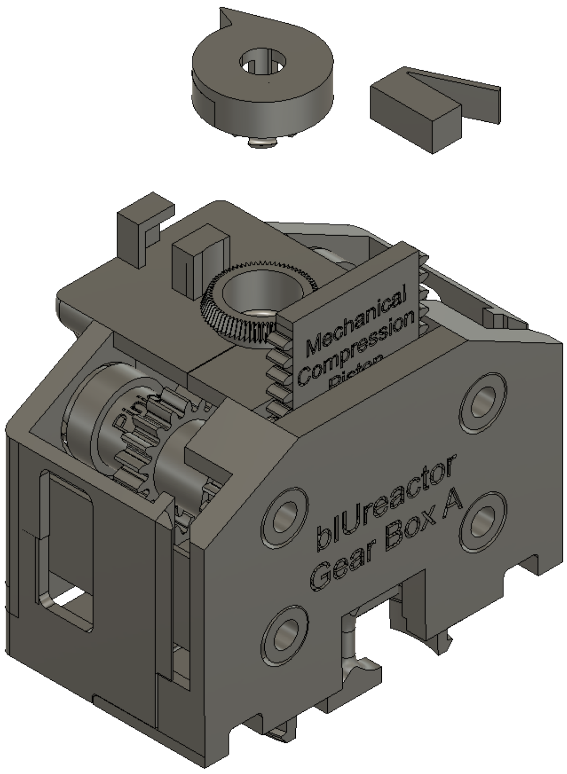

Figure 11-9. Secure GearBox MicroSwitch Trigger and Microswitch into place.

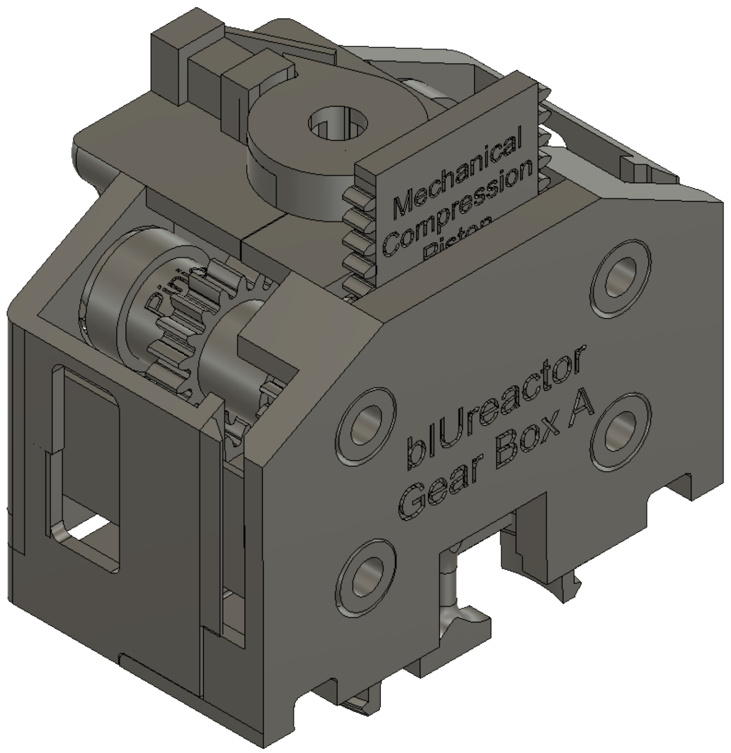

Figure 11-8. This completes Gearbox Transmission Assembly.

Motor Mount Assembly

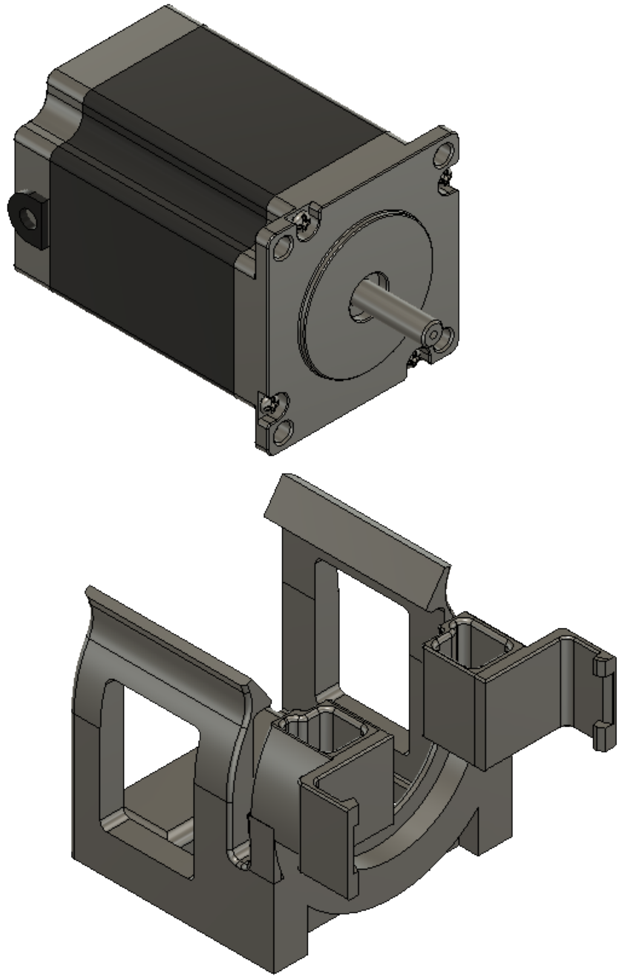

Figure 11-10. Place Stepper Motor into Motor Mount. Ensure arms on Mount snap into place over the Stepper.

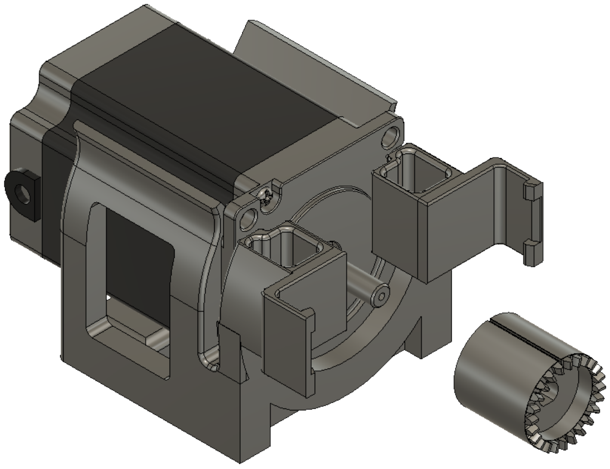

Figure 11-11. Slide Power Gear onto Stepper Motor. Ensure the Power Gear aligns with the D-Slot on the Stepper Motor Shaft.

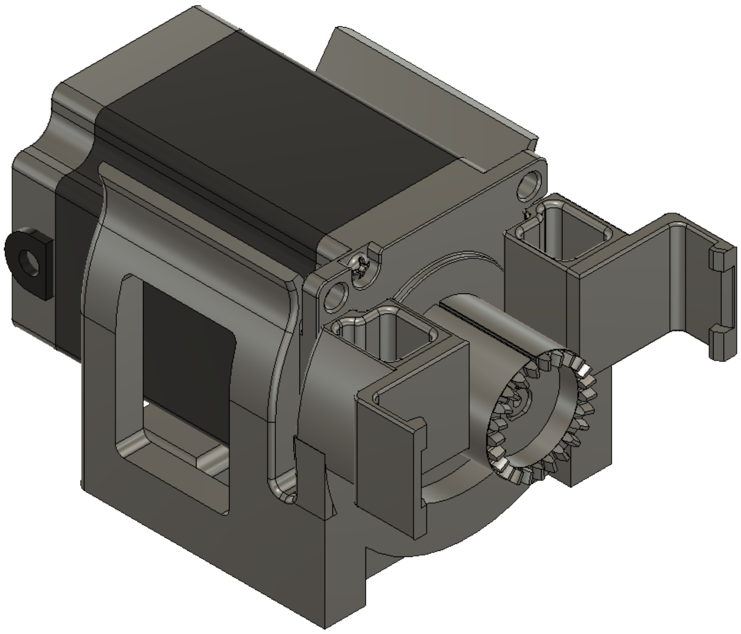

Figure 11-12. This completes Motor Mount Assembly.

Mating Gearbox to Motor Mount

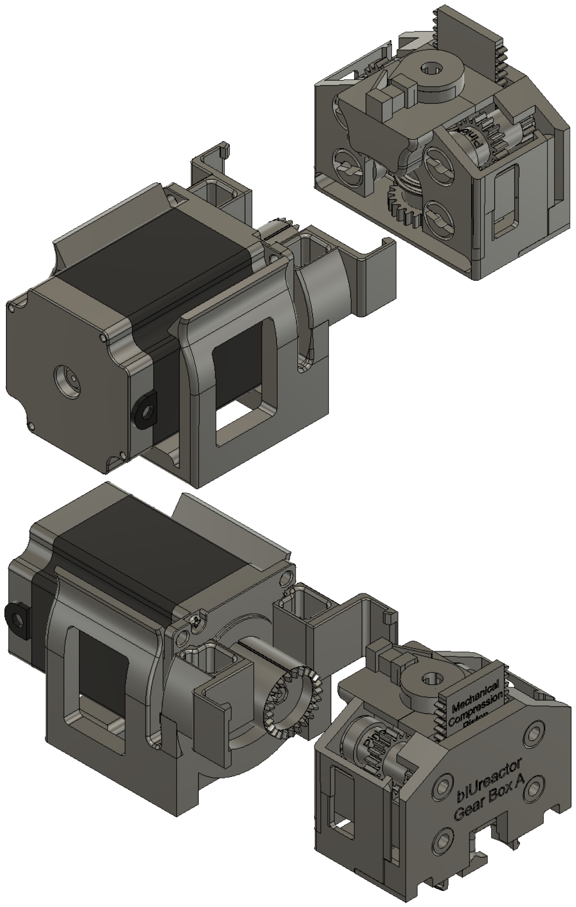

Figure 11-13. Align the two assemblies so the Power Gear can align with the Bottom Gear on the Worm Shaft.

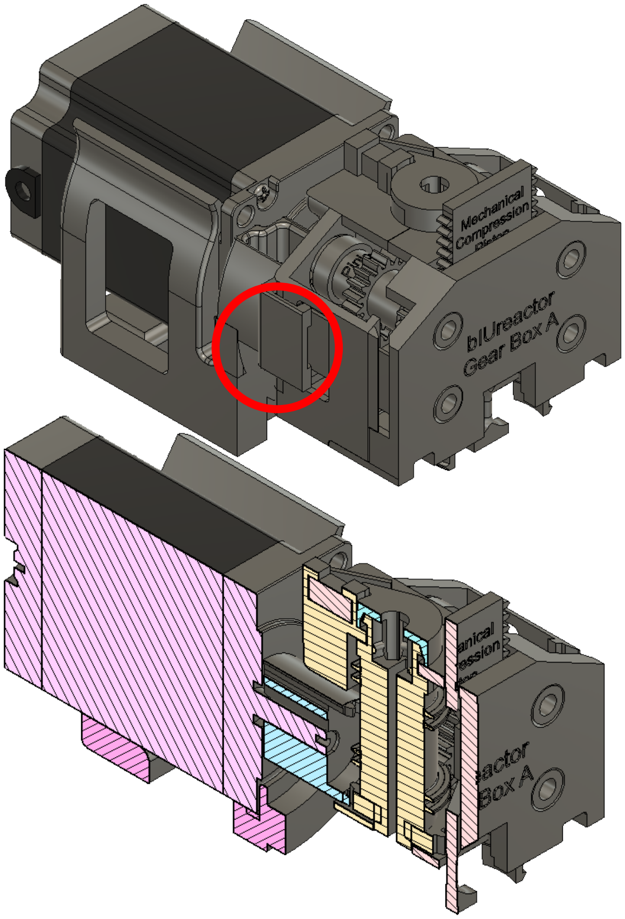

Figure 11-14. Push the Motor Mount onto the GearBox. Ensure the Mount Arms snap into place.

Installing PET Mount

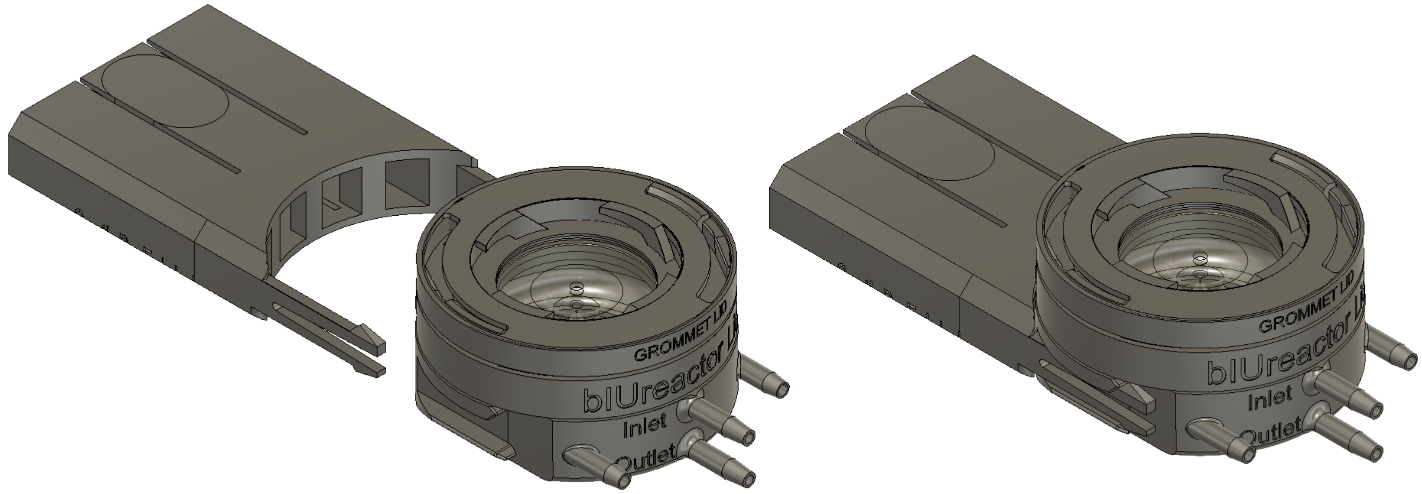

Figure 11-16. The PET Mount must be used with the Gearbox Assembly. Secure the PET Mount to the bIUreactor Chamber. Ensure the Forks are engaged and snap into place. This can be done before or after mating the Gearbox to the Motor Mount.

Chapter 12. Mating Chamber to Gearbox

You will need:

1. Assemblies
    1. Chamber Assembly with PET Mount
    2. Compressor Assembly
2. Printed
    1. GearBox Mount

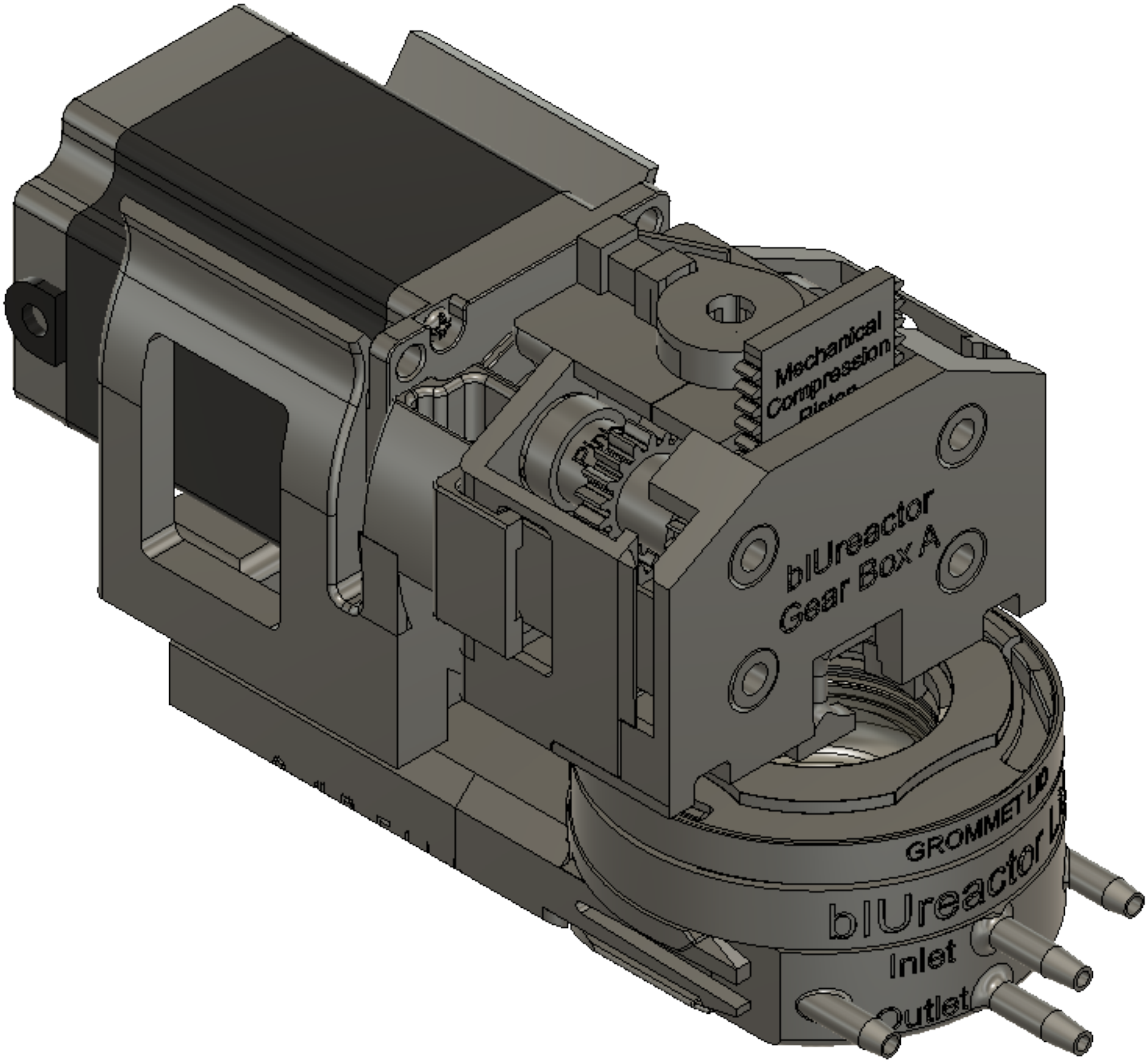

Mating Chamber Assembly to Gearbox Assembly

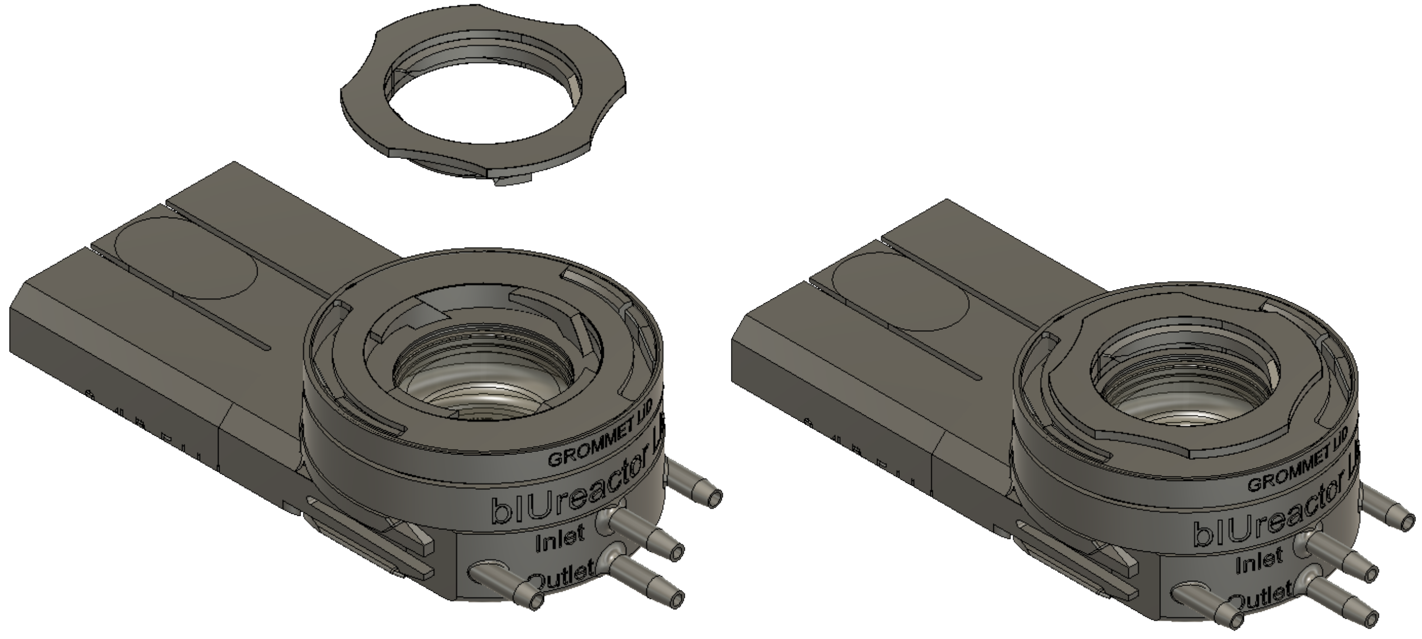

Figure 12-1. Place GearBox Mount onto Grommet Lid of Chamber Assembly.

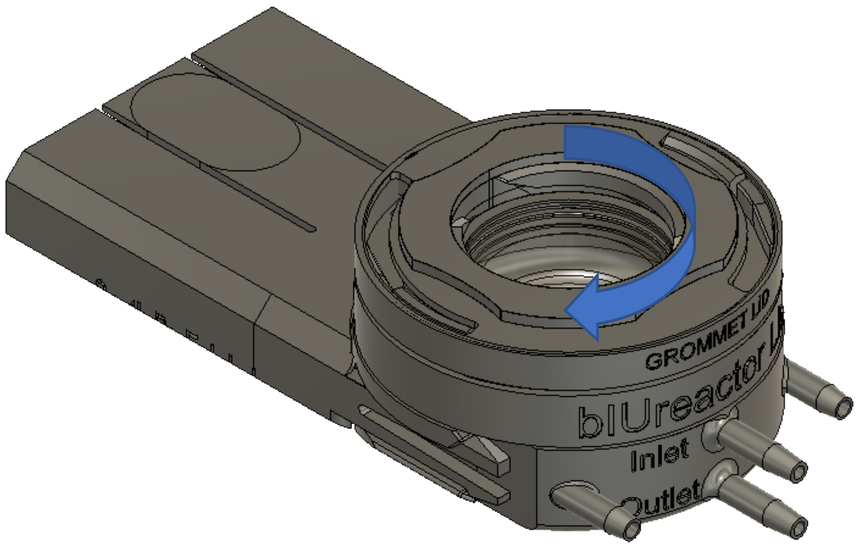

Figure 12-2. Twist GearBox Mount, securing it to the Grommet Lid.

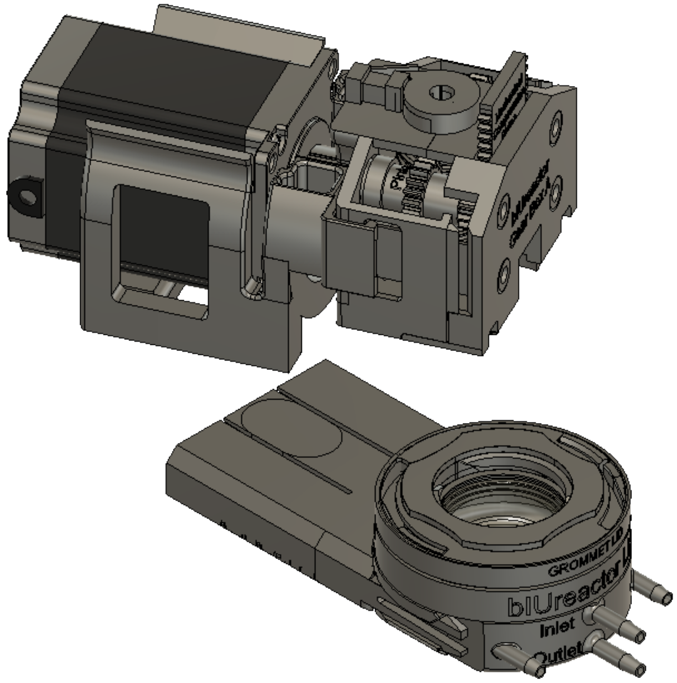

Figure 12-3. Align Compressor over the central axis of the Grommet Lid. The Compressor Assembly should be oriented an eighth to a quarter turn counter-clockwise from the Chamber Assembly.

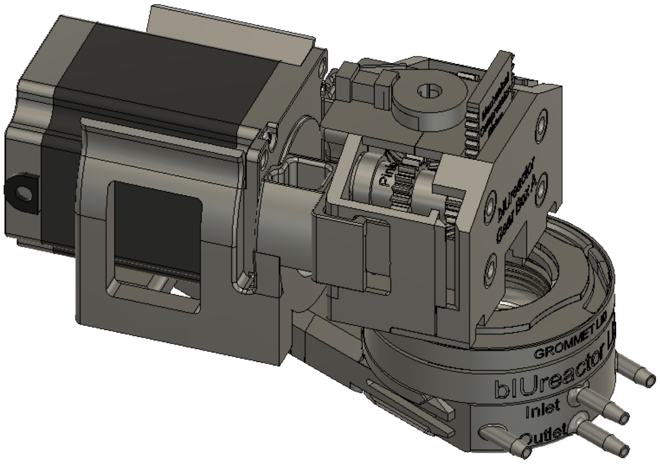

Figure 12-4. Place Compressor Assembly onto Motor Mount.

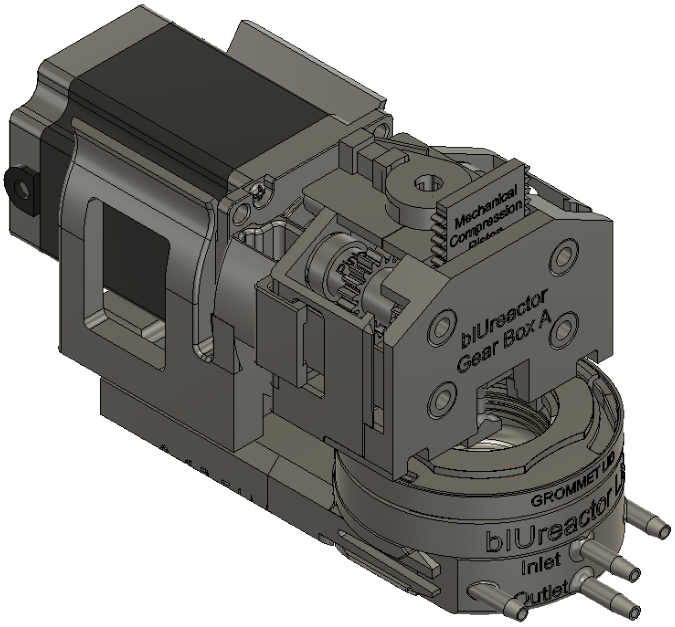

Figure 12-5. Twist Compressor Assembly clockwise until the arms lock into place and it is aligned with the Chamber.

Chapter 13. Motor Control Box Assembly

The Motor Control Box can be used to control a Pump with up to 4 heads or a Compressor which cyclically compresses tissues

You will need:

1. Not printed (See Supplement 2 for component purchasing information)
    1. Exploded Motor Control Box Diagram
    2. Compressor Circuit Diagram
    3. Peristaltic Pump Diagram
    4. Programmed Arduino Uno 3
    5. Shield Kit
    6. Potentiometer(s) (2 for Compressor, 1 for Pump)
    7. Stepper Motor Driver
    8. Rocker Switch
        1. Switches direction for Pump
        2. Powers on and off for Compressor
    9. Power Supply with 12V adapter
    10. Wire Hook Up Kit
    11. Wire Nut Connetors
        1. Used to connect wires instead of solder
    12. Electric Fan
    13. Wire Stripper
2. Printed
    1. Motor Control Box
    2. Motor Control Box Lid

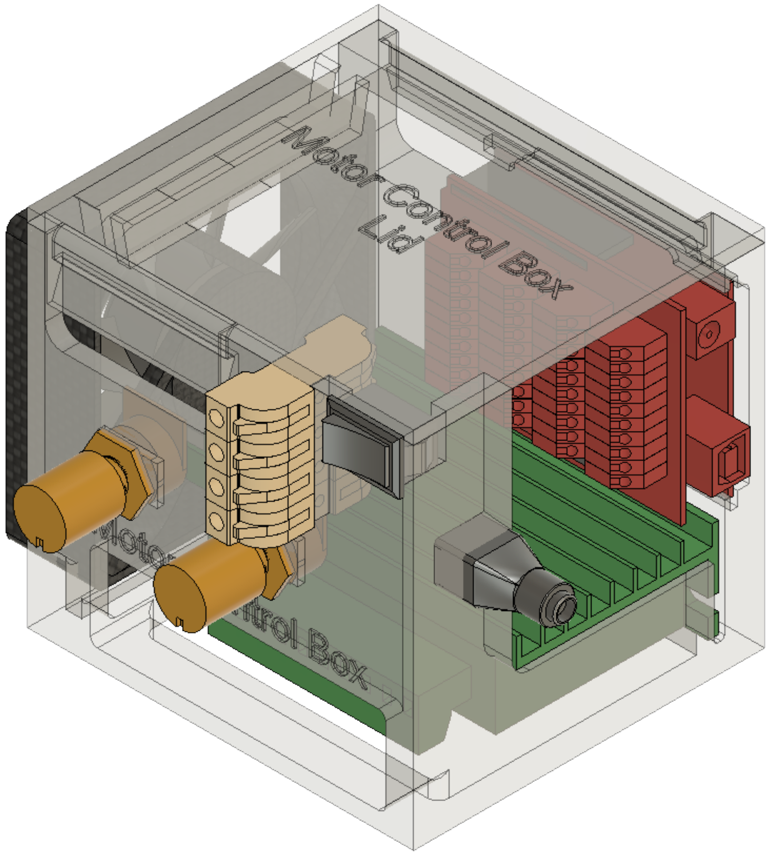

Motor Control Box Exploded Diagram

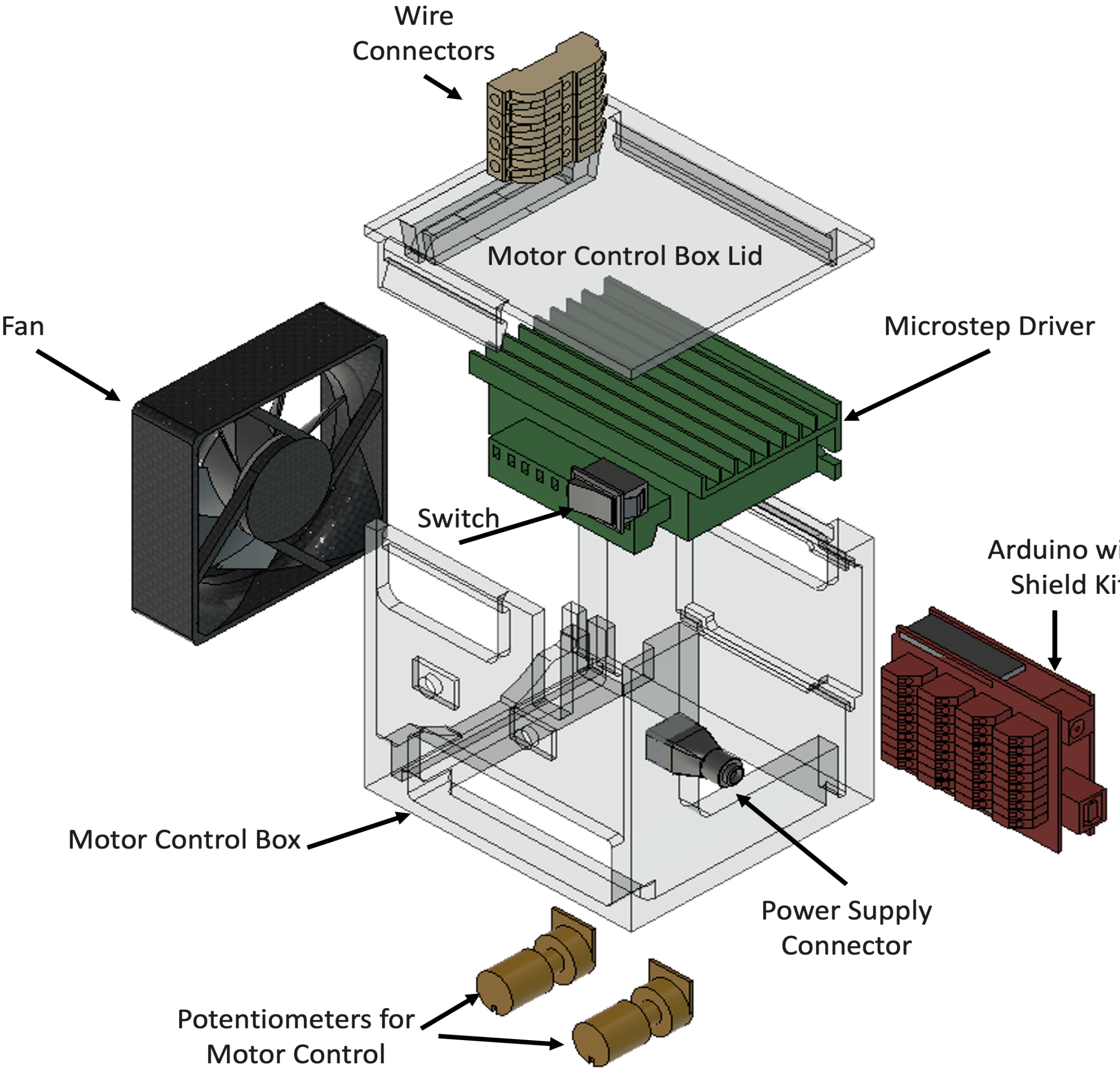

Figure 13-1. Motor Control Box Exploded Diagram.

Motor Control Box Assembly

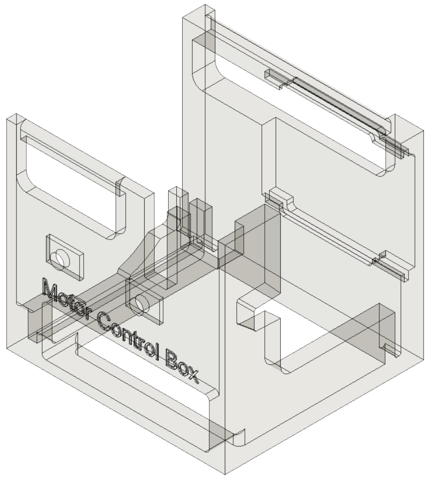

Figure 13-2. Place Motor Control Box with text upright.

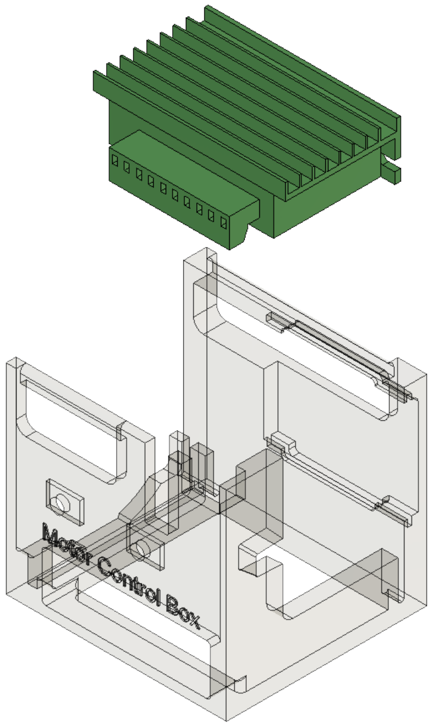

Figure 13-3. Adjust Stepper Motor Driver DIP Switches for Compressor or Pump. See Circuit Diagrams.

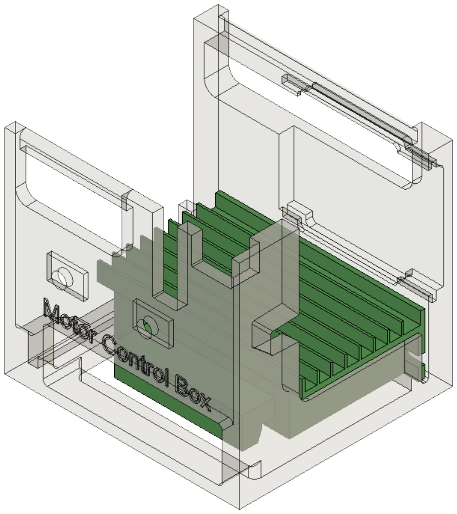

Figure 13-4. Slot in Stepper Motor Driver.

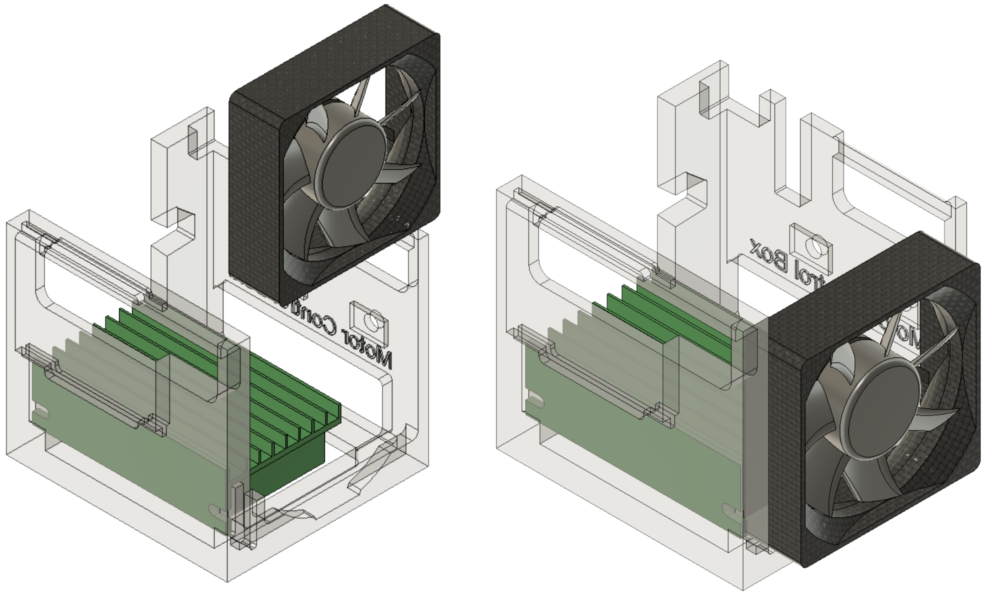

Figure 13-5. Slot in Electric Fan.

Motor Control Box Assembly

The Shield Kit makes conductive joints between the Arduino and the Stepper Motor controller using simple push-in spring connectors without soldering. Similarly, Lever Wire Nut Connectors connect wires to ensure a stable, protected wire circuit. You may find it easier to wire the circuits first and then assemble the components into the Motor Control Box or Wire the circuit after assembly.

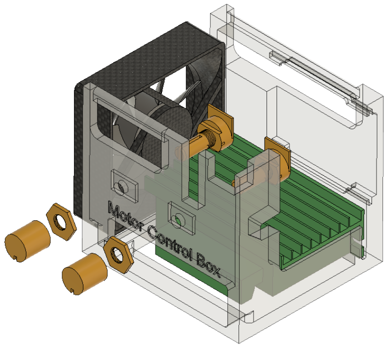

Figure 13-6. Push Potentiometers through knob holes.

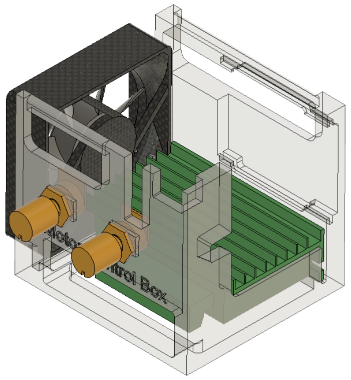

Figure 13-7. Secure nut and knob onto each Potentiometer.

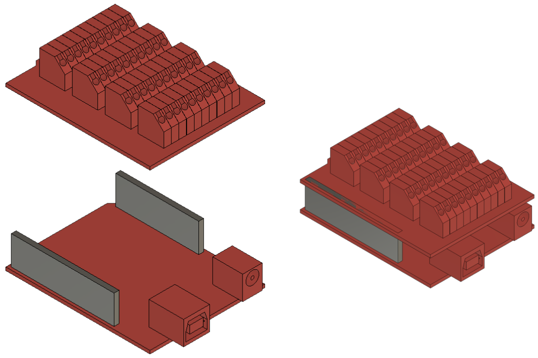

Figure 13-8. Mate the Shield Kit onto the Arduino, ensuring pins are aligned and not bend.

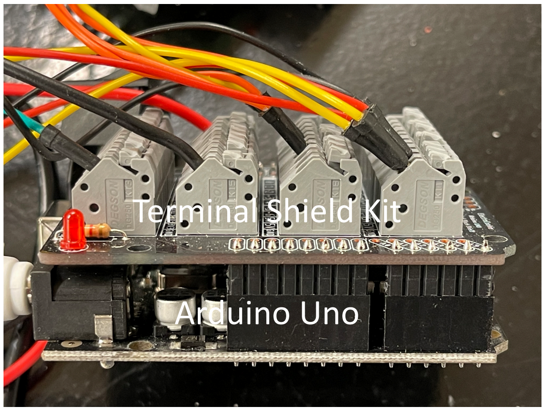

Figure 13-9. Photo of Shield Kit on top of Arduino Uno and wired to Shield Kit

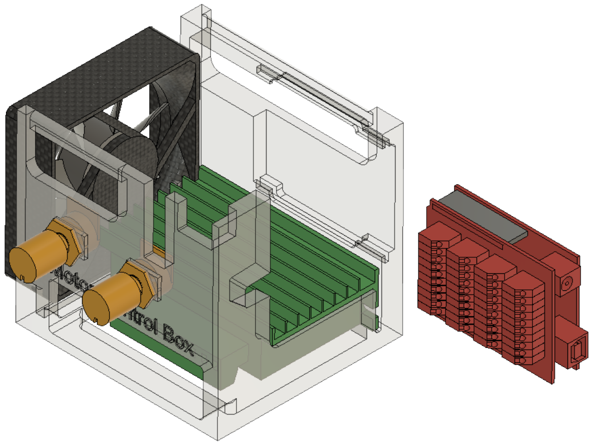

Figure 13-10. Slot Shielded Arduino into Motor Control Box with USB and power ports facing outward with text upright.

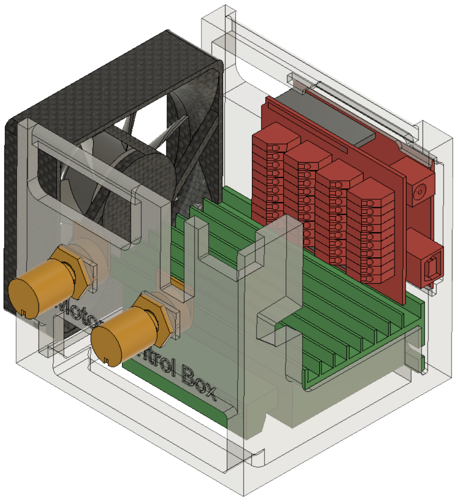

Figure 13-11. Double check wiring. Secure Lid

Compressor Circuit Diagram

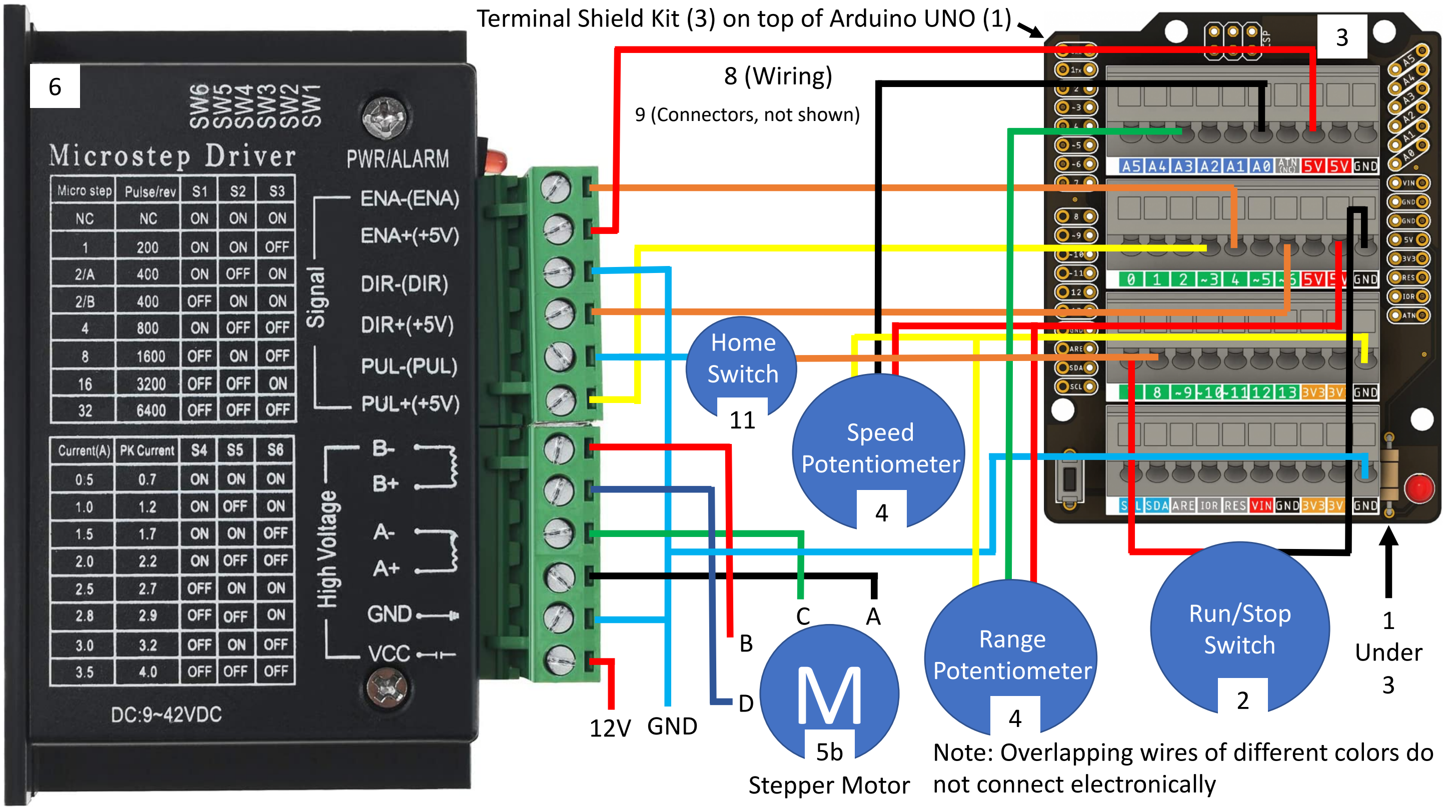

Figure 13-12. Compressor Circuit Diagram.

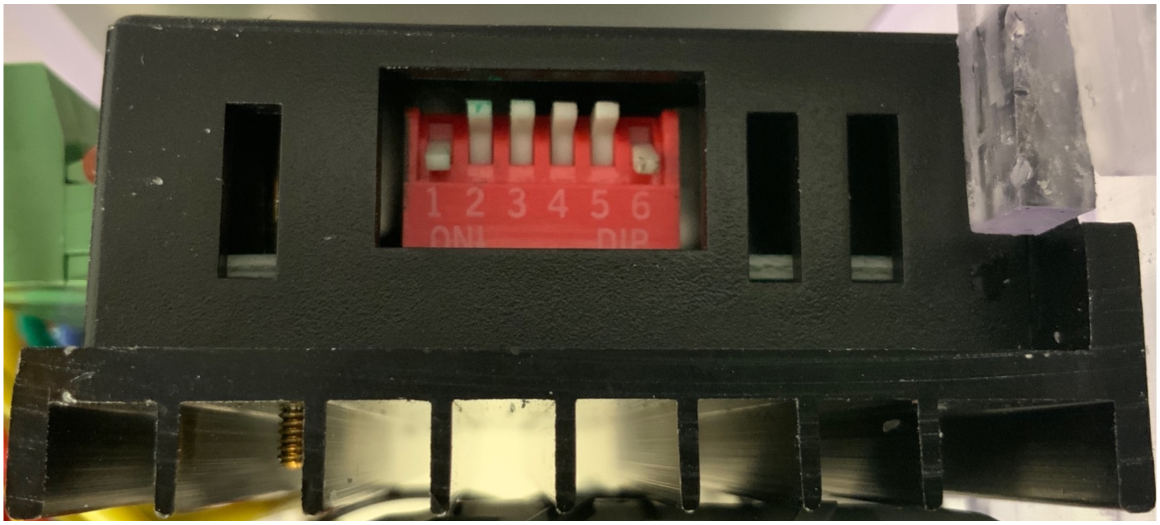

Figure 13-13. Driver DIP switch configuration for the Compressor Module Motor.

Peristaltic Pump Circuit Diagram

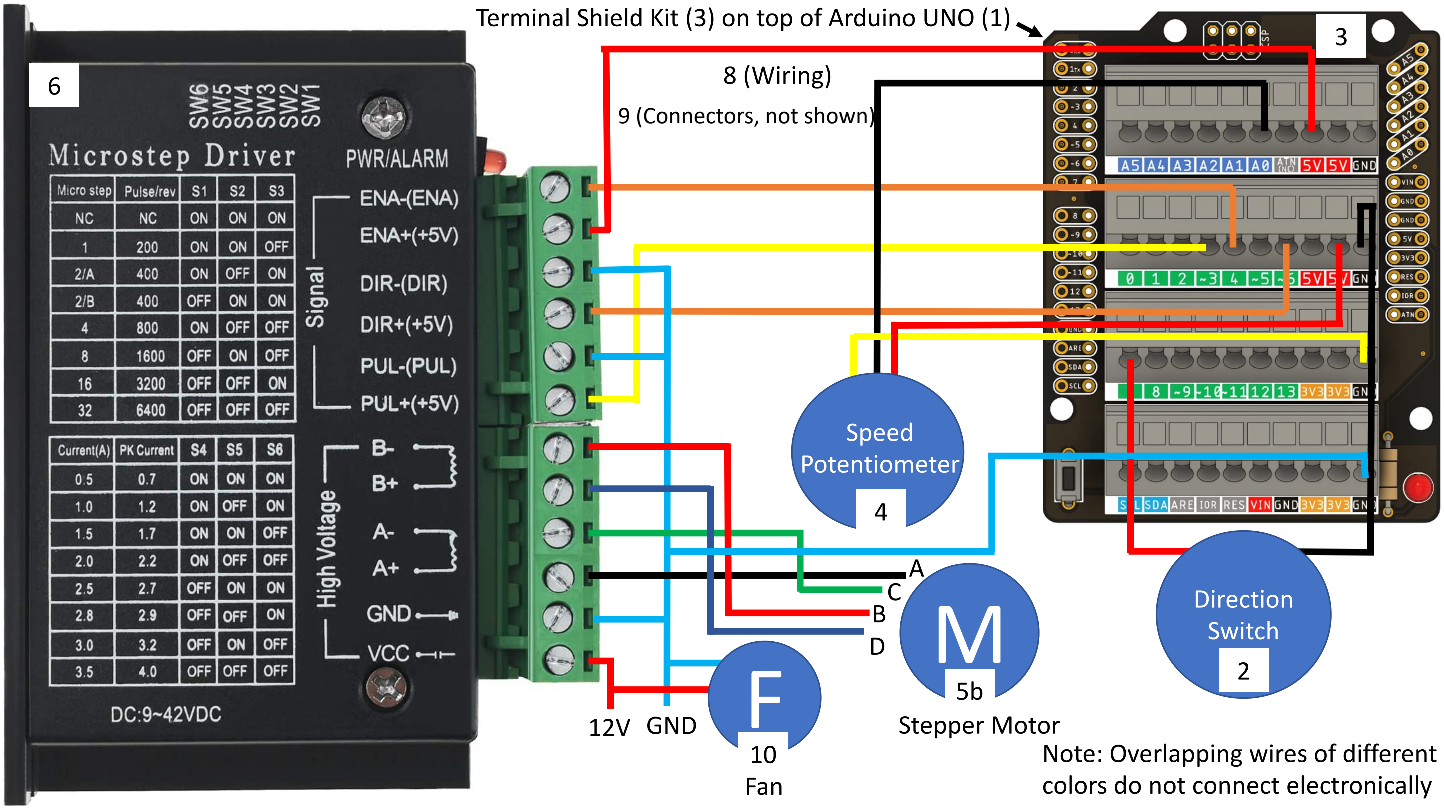

Micro step	Pulse/rev	S1	S2	S3
NC	NC	ON	ON	ON
1	200	ON	ON	OFF
2/A	400	ON	OFF	ON
2/B	400	OFF	ON	ON
4	800	ON	OFF	OFF
8	1600	OFF	ON	OFF
16	3200	OFF	OFF	ON
32	6400	OFF	OFF	OFF

Current(A)	PK Current	S4	S5	S6
0.5	0.7	ON	ON	ON
1.0	1.2	ON	OFF	ON
1.5	1.7	ON	ON	OFF
2.0	2.2	ON	OFF	OFF
2.5	2.7	OFF	ON	ON
2.8	2.9	OFF	OFF	ON
3.0	3.2	OFF	ON	OFF
3.5	4.0	OFF	OFF	OFF

Figure 13-14.Peristaltic Pump wiring diagram.

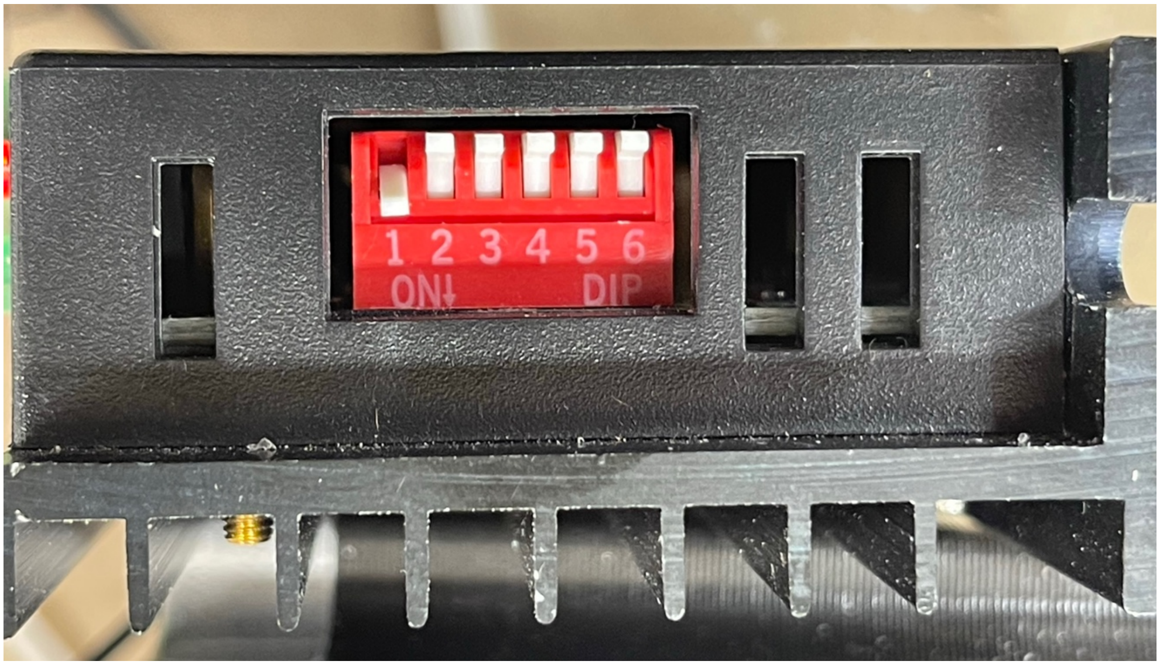

Figure 13-15. Stepper Motor Driver DIP switch configuration for Peristaltic Pump Motor Module.

Chapter 14. Motor Control and Calibration

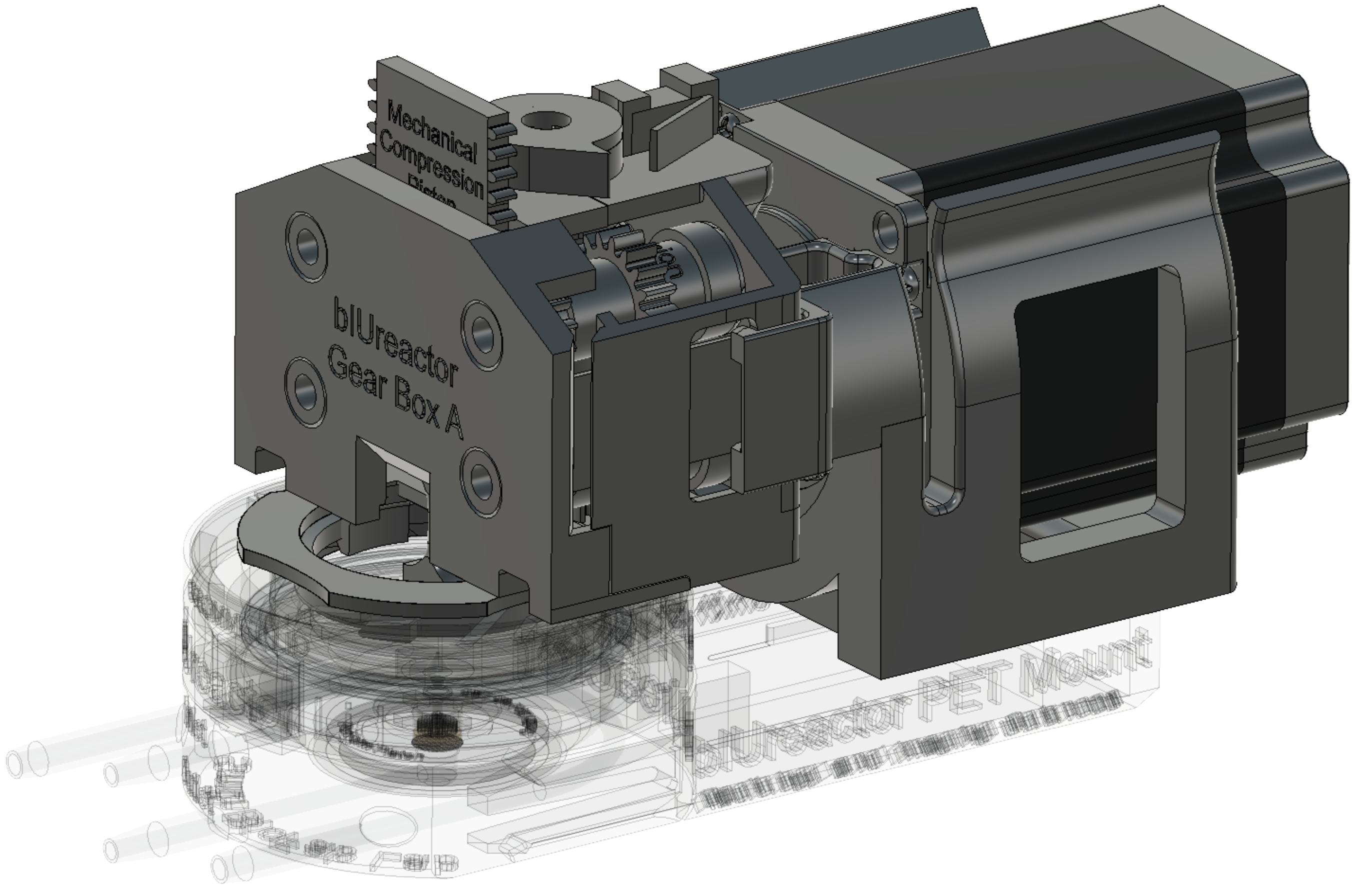

A calibration must be performed to set the piston displacement level (range) and frequency applied by the Compressor to the SSuPerForM Tissue.
A calibration must also be performed to set the flow rate of the Peristaltic Pump.

You will need:

1. Assemblies
    1. Motor Control Box for Compressor
    2. Motor Control Box for Peristaltic Pump
    3. Compressor Assembly

Compressor Calibration

A calibration must be performed to set the piston displacement level (range) applied by the Compressor to the SSuPerForM Tissue. Use caution when working with electricity. Follow the steps below to calibrate the Cyclic Mechanical Compressor.

1. Ensure the Motor Control Box is unplugged and turned off
2. Wire the Stepper Motor in the GearBox Assembly to the Motor Control Box as shown in the Compressor Circuit Diagram
3. Plug the Motor Control Box into power
4. Observe the position of the Displacement Potentiometer
5. Power on the Motor Control Box
6. Note the Motor will start as soon as the Motor Control Box is Powered on
7. Measure Piston displacement using digital Vernier calipers.
8. If the desired displacement is not achieved, turn off the Motor Control Box, adjust the Displacement Potentiometer, and measure again.
9. Repeat until the desired displacement is achieved. Note the Potentiometer position and mark it.
10. Once desired displacement is acheived, adjust the Speed Potentiometer in the same way until the desired number of cycles per minute is achieved.
11. Use a stopwatch to track the number of cycles per minute.

Peristaltic Pump Calibration

A calibration must be performed to set the flow rate of the Peristaltic Pump. Use caution when working with electricity. Follow the steps below to calibrate the Pump.

1. Ensure the Motor Control Box is unplugged
2. Wire the Stepper Motor in the Peristaltic to the Motor Control Box as shown in the Peristaltic Pump Circuit Diagram
3. Plug the Motor Control Box into power
4. Observe the positions of the Displacement and Speed Potentiometers
5. Power on the Motor Control Box
6. Note the Motor will start as soon as the Motor Control Box is powered on
7. Determine flow rate by measuring the amount of media that flows into a graduated cylinder in a minute’s time
8. If the desired flow rate is not achieved, turn off the Motor Control Box, adjust the Speed Potentiometer, restart the Motor Control Box, and measure again.
9. Repeat until the desired flow rate is achieved. Note the Potentiometer position and mark it.

Chapter 15. bIUreactor Research Platform Culture Experiment Preparation Instructions

You will perform:

1. bIUreactor Flow Circuit Assembly
2. bIUreactor Autoclaving
3. Preparation for SSuPerForM (Self-Supported Perfusion during Formation and Maturation) Seeding and Perfusion
4. Spheroid Production and Harvest
5. SSuPerForM (Self-Supported Perfusion during Formation and Maturation) Seeding and Perfusion

You will need:

1. Tubing
2. Luer Connectors (2 male, 2 female)
3. Needle-Free Valves (2x)
4. Barbed Connectors (2x)
5. bIUreactor Chamber Assembly
6. Calibrated Compressor wired to a Motor Control Box
7. Calibrated Pump wired to a Motor Control Box
8. Spheroid Maker Lid
9. Spheroid Maker Plate and Mold Base

bIUreactor Chamber Mode of Operation

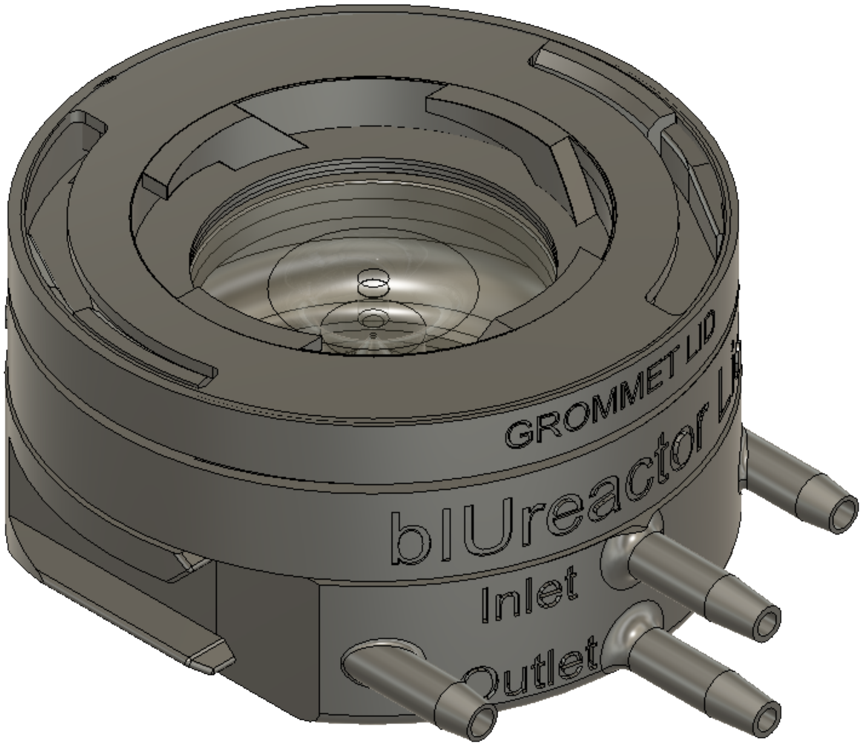

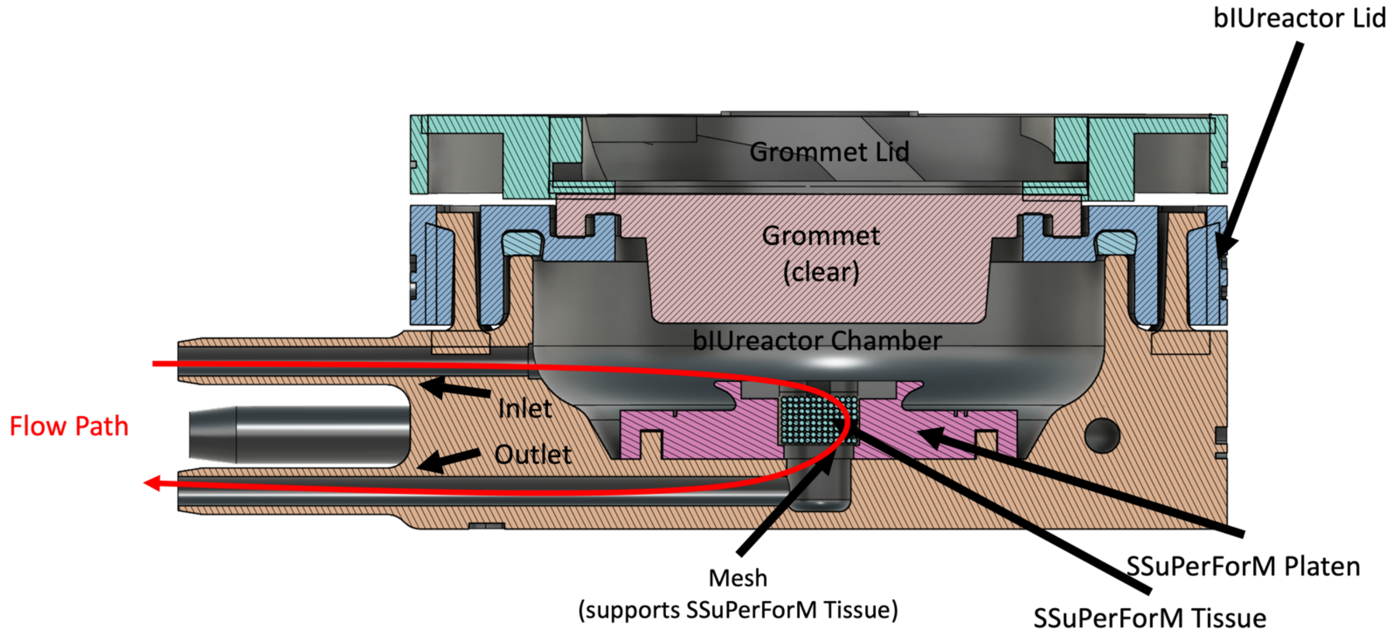

Making Tubing Set

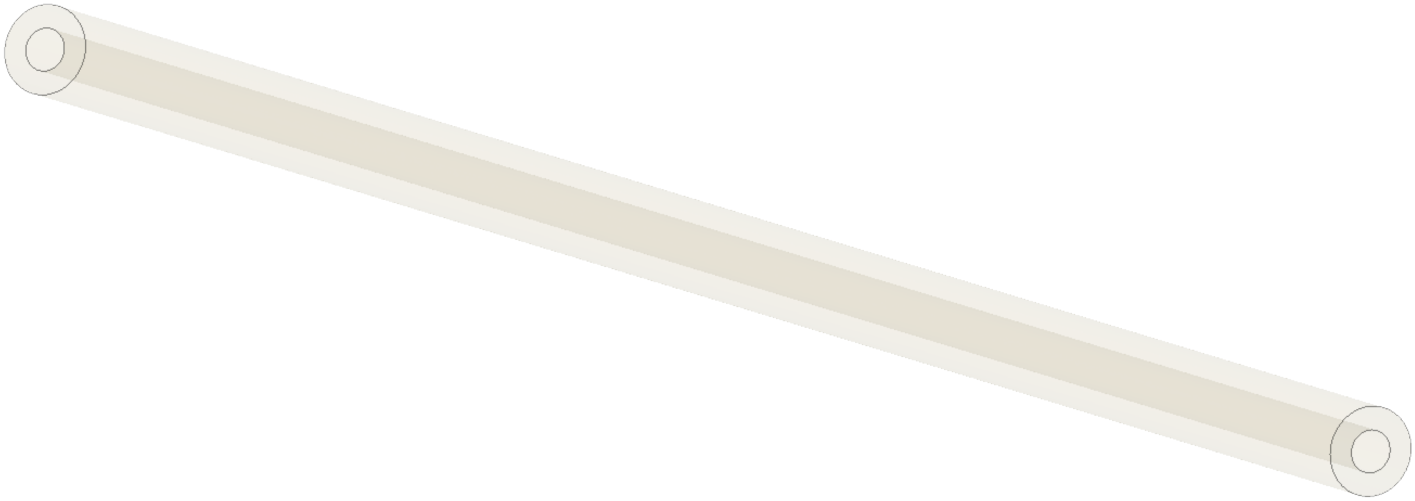

Figure 15-1. Tubing Section.

1. Cut tubing to length
    1. 1x Pump Section (10 cm)
    2. 2x Pump to Needle-Free Section (50 cm)
    3. 2x Needle-Free to Luer Section (50 cm)
    4. 2x Luer to Chamber (200 cm)

bIureactor Flow Circuit Assembly and Autoclaving

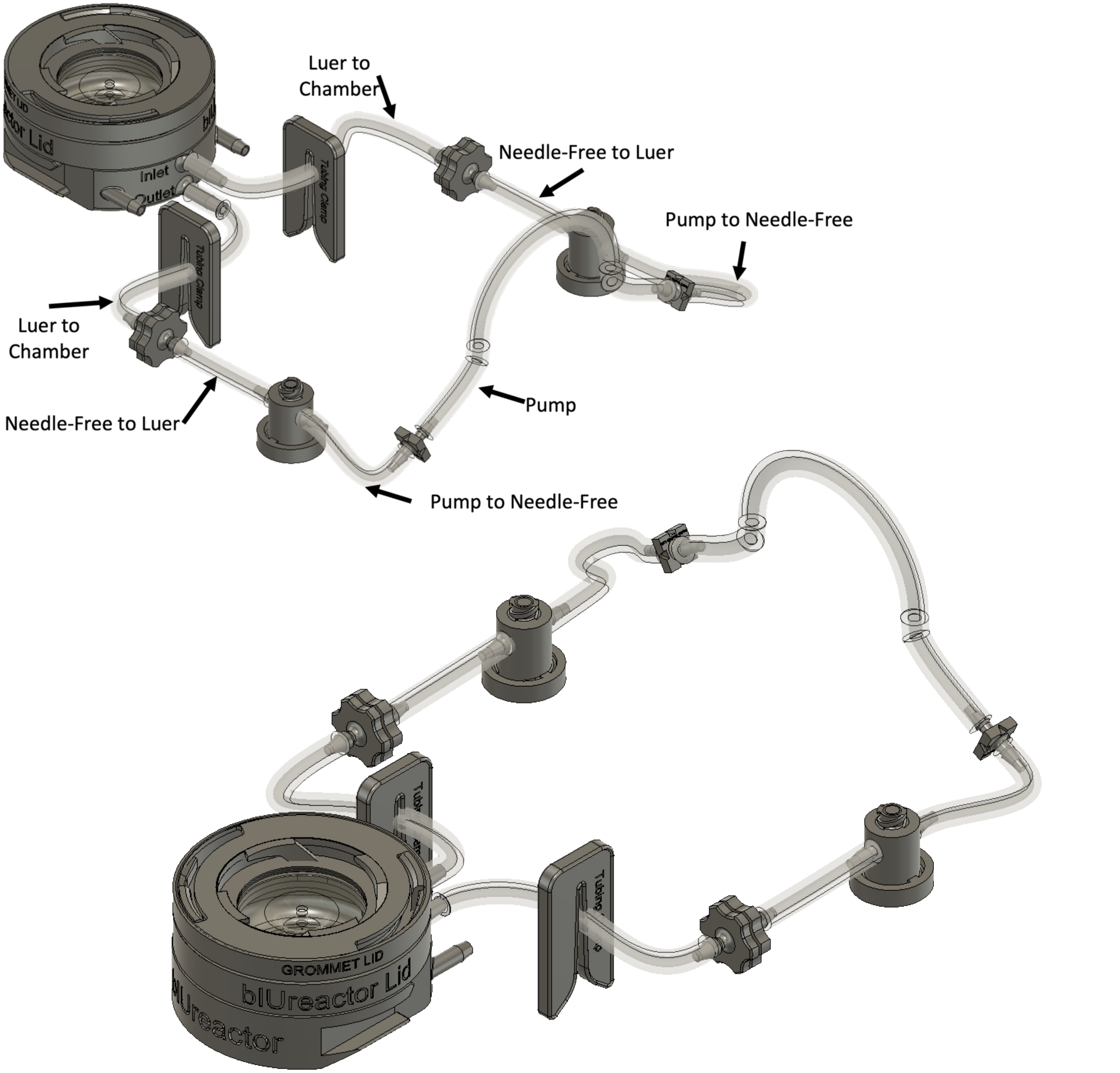

Figure 15-2. Two views of bIUreactor Flow Circuit.

1. Assemble Flow Circuit as shown
    1. Ensure the Platen, Mesh, and End Effector are installed in the bIUreactor Chamber
    2. Ensure the Lid Assembly is twisted loose but left resting on top of Chamber
2. Place in an autoclavable container
3. Autoclave 30 minute steam sterilization
    1. 250 Deg F (121 Deg C)

bIUreactor Preparation for Spheroid SSuPerForM Seeding

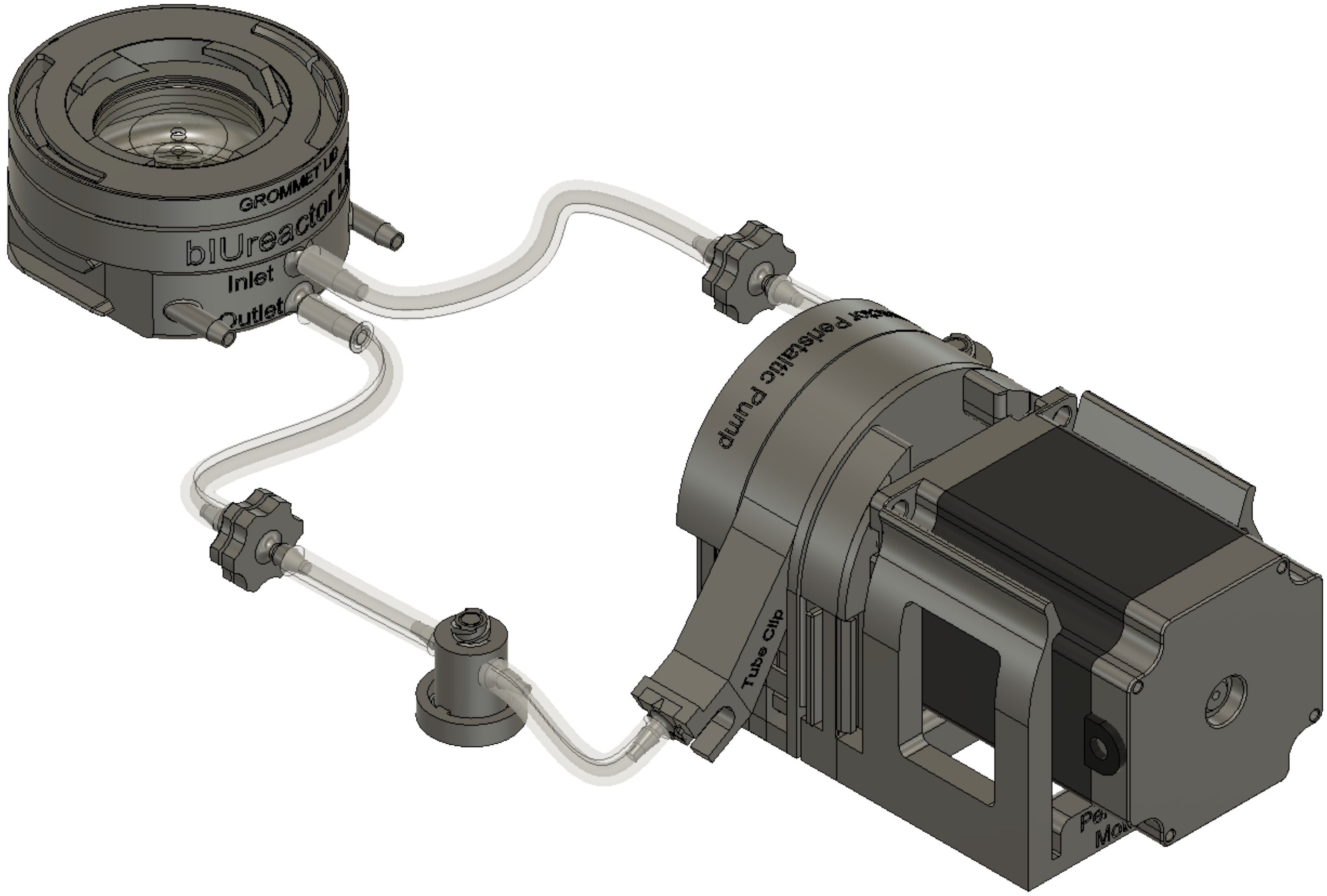

Figure 15-3. Flow circuit connected to Pump.

1. Allow to cool.
2. Aseptically transfer to a cell culture hood (biosafety cabinet).
3. Check that connections are tight.
4. Remove Tubing Clips.
5. Connect Tubing to Peristaltic Pump Assembly.
6. Remove bIUreactor lid.
7. Pump media through flow circuit at 10 ml/min.
    1. Ensure media is flowing into the Inlet and out of the Outlet.
8. Fill Chamber and Tubing with media (approximately 25 ml).
    1. You may need to pipette away bubbles that form during Chamber Priming .

SSuPerForM Seeding

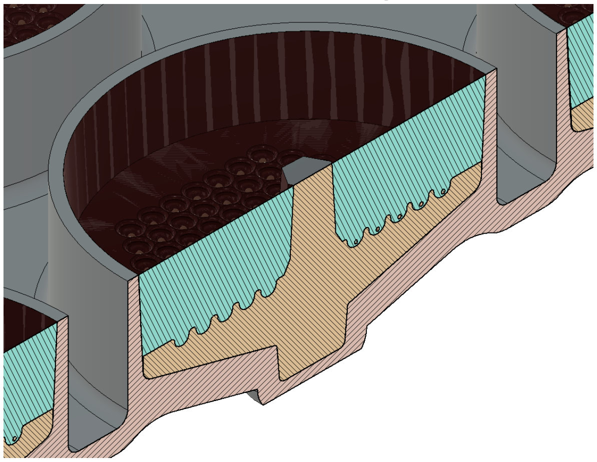

Figure 15-5. Cellular Spheroids form in microwells overnight.

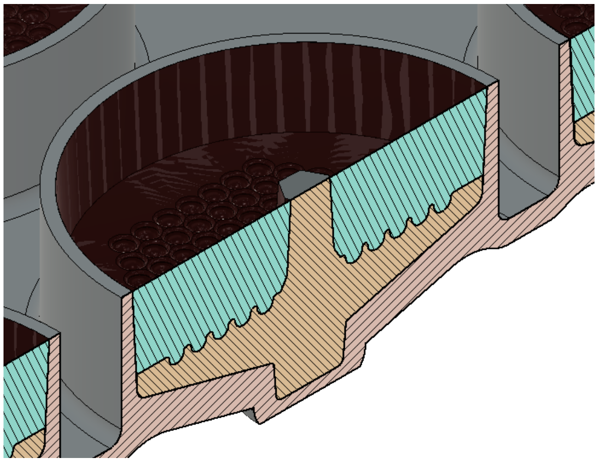

Figure 15-6. Seed Cellular suspension into Spheroid Maker. Each Spheroid Maker has 120 microwells. Titration may be necessary to produce 500 µm Spheroids. Cover with Lid. Centrifuge at 500xg for 5 minutes.

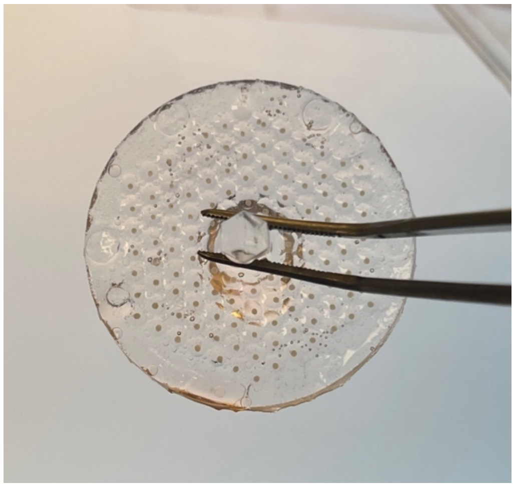

Figure 15-7. Photo of Spheroids within Spheroid Maker.

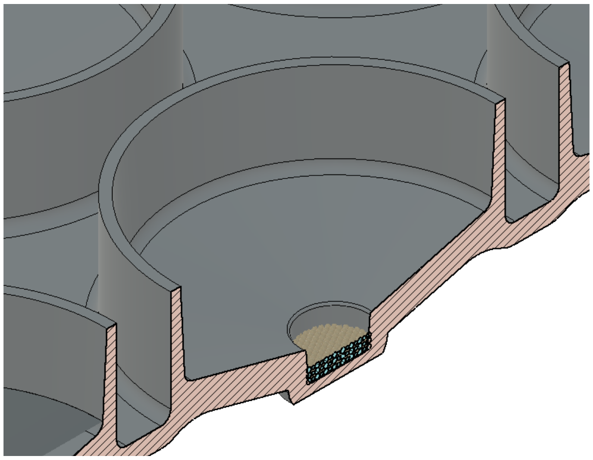

Figure 15-8. Spheroids will fall into the center of the well. Remove Spheroid Maker. Spheroids are ready for Harvest. For next steps, see the chapter on Spheroid Seeding and SSuPerForM Production.

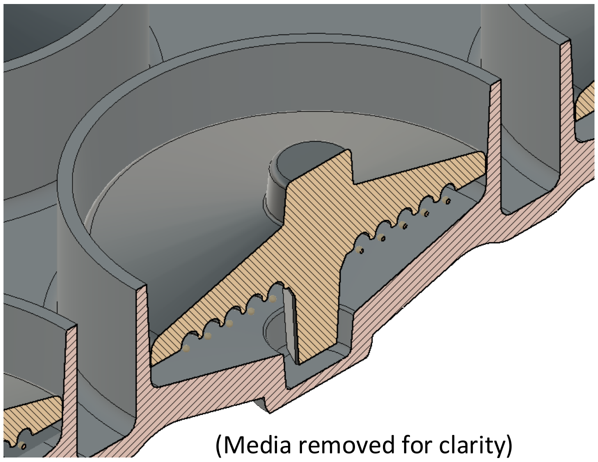

Figure 15-9. Invert Spheroid Makers with sterile forceps. Centrifuge covered at 500xg for 5 minutes.

Spheroid Harvest, Seeding, and SSuPerForm Production

1. With Lid Assembly removed and pump still running, pipette centrifuged Spheroids from the well center into the center of the Platen
    1. All Spheroids can be pipetted one well at a time and transferred
2. Ensure Spheroids are resting on the Mesh
3. This completes SSuPerForM production
4. Carefully secure the Lid Assembly to the Chamber
5. Proceed to transferring the bIUreactor to an incubator (See Chamber Transfer and Perfusion Chapter)

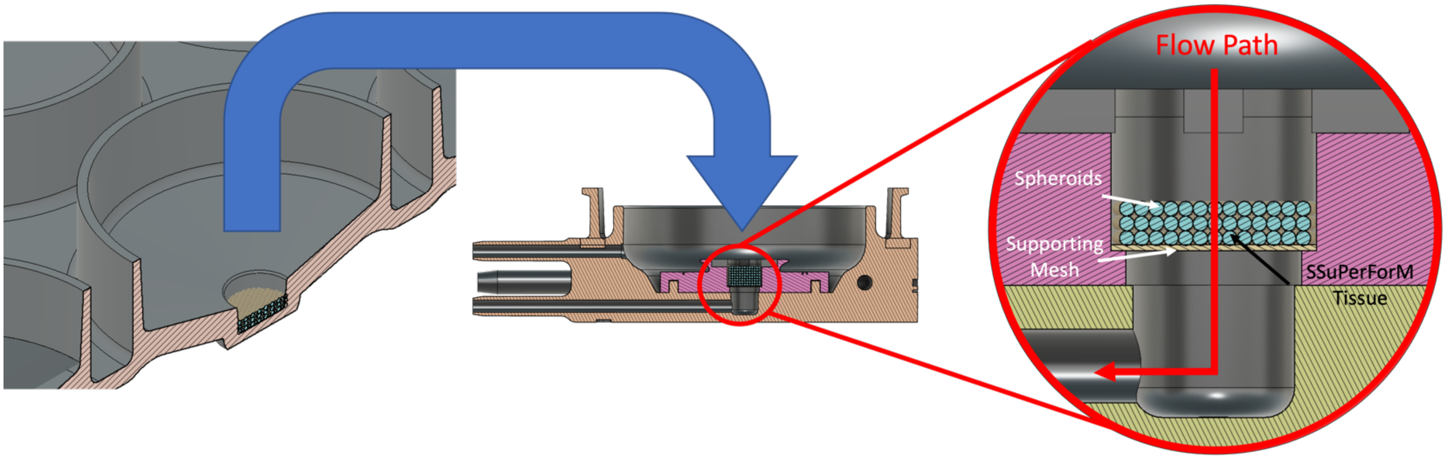

Figure 15-10. SSuPerForM production process.

Chamber Transfer and Perfusion

Transferring the bIUreactor Assembly to and from the incubator and other locations poses a risk of fluid in the flow circuit disrupting the tissue. Following the steps below will minimize risk of tissue damage during transfer. This task may require the cooperation of two people.

1. Stop Pump
2. Secure Tubing Clamps onto Tubing proximal to the Chamber (As shown in the bIUreactor Flow Circuit Assembly and Autoclaving Section)
    a. Tubing Clamps prevent tissue-disrupting backflow during transfer
3. Gently spool the Tubing for easy handling
4. Transfer bIUreactor to an incubator set to desired culture conditions
    a. Be sure to keep the Chamber level. A rolling cart can be used to assist with this.
5. If transferring to or from an incubator, first place the chamber onto the rack, then unspool the Tubing
6. Hold the Chamber still and put the Pump Section of the Tubing into the back opening of the incubator
7. While holding the Chamber steady, have another person slowly pull the tubing through the back opening until the tubing is fully extended
8. Connect Tubing to Pump
9. Check that the Tubing is connected to the correct Pump Head and the Pump is set to the correct flow rate
10. Remove Tubing Clamps
11. Start Pump

Chapter 16. Cyclic Mechanical Compression Setup

Cyclic Mechanical Compression setup allows simultaneous tissue perfusion and cyclic mechanical compression.

You will perform:

1. End-Effector Taring

You will need:

1. bIUreactor Chamber Assembly
2. Compressor Assembly
3. Peristaltic Pump Assembly
4. Motor Control Box Assembly

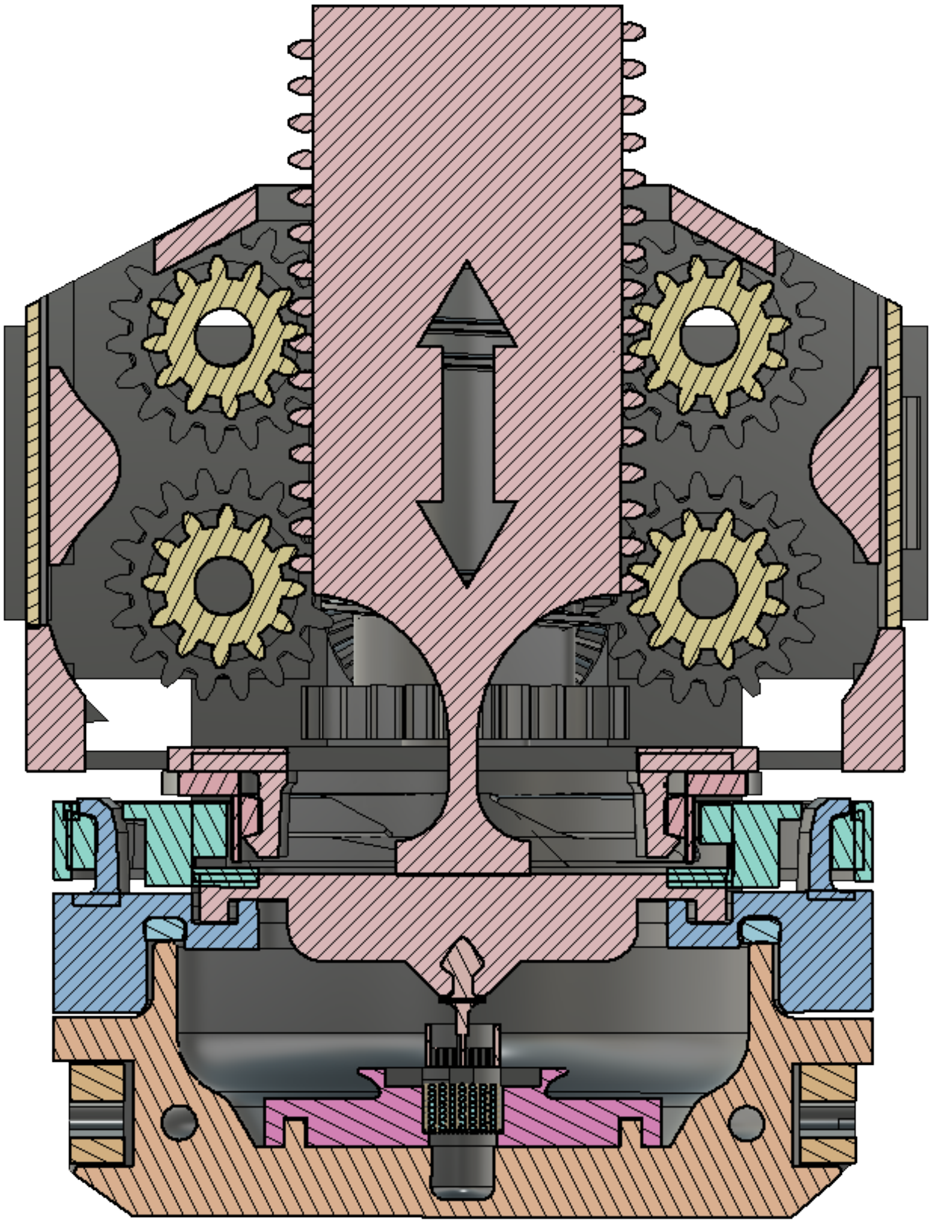

Compressor Mode of Operation

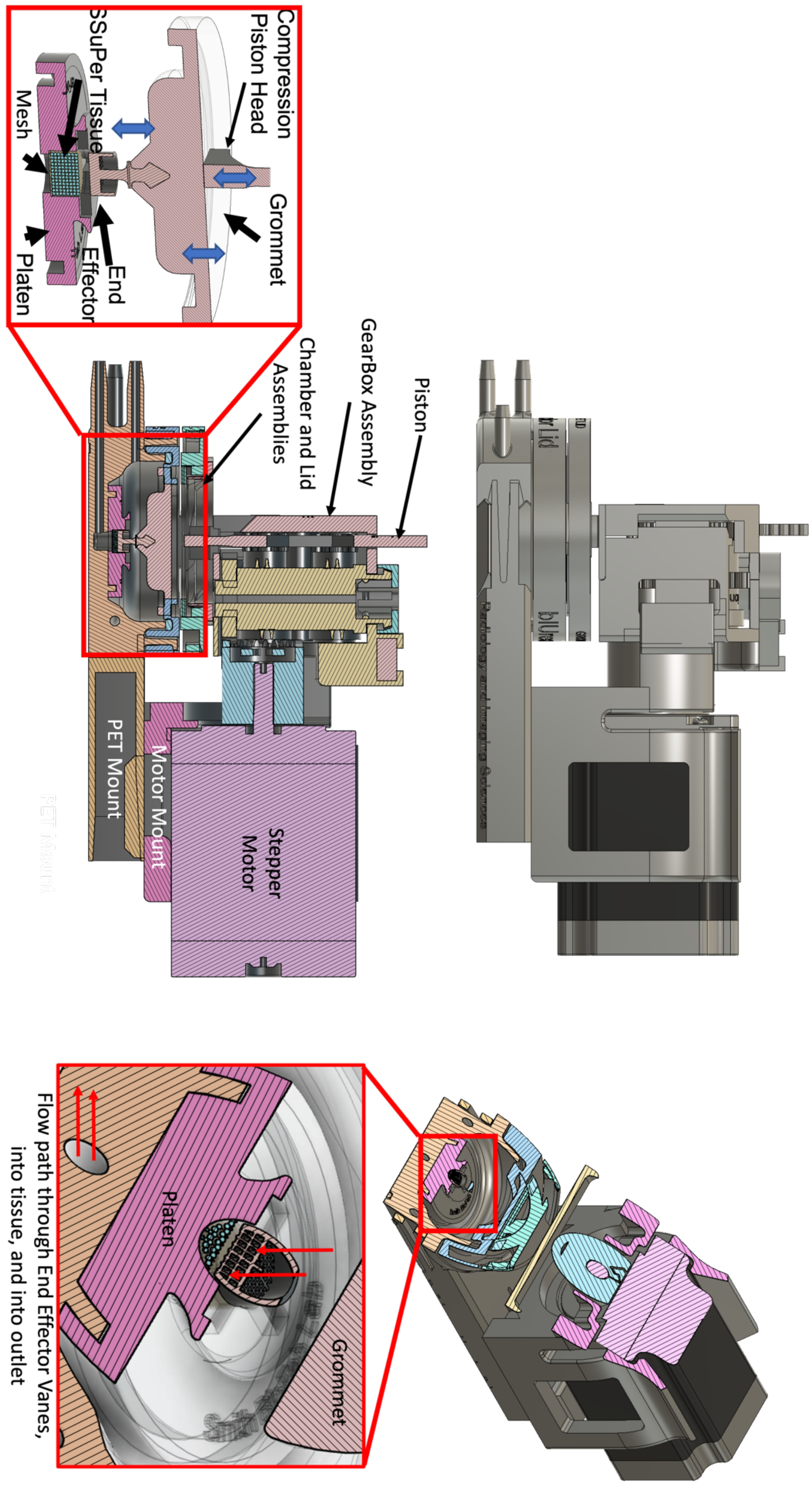

Figure 16-1. Compressor Mode of Operation.

Gearing

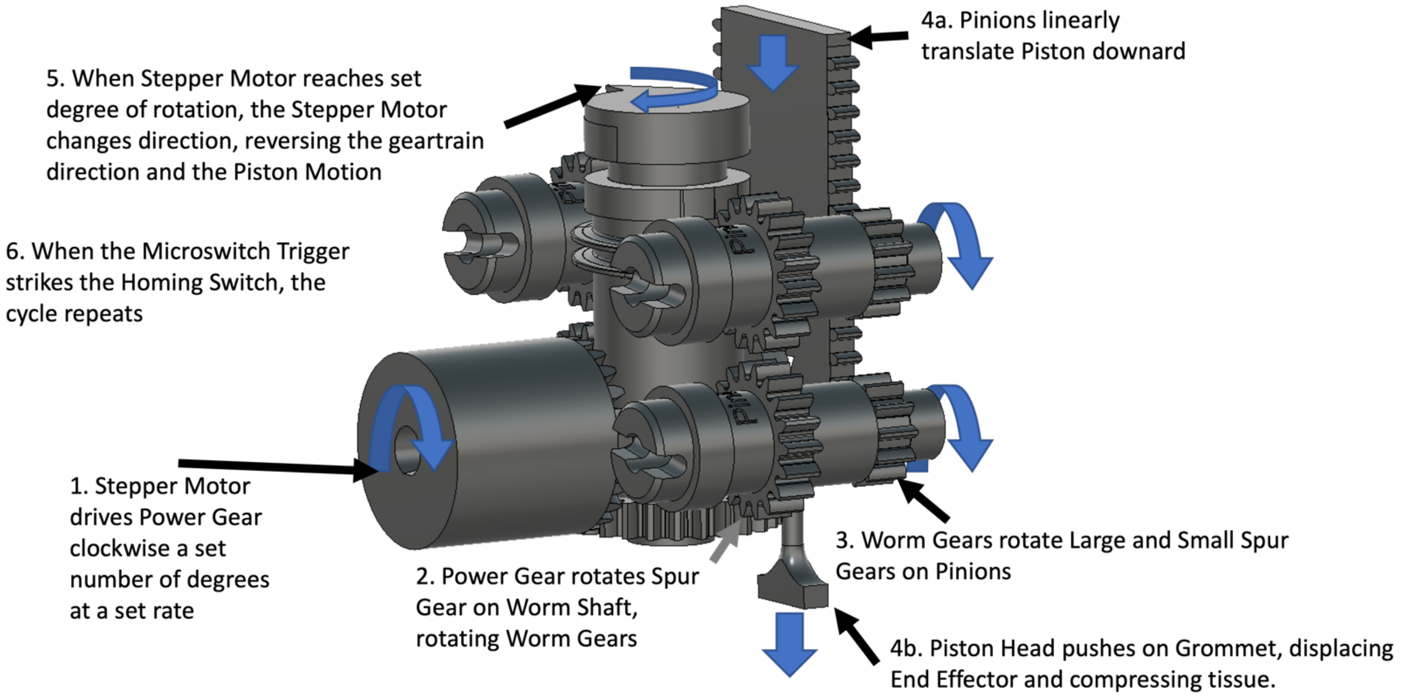

Figure 16-2.Image of Gearbox gearing. The Piston translates linearly 2.5 mm every 360 degrees of Worm Gear Rotation.

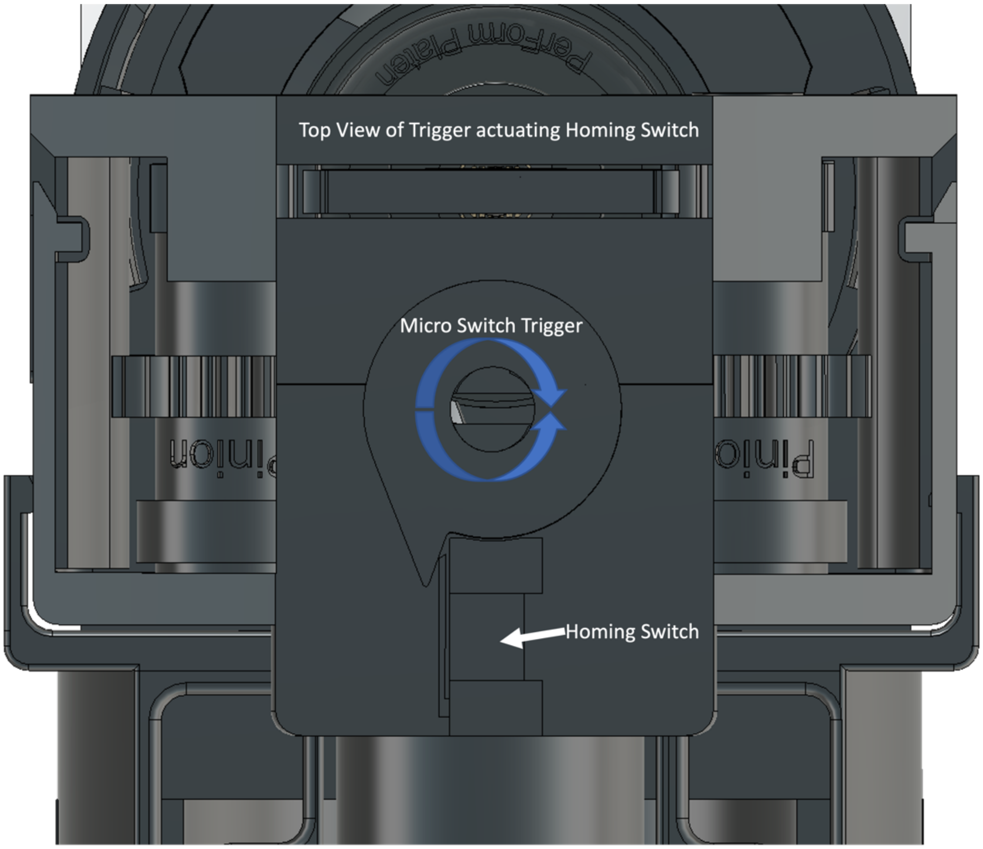

Figure 16-3. Top view of Gearbox assembly showing Microswitch Trigger as it actuates Homing Switch.

Taring End-Effector to top of SSuPerForM Tissue

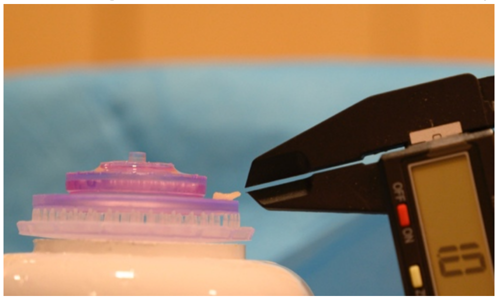

Figure 16-4. Measure tissue height using Vernier Calipers. See SSuPerForM Tissue Removal for information on harvesting the tissue from the Chamber.

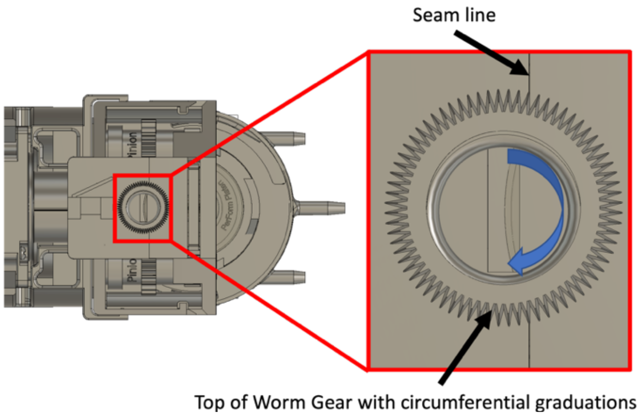

Figure 16-6. Top of Worm Gear (Worm Shaft) the User Rotates to set the End-Effector Position.

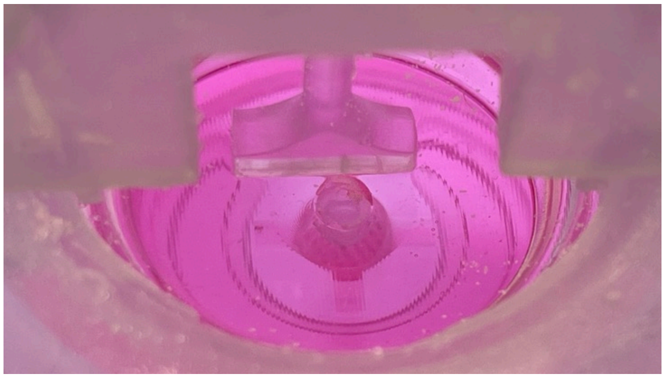

Figure 16-7. View through the Grommet Window. The User peers through the window while adjusting the End-Effector height.

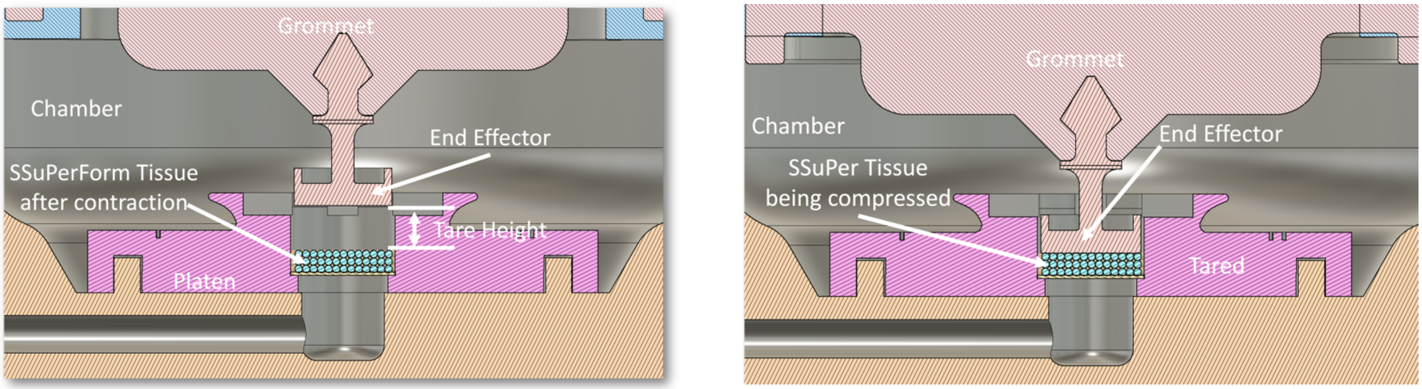

Figure 16-5. The Effector is pushed down by twisting the Top of the Worm Shaft until the End-Effector travels the distance of the Tare Height (TH), contacting the top of the tissue.

1. The tissue contracts to a specific height (SH) after a day of culture. Perform trial culture experiments to determine this height. (See Figure 12-3).
2. Rotate the Worm Gear by hand so the top ridge of the End Effector is level with the top of the Platen (See Figure 12-4)
    1. Use the graduation marks on top of the Worm Gear and the seam line on the GearBox to mark rotation.
    2. The Worm Gear can be turned by hand or with a flat head screwdriver.
    3. You can check this by observing the tissue through the Grommet Window.
    4. At this stage, the distance from the bottom of the End Effector to the top of the Mesh (DEM) is 3.925 mm.
    5. Therefore, the Tare Height (TH) is DEM-SH.
3. Tare the Compressor a distance of the TH so the End Effector is in contact with the top of the tissue. (Figure 12-5)
    1. Do this by rotating the Worm Gear clockwise (360 degrees per 2.5 mm).

Chapter 17. Media Exchange

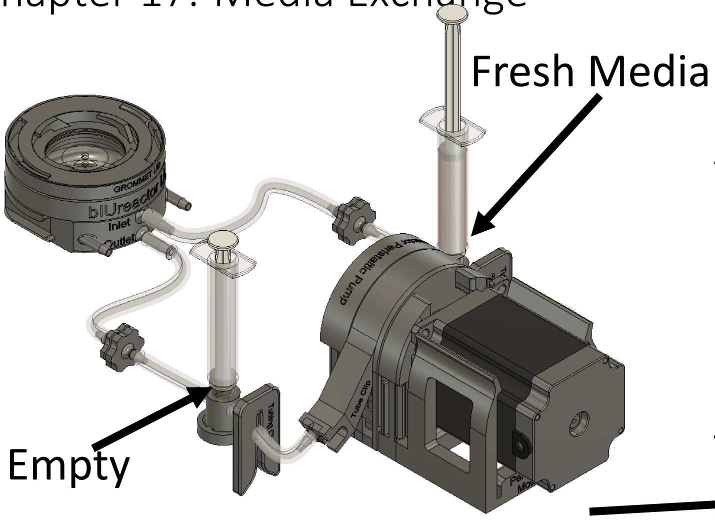

Figure 16-1. Flow Circuit at start of Media Exchange.

A Media Exchange may be necessary to exchange fresh and spent nutrients or add a reagent. It is important to follow the steps below to avoid disrupting the tissue and prevent overpressure.

1. Stop Pump
    1. Secure Tubing Clamps on the Inlet and Outlet sides of the Tubing Proximal to the Tube Clip
    2. The Clips prevent backflow into the Pump
    3. Attach a syringe with fresh media to the Inlet-side Needle-Free Valve and attach an empty syringe to the Outlet-side needle-free valve
2. Slowly pump fresh media into the flow circuit on the Inlet side while slowly drawing out spent media on the Outlet-side syringe at the same rate

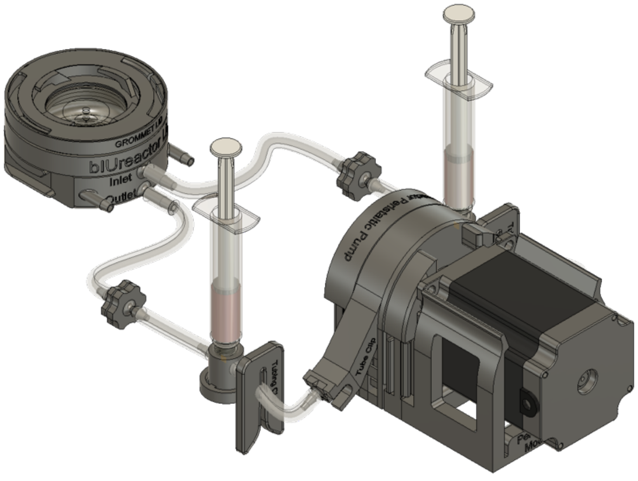

Figure 17-3. Flow Circuit during Media Exchange.

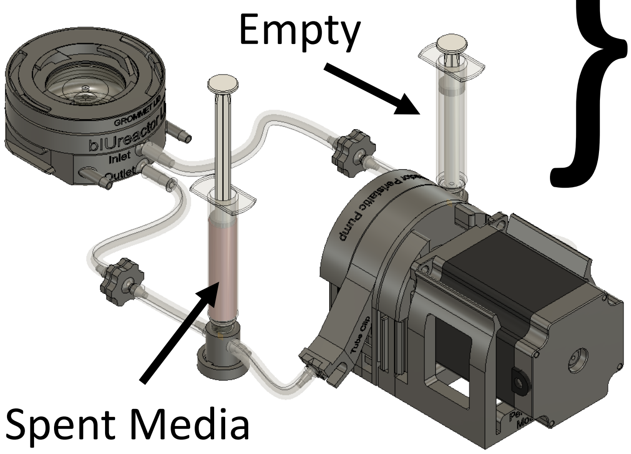

Figure 17-2. Flow Circuit at end of Media Ezxchange.

Chapter 18. Visual and Physical Tissue Access

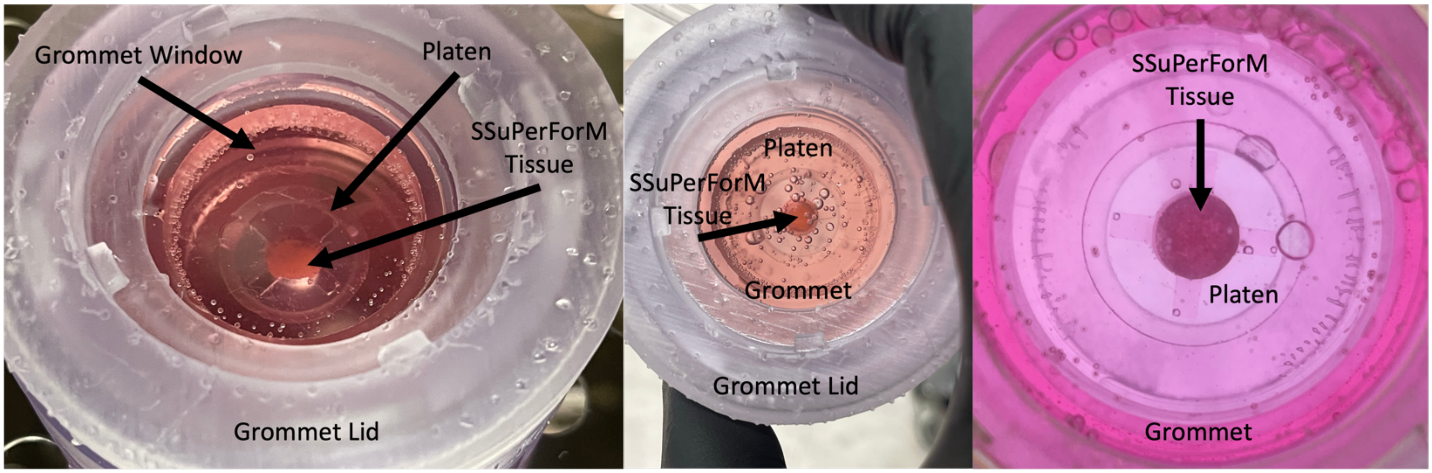

Figure 18-1. The tissue can be observed through the Grommet window during culture. If the User wants to aseptically access the tissue, they can place the bIUreactor in a cell culture hood and remove the Lid Assembly.

Chapter 19. Tissue Removal

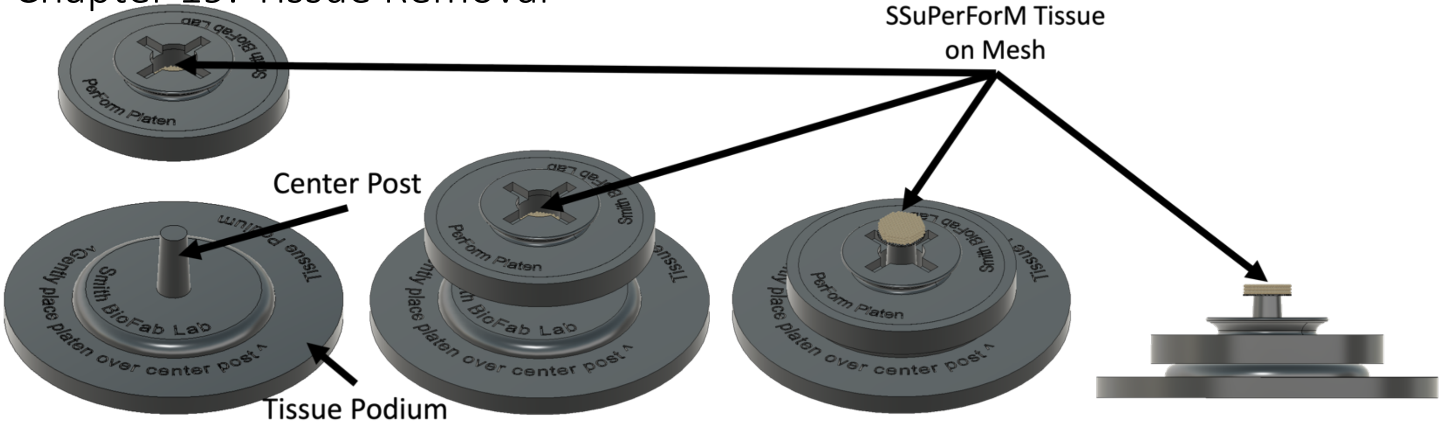

1. Use Forceps to remove the Platen containing the SSuPerForM Tissue from the Chamber and align the center of the Platen over the Center Post of the Tissue Podium using forceps.

2. Gently push the Platen down over the Center Post

3. The Center Post supports the Mesh-supported Tissue.

Forceps can be used to remove and manipulate the Tissue while minimizing damage risk.

Side view of the previous step showing the Center Post supporting the Mesh supporting the tissue.

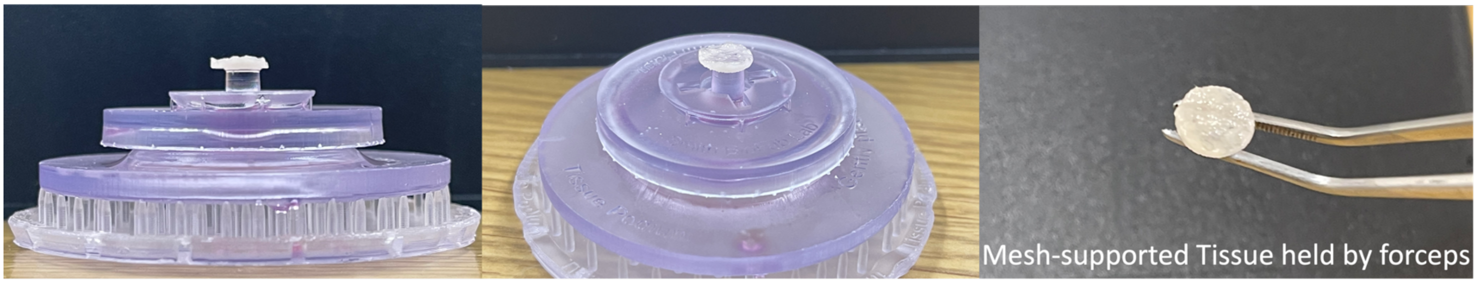

Figure 18-1. Tissue Podium being used to remove the SSuPerForM Tissue from the Platen.

Chapter 20. Cleanup

bIUreactor Cleanup

1. Drain all media.
2. Disassemble all printed and casted parts.
3. Wash printed and casted parts with soap and warm water and bottle brushes and toothbrushes.
4. Allow to air-dry before autoclaving.
5. Discard and replace both the Tubing and the Mesh.

Spheroid Maker Cleanup

1. Wash with Soap and Water using toothbrush.
2. Submerge in 3% hydrogen monoxide and scrub with toothbrush.
3. Soak overnight.
4. Wash with deionized water and toothbrush and shake out to remove all water.
