# Supplementary material for: The ‘bIUreactor’: An Open-Source 3D Tissue Research Platform
Source: Ann Biomed Eng. 2024 Mar 26;52(6):1678–92. doi: 10.1007/s10439-024-03481-5 (PMC11082015; doi:10.1007/s10439-024-03481-5)
Supplement: Supplementary file 5 — Supplementary file5 (PDF 74 kb) [file 10439_2024_3481_MOESM5_ESM.pdf]

Costs

Function	Item Name	Catalog No.	Price ($)	Number	Cost ($)	Vendor	Total Kit (all items)
3D Printing	Form 3B+ Basic Medical Package	PKG-F3B-WSVC-MSP-BASIC	$ 4,299.00	1	4299	Formlabs	$ 7,986.35
	Form 3B+ 3D Printer					Formlabs	Just 3D printing Equipment
	Form 3 Resin Tank V2.1					Formlabs	$ 5,648.00
	Build Platform					Formlabs	Just 3D printing supplies
	Form 3 Finish Kit					Formlabs	$ 2,338.35
	Form 3B Medical Professional Service Plan 1 Year					Formlabs	Just 3D printing Parts (~1.3 L)
3D Printing Materials	BioMed Clear Resin 1L	RS-F2-BMCL-01	$ 349.00	2	698	Formlabs	$458
	Clear V4	RS-F2-GPCL-04	$ 149.00	3	447	Formlabs	
3D Printing Post processing	Form Wash	FH-WA-01	$ 599.00	1	599	Formlabs	
	Form Cure	FH-CU-01	$ 750.00	1	750	Formlabs	
	Isopropanol (2-propanol>99%)	PX1830-4	$ 161.32	1	161.32	VWR	
Silicone Casting	Sylgard 184 (Kwik Gard Sylgard Dispenser)	50-190-1221	$ 220.66	1	220.66	Fisher	
Perfusion	Spectra/Mesh 145882 Screen Discs, Polypropylene, 90 mm, 149 µm, 10/pk	145775	$ 149.00	1	149	Cole-Parmer	
	Tygon Transfer Tubing, BioPharm Platinum-Cured Silicone, 1/16" ID x 1/8" OD; 50 Ft	F00002	$ 161.00	1	161	Cole-Parmer	
GearBox Motor	STEPPERONLINE High Torque Nema 23 CNC Stepper Motor 114mm 425oz.in/3Nm CNC Mill Lathe Router		$ 39.99	1	39.99	Amazon	
Peristaltic Pump Motor	STEPPERONLINE High Torque Nema 23 CNC Stepper Motor 114mm 425oz.in/3Nm CNC Mill Lathe Router		$ 39.99	1	39.99	Amazon	
Motor Control	ELEGOO UNO R3 Project Most Complete Starter Kit (Contains R3 Arduino, Switch for the Arduino, and spare components)		$ 59.99	2	119.98	Amazon	
	Arduino Uno R3 compatible Electrocookie Uno Terminal Block Shield Kit with push-in spring connectors (Shield Kit)		$ 19.99	2	39.98	Amazon	
	TWTADE 3pcs 10k ohm linear taper adjustable rotary potentiometer (wh148 b10k 3 pin with xh2.54-3p connector wire cable) and black knob cover caps 148-10kbk		$ 10.99	1	10.99	Amazon	
	Usongshine Stepper Motor Driver TB6600 4A 9-42V Nema 17 Stepper Motor Driver CNC Controller Single Axes Phase Hybrid Stepper Motor for CNC/42 57 86 Stepper Motor3 pcs)	TB6600	$ 25.99	2	51.98	Amazon	
	Fermerry 18AWG Silicone Wire Hook up Wire Kit Stranded Tinned Copper Wire 18 Gauge 6 Colors 10Ft Each Electrical Wire (10 FT 6 Colors Each, 18AWG)		$ 15.99	1	15.99	Amazon	
	Glutoad Lever Wire Nut Connectors, 20 Pcs 1 Conductor Inline Wire Connectors, Compact Splicing Electrical Connector for Quick Connect 28-12 AWG Wires		$ 11.99	1	11.99	Amazon	
	80mm Moisture-Proof Fan 2-Pack 12V DC 8025 High Airflow Cooling Fan 2 Wire 3Pin 3500RPM		$ 15.99	2	31.98	Amazon	
	KAIWEETS Self Adjusting Wire Stripper - 3 in 1 Heavy Duty Automatic Wire Stripping Tool | 10-24 AWG Wire Cutter for Electrical Cable Cutting, Crimping Tool		$ 16.99	1	16.99	Amazon	
	Adjustable Power Supply with 2.1mm / 5.5mm DC - 3V to 12V at 5A (Power Supply, Adafruit, NY, NY, USA)	4800	$ 17.50	2	35	Adafruit	
	MUZHI SPDT 1NO 1NC Hinge Lever Momentary Push Button Micro Limit Switch AC 5A 125V 250V 3 Pins 12 Pcs (Style 4)		$ 6.69	1	6.69		
Lubricant	Glycerol	G2025-100ML	$ 65.50	1	65.5	Sigma-Aldrich	
Cleaning	Amazon Brand - Solimo Orbit Toothbrushes, 4 Count (Toothbrushes for cleaning parts and brushing on glycerol)		$ 6.33	1	6.33	Amazon	
	16 Pcs Extra Long Straw Cleaning Brush Nylon Cleaning Bottle Brush Cleaning Brushes Tube Washing Cleaner Kit Tool Reusable Bottle Straw Brush in Different Size (Blue-Black,16 Pieces)		$ 7.99	1	7.99	Amazon	

Resin Consumption

bIUreactor Group Name	No. of items	Item name	Item total (mL)	Group Total (mL)
Chamber Group	1	Twist Tab bIUreactor Lid	23.48	
	1	Grommet Lid	18.86	
	1	SSuPerForM Platen	7.15	
	1	bIUreactor Chamber	58.4	
	1	PET Mount	48.55	
Total				156.44
GearBox Group	1	Gear Box B (motor side)	38.41	
	1	Gear box A (Mount side)	36.42	
	1	Gear Box Mount	18.4	
	1	Power Gear	11.53	
	1	Piston	11.65	
	4	Pinion	31	
	1	Switch Key	3.3	
	1	Worm Shaft	19.53	
Total				170.24
Seal Mold Group	1	Gasket Mold and Lid	19.74	
	1	Grommet Actuator Insert	6.83	
	1	Grommet Mold	29.12	
Total				55.69
Spheroid Maker Group	2	Spheroid Maker 6-Well Plate	177.26	
	2	Spheroid Maker 6-Well Plate Lid	125.7	
Total				302.96
Peristaltic Pump Head Group	1	Back Rack	14.81	
	1	Combined Tube Clip	31.5	
	1	Front Rack	17.64	
	1	Key	9.31	
	1	Key Snap Ring	7.52	
	1	Plain Bearing	6.09	
	1	Pump Head Base	76.11	
	8	Roller	14.4	
	2	Tubing Clamp	17.44	
Total				194.82
Pump Motor Group	1	Motor Key	13.54	
	1	NEMA 23 Motor Mount	76.6	
	16	Barbed Connector	26.53	
	6	Barbed Luer Female	15.36	
	6	Barbed Luer Male	17.97	
Total				156.24
Motor Control Box	2	Motor Control Box	204	
	2	Motor Control Box Lid	71.77	
Total				275.77
Total volume (ml)				1312.16
Total Cost ($349/L of BioMed Clear)				457.94384
